# ISGylation of γH2AX retains MDC1 and facilitates homologous recombination repair causing radioresistance in esophageal adenocarcinoma

**DOI:** 10.1016/j.jbc.2026.111358

**Published:** 2026-03-09

**Authors:** Archismaan Ghosh, Paramita Ray, Dafydd Thomas, Vinay Jeeyar, Shreya Pillai, Venkatesha Basrur, Irina V. Bondarenko, Emily Bellile, David H. Wang, Meredith A. Morgan, Qiang Zhang, David G. Beer, Kiran H. Lagisetty, Theodore S. Lawrence, Dipankar Ray

**Affiliations:** 1 Department of Radiation Oncology, The University of Michigan Medical School, Ann Arbor, Michigan, USA; 2 Department of Pathology, The University of Michigan Medical School, Ann Arbor, Michigan, USA; 3 Department of Biostatistics, The University of Michigan Medical School, Ann Arbor, Michigan, USA; 4 VA Medical Center, Ann Arbor, Michigan, USA; 5 Department of Surgery, The University of Michigan Medical School, Ann Arbor, Michigan, USA

**Keywords:** ISGylation, H2AX, homologous recombination repair (HRR), radioresistance, esophageal adenocarcinoma

## Abstract

High expression of interferon-stimulated gene 15 (ISG15) has been associated with poor survival in patients with esophageal adenocarcinoma (EAC). Like ubiquitin, ISG15 utilizes its C-terminal LRGG motif to post-translationally modify target proteins through a process called ISGylation, thereby influencing their stability, function, and interaction networks. Given ISG15’s role in the replication stress response, we hypothesized that it may also contribute to DNA repair mechanisms. We found that ISG15 is upregulated following ionizing radiation (IR), and its knockdown disrupts the IR-induced G2/M checkpoint, leading to increased radiosensitivity in EAC cells. In synchronized cells, ISG15 expression peaks during the S/G2 phases. Knockdown of *ISG15* impairs homologous recombination repair (HRR) with compensatory upregulation of non-homologous end joining (NHEJ). Similarly, cells expressing an ISGylation-defective ISG15^LRAA^ mutant exhibit reduced HRR activity and elevated NHEJ, highlighting the critical role of ISGylation in the DNA damage response (DDR). Further investigation revealed that IR-induced ISG15 modifies γH2AX at lysine 120 (K120). Overexpression of an H2AX^K120R^ mutant in EAC cells resulted in diminished MDC1 retention at DNA damage sites, mirroring the phenotype observed with *ISG15* knockdown. Additionally, depletion of ISG15 delays RAD51 foci formation at damage sites. Using a tissue microarray of chemoresistant EAC patients, we observed that ISG15 is expressed in almost all cases and, along with high RAD51 expression, correlates with poorer prognosis in node-positive patients. Collectively, we identify γH2AX as a novel substrate of IR-induced ISGylation, which facilitates efficient recruitment and retention of downstream HRR proteins and may contribute to radioresistance in EAC.

Esophageal adenocarcinoma (EAC) is a highly lethal malignancy, with a dismal 5-years survival rate of approximately 20%. Alarmingly, the incidence of EAC has increased by over 700% in the past 4 decades (1, 2, 3, 4). The current standard-of-care, which includes neoadjuvant chemoradiation (CRT) followed by surgery as per the CROSS-trial protocol, offers curative benefit to only a minority of patients (5, 6, 7). A study evaluating dose escalation from 50.4 Gy to 61.6 Gy for definitive CRT in patients with EAC showed no further improvement in clinical outcomes (8), underscoring the inherent radioresistance of these tumors. More recently, the ESOPEC trial demonstrated improved survival with a perioperative chemotherapy regimen (FLOT: 5-fluorouracil, leucovorin, oxaliplatin, and docetaxel) compared to CROSS-based CRT (9). However, FLOT is poorly tolerated by a significant proportion of patients, highlighting the need to better understand and overcome mechanisms of radioresistance in EAC.

Interferon-stimulated gene 15 (ISG15) was initially characterized for its role in antiviral immunity (10). However, emerging evidence shows that ISG15 has broader functions, including regulation of autophagy, exosome secretion, DNA translesion synthesis, and immune modulation (11, 12). Similar to ubiquitin, ISG15 can be covalently attached to lysine residues of target proteins through a process known as ISGylation, mediated by an enzymatic cascade involving E1 (UBE1L), E2 (UBCH8), and E3 (*e.g.*, TRIM family) enzymes (13). This post-translational modification can influence protein stability, function, and interaction with other proteins. Additionally, ISG15 can exist in a free, unconjugated form that is secreted by tumor cells into the tumor microenvironment (TME), where it acts as a cytokine to modulate immune responses (14, 15). Of particular relevance, ISG15 has been shown to mitigate replication stress (16). Loss of MRE11 stalls the replication fork, inducing ISG15 expression, which subsequently ISGylates an unidentified target to alleviate replication stress. Conversely, ISG15 knockdown leads to fork stalling and genomic instability. We recently reported that ISG15 expression is correlated with poor survival in treatment-naïve EAC patients, whereas ∼10% of patients with undetectable levels of tumor ISG15 showed better survival (15).

Given its role in replication stress, we hypothesized that ISG15 may influence DNA damage repair. Enhanced DNA repair capability is a key contributor to radioresistance in cancer cells (17). Ionizing radiation (IR) induces DNA double-strand breaks (DSBs), which are marked by phosphorylation of H2AX (γH2AX), initiating recruitment of repair proteins such as MDC1 (18) and downstream assembly of RAD51 foci (19). In our study, we observed that IR exposure in EAC cells upregulates ISG15 expression. Further investigation revealed that γH2AX undergoes ISGylation at lysine 120 (K120) following IR. This modification is critical for the retention of MDC1 and timely accumulation of RAD51 at DNA damage sites, facilitating homologous recombination (HR) repair. Collectively, our findings establish a novel role for ISG15 in promoting HR-mediated DNA repair and suggest that targeting ISG15 could be a promising strategy to radiosensitize EAC cells.

## Results

### Radiation induces ISG15 expression, and ISG15 loss enhances radiosensitivity in EAC cells

**Figure 1:**
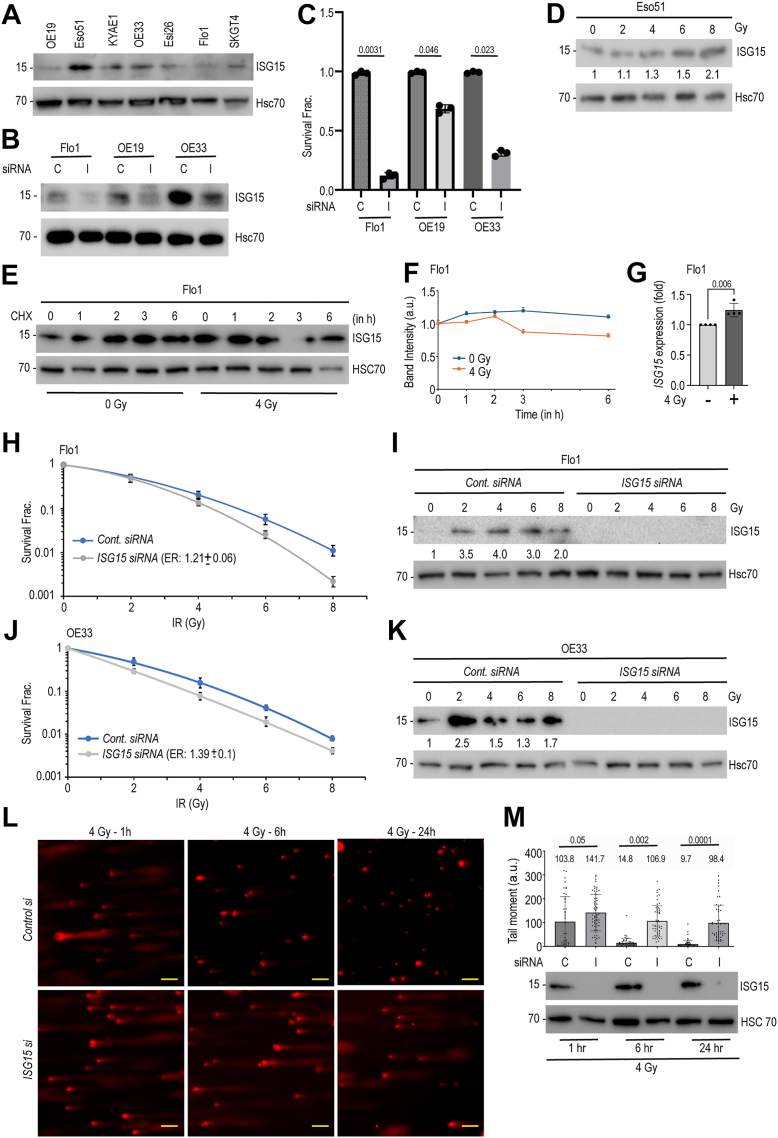
**Radiation induces ISG15 and its knockdown compromises DSB repair to radiosensitize EAC cells.***A*, cell lysates were prepared from different EAC cell lines (as indicated) and subjected to immunoblotting using indicated antibodies. *B*, we selected three different EAC cell lines (Flo1, OE19, and OE33), transfected with either control or *ISG15* siRNA as indicated, and 48 h post-transfection cell lysates were prepared and immunoblotted to show knockdown. *C*, same cell lines were subjected to clonogenic survival assays following control or *ISG15* knockdown. *D*, cell lysates from Eso51 cells irradiated with 4 Gy were harvested 24 h post-irradiation and immunoblotted for ISG15 and Hsc70 (loading control). *E*, Flo1 cells were either sham-irradiated (0 Gy) or exposed to 4 Gy and cultured for another 24 h before subjecting to cycloheximide (CHX) treatment for indicated time points for ISG15 protein half-life studies. *F*, for determination of half-lives, band intensities were quantified using Image J assuming the 0-h time point as ‘1’ (arbitrary unit, a.u.). The plot represents data from three independent studies. *G*, Flo1 cells were either sham irradiated or irradiated as above and 24 h post-irradiation, total RNAs were harvested and quantified for ISG15 transcript levels. GAPDH was used to normalize levels (*p* = 0.006). *H and J*, Flo1 (*panel H*) and OE33 (*panel J*) cells were transfected with either control or *ISG15* siRNAs and were treated with 0- to 8-Gy irradiation as indicated. Twenty-four hours after radiation exposure, cells were plated for clonogenic survival as described in Materials and Methods. The mean radiation enhancement ratios (ER) ± SEM (n = 3) were (Flo1: 1.21 ± 0.06; OE33: 1.39 ± 0.1) upon *ISG15* knockdown and found to be significantly different from control siRNA treated cells (*p* < 0.05). *I* and *K*, cell lysates from control and ISG15 knockdown cells treated with different doses of ionizing radiation were immunoblotted using specified antibodies. *L and M*, Flo1 cells transfected either with control or *ISG15* siRNAs were subjected to 4 Gy radiation and cells ability to repair damaged DNA were determined at different time points (1, 6, and 24 h) post-irradiation using alkaline comet assay. *Panel J*, showing representative images of irradiated control and ISG15 siRNA treated cells collected at 24 h. scale bar, 5 μm. *Panel K*, Quantification of olive tail moment (n = 50 cells) showing repair defects upon loss of ISG15. The statistical significance of differences between control and *ISG15* siRNA treated groups at different time points were evaluated using 2-way ANOVA and *p* values were included.

We began our investigation by assessing basal ISG15 expression across a panel of seven esophageal adenocarcinoma (EAC) cell lines: OE19, Eso51, KYAE1, OE33, Eso26, Flo1, and SK-GT-4. Consistent with our report indicating ISG15 expression in approximately 90% of EAC patient tumors (15), all seven cell lines exhibited detectable ISG15 expression (Fig. 1*A*), with Eso51 and OE33 displaying the highest levels.

To evaluate the functional significance of ISG15 in EAC cell survival, we silenced ISG15 using either a SMARTpool of siRNAs or a 3′ untranslated region (3′ UTR)-specific siRNA. Knockdown of ISG15 significantly reduced clonogenic survival across all tested cell lines (Figs. 1, *B*, *C* and S1). Among them, Flo1 cells exhibited the greatest sensitivity, with an 80 to 90% reduction in colony formation, while the remaining cell lines showed intermediate sensitivity to ISG15 depletion.

Given that ISG15 is induced by multiple genotoxic stressors, including ultraviolet (UV) radiation and camptothecin (a topoisomerase I inhibitor) (20, 21), we next investigated whether ionizing radiation (IR) similarly induces ISG15 in EAC cells. Cells were exposed to increasing doses of IR (0, 2, 4, 6, and 8 Gy), and ISG15 levels were assessed 24 h post-irradiation. As shown in Figure 1*D*, ISG15 expression was upregulated in a dose-dependent manner in multiple EAC cell lines, including Eso51. To determine whether IR-induced ISG15 upregulation occurs at the transcriptional or post-translational levels, Flo1 cells were either sham-radiated or exposed to 4 Gy. Based on cycloheximide (CHX)-based protein half-life studies (Fig. 1, *E* and *F*), we did not notice any significant difference upon ionizing radiation. However, based on qRT-PCR, we noticed increased *ISG15* transcript levels in irradiated Flo1 cells (Fig. 1*G*), which may be attributed to radiation induced activation of type I interferon signaling (22).

To evaluate whether *ISG15* depletion enhances radiosensitivity in EAC cells, we performed clonogenic survival assays following ionizing radiation (IR). Knockdown of *ISG15* significantly sensitized both Flo1 and OE33 cells to IR, with enhancement ratios (ER) of 1.21 ± 0.06 and 1.39 ± 0.10, respectively (Fig. 1, *H* and *J*). Notably, as in Eso51 (Fig. 1*D*), IR exposure induced ISG15 expression in both Flo1 and OE33 cells, as confirmed by immunoblotting (Fig. 1, *I* and *K*), suggesting a radiation-induced upregulation of ISG15 as a potential adaptive resistance mechanism.

To determine the impact of ISG15 on the efficiency of DNA damage repair, we performed alkaline comet assays, which assesses a combination of double-strand breaks (DSBs), single-strand breaks (SSBs) and apurinic sites (APs). Flo1 cells were transfected with control or *ISG15*-targeting siRNA, exposed to 4 Gy, and collected at 1-, 6-, and 24-h post-irradiation. Quantification of DNA damage using olive tail moment revealed that control siRNA-treated cells efficiently repaired within 6 h. In contrast, ISG15 knockdown cells retained significant DNA damage even at 24 h post-irradiation (Fig. 1, *L* and *M*), indicating a critical role for ISG15 in the repair of radiation-induced DNA damage.

To further evaluate the role of ISGylation, we examined whether loss of the ISGylation-specific E2 enzyme *UBE2L6*/UBCH8 influences the radiosensitization of EAC cells. In contrast to *ISG15* depletion, *UBE2L6* knockdown did not radiosensitize Flo1 cells (ER: 1.02 ± 0.12; Fig. S2*A*). UBCH8 is a unique E2 enzyme that functions in both ISGylation and ubiquitination. Notably, c-MYC is among the proteins targeted for degradation by the ELL–UBCH8 E2–E3 complex (23). As expected, UBCH8 loss resulted in c-MYC upregulation in EAC cells (Fig. S2*B*), which may contribute to protecting cells from radiation-induced cell death.

### ISGylation is important for efficient HR repair

**Figure 2:**
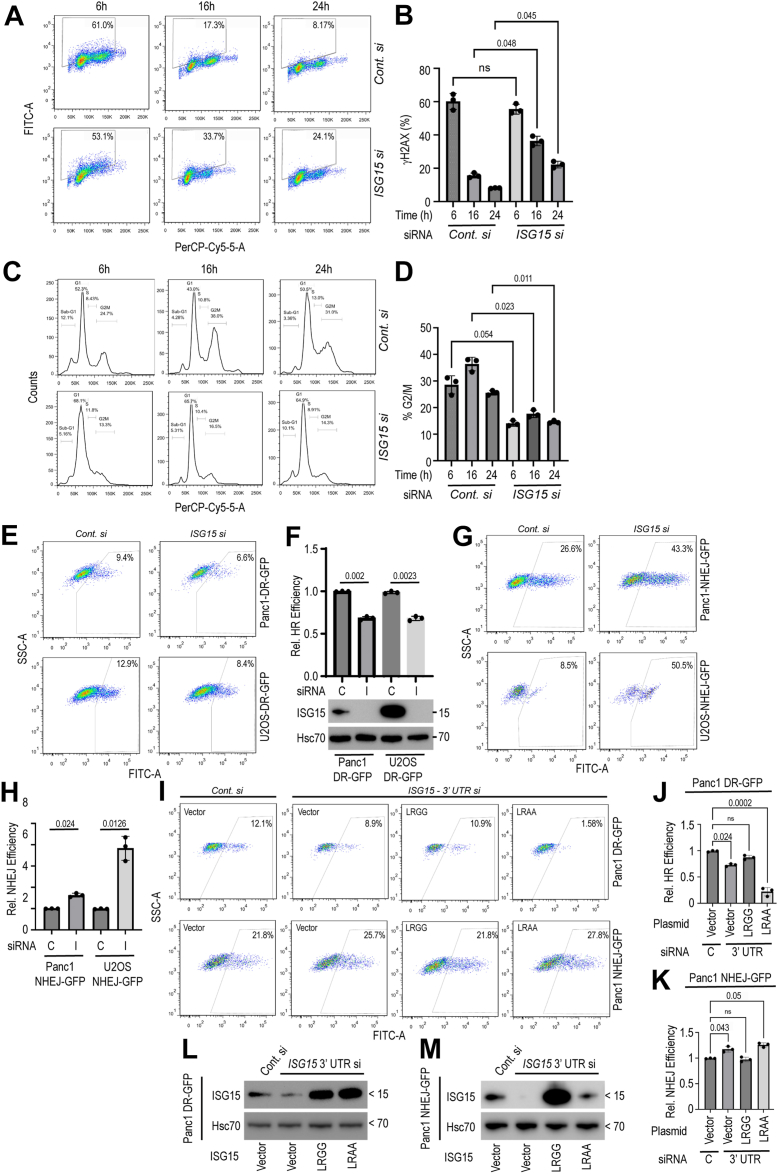
**ISG15 loss compromises HR repair with compensatory upregulation of NHEJ.***A & B*, Flo1 cells with or without ISG15 were exposed to 4 Gy IR and collected after 6, 16, and 24 h post-irradiation. Cells were then stained with γH2AX and quantified using FACS (in A) and quantified (in B). *C & D*, Flo1 cells were similarly subjected to siRNA transfection, irradiated and collected at different time points as above, were stained with PI and analyzed for cell cycle distribution based on DNA amounts (in C). The G2/M (4N DNA content) percentage of cells were plotted (in D). *E*, Panc1 and U2OS cells stably expressing DR-GFP reporter were treated with either control (C) or *ISG15* (I) siRNAs and 24 h post-transfection cells were infected with Adenovirus expressing I-SecI endonuclease to cause double strand DNA breaks as reported earlier (24, 56). HR mediated repair efficiency was quantified based on the percentage of GFP + cells. *F*, relative HR efficiency upon ISG15 loss were calculated considering control siRNA treated cells as ‘1’ showing significance (*p* = 0.002 and 0.0023) (*upper panels*). *ISG15* knockdown efficiency is shown based on immunoblotting data (*lower panel*). *G & H*, similar experiments as above were conducted in Panc1 and U2OS NHEJ-GFP reporter cell lines following control and ISG15 siRNA treatments showing compensatory upregulation (*p* values are included with corresponding groups). *I*, Panc1 DR-GFP and NHEJ-GFP reporter cell lines were either transfected with control siRNA or a siRNA targeting the 3′ UTR of *ISG15* gene for knocking down endogenous ISG15 protein. Cells were then infected with the I-SecI adenovirus and 24 h post-infection, cells were further transfected with either empty vector or cDNAs overexpressing either wild-type (LRGG) or ISGylation deficient (LRAA) ISG15 mutants as indicated. Twenty-four hours of cDNA overexpression, cells were analyzed using FACS to quantify HR and NHEJ mediated DNA repair abilities. *J & K*, plots showing fraction of cells able to repair I-SecI mediated DNA damage by considering adenovirus infected control siRNA treated cells as ‘1’ (*p* values are included for corresponding groups). Data presented as mean ± SD from three independent experiments. Two-tailed student’s *t* tests were used to calculate *p*-values. *L & M*, immunoblotting of cell lysates showing knockdown and overexpression of LRGG and LRAA ISG15 mutants.

To understand the role of ISG15 in promoting radioresistance in EAC, we focused on understanding its role in the DNA damage response (DDR). We began by quantifying γH2AX using FACS in Flo1 cells collected at different time points (6, 16, and 24 h) following 4 Gy. As expected, γH2AX levels were the highest (61%) at 6 h post-irradiation in control siRNA-treated cells, which were substantially reduced (17 and 8%) by 16 h and 24 h post-irradiation, respectively, reflecting efficient DNA repair. In contrast, *ISG15* knockdown Flo1 cells showed persistently higher (24%) γH2AX levels even at 24 h post-IR (Fig. 2, *A* and *B*). We also found that Flo1 cells lacking ISG15 failed to elicit an IR induced G2/M checkpoint arrest (Fig. 2, *C* and *D*), which may cause radiosensitization.

To understand the role of ISG15 with the type of DDR, we focused on homologous recombination (HR) and the non-homologous end joining (NHEJ). We used GFP-based reporters stably expressed in either Panc1 (24) or U2OS cells. As shown in Figure 2, *E* and *F*, we observed ∼30% reduction in HR reporter activity in both Panc1 and U2OS systems following ISG15 knockdown. In contrast, in NHEJ-GFP Panc1 and U2OS reporter cells, loss of ISG15 caused a compensatory upregulation of NHEJ repair (Fig. 2, *G* and *H*). To distinguish whether the free ISG15 or its conjugation to target proteins (ISGylation) is impacting the HR and the NHEJ repair pathways, we first used a 3′UTR specific *ISG15* siRNA to knockdown endogenous protein, followed by overexpression of either ISGylation-proficient wild-type (LRGG) or -deficient (LRAA) mutant proteins. We found that *ISG15* 3′UTR specific siRNA confirmed the loss in HR repair efficiency (Fig. 2, *I*–*K*). Furthermore, the overexpression of ISG15-LRGG partially rescued the HR defect phenotype, but the LRAA mutant behaved as a dominant negative by further inhibiting HR repair ability in the Panc 1 reporter system. In contrast, the effects of ISG15-LRGG and LRAA were opposite while using the NHEJ-GFP reporter (Fig. 2, *L* and *M*). Similar results were obtained using U2OS HR-GFP and NHEJ-GFP reporter systems (Fig. S3).

To evaluate whether compensatory upregulation of NHEJ affects HR repair in ISG15-depleted EAC cells, we performed radiosensitization assays in the presence and absence of the DNA-PKcs inhibitors VX-984 and M3814/peposertib. Consistent with previous reports (25), DNA-PKcs inhibition markedly radiosensitized Flo1 cells (VX-984: ER = 4.4 ± 0.12; peposertib: ER = 1.56 ± 0.2). In contrast, because ISG15 depletion enhances NHEJ activity, DNA-PKcs inhibition in *ISG15*-knockdown cells resulted in reduced radiosensitization (VX-984: ER = 3.65 ± 0.15; peposertib: ER = 1.28 ± 0.16) (Fig. S4, *A*–*C*).

### H2AX is a novel substrate of ISGylation, undergoing modification at the lysine (K) 120 position

**Figure 3:**
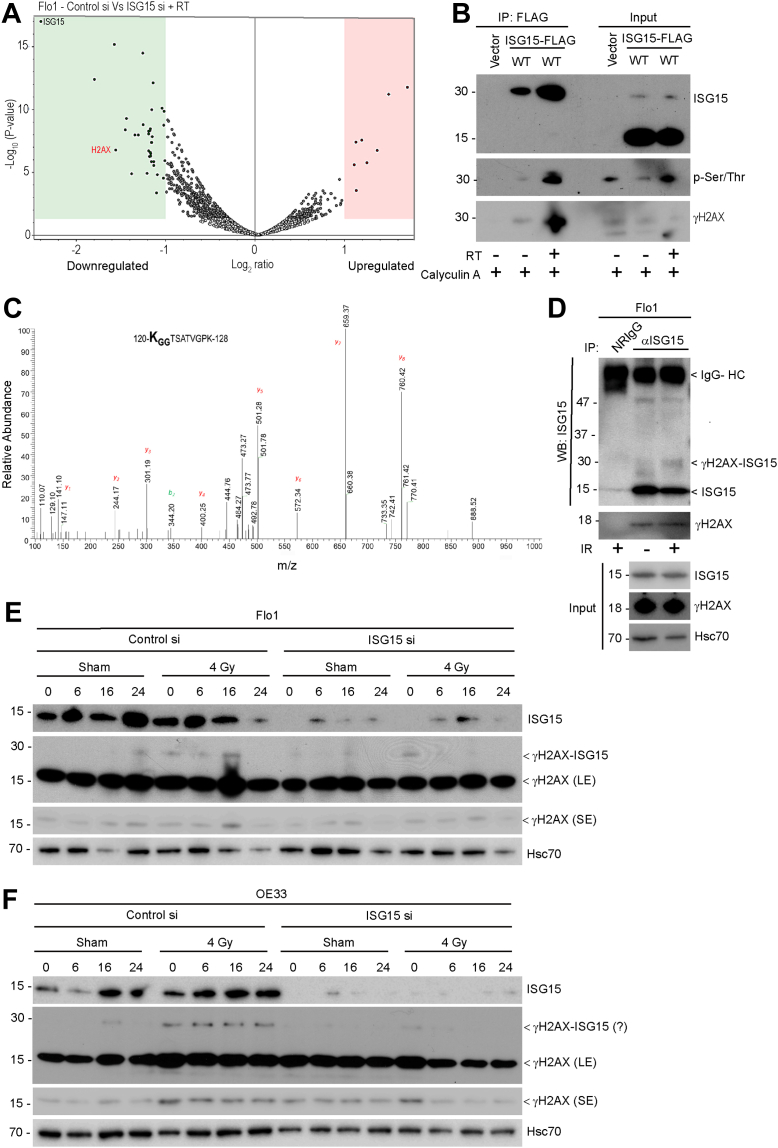
**H2AX is a novel substrate of ISGylation undergoing PTM at the lysine 120 site.***A*, Flo1 cells were transfected with either control or ISG15 siRNAs and 24 h post-transfection cells were either sham radiated or radiated with 4 Gy. Six hours post-irradiation, cell lysates were prepared and 75 μg of lysates from each treatment groups (done in triplicate) were labelled with distinct isotopes of TMT kit according to manufacturer’s protocol and were subjected to mass spectrometry analysis. Data presented as a volcano plot of differentially expressed (up and down-regulated proteins) from control and *ISG15* siRNA treated and irradiated cells which were highly significant (*p* < 0.001). Besides ISG15, the study identified H2AX as one of the down-regulated proteins. *B*, Flo1 cells overexpressing either empty vector or the DDK-tagged ISG15 were irradiated with 4 Gy and incubated for 6 h. One hour before the harvest, cells were treated with calyculin A (0.1 μM) and cell lysates were subjected to immunoprecipitation using FLAG (M2) beads. Immunoblotting was performed using indicated antibodies. *C*, immunoprecipitated proteins (similar as above) were run in a 4 to 12% SDS-PAGE gradient gel, and a piece of gel was cut about the size of 30 kDa and subjected to in-gel digestion followed by mass spectrometry (MS). Tandem MS spectrum showing the ubiquitination of H2AX (Acc. P16104, 120-KggTSATVGPK-128) on K120 site. Observed b- and y-ions are indicated. The presence of b2 with added 114.04 amu supports the modification of K120 with diglycine residue (remnant of ISG15 upon trypsin digestion). *D*, Flo1 cells were either sham radiated or with 4 Gy. Twenty-four post-irradiation, cell lysates were subjected to immunoprecipitation using ISG15 antibody and immunoblotted using indicated antibodies. *E and F*, Flo1 and OE33 cells respectively were either transfected with the control or *ISG15* siRNAs and 48 h post-transfection cells were either sham irradiated or exposed to 4 Gy and collected at indicated times (1, 6, 16, and 24 h) post-irradiation. Cell lysates were then subjected to immunoblotting using indicated antibodies. For clarity and to show the presence of ISGylated form of γH2AX, we have included a longer exposure (LE) and a shorter exposure (SE) of the blots as indicated.

To identify substrate(s) of ISGylation promoting efficient HR repair, we performed tandem mass tags (TMT), a multiplex platform that can concurrently identify and quantify relative protein levels in samples undergoing different treatments. As expected, ISG15 was the most downregulated protein in siRNA knockdown cells, validating the assay conditions. When we compared different treatment groups of irradiated cells with or without ISG15 expression, we identified multiple up and downregulated proteins (Table S1). Based on pathway enrichment analysis, the alterations were primarily noted in the structural constituents of chromatin, nucleosome DNA binding, and extracellular exosomes (Fig. S5). Among the downregulated proteins, we identified multiple histone H2 isoforms including H2AX, an early recognizer of DNA damage sites (Fig. 3*A*). Independently, we performed immunoprecipitation using DDK-tagged ISG15 followed by mass spectrometry to identify proteins undergoing radiation induced ISGylation, which identified a 30 kDa protein as one of the strong interactors (Fig. 3*B*). Based on mass spectrometry, we found that H2AX underwent ISGylation at the lysine 120 (K120) residue (Fig. 3*C*). For confirmation, we conducted immunoprecipitation followed by immunoblotting to identify phosphorylated H2AX (γH2AX) running at ∼30 kDa size (instead of ∼18 kDa) in irradiated samples. The same size band was also detected when probed with the ISG15 antibody. We performed immunoprecipitation followed by immunoblotting using an ISG15 antibody to assess the interaction between endogenous ISG15 and γH2AX. This approach also revealed a higher-molecular-weight species (∼30 kDa) in irradiated Flo1 cells, consistent with a potential ISGylated form of γH2AX (Fig. 3*D*). This higher molecular weight γH2AX was detected in irradiated Flo1 and OE33 cells, which was abolished upon *ISG15* knockdown (Fig. 3, *E* and *F*). Together, we identified H2AX as a novel substrate of ISGylation following IR and that K120 is the site of post-translational modification.

### H2AX ISGylation is important for MDC1 retention at the DNA damage sites

**Figure 4:**
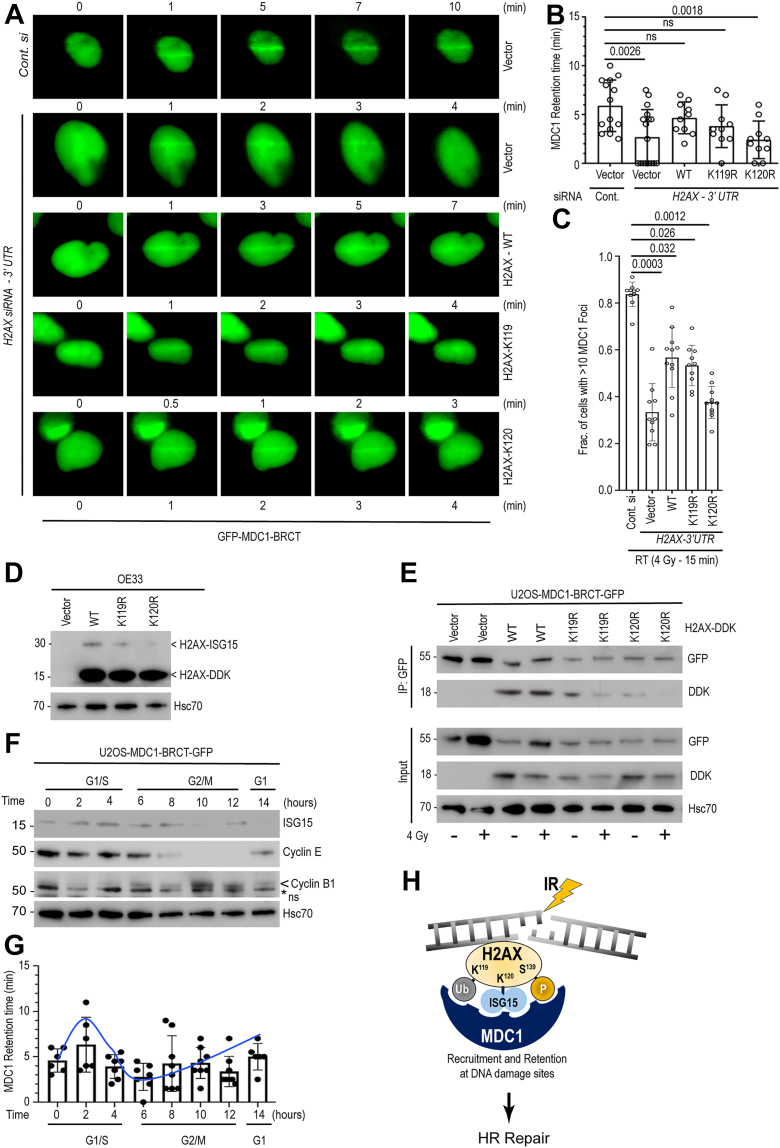
**H2AX ISGylation is important for MDC1 retention at the DNA damage sites.***A*, U2OS cells stably expressing MDC1-BRCT-GFP were transfected with either control or H2AX siRNAs (directed to the 3′ UTR to knockdown endogenous H2AX). Twenty-four hours post siRNA transfection, cells were transfected with plasmids expressing DDK-tagged H2AX cDNA encoding either wild-type (WT), K119R, and K120R mutants as indicated. GFP-BRCT-MDC1 localization along mIR was monitored and imaged at indicated times to show retention at DNA damaged sites. *B*, average GFP-BRCT-MDC1 retention time at the DNA damage sites are shown in minutes. *C*, OE33 cells with H2AX knockdown using 3′ UTR specific siRNAs were transfected with either vector or different H2AX overexpressing plasmids as indicated to perform rescue studies. Twenty-four hours post-transfection, cells were radiated (4 Gy) and fixed after 15 min followed by immunofluorescence staining with MDC1 antibody to quantify amount of recruitment. Cells with MDC1 foci were counted for each sample and fraction of cells with >10 foci were plotted after normalizing with control siRNA treated and irradiated sample as ‘1’. *D*, immunoblotting of OE33 cell lysates were immunoblotted showing overexpression of H2AX-DDK (WT and KR mutants). Blots also show detection of possible ISGylated H2AX. *E*, U2OS cells stably expressing GFP-MDC1-BRCT were transfected with H2AX-DDK (WT, K119R, and K120R). Twenty-four hours post-transfection, cells were irradiated (4 Gy) and 6 h post-IR, cell lysates were subjected to immunoprecipitation using anti-GFP antibody and immunoblotted using indicated antibodies. *F & G*, U2OS cells stably expressing GFP-BRCT-MDC1 were synchronized using double thymidine block and release. Cells were analyzed every 2 h for the accumulation of MDC1 along the DNA damage tracks after mIR and quantified for the retention time (*upper panel*). Cell lysates were also prepared from the above analyzed samples and immunoblotted to show cell cycle distribution using different cyclins (*upper panels*). For Cyclin B1 immunoblotting, we detect a non-specific band (ns). *H*, schematic diagram showing coordinated post-translational modifications at the two (119 and 120) lysine and a serine (139) residues involved in recruiting and retaining MDC1 at the site of DNA damage essential for effective RAD51 recruitment and HR mediated DNA damage repair.

In HR repair, γH2AX is involved in recognizing DNA damage sites that help in recruiting DDR proteins, particularly the mediator of DNA damage checkpoint 1 (MDC1), by interacting *via* its BRCA1 C-terminal (BRCT) domain (26). To understand the role of γH2AX ISGylation in MDC1 recruitment and retention at the DDR sites, we used a U2OS reporter cell line stably expressing a GFP-tagged MDC1-BRCT domain (Fig. S6, *A* and *B*). In this line, we first knocked out endogenous H2AX using a 3′UTR specific siRNA and performed rescue experiments by transiently overexpressing H2AX cDNA encoding wild-type (WT), a K119R mutant (previously reported to compromise mono-ubiquitination, hence DDR functionality) (27), and a K120R mutant. We recorded MDC1 recruitment and retention times in live U2OS cells after DNA damage produced using microscope-controlled UVA laser micro-irradiation (mIR), as described previously (24) (Fig. 4*A*). Irrespective of H2AX mutation types, MDC1-BRCT-GFP recruitment was unaffected following mIR, suggesting H2AX ISGylation is not critical for MDC1 recruitment. In contrast, the retention time for the GFP-MDC1-BRCT, which was for 5.89 ± 0.37 min after control siRNA, was substantially reduced to 2.67 ± 0.33 min upon *H2AX* knockdown using a 3′ UTR specific siRNA, suggesting that H2AX is important in MDC1 *retention* but *not recruitment* at the DNA damage sites (Fig. 4*B*). We then performed rescue experiments by overexpressing either wild-type, K119R, or K120R mutants in 3′UTR *H2AX* siRNA transfected cells. As shown in Figure 4*B*, wild-type H2AX increased MDC1-BRCT-GFP retention time to 4.7 ± 0.32 min which was similar to the control siRNA treated group. In contrast, expression of the K120R single mutant was least effective restoring MDC1-BRCT-GFP retention time (2.4 ± 0.38 min) compared to WT H2AX. Additionally, using U2OS cells, co-overexpression of GFP-tagged ISGylation-proficient (LRGG) and mCherry-tagged ISGylation-deficient (LRAA) mutants of ISG15 when subjected to mIR studies, the GFP-LRGG showed accumulation at the sites of DNA damage, but no signal was detected for mCherry-LRAA mutant (Fig. S7), further confirming the importance of ISGylation and not the free ISG15 in DNA damage response. We have observed similar trend in MDC1 retention at DNA damage sites when OE33 cells were used to knockdown endogenous H2AX followed by overexpression of either wild-type or different mutants and the cells were then exposed to 4 Gy and processed after 15 min post-IR (Fig. 4*C*). In Figure 4*D*, we confirmed IR induced ISGylated H2AX-DDK expression, which was absent in K120R mutants. To test the importance of K119 and K120 sites in the interaction with MDC1, we performed immunoprecipitation studies in U2OS cells stably expressing GFP-MDC1-BRCT overexpressing H2AX-DDK. As expected, compared to wild-type, the K119 R and K120R single mutants showed substantial lower interaction with MDC1 (Fig. 4*E*).

While analyzing data in Fig. 4*B*, we noticed two distinct sub-populations of cells in each treatment group; one cluster of cells showed longer MDC1-BRCT-GFP retention at the DDR sites, while the other showed shorter retention time. We hypothesized that this may be due to cell cycle distribution. To study, we repeated mIR studies as above after synchronizing cells using double thymidine block and release method as described earlier (28). Following a second thymidine block (primarily at the G1-S boundary), cells were released in regular culture medium and assessed for MDC1-BRCT-GFP retention time after 0, 2, 4, 6, 8,10, 12, and 14-h. We also isolated whole cell lysates, which were subjected to immunoblotting to confirm cell cycle stages using cyclin antibodies and probed for ISG15 (Fig. 4*F*). For synchronized cells, we noted longer retention of MDC1-BRCT-GFP in S-phase cells (6.3 ± 1.0 min), in contrast to the shortest retention time in G2-M phase cells (2.8 ± 0.43 min) (Fig. 4*G*). Importantly, ISG15 levels were also found to be cell cycle regulated, with the highest levels found in late S and early G2 phases. This correlates well with ISG15’s role in HR repair, which is the predominant repair mechanism in S-G2 phase of cell cycle (29) and when cells are more radioresistant (30). Together, these data support a model in which multiple PTMs (K119 di-ubiquitination, K120 ISGylation, and Ser139 phosphorylation) act in concert to regulate MDC1 recruitment and retention at DNA damage sites, thereby facilitating efficient HR repair (Fig. 4*H*).

To delineate the relative contributions of H2AX K120 ISGylation and Ser139 phosphorylation to the DNA damage response, we performed radiosensitization studies in the presence or absence of the ATM inhibitor AZD1390, which blocks Ser139 phosphorylation (31). Consistent with observations in other cancer cell lines, AZD1390 treatment markedly radiosensitized Flo1 cells (ER: 2.84 ± 0.06). Loss of *ISG15* also sensitized Flo1 cells to radiation (ER: 1.44 ± 0.07), although to a lesser extent compared to ATM inhibition. Combined *ISG15* depletion and AZD1390 treatment resulted in additional increase in radiosensitization (ER: 3.11 ± 0.06) compared with AZD1390 alone, and this difference was significant (*p* = 0.013) (Fig. S8, *A* and *B*) suggesting the importance of both ISGylation and phosphorylation in H2AX mediated DNA damage response.

**Figure 5:**
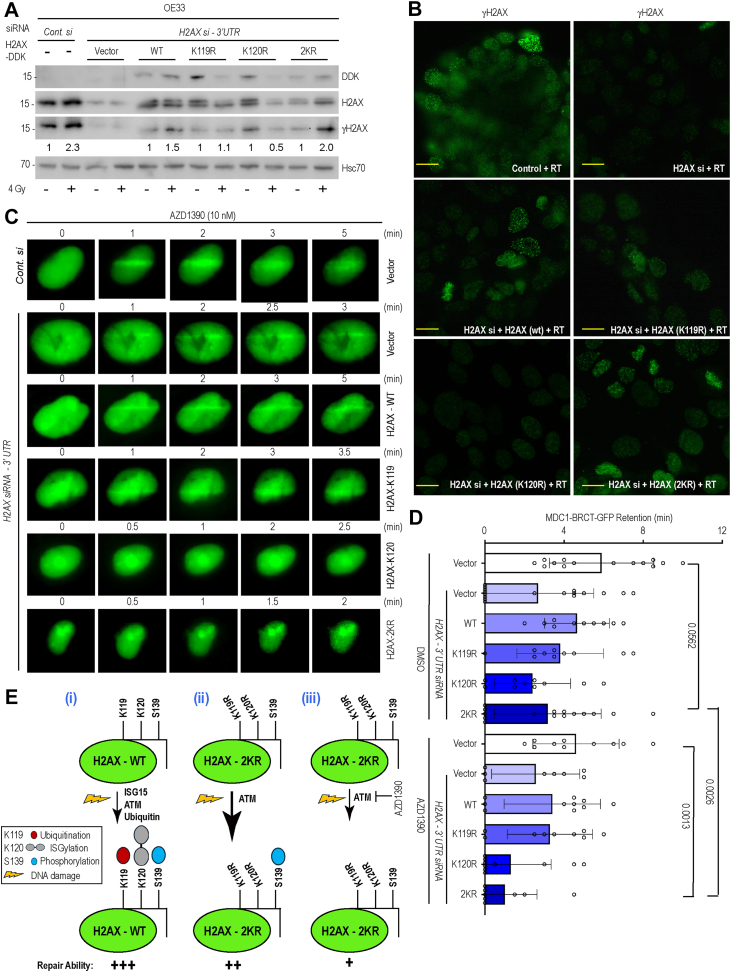
**Lysine (K) 119, 120 and serine 139 site modifications cooperate in DNA damage repair.***A*, cell lysates were prepared from OE33 cells transfected with control or ISG15 siRNA and subsequently overexpressing DDK-tagged H2AX (WT or indicated mutants). Cells were either sham-irradiated or exposed to 4 Gy, and lysates were collected 6 h post-irradiation. Immunoblotting was performed using the indicated antibodies. *B*, representative images of the above experiment showing γH2AX foci in different treatment groups as indicated. Scale bars, 10 μm. *C*, U2OS cells stably expressing GFP-MDC1-BRCT were subjected to siRNAs followed by plasmid transfection as shown. Cells were then treated with an ATM inhibitor (AZD1390) for 1 h and then exposed to mIR and imaged. *D*, GFP-MDC1-BRCT retention time at the site of DNA damage were calculated for each group as shown (*p* values are included for comparison groups). Data presented as mean ± SEM calculated from >15 mIR cells. *E*, proposed model: (i) Cooperative post-translational modifications at K119 (di-ubiquitination), K120 (ISGylation), and Ser139 (phosphorylation) are required for efficient DNA damage repair (repair capacity: +++). (ii) Although the H2AX-2KR mutant cannot be modified at K119 or K120, it exhibits enhanced DNA repair capacity (repair capacity: ++) compared with the single K119R or K120R mutants. We propose that this is due to more efficient ATM-mediated Ser139 phosphorylation in the absence of competing lysine modifications. (iii) Consistent with this model, pharmacologic inhibition of ATM using AZD1390 abolishes the enhanced DNA repair capacity of the H2AX-2KR mutant (repair capacity: +).

While analyzing immunoblotting data from OE33 cells lacking endogenous H2AX and expressing either wild-type or mutant H2AX constructs, we observed increased Ser139 phosphorylation following 4 Gy in the H2AX-K119/120R (2KR) mutant compared with the individual single mutants (Fig. 5*A*). These results suggest that, in the absence of both lysine modifications (ubiquitination and ISGylation), ATM phosphorylates Ser139 more efficiently, potentially compensating for the loss of K119 and K120 PTMs and thereby enhancing γH2AX-2KR functionality. Consistent with these findings, immunofluorescence analysis of OE33 cells expressing the different γH2AX mutants revealed a similar trend (Fig. 5*B*). To assess the functional consequences of these modifications, we measured MDC1-GFP retention at micro-irradiated sites in U2OS cells lacking endogenous H2AX and overexpressing the indicated H2AX mutants, in the presence or absence of AZD1390. As expected, the K120R mutant exhibited reduced MDC1 retention (2.4 ± 0.32 min), whereas the 2KR mutant showed a longer retention time (3.16 ± 0.21 min) in DMSO-treated cells. In contrast, upon AZD1390 treatment, both the K120R and the 2KR mutants displayed markedly reduced MDC1-GFP retention (1.3 ± 0.23 min and 1.00 ± 0.19 min, respectively) (Fig. 5, *C* and *D*).

Together, these data support a model in which the absence of K119 di-ubiquitination and K120 ISGylation permits more efficient Ser139 phosphorylation. As a result, the H2AX-2KR mutant exhibits enhanced MDC1 recruitment and retention at DNA damage sites compared with either single mutant (Fig. 5*E*). Consistently, treatment of cells expressing the H2AX-2KR mutant with AZD1390 abolishes its enhanced DNA repair capacity, underscoring the critical and coordinated roles of all three PTMs in regulating the DNA damage response.

### High RAD51 expression correlates with poor prognosis in patients with node-positive EAC

RAD51 is a key downstream effector of the homologous recombination (HR) repair pathway (32). Given that ISG15 knockdown impairs HR and reduces the retention of MDC1 at DNA damage sites, we next assessed the impact of ISG15 depletion on RAD51 foci formation following IR. As shown in Fig. S9, *A* and *B*, ISG15 knockdown significantly reduced RAD51 foci formation at early time points (1–6 h post-irradiation), indicating impaired recruitment. Interestingly, a delayed accumulation of RAD51 foci was observed at the later time points (16–24 h), suggesting that ISGylation is important for the efficient and timely recruitment or retention of RAD51 at sites of DNA damage, facilitating effective HR repair. These findings underscore the functional importance of ISGylation in orchestrating the DNA damage response (DDR) following ionizing radiation.

**Figure 6:**
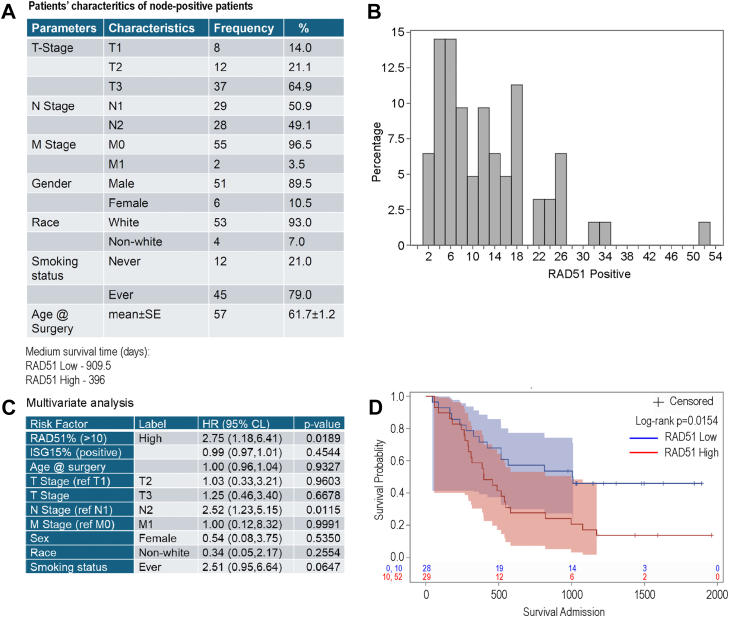
**High RAD51 is correlated with increased hazard ratio in patients with node-positive EAC.** We have previously established a tissue microarray (TMA) from treatment resistant EAC patients, which was subjected to IHC staining using RAD51 and ISG15 antibodies as described. Stained slide was scanned and quantified to determine percentage RAD51 and ISG15 positive cells and were used for clinical correlation analysis. *A*, table showing patients’ distribution based on T/N/M stages, gender, race, and smoking history. *B*, Bar showing distribution of RAD51 expression (in percent) among the node-positive EAC patients (n = 57). *C*, results showing hazard ratio (HR) and corresponding *p* values of multivariate analysis based on criteria as listed. *D*, Kaplan–Meier survival curve with 95% Hall-Wellner confidence bands among patients with node-positive (n = 57) EAC grouped by RAD51 expression levels. High expression (*red*) levels were defined as values above the cohort’s median expression (10–52) level, while low (*blue*) was defined as below the median expression (0–10).

We then evaluated ISG15 and RAD51 expression levels in chemoresistant esophageal adenocarcinoma (EAC) patient samples. We generated a tissue microarray (TMA) composed of tumor tissues from 113 chemoresistant patients with EAC, 57 of whom (∼44%) were node-positive, a subgroup associated with worse clinical outcomes (Fig. 6*A*). Each patient was represented by three independent tumor cores, annotated by a trained pathologist. Immunohistochemical (IHC) analysis was performed to quantify ISG15 and RAD51 expression, and mean positivity scores were correlated with clinical outcomes. ISG15 expression was detected in almost all cases, highlighting its prevalent upregulation in chemoresistant EAC tumors. This is similar to our previous findings in treatment-naïve EACs, where only 12% of patients lacked ISG15 expression (and exhibited better survival outcomes (15)). ISG15 staining intensity did not correlate with survival (Fig. S10, *A*–*C*), though the near-universal positivity suggests ISG15 upregulation may be associated with poor prognosis in EAC. We additionally evaluated the association between RAD51 and ISG15 across all TMA cores and found no significant correlation (Spearman *ρ* = −0.048, *p* = 0.36).

Although RAD51 expression did not correlate with overall survival across the entire cohort, multivariate analysis adjusted for age and TNM staging revealed that in the node-positive subgroup (n = 57), high RAD51 expression was significantly associated with worse survival outcomes (hazard ratio [HR] = 2.35; *p* = 0.0154) (Fig. 6, *B*–*D*). This finding aligns with prior studies implicating elevated RAD51 expression in aggressive disease phenotypes across multiple cancer types (33, 34, 35). Collectively, these data support a model wherein ISGylation promotes HR-mediated repair through RAD51 recruitment, and high RAD51 expression may serve as a prognostic marker for poor outcome in node-positive EAC patients.

## Discussion

In this study, we identified a novel post-translational modification of H2AX (ISGylation at lysine 120 (K120)) that plays a critical role in facilitating efficient HR repair following IR. We demonstrate that this modification is essential for the retention of MDC1 at DNA double-strand break (DSB) sites and the timely recruitment of RAD51, thereby enabling effective HR-mediated DNA repair. These findings support our observations linking high ISG15 expression to poor overall survival in EAC patients (15). Mechanistically, we have shown that ISG15 is directly involved in the DDR by modifying H2AX that cooperates with already reported serine 139 (S139) phosphorylation by ATM and lysine 119 (K119) di-ubiquitination by different ubiquitin ligases. Together, these modifications orchestrate MDC1 recruitment and retention, followed by RAD51 accumulation, to enable effective HR repair (Fig. 4*H*). Perturbing this axis either *via ISG15* knockdown, overexpression of an ISGylation-deficient mutant (LRAA), or overexpression of an H2AX-K120R mutant impairs HR repair and sensitizes EAC cells to radiation. These findings highlight ISG15 as a key mediator of IR-induced DDR and a potential target for radiosensitizing EAC cells.

ISG15 has previously been implicated in alleviating replication stress by modifying DNA polymerases and PCNA, which can facilitate bypassing replication blocks, contributing to therapy resistance (36, 37). However, its role in IR-induced DDR was unknown. Here, we have established ISG15 as a key modulator for γH2AX function and HR repair. HR is a slow but accurate repair mechanism during the S/G2 phases, whereas non-homologous end joining (NHEJ) is a faster but error-prone alternative (38, 39). Using multiple model systems (Panc1, U2OS, and EAC lines), we confirm that ISG15 is essential for HR. Importantly, this role is dependent on ISGylation activity; the LRAA mutant, which cannot conjugate to targets, failed to support HR in mIR assays. Furthermore, ISG15 deficiency induced compensatory upregulation of NHEJ, a phenomenon observed in other models of HR deficiency (40). As NHEJ is error-prone, targeting ISG15 might further destabilize the genome, enhancing therapeutic efficacy against resistant tumor cells (41).

Given that HR repair is tightly linked to the cell cycle, with maximal activity during S/G2 phases (30), we examined ISG15 expression in synchronized cells. ISG15 levels were elevated during S/G2, correlating with prolonged MDC1 retention at mIR-induced DSBs. This pattern is reminiscent of other HR regulators, such as Bloom syndrome helicase (BLM), whose S-phase expression is associated with poor prognosis and therapy resistance in prostate cancer, glioma, and multiple myeloma (42, 43). Similarly, elevated MDC1 and RAD51 levels have been associated with adverse outcomes across cancers (33, 44). Our clinical analysis further revealed that high RAD51 expression in node-positive EAC patients correlates with poor survival, consistent with its known role in therapy resistance. Our data suggest that these chemoresistant EAC patients may benefit from RAD51 inhibitors, which are currently under clinical investigation (45, 46, 47). Additionally, since both chemotherapy and radiation impact DDR pathways, targeting ISGylation may be a common vulnerability that can be explored as a novel strategy for patients with EAC irrespective of either the CROSS or FLOT regimens.

We acknowledge certain limitations in our study. Due to similar molecular weights, we could not definitively distinguish between K120 ISGylation and di-ubiquitination, both of which can increase H2AX mass by ∼15 kDa. These modifications may be mutually exclusive or context-dependent and vary by cell type or extent of DNA damage. Additionally, our TMT mass spectrometry data showed ISG15 loss results in downregulation of multiple H2A isoforms, including H2AX, warranting further investigation into ISG15’s broader impact on chromatin structure. Understanding how ISG15 deficiency activates NHEJ and how mutant p53 might influence repair pathway choice could offer further insight into EAC therapy resistance. Our *in vitro* studies (Fig. S4) show that ISG15 targeting may dampen DNA-PKcs inhibitor’s radiosensitizing potential, however this requires further preclinical testing.

In summary, our study identifies H2AX as a novel ISGylation substrate and reveals ISG15 as a critical regulator of HR-mediated DNA repair following IR. These findings provide mechanistic insight into how ISG15 and its conjugation pathway contribute to therapy resistance in EAC and offer a compelling rationale for developing ISGylation-targeting strategies to overcome resistance in this increasingly prevalent disease.

## Experimental procedures

### Cell lines and reagents

Human embryonic kidney (HEK-293), cervical carcinoma (HeLa), osteosarcoma (U2OS), and EAC cell lines (OE33, OE19, and Flo1) were purchased from the American Type Culture Collection (ATCC). The other EAC cell lines (Eso51, Eso26, SKGT4, and KYAE1) were purchased from SigmaMillipore (Burlington, MA). Cells were either grown in RPMI1640 or DMEM supplemented with 10% fetal bovine serum and antibiotics, as we previously reported (48). Cells were routinely tested for any unwanted pathogen infection and genotyped for authenticity at the University of Michigan Advanced Genomics Core (AGC). For most of our experiments, cells were plated a day before the treatment. Plasmid transfections were performed using Lipofectamine 2000 (Invitrogen) or FuGENE (Promega) according to the manufacturer’s protocol. We used Lipofectamine RNAiMAX (Invitrogen) for siRNA mediated knockdown studies as previously described (49).

Antibodies included anti-DDK (OTI4C5; Origene Technologies), anti-GFP (JL-8; Takara Bio, Fitchburg, WI) and Hsc70 (sc-7298) were from Santa Cruz Biotechnology. The RAD51 antibody was purchased from SigmaMillipore (Cat# PC130) and ISG15 (Cat# MA5-29371) and c-Myc (MA1-980) antibodies were from Invitrogen. Rabbit polyclonal UBE2L6/UBCH8 antibody (Cat# NBP3-00032) was purchased from Novus Biologicals. FuGENE HD (Promega Cat# E2311) and Lipofectamine 2000 (Cat# 11668019) were from ThermoFisher Scientific. We used *ISG15* SMARTpool (Cat# L-002435-03), *ISG15* 3′ UTR specific siRNA (Cat# J-002435-005), *H2AX* 3′ UTR specific siRNA (Cat# L-011682-00-0005), and *UBE2L6* SMARTpool (Cat# L-008569-00-005) from Dharmacon.

### Protein isolation, analysis and immunoblotting

Proteins were isolated according to the protocol as described (49). Briefly, the lysis buffer was prepared by adding 50 mM HEPES/KOH, 150 mM NaCl, 1 mM EDTA, 2.5 mM EGTA, 1 mM N-ethylmaleimide, 1 mM NaF, 100 μM sodium orthovanadate, 10% glycerol, 10 mM β-glycerophosphate, 0.1% NP-40, and protease inhibitor cocktail. To prevent degradation of proteins, the entire procedure of extraction was carried out on ice and the protein content was estimated using Bradford reagent. Gel electrophoresis was performed in 4 to 12% precast bis-tris gel (Invitrogen) and blotted on PVDF membrane and subjected to immunoblotting.

### RNA isolation and quantitative RT-PCR

RNA isolation and qRT-PCR were performed as recently published. Briefly, total RNA was isolated using the RNeasy mini kit (Qiagen, SCR_008539). RNA samples (1 μg) were reverse-transcribed using the High-Capacity cDNA Reverse Transcription System (Applied Biosystems, Cat. # 436884, SCR_005039) according to the manufacturer’s protocol. For the ISG15 and GAPDH transcript quantification, we used real-time PCR using ABI PRISM 7700 using Power SYBR Green PCR Master Mix (Applied Biosystems). The following validated human *ISG15* and *GAPDH* primer sets were used for the PCR: ISG15 (5′-GAGAGGCAG CGAACTCATCT-3′; 5′-CTTCAGCTCTGACACCGACA-3′) and GAPDH (5′ GAGTCAACGGATTTGGTCGT-3′; 5′-TTGATTTTGGAGGGATCTCG-3′) primer pairs, as reported previously (50, 51).

### Immunofluorescence studies

Immunofluorescence was performed according to the protocol mentioned in (49). Briefly, cells were fixed either using 10% buffered formalin or ice-cold methanol. Cells were permeabilized using 0.2% Triton X-100, followed by blocking for an hour and incubation with primary antibody overnight at 4 °C, in a humidified chamber. Next day, cells were stained with fluorescently labelled secondary antibody, followed by washing with PBS-Tween20 and mounted with ProLong Gold anti-fade reagent with DAPI. We used either Nikon or Olympus fluorescent microscopes to capture the images.

### Laser mIR and live cell imaging

Stably transfected MDC1-GFP (either the full length or BRCT-domain containing) U2OS cells were plated on a glass bottomed culture dishes, which were then subjected to laser mIR using an Olympus IX71 microscope attached with MicroPoint Laser Illumination and Ablation System. DNA damage was induced using a 365 nm wavelength laser. The energy and the frequency of the laser pulse is 170 mJ and 10 Hz respectively. Lasers were used to induce DNA damage and localization of MDC1-BRCT-GFP at the DNA damage sites were captured for up to 15 min for each mIR cell. Images were captured using the Cellsens software. Time for complete dissipation of the fluorescent signal at the DNA damage sites were captured for ten cells for each treatment subgroup and presented as a mean as shown in the results section.

### Clonogenic cell survival assay

Colony forming capacity of the cancer cells were assessed using the clonogenic cell survival assay as described previously (49). The effect of *ISG15* knockdown causing radiosensitization in different EAC cell lines were assessed using this technique.

### NHEJ I-SceI reporter assays

HR and NHEJ repair activity was detected by flowcytometry using I-SceI expressing adenovirus as described previously (24). This is a GFP based reporter assay which helps in determining the type of DNA repair taking place in cells.

### Comet Assay

An alkaline Comet assay was performed as reported (52). Briefly, frosted slides were coated with a layer of agarose and dried. About 2000 irradiated cells were suspended in PBS and mixed with triple the volume of 1% low melting point (LMP) agarose at 37 °C and spread over the agarose coated slides to make a thin layer. These slides were then kept in cold room and allowed to solidify for 4 h. The slides were then kept submerged in a highly alkaline lysis buffer (1.2 M NaCl, 100 mM Na_2_EDTA, 0.1% sodium lauryl sarcosinate, 0.26 M NaOH (pH > 13) overnight for (18–20 h) at 4 °C. Next day, the slides were rinsed three times at room temperature in alkaline rinse and electrophoresis solution (0.03 M NaOH, 2 mM Na_2_EDTA pH 12.3) to remove excess salts from the lysis buffer. The slides were then placed onto a horizontal electrophoresis camber and electrophoresed for 25 min at a voltage of 0.6 V/cm. The slides were then rinsed again with distilled water to remove salts. The gels on the slides were now stained with 2.5 μg/ml of propidium iodide (PI) dissolved in distilled water for 20 min. The slides were rinsed once more with distilled water to remove excess PI and dried overnight in the cold room. Next day, fluorescence images were captured under microscope. For each treatment, 50 comet images were captured, and the olive tail moments were calculated using Comet Assay IV, Instem software (Staffordshire, UK). The average tail moment was calculated for control and ISG15 siRNA treated groups at different time points (1, 6, and 24 h post-irradiation) and were subjected to statistical analysis.

### Cell synchronization studies

Synchronization of cell cycle was done using a double-thymidine block protocol as mentioned previously (53). Briefly, cells were maintained in 2 mM of thymidine for 18 h followed by a release in fresh media for 9 h. The cell cycle was then blocked again using the same concentration of thymidine for the next 15 h. Finally, the G1 synchronized cells were released again and collected every 2 h, subjected to mIR to investigate the effect of cell cycle on the DNA repair capacity using MDC1-BRCT-GFP transfected U2OS cells. Cell lysates were also prepared and subjected to immunoblotting at each time point up to 14 h.

### Co-immunoprecipitation

Co-immunoprecipitation was performed according to the protocol mentioned previously (49). Briefly, at least 400 μg of protein from each cell lysate were normalized to equal volumes followed by overnight incubation with the primary antibody as indicated. Sepharose Protein A/G beads or DDK-tagged agarose beads were used to precipitate target proteins followed by immunoblotting using respective antibodies.

### Mass spectrometry

Immunoprecipitated samples were separated on a polyacrylamide gel, and the gel was stained with colloidal Coomassie. In-gel digestion followed by identification of post-translationally modified sites was performed similarly as described previously (54). After trypsin digestion, peptides were resolved on a nano-capillary reverse phase column, and eluent was directly introduced into Orbitrap Fusion tribrid mass spectrometer (Thermo Scientific) using an EasySpray source. MS1 scans were acquired at 120K resolution (AGC target = 1 × 10^6^; max IT=50 ms). Data-dependent collision-induced dissociation MS/MS spectra were acquired using Top speed method (3 s) following each MS1 scan (NCE ∼32%; AGC target 1 × 10^5^; max IT 45 ms). Proteins were identified by searching for the data against UniProt *H*. *sapien* protein database using Proteome Discoverer (v3.0, Thermo Scientific). Search parameters included MS1 mass tolerance of 10 ppm and fragment tolerance of 0.05 Da; two missed cleavages were allowed; carbamidimethylation of cysteine, oxidation of methionine, and deamidation of asparagine and glutamine, ubiquitin remnant (K-gg; +114.043 Da) on lysine were considered as potential modifications. Percolator node of Proteome Discoverer was used to filter and retain high-quality proteins/peptides. Spectral matches to ubiquitinated/ISGylated peptides were manually verified.

### Tandem mass tags (TMT_pro_)

For our studies, we have purchased the TMT_pro_ 16-plex kit from Invitrogen (Cat# A44520) and used it according to the manufacturer’s protocol. Briefly, we used Flo1 cells transfected either with control or *ISG15* siRNAs and were then exposed to either sham or 4 Gy. Samples were collected 6 h post-IR and equal (75 mg) amounts of proteins were labeled using specific isotopes using a multiplex TMT kit. Mass spectrometry was performed at the University of Michigan Proteomic Resource Facility using their optimized protocol. Briefly, after trypsin digestion the samples were labeled with TMTpro reagents as per the manufacturer’s protocol. After combining the TMT-labeled samples, an offline fractionation into eight fractions was performed using high pH reversed-phase peptide fractionation kit (Pierce; Cat #84868). Fractions were dried and reconstituted in 9 ml. Orbitrap Ascend Tribrid equipped with FAIMS source (Thermo Fisher Scientific) and Vanquish Neo UHPLC were used to acquire the data. Two of the sample was resolved on an Easy-Spray PepMap Neo column (75 μm i.d. × 50 cm; Thermo Scientific) at the flow-rate of 300 nl/min using 0.1% formic acid/acetonitrile gradient system (3–19% acetonitrile in 72 min;19–29% acetonitrile in 28 min; 29–41% in 20 min followed by 10 min column wash at 95% acetonitrile and re-equilibration) and directly spray onto the mass spectrometer using EasySpray source (Thermo Fisher Scientific). FAIMS source was operated in standard resolution mode, with a nitrogen gas flow of 4.2 L/min, and inner and outer electrode temperature of 100 °C and dispersion voltage or −5000 V. Two compensation voltages (CVs) of −45 and −65 V, 1.5 s per CV, were employed to select ions that enter the mass spectrometer for MS1 scan and MS/MS cycles. Mass spectrometer was set to collect MS1 scan (Orbitrap; 400–1600 m/z; 120K resolution; AGC target of 100%; max IT in Auto) following which precursor ions with charge states of 2 to 6 were isolated by quadrupole mass filter at 0.7 m/z width and fragmented by collision induced dissociation in ion trap (NCE 30%; normalized AGC target of 100%; max IT 35 ms). For multinotch-MS3, the top 10 precursors from each MS2 were fragmented by HCD followed by Orbitrap analysis (NCE 55; 45K resolution; normalized AGC target of 200%; max IT 200 ms, 100–500 m/z scan range).

### TMT data analysis

Proteome Discoverer (v3.0; Thermo Fisher) was used for data analysis. MS2 spectra were searched against SwissProt human protein database (20,347 entries; sp_canonical TaxID=9606 (v2023-03-01)) using the following search parameters: MS1 and MS2 tolerance were set to 10 ppm and 0.6 Da, respectively; carbamidomethylation of cysteines (57.02146 Da) and TMTpro labeling of lysine and N-termini of peptides were considered static modifications; oxidation of methionine (15.9949 Da) and deamidation of asparagine and glutamine (0.98401 Da) were considered variable. Identified proteins and peptides were filtered to retain only those that passed ≤1% FDR threshold. Quantitation was performed using high-quality MS3 spectra (Average signal-to-noise ratio of 10 and <60% isolation interference). Cell lysates from three independent replicates were used and compared to identify reliable proteomic alterations (up and downregulated proteins) with statistical significance (*p* > 0.05).

### Patients’ characteristics, establishment of TMA, and IHC

The resected tumor samples were collected from EAC patients (n = 113) who underwent neoadjuvant therapy and subsequent esophagectomy at the University of Michigan. Human studies reported here were abiding by the Declaration of Helsinki principles. The TMA was constructed with tissues from patients who demonstrated residual tumor following resection as determined by a pathologist. Tissue microarray (TMA) was established as described earlier (15) with each patient represented by three independent tumor tissue cores. The TMA was then subjected to immunohistochemical (IHC) staining using a human RAD51 antibody. For quantification, stained slides were digitized using a Leica-Aperio AT2 scanner (Leica, Deer Park, IL) at a maximum magnification of 200x. Digital images were uploaded to QuPath v0.5 (55). The TMA de-arrayer was used according to the software instructions and annotated using the TMA map. Positive cell detection was then applied to determine the number of positive cells. Based on clinical parameters, we identified 57 patients as node-positive and used for analysis.

### Statistical analysis

We examined distribution of ISG15 and RAD51, as well as clinical (T-stage, N-stage, M-stage) and demographic factors (age, gender, race, history of smoking) for 113 chemoresistant patients with EAC that included 57 node-positive patients in the dataset. Survival time was defined as time between the date-of surgery and the date of death or last contact. We use the median of RAD51 distribution to define low and high RAD51 groups. We proceeded with computing nonparametric estimates of the survivor function Kaplan-Meier method and used log-rank test to test the difference between low and high values of RAD51. Finally, we used Cox proportional hazards model to estimate hazard ration associated with high values of RAD51, adjusted for clinical and demographic factors. All these analyses were conducted using SAS 9.4 software.

For other data analyses, we have used GraphPad Prism v 10.2.2. Statistical analysis between different ISG15 and H2AX mutant groups, we have performed One-Way ANOVA analysis followed by Tukey’s multiple comparison test to determine significant differences upon incorporation of different mutations.

## Data availability

All data presented in this manuscript are predominantly included in the Main Figures, Supporting Figures (S1-S10) and Supporting Table S1. We have further uploaded raw MS data (H2AX ISGylation and TMT set) in PRIDE with accession# PXD071548).

## Supporting information

This article contains Supporting Information (Figs. S1-S10 and a table).

## Conflict of interest

The authors declare that they have no conflicts of interest with the contents of this article.

## References

[1] Coleman H.G., Xie S.H., Lagergren J. (2018). The epidemiology of esophageal adenocarcinoma. Gastroenterology.

[2] Ferlay J., Colombet M., Soerjomataram I., Mathers C., Parkin D.M., Pineros M. (2019). Estimating the global cancer incidence and mortality in 2018: GLOBOCAN sources and methods. Int. J. Cancer.

[3] Kroep S., Lansdorp-Vogelaar I., Rubenstein J.H., Lemmens V.E., van Heijningen E.B., Aragones N. (2014). Comparing trends in esophageal adenocarcinoma incidence and lifestyle factors between the United States, Spain, and the Netherlands. Am. J. Gastroenterol..

[4] Njei B., McCarty T.R., Birk J.W. (2016). Trends in esophageal cancer survival in United States adults from 1973 to 2009: a SEER database analysis. J. Gastroenterol. Hepatol..

[5] Shapiro J., van Lanschot J.J.B., Hulshof M., van Hagen P., van Berge Henegouwen M.I., Wijnhoven B.P.L. (2015). Neoadjuvant chemoradiotherapy plus surgery versus surgery alone for oesophageal or junctional cancer (CROSS): long-term results of a randomised controlled trial. Lancet Oncol..

[6] Huang H., Fang W., Lin Y., Zheng Z., Wang Z., Chen X. (2021). Predictive model for overall survival and cancer-specific survival in patients with esophageal adenocarcinoma. J. Oncol..

[7] van der Zijden C.J., Bouwman A., Mostert B., Nuyttens J., van der Sluis P.C., Spaander M.C.W. (2024). Overall survival after definitive chemoradiotherapy for patients with esophageal cancer: a retrospective cohort study. Dis. Esophagus.

[8] Hulshof M., Geijsen E.D., Rozema T., Oppedijk V., Buijsen J., Neelis K.J. (2021). Randomized Study on dose escalation in definitive chemoradiation for patients with locally advanced esophageal cancer (ARTDECO Study). J. Clin. Oncol..

[9] Hoeppner J., Brunner T., Schmoor C., Bronsert P., Kulemann B., Claus R. (2025). Perioperative chemotherapy or preoperative chemoradiotherapy in esophageal cancer. New Engl. J. Med..

[10] Freitas B.T., Scholte F.E.M., Bergeron E., Pegan S.D. (2020). How ISG15 combats viral infection. Virus Res..

[11] Perng Y.C., Lenschow D.J. (2018). ISG15 in antiviral immunity and beyond. Nat. Rev. Microbiol..

[12] Villarroya-Beltri C., Baixauli F., Mittelbrunn M., Fernandez-Delgado I., Torralba D., Moreno-Gonzalo O. (2016). ISGylation controls exosome secretion by promoting lysosomal degradation of MVB proteins. Nat. Commun..

[13] Dzimianski J.V., Scholte F.E.M., Bergeron E., Pegan S.D. (2019). ISG15: it's complicated. J. Mol. Biol..

[14] Swaim C.D., Scott A.F., Canadeo L.A., Huibregtse J.M. (2017). Extracellular ISG15 signals cytokine secretion through the LFA-1 integrin receptor. Mol. Cell.

[15] McEwen D.P., Ray P., Nancarrow D.J., Wang Z., Kasturirangan S., Abdullah S. (2024). ISG15-GRAIL1-CD3 axis influences survival of esophageal adenocarcinoma patients. JCI Insight.

[16] Wardlaw C.P., Petrini J.H.J. (2022). ISG15 conjugation to proteins on nascent DNA mitigates DNA replication stress. Nat. Commun..

[17] Huang R., Zhou P.K. (2021). DNA damage repair: historical perspectives, mechanistic pathways and clinical translation for targeted cancer therapy. Signal Transduct Target Ther..

[18] Podhorecka M., Skladanowski A., Bozko P. (2010). H2AX phosphorylation: its role in DNA damage response and cancer therapy. J. Nucleic Acids.

[19] Baumann P., West S.C. (1998). Role of the human RAD51 protein in homologous recombination and double-stranded-break repair. Trends Biochem. Sci..

[20] Liu M., Hummer B.T., Li X., Hassel B.A. (2004). Camptothecin induces the ubiquitin-like protein, ISG15, and enhances ISG15 conjugation in response to interferon. J. Interferon Cytokine Res..

[21] Park J.H., Yang S.W., Park J.M., Ka S.H., Kim J.H., Kong Y.Y. (2016). Positive feedback regulation of p53 transactivity by DNA damage-induced ISG15 modification. Nat. Commun..

[22] Feng X., Tubbs A., Zhang C., Tang M., Sridharan S., Wang C. (2020). ATR inhibition potentiates ionizing radiation-induced interferon response via cytosolic nucleic acid-sensing pathways. EMBO J..

[23] Chen Y., Zhou C., Ji W., Mei Z., Hu B., Zhang W. (2016). ELL targets c-Myc for proteasomal degradation and suppresses tumour growth. Nat. Commun..

[24] Zhang Q., Karnak D., Tan M., Lawrence T.S., Morgan M.A., Sun Y. (2016). FBXW7 facilitates nonhomologous end-joining via K63-Linked polyubiquitylation of XRCC4. Mol. Cell.

[25] Dragojevic S., Smith E.J., Regan M.S., Stopka S.A., Baquer G., Xue Z. (2025). DNA-PK inhibition shows differential radiosensitization in orthotopic GBM PDX models based on DDR pathway deficits. Mol. Cancer Ther..

[26] Lee M.S., Edwards R.A., Thede G.L., Glover J.N. (2005). Structure of the BRCT repeat domain of MDC1 and its specificity for the free COOH-terminal end of the gamma-H2AX histone tail. J. Biol. Chem..

[27] Pan M.R., Peng G., Hung W.C., Lin S.Y. (2011). Monoubiquitination of H2AX protein regulates DNA damage response signaling. J. Biol. Chem..

[28] Huang W., Ray P., Ji W., Wang Z., Nancarrow D., Chen G. (2020). The cytochrome P450 enzyme CYP24A1 increases proliferation of mutant KRAS-dependent lung adenocarcinoma independent of its catalytic activity. J. Biol. Chem..

[29] Zhao X., Wei C., Li J., Xing P., Li J., Zheng S. (2017). Cell cycle-dependent control of homologous recombination. Acta Biochim. Biophys. Sin (Shanghai).

[30] Pawlik T.M., Keyomarsi K. (2004). Role of cell cycle in mediating sensitivity to radiotherapy. Int. J. Radiat. Oncol. Biol. Phys..

[31] Chen J., Laverty D.J., Talele S., Bale A., Carlson B.L., Porath K.A. (2024). Aberrant ATM signaling and homology-directed DNA repair as a vulnerability of p53-mutant GBM to AZD1390-mediated radiosensitization. Sci. Transl. Med..

[32] Li X., Heyer W.D. (2008). Homologous recombination in DNA repair and DNA damage tolerance. Cell Res..

[33] Wu R., Patel A., Tokumaru Y., Asaoka M., Oshi M., Yan L. (2022). High RAD51 gene expression is associated with aggressive biology and with poor survival in breast cancer. Breast Cancer Res. Treat.

[34] Tsai Y.F., Chan L.P., Chen Y.K., Su C.W., Hsu C.W., Wang Y.Y. (2023). RAD51 is a poor prognostic marker and a potential therapeutic target for oral squamous cell carcinoma. Cancer Cell Int..

[35] Takahashi K., Yan L., An N., Chida K., Tian W., Oshi M. (2024). RAD51 high-expressed hepatocellular carcinomas are associated with high cell proliferation. J. Surg. Res..

[36] Park J.M., Yang S.W., Yu K.R., Ka S.H., Lee S.W., Seol J.H. (2014). Modification of PCNA by ISG15 plays a crucial role in termination of error-prone translesion DNA synthesis. Mol. Cell.

[37] Zierhut C. (2024). Potential cGAS-STING pathway functions in DNA damage responses, DNA replication and DNA repair. DNA Repair (Amst).

[38] Her J., Bunting S.F. (2018). How cells ensure correct repair of DNA double-strand breaks. J. Biol. Chem..

[39] Leadon S.A. (1996). Repair of DNA damage produced by ionizing radiation: a minireview. Semin. Radiat. Oncol..

[40] Li Y.H., Wang X., Pan Y., Lee D.H., Chowdhury D., Kimmelman A.C. (2012). Inhibition of non-homologous end joining repair impairs pancreatic cancer growth and enhances radiation response. PLoS One.

[41] Sishc B.J., Davis A.J. (2017). The role of the core non-homologous end joining factors in carcinogenesis and cancer. Cancers (Basel).

[42] Manthei K.A., Keck J.L. (2013). The BLM dissolvasome in DNA replication and repair. Cell Mol. Life Sci..

[43] Wojnicki K., Kaczmarczyk A., Wojtas B., Kaminska B. (2023). BLM helicase overexpressed in human gliomas contributes to diverse responses of human glioma cells to chemotherapy. Cell Death Discov..

[44] Liu X., Dong R., Jiang Z., Wei Y., Li Y., Wei L. (2015). MDC1 promotes ovarian cancer metastasis by inducing epithelial-mesenchymal transition. Tumour. Biol..

[45] Wang Z., Jia R., Wang L., Yang Q., Hu X., Fu Q. (2022). The emerging roles of Rad51 in cancer and its potential as a therapeutic target. Front Oncol..

[46] Makino E., Frohlich L.M., Sinnberg T., Kosnopfel C., Sauer B., Garbe C. (2020). Targeting Rad51 as a strategy for the treatment of melanoma cells resistant to MAPK pathway inhibition. Cell Death Dis..

[47] Kim Y.N., Kim K., Joung J.G., Kim S.W., Kim S., Lee J.Y. (2024). RAD51 as an immunohistochemistry-based marker of poly(ADP-ribose) polymerase inhibitor resistance in ovarian cancer. Front Oncol..

[48] Ray P., Jaiswal S., Ferrer-Torres D., Wang Z., Nancarrow D., Curtin M. (2024). GRAIL1 stabilizes misfolded mutant p53 through a ubiquitin ligase-independent, chaperone regulatory function. Mol Cancer Res.

[49] Ray D., Ahsan A., Helman A., Chen G., Hegde A., Gurjar S.R. (2011). Regulation of EGFR protein stability by the HECT-type ubiquitin ligase SMURF2. Neoplasia.

[50] Bektas N., Noetzel E., Veeck J., Press M.F., Kristiansen G., Naami A. (2008). The ubiquitin-like molecule interferon-stimulated gene 15 (ISG15) is a potential prognostic marker in human breast cancer. Breast Cancer Res..

[51] Shukla S., Allam U.S., Ahsan A., Chen G., Krishnamurthy P.M., Marsh K. (2014). KRAS protein stability is regulated through SMURF2: UBCH5 complex-mediated beta-TrCP1 degradation. Neoplasia.

[52] Olive P.L., Banath J.P. (2006). The comet assay: a method to measure DNA damage in individual cells. Nat. Protoc..

[53] Osmundson E.C., Ray D., Moore F.E., Gao Q., Thomsen G.H., Kiyokawa H. (2008). The HECT E3 ligase Smurf2 is required for Mad2-dependent spindle assembly checkpoint. J. Cell Biol..

[54] Maine G.N., Li H., Zaidi I.W., Basrur V., Elenitoba-Johnson K.S., Burstein E. (2010). A bimolecular affinity purification method under denaturing conditions for rapid isolation of a ubiquitinated protein for mass spectrometry analysis. Nat. Protoc..

[55] Bankhead P., Loughrey M.B., Fernandez J.A., Dombrowski Y., McArt D.G., Dunne P.D. (2017). QuPath: open source software for digital pathology image analysis. Sci. Rep..

[56] Arnoult N., Correia A., Ma J., Merlo A., Garcia-Gomez S., Maric M. (2017). Regulation of DNA repair pathway choice in S and G2 phases by the NHEJ inhibitor CYREN. Nature.

