# Supplementary material for: ISGylation of γH2AX retains MDC1 and facilitates homologous recombination repair causing radioresistance in esophageal adenocarcinoma
Source: J Biol Chem. 2026 Mar 9;302(5):111358. doi: 10.1016/j.jbc.2026.111358 (PMC13084363; doi:10.1016/j.jbc.2026.111358)
Supplement: Supporting Figures [file mmc1.pdf]

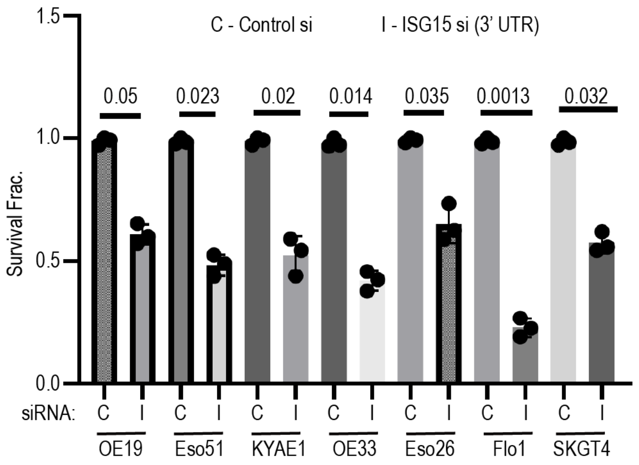

Supporting Fig. S1. *ISG15* siRNA directed against 3' UTR reduces clonogenic survival of EAC cells. EAC cells as indicated were transfected with either control (C) or ISG15 (I) siRNA directed against the 3' UTR of the gene. Forty-eight hours post-transfection, cells were trypsinized and plated at clonal density to perform clonogenic assay as described in materials and methods. Pairwise comparisons showing corresponding p-values, all showing significance.

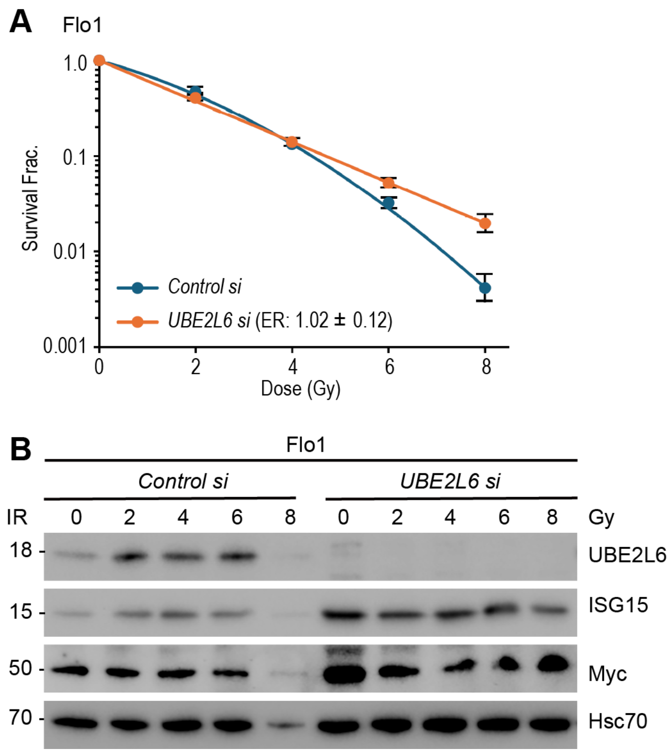

Supporting Fig. S2. Loss of *UBE2L6* fail to radiosensitize EAC cells. (**A**) Flo1 cells were transfected with either control or UBE2L6 siRNAs. Firty-eight hours post-transfection, cells were subjected to different doses of ionizing radiation (0, 2, 4, 6, and 8 Gy) and 24 h post-irradiation, cells were trypsinized and plated for clonogenic survival. Representative curve from an experiment showing an enhancement ratio (ER: 1.02±0.12) are shown. (**B**) Immunoblotting of isolated samples showing loss of UBE2L6/UBCH8 that resulted in c-Myc upregulation.

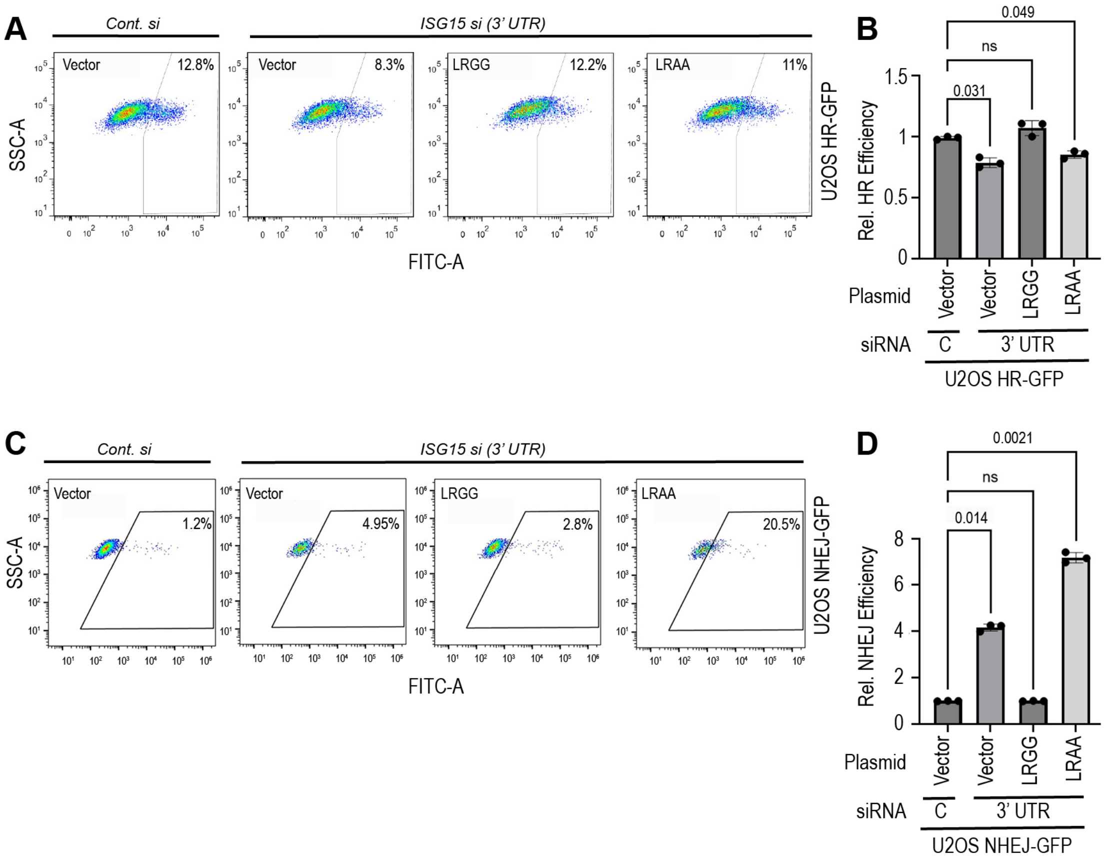

Supporting Fig. S3. Loss of ISG15 leads to loss of HR and compensatory activation of NHEJ in U2OS DDR reporter cell lines. (**A** & **C**) U2OS DR-GFP (in A) or U2OS NHEJ-GFP (in C) cell lines were transfected either with control or *ISG15* siRNAs followed by overexpression of either vector, ISG15 WT (LRGG) or conjugation deficient (LRAA) mutant as shown. To induce double stranded DNA breaks, cells were transduced with Adenovirus overexpressing I-SecI endonuclease. Cells were further cultured for 48 h and collected for FACS analysis to quantify repair abilities via two different repair mechanisms. (**B** & **D**) Relative HR and NHEJ repair efficiency were quantified considering control siRNA sample as '1'.

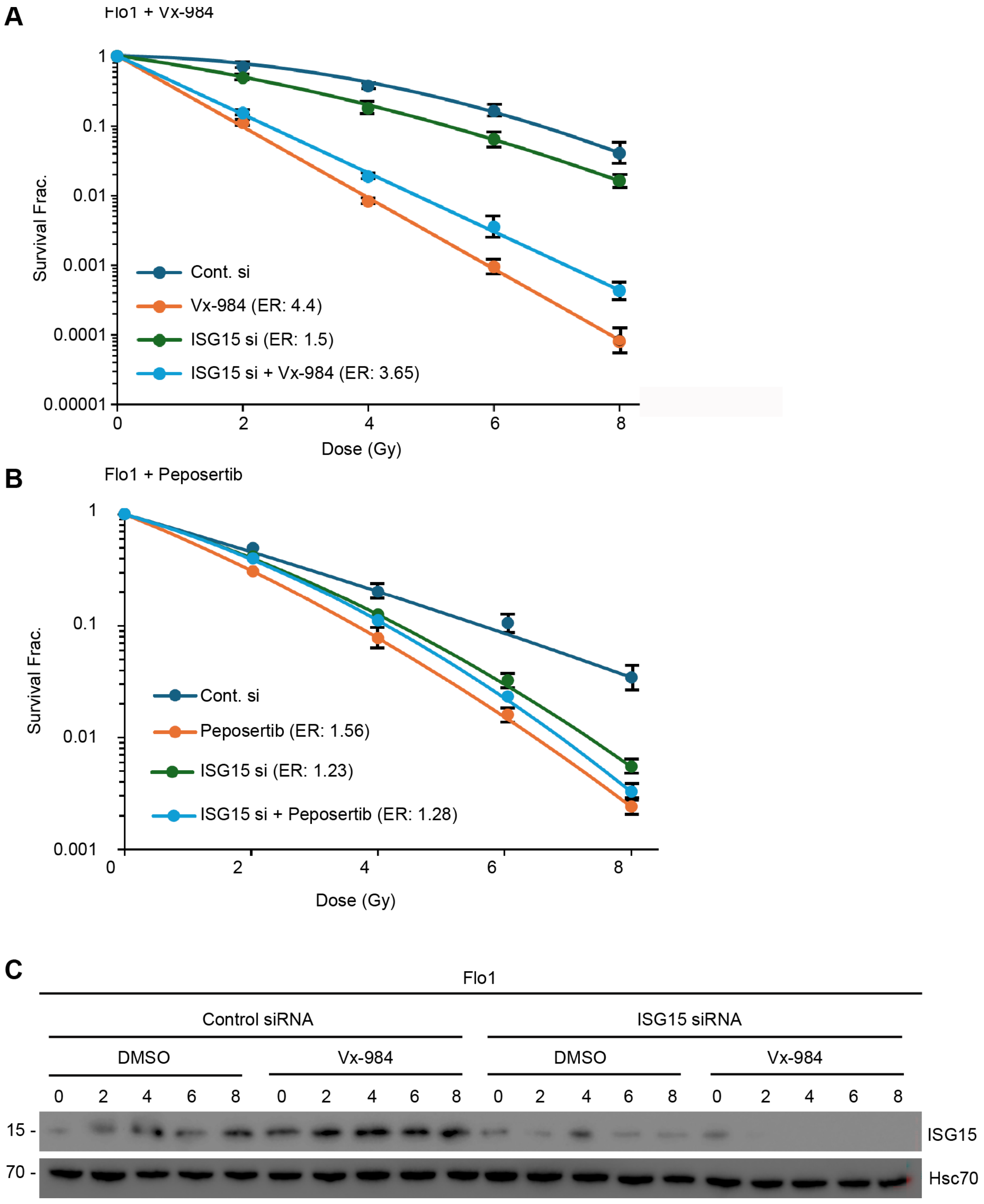

Supporting Fig. S4. ISG15 expression is correlated with increased hazard ratio in node-positive EAC patients. (**A**) Flo1 cells were first transfected with either control or ISG15 siRNAs and 48 h post-transfection, cells were then further treated DNA-PKcs inhibitors (Vx-984 - 1 mM; peposertib - 500 nM) an hour prior to IR (0, 2, 4, 6, 8 Gy). Twenty-four hours post-irradiation, cells were trypsinized plated for clonogenic survival assays. Results showing radiosensitizing potential of DNA-PKcs inhibitors in the presence and absence of ISG15 as indicated by enhancement ratio (ER). (**B**) Cell lysates from the above study were subjected to immunoblotting using indicated antibodies, confirming ISG15 loss.

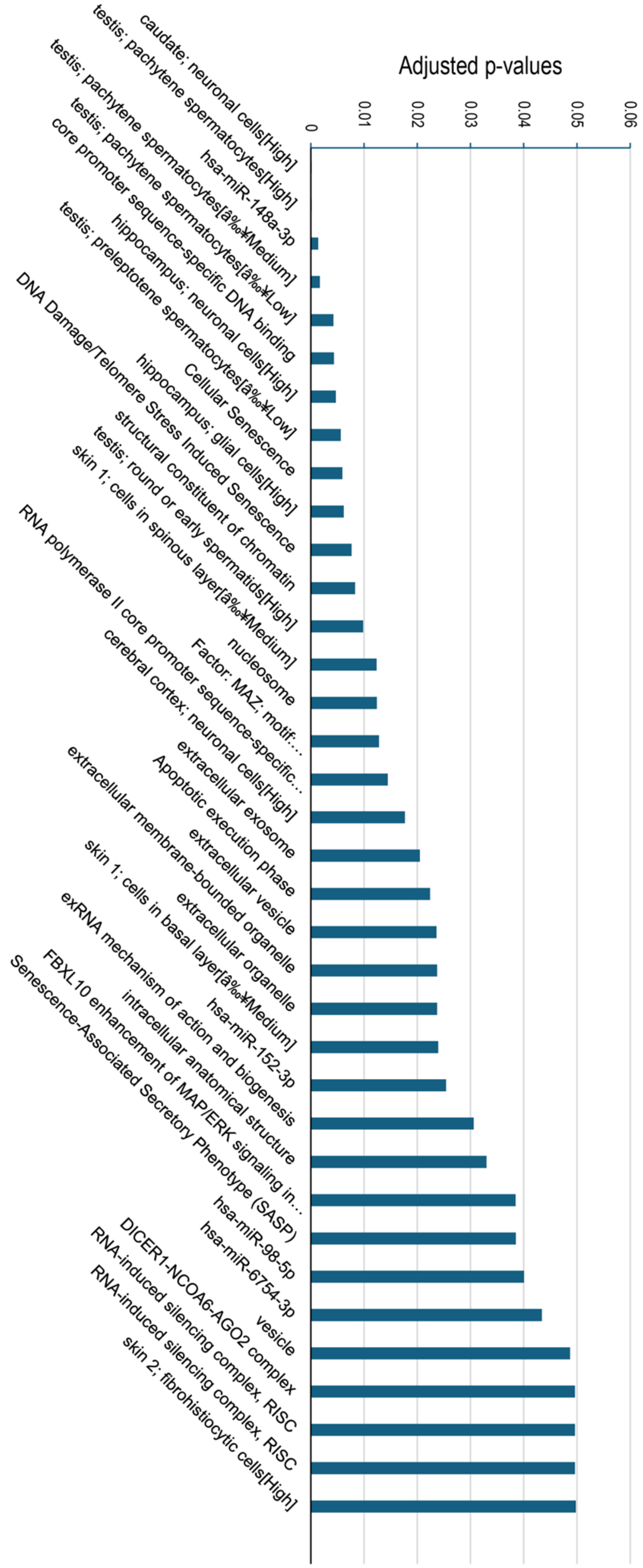

Supporting Fig. S5. Altered pathways identified based on up and downregulated proteins from the TMT array studies isolated from control and *ISG15* siRNA treated cells exposed to 4 Gy and collected after 6 h. Adjusted *p*-values (up to 0.05) were considered significant.

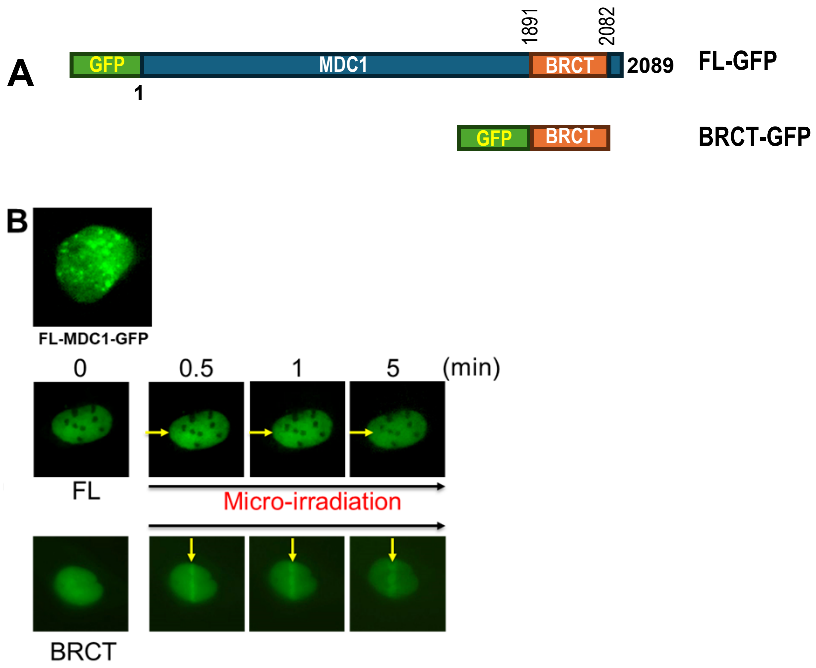

Supporting Fig. S6. Construction of GFP-MDC1-BRCT. (**A**) Schematic structure of full-length (FL) and BRCT domain containing human MDC1-GFP. (**B**) U2OS cells were stably transfected with either of the two constructs and subjected to microIR studies. Accumulation of BRCT-only MDC1-GFP at the DNA damage sites were evident.

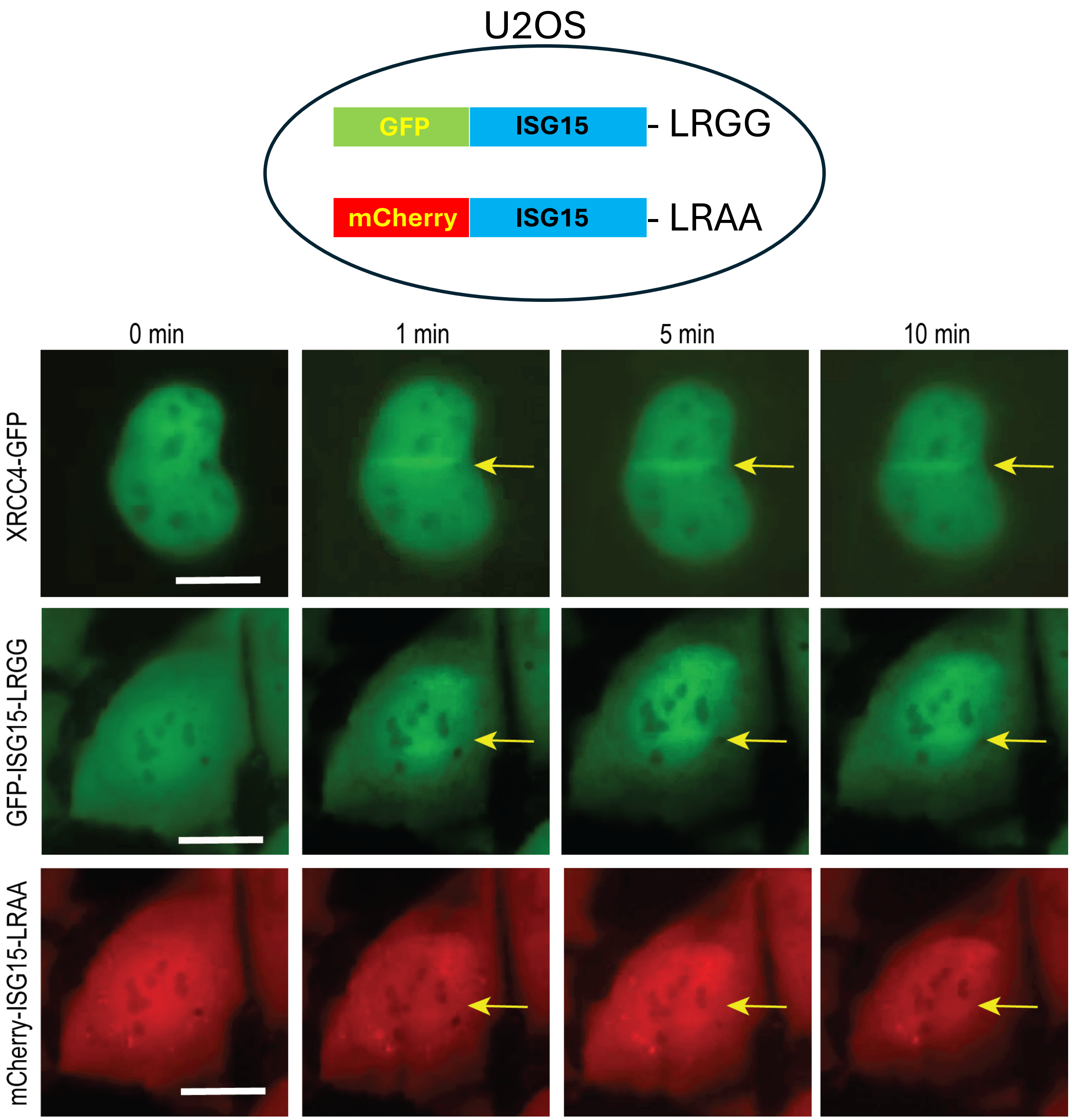

Supporting Fig. S7. Localization of ISGylation proficient (LRGG) ISG15 at the site of DNA damage. U2OS cells were co-transfected with GFP-ISG15 (LRGG) and mCherry-ISG15 (LRAA). Cells were subjected to microIR showing accumulation of GFP-ISG15 at the DNA damage sites, but the LRAA mutant failed to show similar accumulation. GFP-XRCC was used as a positive control.

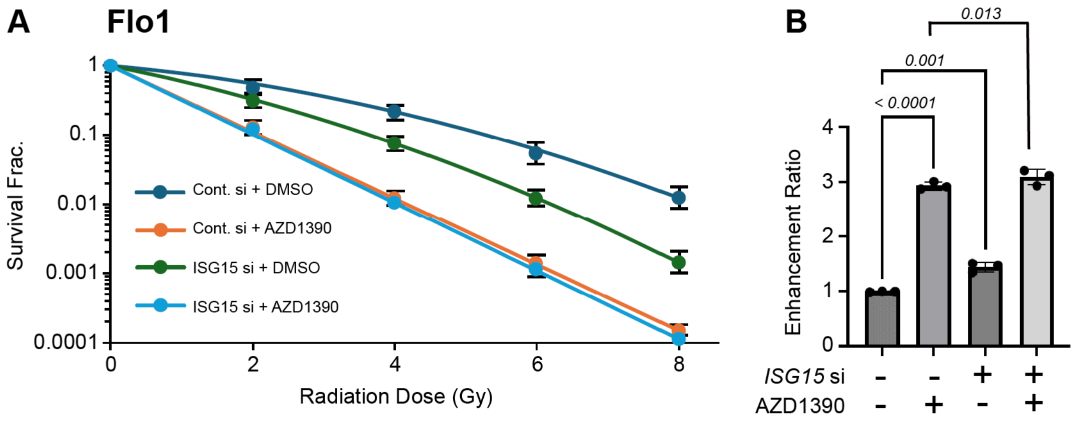

Supporting Fig. S8. Loss of *ISG15* and inhibition of ATM kinase activity radiosensitizes Flo1 cells. (A) FLO1 cells were transfected with either control or *ISG15* siRNAs. Forty-eight hours post-transfection, cells were treated with vehicle (DMSO) or the ATM inhibitor AZD1390 (100 nM). Two hours after drug treatment, cells were exposed to 4 Gy irradiation. Twenty-four hours post-irradiation, cells were replated for clonogenic survival as described in the Materials and Methods. Representative results demonstrate the radiosensitizing effects of *ISG15* knockdown and ATM inhibition, both as single agents and in combination. (**B**) The experiment was repeated three independent times to calculate enhancement ratios (ER), and data are presented as mean ± SEM. Paired comparisons were performed, and p values are indicated. The difference between AZD1390 treatment alone (ER: 2.84 ± 0.06) and combined *ISG15* knockdown plus AZD1390 treatment (ER: 3.11 ± 0.06) was also statistically significant (p = 0.013).

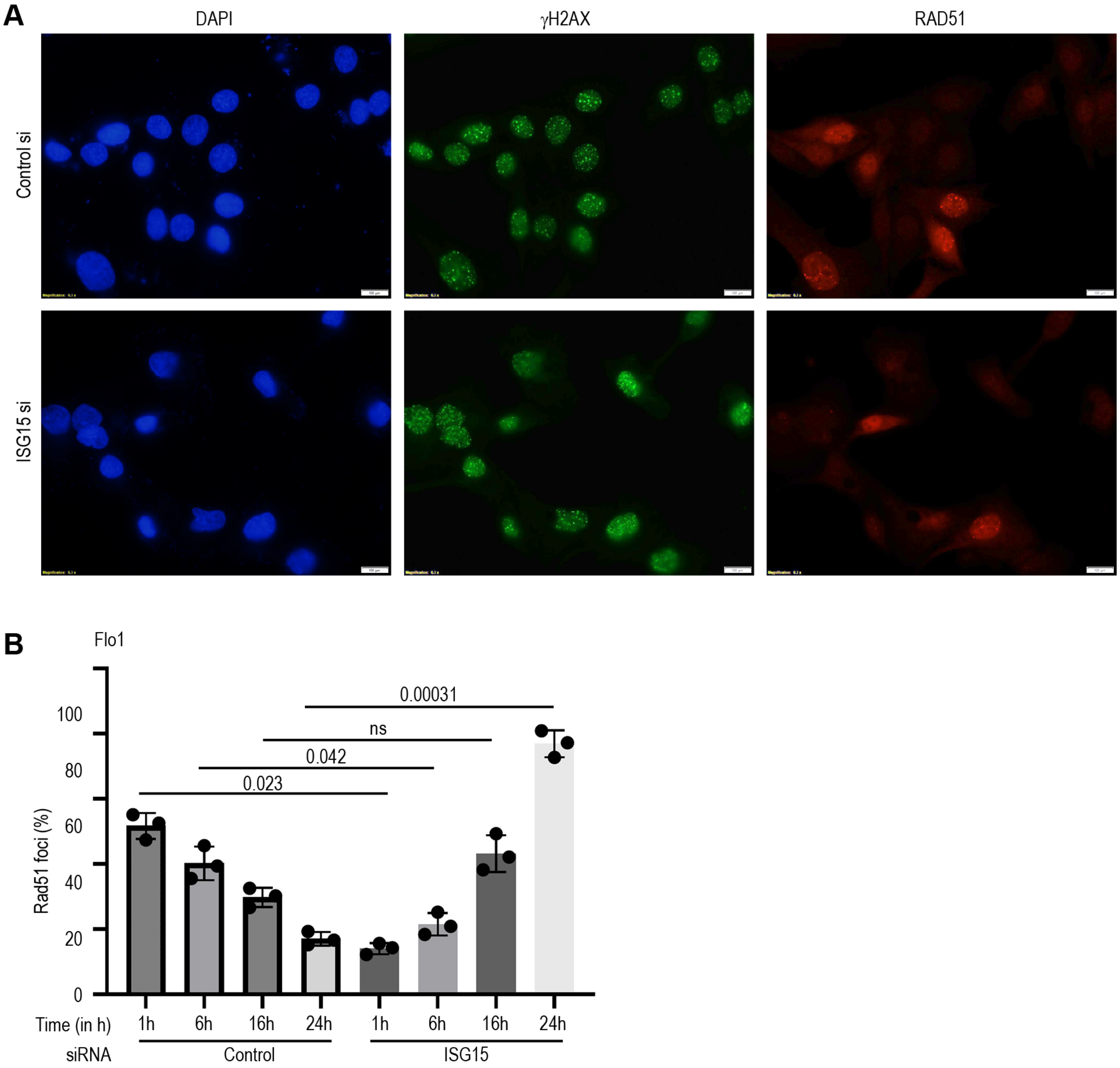

Supporting Fig. S9. Loss of ISG15 delays RAD51 foci formation at DNA damage sites. (**A**) Flo1 cells were transfected with either control or *ISG15* siRNAs and 48 hours post-transfection, cells were exposed to 4 Gy. At different times (0, 1, 6, 16, and 24h) post-irradiation, cells were fixed and stained using both γH2AX and RAD51 antibodies as described in materials and methods. DAPI (blue) was used to counterstain nuclei to calculate total numbers of cells. (**B**) Cells showing more than 5 foci for RAD51 were scored, and percentage positive cells were calculated at different time points post-irradiation transfected either with control (C) or *ISG15* (I) siRNAs as indicated. Paired comparison showing p values. ns – non-significant.

A

Risk Factor	Label	HR (95% CL)	p-value
ISG15% (>10)	High	0.72 (0.35,1.47)	0.3674
Age @ surgery		0.98 (0.95,1.02)	0.3681
T Stage (ref T1)	T2	1.26 (0.41,3.88)	0.6807
T Stage	T3	1.02 (0.40,2.62)	0.9710
N Stage (ref N1)	N2	2.54 (1.21,5.32)	0.0138
M Stage (ref M0)	M1	0.53 (0.07,4.06)	0.5424
Sex	Female	0.67 (0.15,3.08)	0.6080
Race	Non-white	0.36 (0.08,1.69)	0.1950
Smoking status	Ever	2.41 (0.97,6.02)	0.0585

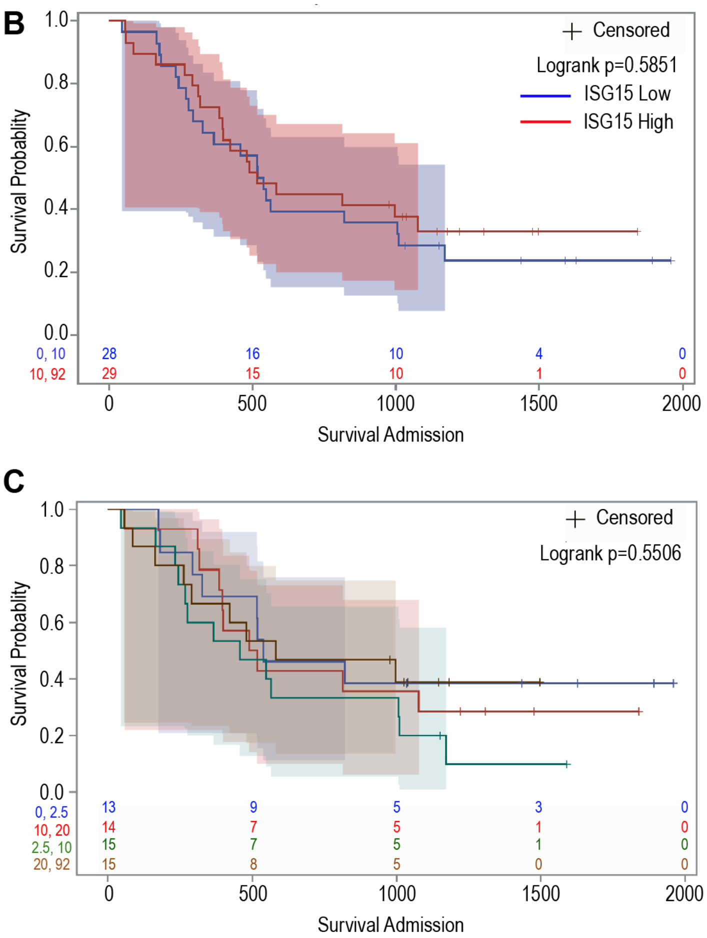

Supporting Fig. S10. ISG15 expression is correlated with increased hazard ratio in node-positive EAC patients. Similar to Fig. 5, chemoresistant TMA was subjected to IHC staining using ISG15 antibody, scanned and quantified to determine percentage ISG15 positive cells and was used for clinical correlation analysis among the node-positive EAC patients (n=57). (**A**) Results showing hazard ratio (HR) and corresponding p-values of multivariate analysis based on criteria as listed. (**B**) Kaplan-Meier survival curve with 95% Hall-Wellner confidence bands among node-positive (n=57) EAC patients grouped by ISG15 expression levels. High expression (red) levels were defined as values above the cohort's median expression (10-92) level, while low (blue) was defined as below the median expression (0-10). (**C**) Above samples were subjected to quartile analysis based on ISG15 staining intensity and plotted for Kaplan-Meier survival curves.
