# Supplementary material for: ISGylation of γH2AX retains MDC1 and facilitates homologous recombination repair causing radioresistance in esophageal adenocarcinoma
Source: J Biol Chem. 2026 Mar 9;302(5):111358. doi: 10.1016/j.jbc.2026.111358 (PMC13084363; doi:10.1016/j.jbc.2026.111358)
Supplement: Supporting Table S1 [file mmc2.pdf]

Supporting Table 1: Protein names	Abundance Ratio (ISG15 si-RT)/(Control si-RT)	P-Value (ISG15 si-RT)/(Control si-RT)
Ubiquitin-like protein ISG15 [OS=Homo sapiens]	0.191	1.03136E-09
Mitotic interactor and substrate of PLK1 [OS=Homo sapiens]	0.288	0.040269192
EF-hand domain-containing protein D1 [OS=Homo sapiens]	0.336	0.03477475
Zinc finger protein 860 [OS=Homo sapiens]	0.34	0.443310949
ATPase family AAA domain-containing protein 2 [OS=Homo sapiens]	0.366	0.169593764
Myotubularin-related protein 6 [OS=Homo sapiens]	s	4.58233E-10
Mis18-binding protein 1 [OS=Homo sapiens]	0.384	0.117475978
Bromodomain adjacent to zinc finger domain protein 1A [OS=Homo sapiens]	0.394	0.000375424
Ribosome biogenesis regulatory protein homolog [OS=Homo sapiens]	0.406	0.07446271
Vimentin [OS=Homo sapiens]	0.419	0.218910523
Histone H2A type 2-B [OS=Homo sapiens]	0.42	0.369478817
CCAAT/enhancer-binding protein beta [OS=Homo sapiens]	0.434	0.161639249
SURP and G-patch domain-containing protein 2 [OS=Homo sapiens]	0.438	0.04273389
Histone H3.3 [OS=Homo sapiens]	0.439	0.45190703
Histone H2A.Z [OS=Homo sapiens]	0.439	0.481521287
SAFB-like transcription modulator [OS=Homo sapiens]	0.442	0.179890682
Insulin, isoform 2 [OS=Homo sapiens]	0.442	0.897276349
Ribosome production factor 2 homolog [OS=Homo sapiens]	0.444	0.128239354
CTD small phosphatase-like protein 2 [OS=Homo sapiens]	0.444	0.113173718
U3 small nucleolar ribonucleoprotein protein MPP10 [OS=Homo sapiens]	0.445	0.165536888
Y-box-binding protein 2 [OS=Homo sapiens]	0.446	4.15694E-11
Protein AATF [OS=Homo sapiens]	0.448	0.821187736
Serine/threonine-protein kinase PLK1 [OS=Homo sapiens]	0.449	2.11871E-07
RNA-binding protein Raly [OS=Homo sapiens]	0.45	0.179565058
Dynein axonemal heavy chain 3 [OS=Homo sapiens]	0.45	0.657745241
Histone H1.1 [OS=Homo sapiens]	0.451	0.023011858
Lamin-B1 [OS=Homo sapiens]	0.454	0.274315321
Sentrin-specific protease 1 [OS=Homo sapiens]	0.456	0.333356531
Keratin, type II cytoskeletal 80 [OS=Homo sapiens]	0.457	0.073777451
ORC ubiquitin ligase 1 [OS=Homo sapiens]	0.467	0.000797321
Protein mono-ADP-ribosyltransferase PARP16 [OS=Homo sapiens]	0.469	2.34491E-05
Scaffold attachment factor B1 [OS=Homo sapiens]	0.487	0.326146319
Tumor necrosis factor alpha-induced protein 8 [OS=Homo sapiens]	0.492	2.14398E-06
Apoptotic chromatin condensation inducer in the nucleus [OS=Homo sapiens]	0.493	0.142138498
Histone H2AX [OS=Homo sapiens]	0.494	0.516012976
Core histone macro-H2A.1 [OS=Homo sapiens]	0.498	0.591213932
Capping protein-inhibiting regulator of actin dynamics [OS=Homo sapiens]	0.503	0.38014214
Inner centromere protein [OS=Homo sapiens]	0.505	0.093130511
HEAT repeat-containing protein 1 [OS=Homo sapiens]	0.509	0.033976489
Histone H2B type 1-K [OS=Homo sapiens]	0.509	0.501023855
Crooked neck-like protein 1 [OS=Homo sapiens]	0.509	0.039206286
Cell adhesion molecule 1 [OS=Homo sapiens]	0.509	0.004888524
Proliferation marker protein Ki-67 [OS=Homo sapiens]	0.511	0.069527411
Membrane protein FAM174B [OS=Homo sapiens]	0.513	9.978E-05
Nucleolar protein 8 [OS=Homo sapiens]	0.514	0.177231586
Scaffold attachment factor B2 [OS=Homo sapiens]	0.516	0.26370741
Baculoviral IAP repeat-containing protein 5 [OS=Homo sapiens]	0.516	0.177809085
U3 small nucleolar ribonucleoprotein protein IMP4 [OS=Homo sapiens]	0.517	0.391960324
Core histone macro-H2A.2 [OS=Homo sapiens]	0.518	0.278876819
Pinin [OS=Homo sapiens]	0.522	0.202169608
Serine/threonine-protein kinase PRP4 homolog [OS=Homo sapiens]	0.523	0.136651995
DBIRD complex subunit ZNF326 [OS=Homo sapiens]	0.523	0.290102248
Inhibitor of Bruton tyrosine kinase [OS=Homo sapiens]	0.523	4.4026E-07
Zinc finger CCCH domain-containing protein 11A [OS=Homo sapiens]	0.525	0.058176275
Histone H4 [OS=Homo sapiens]	0.526	0.44900666
Periphilin-1 [OS=Homo sapiens]	0.526	0.249553901
RNA-binding motif protein, X chromosome [OS=Homo sapiens]	0.527	0.384304912
Cytochrome c oxidase subunit 6A1, mitochondrial [OS=Homo sapiens]	0.527	0.571241547
NF-kappa-B-activating protein [OS=Homo sapiens]	0.528	0.000104173
Keratin, type I cytoskeletal 19 [OS=Homo sapiens]	0.529	0.136528004
Chromobox protein homolog 5 [OS=Homo sapiens]	0.53	0.005050219
Histone H2B type 1-J [OS=Homo sapiens]	0.532	0.59907599
Heterogeneous nuclear ribonucleoproteins C1/C2 [OS=Homo sapiens]	0.533	0.386960978
WD repeat-containing protein 75 [OS=Homo sapiens]	0.533	0.04426587
Nucleolar complex protein 3 homolog [OS=Homo sapiens]	0.535	0.398680095
Protein SON [OS=Homo sapiens]	0.536	0.277520194
Protein NPAT [OS=Homo sapiens]	0.537	0.487006109
ATP-dependent RNA helicase DDX18 [OS=Homo sapiens]	0.538	0.112621949
Tyrosine-protein kinase BAZ1B [OS=Homo sapiens]	0.538	0.271922849
Histone deacetylase complex subunit SAP18 [OS=Homo sapiens]	0.538	0.26138466
Myotubularin-related protein 9 [OS=Homo sapiens]	0.54	0.00433254
CCAAT/enhancer-binding protein zeta [OS=Homo sapiens]	0.544	0.270654764
Nucleolar protein 7 [OS=Homo sapiens]	0.544	0.533557539
Ribosomal RNA-processing protein 7 homolog A [OS=Homo sapiens]	0.546	0.223141891

Sideroflexin-1 [OS=Homo sapiens]	0.547	0.058213273
Nuclear envelope pore membrane protein POM 121 [OS=Homo sapiens]	0.547	0.231462341
Metaxin-2 [OS=Homo sapiens]	0.547	0.495996593
Protein RRP5 homolog [OS=Homo sapiens]	0.548	0.489637434
Mitochondrial import receptor subunit TOM6 homolog [OS=Homo sapiens]	0.548	0.254949396
Refilin-A [OS=Homo sapiens]	0.548	0.021896252
Transcription factor 20 [OS=Homo sapiens]	0.549	0.572435743
RNA exonuclease 5 [OS=Homo sapiens]	0.549	0.006748877
SUN domain-containing protein 1 [OS=Homo sapiens]	0.551	0.448763526
U3 small nucleolar RNA-associated protein 14 homolog A [OS=Homo sapiens]	0.551	0.279142092
Ribosome biogenesis protein NSA2 homolog [OS=Homo sapiens]	0.551	0.400331387
DNA replication complex GINS protein PSF1 [OS=Homo sapiens]	0.552	9.61428E-07
Breast cancer type 2 susceptibility protein [OS=Homo sapiens]	0.553	0.020254489
Cilia- and flagella-associated protein 69 [OS=Homo sapiens]	0.553	0.635957821
Nucleolar protein 56 [OS=Homo sapiens]	0.554	0.536980841
Ribosomal L1 domain-containing protein 1 [OS=Homo sapiens]	0.555	0.236654164
Heterogeneous nuclear ribonucleoprotein H3 [OS=Homo sapiens]	0.558	0.227633171
A-kinase anchor protein 2 [OS=Homo sapiens]	0.558	0.00954775
Cell division cycle-associated protein 2 [OS=Homo sapiens]	0.558	0.024439979
AT-rich interactive domain-containing protein 3A [OS=Homo sapiens]	0.558	0.207113774
Histone H1.3 [OS=Homo sapiens]	0.559	0.34456157
DNA dC->dU-editing enzyme APOBEC-3B [OS=Homo sapiens]	0.559	0.14141569
Zinc finger protein castor homolog 1 [OS=Homo sapiens]	0.561	0.514228792
Chromatin target of PRMT1 protein [OS=Homo sapiens]	0.563	0.486477261
Ribosome biogenesis protein BRX1 homolog [OS=Homo sapiens]	0.563	0.257746181
KRR1 small subunit processome component homolog [OS=Homo sapiens]	0.563	0.245119
Serine/arginine-rich splicing factor 10 [OS=Homo sapiens]	0.564	0.19674715
DNA replication complex GINS protein PSF3 [OS=Homo sapiens]	0.564	0.00029791
POU domain, class 2, transcription factor 1 [OS=Homo sapiens]	0.564	4.93316E-05
Zinc finger protein 512 [OS=Homo sapiens]	0.564	0.186803524
RNA-binding protein 15 [OS=Homo sapiens]	0.565	0.130782536
Prelamin-A/C [OS=Homo sapiens]	0.566	0.371274327
Zinc finger RNA-binding protein [OS=Homo sapiens]	0.566	0.158625002
U3 small nucleolar RNA-associated protein 18 homolog [OS=Homo sapiens]	0.568	0.371257987
Wilms tumor protein [OS=Homo sapiens]	0.568	0.108673289
Zinc finger protein 367 [OS=Homo sapiens]	0.568	0.202396582
Matrin-3 [OS=Homo sapiens]	0.569	0.34663564
PHD finger protein 14 [OS=Homo sapiens]	0.569	0.143807661
Nuclear pore complex protein Nup153 [OS=Homo sapiens]	0.57	0.078367176
Nucleolar complex protein 2 homolog [OS=Homo sapiens]	0.572	0.307335146
Centromere protein F [OS=Homo sapiens]	0.573	0.010203588
RNA-binding protein 14 [OS=Homo sapiens]	0.573	0.212439073
Germinal-center associated nuclear protein [OS=Homo sapiens]	0.574	0.403145452
PWWP domain-containing protein 2A [OS=Homo sapiens]	0.574	0.089103116
H/ACA ribonucleoprotein complex subunit DKC1 [OS=Homo sapiens]	0.575	0.025397386
Sulfate transporter [OS=Homo sapiens]	0.576	0.022195644
H/ACA ribonucleoprotein complex subunit 1 [OS=Homo sapiens]	0.576	0.569454046
RNA cytidine acetyltransferase [OS=Homo sapiens]	0.577	0.46991509
U3 small nucleolar RNA-associated protein 15 homolog [OS=Homo sapiens]	0.578	0.284159492
Mitochondrial import receptor subunit TOM20 homolog [OS=Homo sapiens]	0.578	0.363414854
Ribosome production factor 1 [OS=Homo sapiens]	0.578	0.667224523
Cohesin subunit SA-3 [OS=Homo sapiens]	0.585	9.64856E-06
Lamin-B2 [OS=Homo sapiens]	0.586	0.716449351
Sorting and assembly machinery component 50 homolog [OS=Homo sapiens]	0.586	0.63168211
E3 ubiquitin-protein ligase RBBP6 [OS=Homo sapiens]	0.587	0.072405382
RNA-binding protein 34 [OS=Homo sapiens]	0.587	0.173066958
Cadherin-1 [OS=Homo sapiens]	0.587	0.246594013
Testis-expressed protein 10 [OS=Homo sapiens]	0.588	0.504870577
Nuclear receptor coactivator 5 [OS=Homo sapiens]	0.588	0.390008124
Ankyrin repeat domain-containing protein 61 [OS=Homo sapiens]	0.589	0.852179859
Protein ELYS [OS=Homo sapiens]	0.59	0.578152069
Ribosomal biogenesis protein LAS1L [OS=Homo sapiens]	0.592	0.172145106
WD repeat-containing protein 43 [OS=Homo sapiens]	0.592	0.429331678
Pumilio homolog 3 [OS=Homo sapiens]	0.594	0.41012057
Probable ATP-dependent RNA helicase DDX52 [OS=Homo sapiens]	0.594	0.586780895
NK-tumor recognition protein [OS=Homo sapiens]	0.594	0.355570573
MICOS complex subunit MIC60 [OS=Homo sapiens]	0.595	0.72949075
Heterogeneous nuclear ribonucleoprotein U-like protein 2 [OS=Homo sapiens]	0.595	0.289879409
Spermatid perinuclear RNA-binding protein [OS=Homo sapiens]	0.595	0.181917339
Nucleoporin p54 [OS=Homo sapiens]	0.595	0.353972337
Deoxynucleotidyltransferase terminal-interacting protein 2 [OS=Homo sapiens]	0.595	0.143755306
E3 ISG15--protein ligase HERC5 [OS=Homo sapiens]	0.595	0.029526352
Homeobox protein Hox-B9 [OS=Homo sapiens]	0.595	0.022998501
Cyclin-dependent kinase 6 [OS=Homo sapiens]	0.596	2.0184E-06
Syntaxin-16 [OS=Homo sapiens]	0.596	0.373888235

WD repeat-containing protein 76 [OS=Homo sapiens]	0.596	0.010223669
Keratin, type II cytoskeletal 8 [OS=Homo sapiens]	0.597	0.278375054
Nucleolar protein 58 [OS=Homo sapiens]	0.597	0.301586764
Transcription factor AP-2-alpha [OS=Homo sapiens]	0.597	0.161346142
Suppressor of SWI4 1 homolog [OS=Homo sapiens]	0.598	0.377734228
U4/U6 small nuclear ribonucleoprotein Prp3 [OS=Homo sapiens]	0.598	0.073020524
Heterogeneous nuclear ribonucleoprotein M [OS=Homo sapiens]	0.599	0.296494312
Interferon-related developmental regulator 1 [OS=Homo sapiens]	0.599	0.234147598
Drebrin [OS=Homo sapiens]	0.6	0.566157633
Epsin-2 [OS=Homo sapiens]	0.6	0.000105451
Probable RNA-binding protein 19 [OS=Homo sapiens]	0.6	0.445849419
Nucleolar protein 6 [OS=Homo sapiens]	0.601	0.392914454
Caveolae-associated protein 3 [OS=Homo sapiens]	0.601	0.120691657
Cohesin subunit SA-1 [OS=Homo sapiens]	0.602	0.076217881
Mediator of DNA damage checkpoint protein 1 [OS=Homo sapiens]	0.603	0.045507962
Protein TASOR 2 [OS=Homo sapiens]	0.603	0.158524994
Zinc finger protein 106 [OS=Homo sapiens]	0.603	0.118134717
ATP-dependent RNA helicase DDX51 [OS=Homo sapiens]	0.605	0.183963272
H/ACA ribonucleoprotein complex subunit 2 [OS=Homo sapiens]	0.605	0.017391095
Pre-mRNA 3'-end-processing factor FIP1 [OS=Homo sapiens]	0.606	0.222925084
Neuroguidin [OS=Homo sapiens]	0.607	0.376168923
Probable ribosome biogenesis protein RLP24 [OS=Homo sapiens]	0.608	0.13914453
RNA-binding protein 45 [OS=Homo sapiens]	0.608	0.102898818
Receptor-transporting protein 3 [OS=Homo sapiens]	0.608	0.535768155
pre-rRNA 2'-O-ribose RNA methyltransferase FTSJ3 [OS=Homo sapiens]	0.609	0.305872425
Pre-mRNA-splicing regulator WTAP [OS=Homo sapiens]	0.609	0.323493791
Sterile alpha motif domain-containing protein 1 [OS=Homo sapiens]	0.61	0.212652568
Mitochondrial import inner membrane translocase subunit Tim17-A [OS=Homo s	0.61	0.180800253
Telomere-associated protein RIF1 [OS=Homo sapiens]	0.611	0.114379991
Heterogeneous nuclear ribonucleoprotein A3 [OS=Homo sapiens]	0.611	0.359166456
THO complex subunit 5 homolog [OS=Homo sapiens]	0.612	0.322806072
N-acetyltransferase ESCO2 [OS=Homo sapiens]	0.612	0.520950315
Probable global transcription activator SNF2L1 [OS=Homo sapiens]	0.613	0.13608606
Heterogeneous nuclear ribonucleoproteins A2/B1 [OS=Homo sapiens]	0.614	0.263677048
THO complex subunit 2 [OS=Homo sapiens]	0.614	0.420940242
YTH domain-containing protein 1 [OS=Homo sapiens]	0.614	0.239601941
THO complex subunit 1 [OS=Homo sapiens]	0.615	0.375167444
WD repeat-containing protein 46 [OS=Homo sapiens]	0.615	0.404526656
Splicing factor 45 [OS=Homo sapiens]	0.616	0.003975755
INO80 complex subunit E [OS=Homo sapiens]	0.616	0.325308068
Keratin, type I cytoskeletal 18 [OS=Homo sapiens]	0.617	0.378311128
Polynucleotide 5'-hydroxyl-kinase NOL9 [OS=Homo sapiens]	0.617	0.079627691
Ribosomal RNA processing protein 36 homolog [OS=Homo sapiens]	0.617	0.017002996
Nucleolar protein 11 [OS=Homo sapiens]	0.618	0.501674623
Nucleolar protein 10 [OS=Homo sapiens]	0.618	0.415717625
Nucleoprotein TPR [OS=Homo sapiens]	0.62	0.342454821
Zinc finger CCCH domain-containing protein 14 [OS=Homo sapiens]	0.62	0.200703678
Zinc finger protein 638 [OS=Homo sapiens]	0.622	0.207456506
RNA exonuclease 4 [OS=Homo sapiens]	0.622	0.221610663
Zinc finger CCCH domain-containing protein 13 [OS=Homo sapiens]	0.623	0.215098963
Tyrosine-protein kinase Lyn [OS=Homo sapiens]	0.623	0.274309697
RNA 3'-terminal phosphate cyclase-like protein [OS=Homo sapiens]	0.624	0.284139186
Mitochondrial import receptor subunit TOM5 homolog [OS=Homo sapiens]	0.624	0.497433749
Centrosomal protein of 89 kDa [OS=Homo sapiens]	0.624	0.180996173
Little elongation complex subunit 1 [OS=Homo sapiens]	0.624	0.182228798
Nucleolar complex protein 4 homolog [OS=Homo sapiens]	0.625	0.626685614
Coiled-coil domain-containing protein 137 [OS=Homo sapiens]	0.625	0.425482667
Hepatocyte nuclear factor 4-alpha [OS=Homo sapiens]	0.625	0.554266417
M-phase phosphoprotein 8 [OS=Homo sapiens]	0.625	0.290419816
RB-associated KRAB zinc finger protein [OS=Homo sapiens]	0.626	0.042452793
Probable ATP-dependent RNA helicase DDX10 [OS=Homo sapiens]	0.627	0.118539067
Lymphokine-activated killer T-cell-originated protein kinase [OS=Homo sapiens]	0.628	2.34409E-06
Mitochondrial import receptor subunit TOM22 homolog [OS=Homo sapiens]	0.629	0.31823488
Surfeit locus protein 6 [OS=Homo sapiens]	0.63	0.336366135
Structural maintenance of chromosomes protein 5 [OS=Homo sapiens]	0.631	0.194862073
Neurabin-2 [OS=Homo sapiens]	0.631	0.625824689
DNA replication complex GINS protein PSF2 [OS=Homo sapiens]	0.631	1.17374E-07
Ribonucleoprotein PTB-binding 1 [OS=Homo sapiens]	0.632	4.24073E-08
Mothers against decapentaplegic homolog 5 [OS=Homo sapiens]	0.632	1.45774E-05
Nuclear cap-binding protein subunit 3 [OS=Homo sapiens]	0.632	0.243624011
Nucleolar pre-ribosomal-associated protein 1 [OS=Homo sapiens]	0.632	0.091631921
Histone H2A type 1 [OS=Homo sapiens]	0.632	0.557168688
Transcription initiation factor TFIID subunit 1-like [OS=Homo sapiens]	0.632	0.206014052
Unconventional myosin-IXb [OS=Homo sapiens]	0.633	0.004766803
Keratin, type I cytoskeletal 9 [OS=Homo sapiens]	0.633	0.999965907

THO complex subunit 4 [OS=Homo sapiens]	0.633	0.196598061
Splicing factor, arginine/serine-rich 19 [OS=Homo sapiens]	0.633	0.636957444
Sentrin-specific protease 3 [OS=Homo sapiens]	0.633	0.549212907
Constitutive coactivator of PPAR-gamma-like protein 2 [OS=Homo sapiens]	0.633	0.001470204
Lamina-associated polypeptide 2, isoforms beta/gamma [OS=Homo sapiens]	0.635	0.178866618
Transcriptional coactivator YAP1 [OS=Homo sapiens]	0.635	3.68278E-06
Aminopeptidase N [OS=Homo sapiens]	0.636	0.040450133
Heterochromatin protein 1-binding protein 3 [OS=Homo sapiens]	0.636	0.378502207
Ribosome biogenesis protein BMS1 homolog [OS=Homo sapiens]	0.636	0.262117951
Pre-mRNA-splicing factor RBM22 [OS=Homo sapiens]	0.636	0.095902946
Zinc finger protein 280C [OS=Homo sapiens]	0.636	0.809400945
Sarcoma antigen 1 [OS=Homo sapiens]	0.636	0.08153119
Histone H1.5 [OS=Homo sapiens]	0.637	0.506310613
Zinc finger protein 22 [OS=Homo sapiens]	0.637	0.241469808
Protein KRI1 homolog [OS=Homo sapiens]	0.639	0.137703802
Caspase activity and apoptosis inhibitor 1 [OS=Homo sapiens]	0.639	0.288678784
Homeobox protein Hox-C9 [OS=Homo sapiens]	0.639	0.094864379
CD109 antigen [OS=Homo sapiens]	0.64	0.016585762
Glycerol kinase [OS=Homo sapiens]	0.64	0.294356159
Protein MMS22-like [OS=Homo sapiens]	0.64	0.086949665
Aurora kinase B [OS=Homo sapiens]	0.641	0.054783859
eIF-2-alpha kinase activator GCN1 [OS=Homo sapiens]	0.642	8.32573E-07
Heterogeneous nuclear ribonucleoprotein R [OS=Homo sapiens]	0.642	0.363645679
ATP-dependent RNA helicase DDX54 [OS=Homo sapiens]	0.642	0.297189758
CD59 glycoprotein [OS=Homo sapiens]	0.642	0.335610065
Nuclear mitotic apparatus protein 1 [OS=Homo sapiens]	0.643	0.30783541
DNA topoisomerase 2-alpha [OS=Homo sapiens]	0.643	0.033152046
Nesprin-2 [OS=Homo sapiens]	0.643	0.167332792
Nucleoporin NUP42 [OS=Homo sapiens]	0.643	0.030774893
ATP-dependent RNA helicase A [OS=Homo sapiens]	0.644	0.235574665
WW domain-binding protein 11 [OS=Homo sapiens]	0.644	0.00760539
Coilin [OS=Homo sapiens]	0.644	0.423319355
Sphingomyelin phosphodiesterase 4 [OS=Homo sapiens]	0.644	0.53792958
RNA-binding protein with serine-rich domain 1 [OS=Homo sapiens]	0.645	0.326883305
Kinesin-like protein KIF22 [OS=Homo sapiens]	0.645	0.018031598
Corepressor interacting with RBPJ 1 [OS=Homo sapiens]	0.645	0.152339
Cytospin-B [OS=Homo sapiens]	0.646	0.059633597
Transcriptional enhancer factor TEF-3 [OS=Homo sapiens]	0.646	0.060799319
ADP-ribosylation factor-like protein 2-binding protein [OS=Homo sapiens]	0.646	7.81456E-05
Transcription factor A, mitochondrial [OS=Homo sapiens]	0.647	0.700088668
SAP domain-containing ribonucleoprotein [OS=Homo sapiens]	0.647	0.221816271
DDB1- and CUL4-associated factor 13 [OS=Homo sapiens]	0.647	0.168201825
Tumor suppressor ARF [OS=Homo sapiens]	0.647	0.000275828
Pericentrin [OS=Homo sapiens]	0.648	0.000136052
TOM1-like protein 2 [OS=Homo sapiens]	0.648	0.003867192
Nucleolar RNA helicase 2 [OS=Homo sapiens]	0.649	0.248320028
Kinesin-like protein KIF20A [OS=Homo sapiens]	0.649	0.021038041
WD repeat-containing protein 18 [OS=Homo sapiens]	0.649	0.686518005
Glycolipid transfer protein [OS=Homo sapiens]	0.649	0.000605412
MMS19 nucleotide excision repair protein homolog [OS=Homo sapiens]	0.651	0.000314451
Cleavage and polyadenylation specificity factor subunit 4 [OS=Homo sapiens]	0.651	0.430872811
U2 snRNP-associated SURP motif-containing protein [OS=Homo sapiens]	0.652	0.001135314
Stearoyl-CoA desaturase 5 [OS=Homo sapiens]	0.652	0.050421118
ATPase family AAA domain-containing protein 3A [OS=Homo sapiens]	0.653	0.684649686
Protein TASOR [OS=Homo sapiens]	0.653	0.308389689
Transformer-2 protein homolog alpha [OS=Homo sapiens]	0.653	0.598040693
Abnormal spindle-like microcephaly-associated protein [OS=Homo sapiens]	0.654	0.071685622
SWI/SNF-related matrix-associated actin-dependent regulator of chromatin sub	0.655	0.351190198
Probable ATP-dependent RNA helicase DDX41 [OS=Homo sapiens]	0.655	0.000285025
Bromodomain-containing protein 1 [OS=Homo sapiens]	0.655	0.625647055
Lysine-specific demethylase 5A [OS=Homo sapiens]	0.655	0.687153743
Mitochondrial import inner membrane translocase subunit Tim23 [OS=Homo sa	0.656	0.488493564
Ribosomal RNA processing protein 1 homolog B [OS=Homo sapiens]	0.657	0.268105307
Cytochrome c oxidase subunit 7A2, mitochondrial [OS=Homo sapiens]	0.657	0.582945493
Syntaxin-3 [OS=Homo sapiens]	0.657	0.012795531
mRNA export factor GLE1 [OS=Homo sapiens]	0.657	0.560886043
Polycomb complex protein BMI-1 [OS=Homo sapiens]	0.657	0.206694706
Post-GPI attachment to proteins factor 4 [OS=Homo sapiens]	0.658	0.999947978
Histone H1.4 [OS=Homo sapiens]	0.659	0.638725388
Epiplakin [OS=Homo sapiens]	0.659	0.040536135
Caveolin-1 [OS=Homo sapiens]	0.659	0.185182429
Mitochondrial import inner membrane translocase subunit Tim29 [OS=Homo sa	0.659	0.468071436
U3 small nucleolar ribonucleoprotein protein IMP3 [OS=Homo sapiens]	0.659	0.729763857
Cell division cycle 5-like protein [OS=Homo sapiens]	0.66	0.118109619
SNW domain-containing protein 1 [OS=Homo sapiens]	0.66	0.074520762

NADH dehydrogenase [ubiquinone] 1 beta subcomplex subunit 7 [OS=Homo sap	0.66	0.595795889
SIN3-HDAC complex-associated factor [OS=Homo sapiens]	0.66	0.003514363
Protein ECT2 [OS=Homo sapiens]	0.661	0.007312351
Transcription factor Sp3 [OS=Homo sapiens]	0.662	0.088424727
SUZ domain-containing protein 1 [OS=Homo sapiens]	0.662	3.99131E-06
MutS protein homolog 4 [OS=Homo sapiens]	0.662	0.490541934
Lamina-associated polypeptide 2, isoform alpha [OS=Homo sapiens]	0.663	0.033251622
E3 SUMO-protein ligase NSE2 [OS=Homo sapiens]	0.663	0.421572328
OTU domain-containing protein 4 [OS=Homo sapiens]	0.664	1.53105E-05
rRNA-processing protein FCF1 homolog [OS=Homo sapiens]	0.665	0.630034556
Ubiquitin carboxyl-terminal hydrolase 3 [OS=Homo sapiens]	0.665	0.873718563
PH-interacting protein [OS=Homo sapiens]	0.665	0.936570702
Transcription factor SOX-11 [OS=Homo sapiens]	0.665	0.11530013
Zinc finger protein Rlf [OS=Homo sapiens]	0.665	0.482139963
Thyroid hormone receptor-associated protein 3 [OS=Homo sapiens]	0.666	0.139664522
Heterogeneous nuclear ribonucleoprotein A/B [OS=Homo sapiens]	0.666	0.127731408
MICOS complex subunit MIC26 [OS=Homo sapiens]	0.666	0.641270664
A-kinase anchor protein 17A [OS=Homo sapiens]	0.666	0.166259965
Cytosolic iron-sulfur assembly component 2B [OS=Homo sapiens]	0.666	0.000105313
Nucleoporin p58/p45 [OS=Homo sapiens]	0.666	0.700735364
MICOS complex subunit MIC19 [OS=Homo sapiens]	0.667	0.78858903
Serine protease FAM111A [OS=Homo sapiens]	0.667	0.181981296
Zinc finger protein 644 [OS=Homo sapiens]	0.667	0.53843543
Ninein [OS=Homo sapiens]	0.668	0.64467183
Probable 28S rRNA (cytosine(4447)-C(5))-methyltransferase [OS=Homo sapiens	0.669	0.413506133
Peptidyl-prolyl cis-trans isomerase D [OS=Homo sapiens]	0.669	2.70925E-06
pre-mRNA 3' end processing protein WDR33 [OS=Homo sapiens]	0.669	0.314999321
Calcium-binding mitochondrial carrier protein SCaMC-1 [OS=Homo sapiens]	0.67	0.37311276
Centrosomal protein of 131 kDa [OS=Homo sapiens]	0.67	0.025022383
Zinc finger protein RFP [OS=Homo sapiens]	0.67	0.09330421
snRNA-activating protein complex subunit 4 [OS=Homo sapiens]	0.67	0.498458083
High mobility group protein 20A [OS=Homo sapiens]	0.671	0.25894474
Methyl-CpG-binding protein 2 [OS=Homo sapiens]	0.672	0.873431
RNA-binding protein 5 [OS=Homo sapiens]	0.672	0.19147705
Centrosomal protein of 68 kDa [OS=Homo sapiens]	0.672	0.052504008
CCR4-NOT transcription complex subunit 8 [OS=Homo sapiens]	0.672	0.128700553
Targeting protein for Xklp2 [OS=Homo sapiens]	0.673	0.002063363
Ribonucleoside-diphosphate reductase subunit M2 [OS=Homo sapiens]	0.673	8.60414E-05
Cytochrome b-c1 complex subunit 7 [OS=Homo sapiens]	0.673	0.545096002
Zinc finger matrin-type protein 2 [OS=Homo sapiens]	0.673	0.010858089
RNA-binding protein 12B [OS=Homo sapiens]	0.673	0.417537926
Melanotransferrin [OS=Homo sapiens]	0.674	0.109292635
Dual specificity protein phosphatase 9 [OS=Homo sapiens]	0.674	0.5539477
Protein HIRA [OS=Homo sapiens]	0.674	0.568538613
Zinc finger CCCH domain-containing protein 18 [OS=Homo sapiens]	0.675	0.187408618
UL16-binding protein 1 [OS=Homo sapiens]	0.675	0.338088999
Bifunctional methylenetetrahydrofolate dehydrogenase/cyclohydrolase, mitoch	0.676	5.36774E-06
Histone H1.0 [OS=Homo sapiens]	0.676	0.760467639
Testis-specific Y-encoded-like protein 1 [OS=Homo sapiens]	0.676	0.001441202
AKT-interacting protein [OS=Homo sapiens]	0.676	0.019666381
Ras-related protein R-Ras2 [OS=Homo sapiens]	0.677	0.000896994
MKI67 FHA domain-interacting nucleolar phosphoprotein [OS=Homo sapiens]	0.677	0.140883612
DNA repair protein complementing XP-C cells [OS=Homo sapiens]	0.677	0.49098618
U5 small nuclear ribonucleoprotein 40 kDa protein [OS=Homo sapiens]	0.678	0.154560734
Lysine-rich nucleolar protein 1 [OS=Homo sapiens]	0.678	0.252739558
Transformer-2 protein homolog beta [OS=Homo sapiens]	0.68	0.483141603
Mitochondrial 2-oxoglutarate/malate carrier protein [OS=Homo sapiens]	0.681	0.469219656
Cytochrome c oxidase subunit 2 [OS=Homo sapiens]	0.681	0.462340001
Nuclear pore complex protein Nup205 [OS=Homo sapiens]	0.682	0.389687195
Proline-, glutamic acid- and leucine-rich protein 1 [OS=Homo sapiens]	0.682	0.347502616
Probable ATP-dependent RNA helicase DDX47 [OS=Homo sapiens]	0.682	0.036675544
60S ribosome subunit biogenesis protein NIP7 homolog [OS=Homo sapiens]	0.682	0.426985322
AP-4 complex accessory subunit RUSC2 [OS=Homo sapiens]	0.683	0.155290274
Mitochondrial import receptor subunit TOM40 homolog [OS=Homo sapiens]	0.684	0.484456997
Ras-related protein Rap-2b [OS=Homo sapiens]	0.684	0.47738802
ATP-dependent RNA helicase DDX39A [OS=Homo sapiens]	0.685	0.007496708
Polymerase delta-interacting protein 3 [OS=Homo sapiens]	0.685	0.199444722
Transcriptional regulator ATRX [OS=Homo sapiens]	0.686	0.148588484
Kinesin-like protein KIF20B [OS=Homo sapiens]	0.686	0.110232156
Probable rRNA-processing protein EBP2 [OS=Homo sapiens]	0.687	0.464617689
Protein virilizer homolog [OS=Homo sapiens]	0.687	0.379545733
Cytochrome b-c1 complex subunit 2, mitochondrial [OS=Homo sapiens]	0.688	0.330940861
Supervillin [OS=Homo sapiens]	0.688	0.515660799
Retinoic acid receptor RXR-alpha [OS=Homo sapiens]	0.688	0.013899007
PHD finger protein 3 [OS=Homo sapiens]	0.689	0.293500831

DNA-directed RNA polymerase I subunit RPA49 [OS=Homo sapiens]	0.69	0.052720377
Homeobox protein cut-like 1 [OS=Homo sapiens]	0.691	0.279525655
Heterogeneous nuclear ribonucleoprotein D-like [OS=Homo sapiens]	0.692	0.210745335
Protein arginine N-methyltransferase 3 [OS=Homo sapiens]	0.692	0.019172143
Chromatin assembly factor 1 subunit B [OS=Homo sapiens]	0.692	9.03383E-05
SH3 domain-containing kinase-binding protein 1 [OS=Homo sapiens]	0.692	3.72347E-05
Pleiotropic regulator 1 [OS=Homo sapiens]	0.692	0.170124388
CKLF-like MARVEL transmembrane domain-containing protein 6 [OS=Homo sap	0.692	0.04793753
Interleukin enhancer-binding factor 2 [OS=Homo sapiens]	0.693	0.404211809
SOSS complex subunit B1 [OS=Homo sapiens]	0.693	0.092055989
Stomatin-like protein 2, mitochondrial [OS=Homo sapiens]	0.694	0.739144084
E3 SUMO-protein ligase RanBP2 [OS=Homo sapiens]	0.695	0.518438127
Cell growth-regulating nucleolar protein [OS=Homo sapiens]	0.695	0.151293474
Ubiquitin carboxyl-terminal hydrolase CYLD [OS=Homo sapiens]	0.695	0.00196384
RNA-binding protein 10 [OS=Homo sapiens]	0.696	0.318506382
ATP-binding cassette sub-family D member 3 [OS=Homo sapiens]	0.697	0.046584429
A-kinase anchor protein 8 [OS=Homo sapiens]	0.697	0.289693884
1-phosphatidylinositol 4,5-bisphosphate phosphodiesterase delta-3 [OS=Homo	0.697	0.341203536
RNA-binding protein 28 [OS=Homo sapiens]	0.698	0.162426602
Voltage-dependent anion-selective channel protein 3 [OS=Homo sapiens]	0.698	0.683272295
Outer mitochondrial transmembrane helix translocase [OS=Homo sapiens]	0.698	0.07518915
Cystine/glutamate transporter [OS=Homo sapiens]	0.698	0.001988946
Protein downstream neighbor of Son [OS=Homo sapiens]	0.698	0.029192611
Neuroblast differentiation-associated protein AHNAK [OS=Homo sapiens]	0.699	1.34795E-09
Nucleoporin NUP35 [OS=Homo sapiens]	0.699	0.230267155
Mitochondrial import inner membrane translocase subunit Tim21 [OS=Homo sa	0.699	0.374868224
Transcriptional enhancer factor TEF-1 [OS=Homo sapiens]	0.699	0.033211659
Heterogeneous nuclear ribonucleoprotein A0 [OS=Homo sapiens]	0.7	0.167941314
Pre-mRNA-splicing factor SPF27 [OS=Homo sapiens]	0.7	0.112629691
Borealin [OS=Homo sapiens]	0.7	0.131106401
Unhealthy ribosome biogenesis protein 2 homolog [OS=Homo sapiens]	0.7	0.141710832
Polyglutamine-binding protein 1 [OS=Homo sapiens]	0.701	0.002352446
Gem-associated protein 5 [OS=Homo sapiens]	0.702	7.0112E-05
U4/U6 small nuclear ribonucleoprotein Prp4 [OS=Homo sapiens]	0.702	0.012354634
E3 ubiquitin-protein ligase NRDP1 [OS=Homo sapiens]	0.702	0.002509611
GDNF family receptor alpha-3 [OS=Homo sapiens]	0.702	0.487362608
Structural maintenance of chromosomes flexible hinge domain-containing prote	0.703	1.21467E-08
Phosphoserine aminotransferase [OS=Homo sapiens]	0.703	0.000390041
Nuclear pore membrane glycoprotein 210 [OS=Homo sapiens]	0.704	0.462912391
CDK5 regulatory subunit-associated protein 2 [OS=Homo sapiens]	0.705	0.000649668
MAX gene-associated protein [OS=Homo sapiens]	0.706	0.394268013
Pre-mRNA-splicing factor CWC22 homolog [OS=Homo sapiens]	0.706	0.032406819
DNA replication complex GINS protein SLD5 [OS=Homo sapiens]	0.706	0.000118802
Transcription factor JunB [OS=Homo sapiens]	0.706	0.001261026
Pre-mRNA-processing factor 40 homolog A [OS=Homo sapiens]	0.707	0.194717589
Stomatin [OS=Homo sapiens]	0.707	0.25224609
Ribosomal RNA processing protein 1 homolog A [OS=Homo sapiens]	0.707	0.061446095
DNA repair protein SWI5 homolog [OS=Homo sapiens]	0.707	0.165388825
Zinc finger and BTB domain-containing protein 7A [OS=Homo sapiens]	0.708	0.015646904
ER degradation-enhancing alpha-mannosidase-like protein 3 [OS=Homo sapien	0.708	0.002525763
UPF0461 protein C5orf24 [OS=Homo sapiens]	0.708	0.800707109
Probable ATP-dependent RNA helicase DDX56 [OS=Homo sapiens]	0.709	0.532063538
Prohibitin 1 [OS=Homo sapiens]	0.71	0.502678004
Serine/arginine-rich splicing factor 7 [OS=Homo sapiens]	0.71	0.211032317
Transmembrane protein 209 [OS=Homo sapiens]	0.71	0.285600539
ELAV-like protein 1 [OS=Homo sapiens]	0.711	0.248970756
ATP-dependent RNA helicase DDX50 [OS=Homo sapiens]	0.711	0.012326906
NADH dehydrogenase [ubiquinone] 1 beta subcomplex subunit 8, mitochondrial	0.711	0.623048021
Claspin [OS=Homo sapiens]	0.711	3.03201E-05
Breast cancer type 1 susceptibility protein [OS=Homo sapiens]	0.711	0.000416599
High affinity cationic amino acid transporter 1 [OS=Homo sapiens]	0.711	0.351440943
ATPase family AAA domain-containing protein 3B [OS=Homo sapiens]	0.712	0.734860694
Probable ATP-dependent RNA helicase DDX31 [OS=Homo sapiens]	0.712	0.26912183
Ubiquitin-like-conjugating enzyme ATG3 [OS=Homo sapiens]	0.712	6.31559E-05
Protein C-ets-1 [OS=Homo sapiens]	0.712	0.008985206
cGMP-inhibited 3',5'-cyclic phosphodiesterase 3B [OS=Homo sapiens]	0.712	0.103142883
RNA binding protein fox-1 homolog 1 [OS=Homo sapiens]	0.713	0.017427354
Circadian clock protein PASD1 [OS=Homo sapiens]	0.714	0.239379282
Cytochrome c oxidase assembly protein COX15 homolog [OS=Homo sapiens]	0.714	0.410493625
Keratin, type II cytoskeletal 2 epidermal [OS=Homo sapiens]	0.715	0.999793883
Mitochondrial dicarboxylate carrier [OS=Homo sapiens]	0.715	0.472713296
Progesterone-induced-blocking factor 1 [OS=Homo sapiens]	0.715	0.148830941
Ribosome biogenesis protein BOP1 [OS=Homo sapiens]	0.716	0.091436945
Nuclear speckle splicing regulatory protein 1 [OS=Homo sapiens]	0.716	3.48629E-05
Acyl-CoA 6-desaturase [OS=Homo sapiens]	0.716	9.83824E-05

Cytochrome P450 1B1 [OS=Homo sapiens]	0.716	0.420715872
Leucine-rich repeat-containing protein 15 [OS=Homo sapiens]	0.717	0.618959971
Probable ATP-dependent RNA helicase DDX27 [OS=Homo sapiens]	0.718	0.543433771
Synaptojanin-2-binding protein [OS=Homo sapiens]	0.718	0.51790302
Cleavage and polyadenylation specificity factor subunit 2 [OS=Homo sapiens]	0.718	0.076610739
Lymphoid-specific helicase [OS=Homo sapiens]	0.719	0.000318416
Calcium homeostasis endoplasmic reticulum protein [OS=Homo sapiens]	0.719	0.009680222
Cleavage and polyadenylation specificity factor subunit 3 [OS=Homo sapiens]	0.719	0.027510532
U3 small nucleolar RNA-associated protein 6 homolog [OS=Homo sapiens]	0.719	0.40373228
Mitochondrial import inner membrane translocase subunit Tim9 [OS=Homo sap	0.719	0.638197951
U3 small nucleolar RNA-associated protein 4 homolog [OS=Homo sapiens]	0.719	0.360656583
60S ribosomal protein L7-like 1 [OS=Homo sapiens]	0.719	0.300806039
Zinc finger protein 703 [OS=Homo sapiens]	0.719	0.013236326
Interferon-related developmental regulator 2 [OS=Homo sapiens]	0.719	0.498922976
Cleavage stimulation factor subunit 3 [OS=Homo sapiens]	0.72	0.265405448
Kinetochore protein Nuf2 [OS=Homo sapiens]	0.72	0.000247378
Neutral amino acid transporter B(0) [OS=Homo sapiens]	0.72	0.502449033
Fidgetin-like protein 1 [OS=Homo sapiens]	0.72	0.029340497
Dixin [OS=Homo sapiens]	0.72	0.96209112
Sentrin-specific protease 5 [OS=Homo sapiens]	0.72	0.240490703
Syndecan-1 [OS=Homo sapiens]	0.721	0.017232511
MYND-type zinc finger-containing chromatin reader ZMYND8 [OS=Homo sapiens	0.722	0.279309019
ATP-binding cassette sub-family B member 10, mitochondrial [OS=Homo sapier	0.722	0.457417172
Spindle and kinetochore-associated protein 1 [OS=Homo sapiens]	0.722	0.02977466
Nuclear RNA export factor 1 [OS=Homo sapiens]	0.724	0.302049235
Nuclear factor of activated T-cells 5 [OS=Homo sapiens]	0.724	0.816572536
NADH dehydrogenase [ubiquinone] 1 alpha subcomplex subunit 13 [OS=Homo s	0.724	0.689318921
SOSS complex subunit C [OS=Homo sapiens]	0.724	0.09329755
Mitochondrial import inner membrane translocase subunit Tim10 B [OS=Homo s	0.724	0.783716191
ESF1 homolog [OS=Homo sapiens]	0.724	0.681174171
UAP56-interacting factor [OS=Homo sapiens]	0.724	0.237895288
RRP15-like protein [OS=Homo sapiens]	0.725	0.520961423
Putative E3 ubiquitin-protein ligase UBR7 [OS=Homo sapiens]	0.725	0.00064024
Msx2-interacting protein [OS=Homo sapiens]	0.725	0.220449398
Nuclear valosin-containing protein-like [OS=Homo sapiens]	0.726	0.004957067
DNA-directed RNA polymerase I subunit RPA34 [OS=Homo sapiens]	0.727	0.127600571
Syndecan-4 [OS=Homo sapiens]	0.727	0.000747099
Serine/arginine-rich splicing factor 9 [OS=Homo sapiens]	0.727	0.643718585
Threonylcarbamoyladenosine tRNA methylthiotransferase [OS=Homo sapiens]	0.727	0.000535107
Origin recognition complex subunit 6 [OS=Homo sapiens]	0.727	0.175170759
NADH dehydrogenase [ubiquinone] 1 alpha subcomplex subunit 8 [OS=Homo sa	0.727	0.879541378
Angiomotin-like protein 2 [OS=Homo sapiens]	0.727	0.705728147
Coiled-coil domain-containing protein 86 [OS=Homo sapiens]	0.728	0.244888923
Pyruvate dehydrogenase protein X component, mitochondrial [OS=Homo sapier	0.728	0.000826998
Thioredoxin-like protein 4A [OS=Homo sapiens]	0.728	0.58862095
Protein Red [OS=Homo sapiens]	0.729	0.071327405
Nuclear pore complex protein Nup160 [OS=Homo sapiens]	0.729	0.627560869
60S ribosomal export protein NMD3 [OS=Homo sapiens]	0.729	5.69105E-05
NADH dehydrogenase [ubiquinone] 1 beta subcomplex subunit 9 [OS=Homo sap	0.729	0.801419213
Histone-lysine N-methyltransferase EHMT1 [OS=Homo sapiens]	0.729	0.264807039
Spliceosome-associated protein CWC27 homolog [OS=Homo sapiens]	0.729	1.10279E-06
YLP motif-containing protein 1 [OS=Homo sapiens]	0.73	0.580360946
Microfibrillar-associated protein 1 [OS=Homo sapiens]	0.73	0.635366754
Zinc finger protein 217 [OS=Homo sapiens]	0.73	0.000138515
Polyhomeotic-like protein 2 [OS=Homo sapiens]	0.73	0.025061465
Thioredoxin reductase 1, cytoplasmic [OS=Homo sapiens]	0.732	0.000976128
Bcl-2-associated transcription factor 1 [OS=Homo sapiens]	0.732	0.57218785
Nuclear pore glycoprotein p62 [OS=Homo sapiens]	0.732	0.206847623
Ribonuclease P protein subunit p14 [OS=Homo sapiens]	0.732	0.297002328
DNA topoisomerase 3-alpha [OS=Homo sapiens]	0.732	0.009725324
Transmembrane protein 223 [OS=Homo sapiens]	0.732	0.890122638
Structural maintenance of chromosomes protein 1A [OS=Homo sapiens]	0.733	0.031446302
WD repeat-containing protein 36 [OS=Homo sapiens]	0.733	0.631878411
Cell division cycle and apoptosis regulator protein 1 [OS=Homo sapiens]	0.733	0.144564929
Enhancer of polycomb homolog 2 [OS=Homo sapiens]	0.733	0.500477143
Profilin-3 [OS=Homo sapiens]	0.733	0.2025656
Protein POLR1D, isoform 2 [OS=Homo sapiens]	0.733	0.053324402
NAD(P) transhydrogenase, mitochondrial [OS=Homo sapiens]	0.734	0.539783994
Cytotoxic granule associated RNA binding protein TIA1 [OS=Homo sapiens]	0.734	0.004143677
NADH dehydrogenase [ubiquinone] 1 subunit C2 [OS=Homo sapiens]	0.734	0.847451208
Spindle and kinetochore-associated protein 3 [OS=Homo sapiens]	0.734	0.235398175
Dual specificity protein kinase CLK1 [OS=Homo sapiens]	0.734	0.402008705
Cancer/testis antigen family 45 member A8 [OS=Homo sapiens]	0.735	2.12942E-05
Periodic tryptophan protein 2 homolog [OS=Homo sapiens]	0.735	0.865132106
Vacuolar protein sorting-associated protein 26B [OS=Homo sapiens]	0.735	0.000869625

Zinc finger protein 280D [OS=Homo sapiens]	0.735	0.288728102
Pre-mRNA-processing factor 6 [OS=Homo sapiens]	0.736	0.237285233
NADH dehydrogenase [ubiquinone] 1 alpha subcomplex subunit 12 [OS=Homo s	0.736	0.714351295
Zinc finger protein 654 [OS=Homo sapiens]	0.736	0.84914981
Unconventional myosin-VIIb [OS=Homo sapiens]	0.736	0.809704058
Prohibitin-2 [OS=Homo sapiens]	0.737	0.63072828
Transcriptional repressor CTCF [OS=Homo sapiens]	0.737	0.347251319
Adenylate kinase isoenzyme 6 [OS=Homo sapiens]	0.738	5.57553E-06
Uncharacterized protein KIAA1671 [OS=Homo sapiens]	0.738	0.120657886
Mitochondrial nicotinamide adenine dinucleotide transporter SLC25A52 [OS=Hc	0.738	0.388684479
E3 ubiquitin-protein ligase UHRF1 [OS=Homo sapiens]	0.739	0.001039163
5'-nucleotidase [OS=Homo sapiens]	0.739	0.73601519
Electrogenic aspartate/glutamate antiporter SLC25A12, mitochondrial [OS=Hon	0.739	0.840424977
Cytochrome c oxidase subunit 6C [OS=Homo sapiens]	0.739	0.667989076
U4/U6.U5 tri-snRNP-associated protein 1 [OS=Homo sapiens]	0.74	0.204860571
U3 small nucleolar RNA-interacting protein 2 [OS=Homo sapiens]	0.74	0.32292996
RAC-beta serine/threonine-protein kinase [OS=Homo sapiens]	0.74	0.000921011
Microsomal glutathione S-transferase 3 [OS=Homo sapiens]	0.74	0.741223932
Protein WWC2 [OS=Homo sapiens]	0.74	0.390812081
Centromere protein J [OS=Homo sapiens]	0.74	0.238490387
Nuclear pore complex protein Nup133 [OS=Homo sapiens]	0.741	0.809940913
Mitochondrial calcium uniporter regulator 1 [OS=Homo sapiens]	0.741	0.902034952
Rho guanine nucleotide exchange factor 26 [OS=Homo sapiens]	0.741	0.149193948
GTP-binding protein 4 [OS=Homo sapiens]	0.742	0.265707245
Double-strand-break repair protein rad21 homolog [OS=Homo sapiens]	0.742	0.072393119
1-acyl-sn-glycerol-3-phosphate acyltransferase epsilon [OS=Homo sapiens]	0.742	0.576516605
Tricarboxylate transport protein, mitochondrial [OS=Homo sapiens]	0.742	0.854963723
NADH dehydrogenase [ubiquinone] iron-sulfur protein 4, mitochondrial [OS=Hor	0.742	0.767808802
Uncharacterized protein C1orf198 [OS=Homo sapiens]	0.742	0.00507996
GPALPP motifs-containing protein 1 [OS=Homo sapiens]	0.742	0.004567573
Serine/threonine-protein kinase VRK2 [OS=Homo sapiens]	0.742	0.000401921
Lipolysis-stimulated lipoprotein receptor [OS=Homo sapiens]	0.742	0.001044896
Dual specificity protein kinase CLK4 [OS=Homo sapiens]	0.742	0.301965606
Exosome component 10 [OS=Homo sapiens]	0.743	0.01127584
Guanine nucleotide-binding protein-like 3 [OS=Homo sapiens]	0.743	0.430590555
Cytochrome c oxidase subunit 5B, mitochondrial [OS=Homo sapiens]	0.743	0.528004659
NADH dehydrogenase [ubiquinone] 1 beta subcomplex subunit 4 [OS=Homo sa	0.743	0.692675609
Complex I assembly factor TMEM126B, mitochondrial [OS=Homo sapiens]	0.743	0.330467887
Serine/arginine repetitive matrix protein 2 [OS=Homo sapiens]	0.744	0.175644067
Cytochrome b-c1 complex subunit 6, mitochondrial [OS=Homo sapiens]	0.744	0.529179964
Rac GTPase-activating protein 1 [OS=Homo sapiens]	0.744	1.12922E-05
Non-structural maintenance of chromosomes element 3 homolog [OS=Homo sa	0.744	0.154978591
Protein polybromo-1 [OS=Homo sapiens]	0.744	0.080153849
BCL-6 corepressor-like protein 1 [OS=Homo sapiens]	0.744	0.975298407
EH domain-containing protein 4 [OS=Homo sapiens]	0.745	0.001097549
RNA binding motif protein, X-linked-like-1 [OS=Homo sapiens]	0.745	0.818485889
N-acylneuraminate cytidylyltransferase [OS=Homo sapiens]	0.746	0.003435981
rRNA-processing protein UTP23 homolog [OS=Homo sapiens]	0.746	0.483193588
Centrosomal protein of 152 kDa [OS=Homo sapiens]	0.746	0.700115539
Cold shock domain-containing protein E1 [OS=Homo sapiens]	0.747	0.001449001
Polypyrimidine tract-binding protein 3 [OS=Homo sapiens]	0.747	0.005217641
Catenin beta-1 [OS=Homo sapiens]	0.747	0.054036275
Centromere protein V [OS=Homo sapiens]	0.747	0.477721207
Centrosomal protein of 72 kDa [OS=Homo sapiens]	0.747	0.313958874
Nuclear pore complex protein Nup93 [OS=Homo sapiens]	0.748	0.314876174
Citron Rho-interacting kinase [OS=Homo sapiens]	0.748	0.000138441
Heterogeneous nuclear ribonucleoprotein L [OS=Homo sapiens]	0.748	0.076810255
Phospholipid-transporting ATPase IF [OS=Homo sapiens]	0.748	0.873335038
Plectin [OS=Homo sapiens]	0.749	0.387853154
Fanconi anemia group D2 protein [OS=Homo sapiens]	0.749	0.002591482
Regulation of nuclear pre-mRNA domain-containing protein 2 [OS=Homo sapien	0.749	0.148484236
SHC SH2 domain-binding protein 1 [OS=Homo sapiens]	0.749	0.020933624
NADH dehydrogenase [ubiquinone] iron-sulfur protein 7, mitochondrial [OS=Hor	0.749	0.606242827
Membrane-bound transcription factor site-2 protease [OS=Homo sapiens]	0.749	0.982578158
Interleukin enhancer-binding factor 3 [OS=Homo sapiens]	0.75	0.5957298
Cytochrome b-c1 complex subunit 1, mitochondrial [OS=Homo sapiens]	0.75	0.50513794
Transcription factor IIIB 90 kDa subunit [OS=Homo sapiens]	0.75	0.714464459
Meiotic nuclear division protein 1 homolog [OS=Homo sapiens]	0.75	0.645889421
Protein phosphatase 1 regulatory subunit 35 [OS=Homo sapiens]	0.75	0.005124607
Protein SCAF11 [OS=Homo sapiens]	0.751	0.3957478
Neurofilament heavy polypeptide [OS=Homo sapiens]	0.751	0.008938012
Nuclear envelope integral membrane protein 1 [OS=Homo sapiens]	0.751	0.810757657
Histone-lysine N-methyltransferase 2A [OS=Homo sapiens]	0.752	0.364313692
Thioredoxin-related transmembrane protein 2 [OS=Homo sapiens]	0.752	0.004091969
NADH dehydrogenase [ubiquinone] 1 beta subcomplex subunit 6 [OS=Homo sa	0.752	0.655342019

Calcium uniporter protein, mitochondrial [OS=Homo sapiens]	0.752	0.848131625
Cleavage stimulation factor subunit 1 [OS=Homo sapiens]	0.752	0.209523051
Pre-mRNA-processing factor 17 [OS=Homo sapiens]	0.752	0.82103805
Cytochrome c oxidase subunit 8A, mitochondrial [OS=Homo sapiens]	0.752	0.582264763
MBT domain-containing protein 1 [OS=Homo sapiens]	0.753	0.666881544
Ras-related protein Rap-2a [OS=Homo sapiens]	0.753	0.458328537
Mitochondrial folate transporter/carrier [OS=Homo sapiens]	0.753	0.749433242
Coronin-1C [OS=Homo sapiens]	0.754	0.500882286
NADH dehydrogenase [ubiquinone] iron-sulfur protein 5 [OS=Homo sapiens]	0.754	0.794841759
Meiosis regulator and mRNA stability factor 1 [OS=Homo sapiens]	0.754	0.176084743
Transmembrane protein 35B [OS=Homo sapiens]	0.754	0.436342419
TGF-beta receptor type-1 [OS=Homo sapiens]	0.754	0.020337203
Mitochondrial carrier homolog 1 [OS=Homo sapiens]	0.755	0.789649426
Transcription factor Dp-1 [OS=Homo sapiens]	0.755	0.167391069
Phospholipid-transporting ATPase IH [OS=Homo sapiens]	0.755	0.207358873
Zinc finger protein 593 [OS=Homo sapiens]	0.755	0.000659571
Nuclear pore complex protein Nup107 [OS=Homo sapiens]	0.756	0.74969271
WD repeat-containing protein 3 [OS=Homo sapiens]	0.756	0.799271809
NHL repeat-containing protein 2 [OS=Homo sapiens]	0.756	0.000168485
Probable mitochondrial glutathione transporter SLC25A40 [OS=Homo sapiens]	0.756	0.728618579
(E3-independent) E2 ubiquitin-conjugating enzyme [OS=Homo sapiens]	0.757	0.006662725
Synaptopodin [OS=Homo sapiens]	0.757	0.751509298
Centriolar coiled-coil protein of 110 kDa [OS=Homo sapiens]	0.757	0.005635234
PHD and RING finger domain-containing protein 1 [OS=Homo sapiens]	0.757	0.351585842
Pre-mRNA-processing factor 19 [OS=Homo sapiens]	0.758	0.231205079
RNA-binding protein 39 [OS=Homo sapiens]	0.758	0.007192171
M-phase phosphoprotein 6 [OS=Homo sapiens]	0.758	0.020317231
Protein SSXT [OS=Homo sapiens]	0.758	0.376548784
Pre-rRNA-processing protein TSR1 homolog [OS=Homo sapiens]	0.759	0.000400779
E3 ubiquitin-protein ligase RFWD3 [OS=Homo sapiens]	0.759	0.107972848
U2 small nuclear ribonucleoprotein B'' [OS=Homo sapiens]	0.76	0.254019427
NADH dehydrogenase [ubiquinone] 1 alpha subcomplex subunit 2 [OS=Homo sa	0.76	0.235102304
Regulator of microtubule dynamics protein 3 [OS=Homo sapiens]	0.76	0.874721838
Dual specificity protein kinase CLK2 [OS=Homo sapiens]	0.76	0.957349927
Cytoskeleton-associated protein 2 [OS=Homo sapiens]	0.761	0.000219772
Polypeptide N-acetylgalactosaminyltransferase 1 [OS=Homo sapiens]	0.761	0.080947077
Zinc finger protein 184 [OS=Homo sapiens]	0.761	0.172557659
Nuclear pore complex protein Nup98-Nup96 [OS=Homo sapiens]	0.762	0.744376676
rRNA 2'-O-methyltransferase fibrillarin [OS=Homo sapiens]	0.762	0.748646522
Equilibrative nucleobase transporter 1 [OS=Homo sapiens]	0.762	0.833466896
NADH dehydrogenase [ubiquinone] 1 alpha subcomplex subunit 9, mitochondri	0.763	0.709092838
60S ribosomal protein L22-like 1 [OS=Homo sapiens]	0.763	0.003439701
NADH dehydrogenase [ubiquinone] 1 alpha subcomplex subunit 11 [OS=Homo s	0.763	0.718533345
Plakophilin-4 [OS=Homo sapiens]	0.763	0.311835009
Ankyrin repeat domain-containing protein 11 [OS=Homo sapiens]	0.763	0.295979178
PHD finger protein 10 [OS=Homo sapiens]	0.763	0.965745292
TOX high mobility group box family member 4 [OS=Homo sapiens]	0.764	0.006760378
Peptidyl-prolyl cis-trans isomerase G [OS=Homo sapiens]	0.764	0.481881831
ATP-dependent zinc metalloprotease YME1L1 [OS=Homo sapiens]	0.764	0.571652235
Rab-interacting lysosomal protein [OS=Homo sapiens]	0.764	0.014012908
Homeobox protein Hox-C10 [OS=Homo sapiens]	0.764	0.536495207
Anillin [OS=Homo sapiens]	0.765	0.000353605
Flotillin-1 [OS=Homo sapiens]	0.765	0.565763292
Centromere protein H [OS=Homo sapiens]	0.765	0.002764437
Brain acid soluble protein 1 [OS=Homo sapiens]	0.766	0.207278554
Peroxisomal membrane protein PEX14 [OS=Homo sapiens]	0.766	0.147581635
Spindle and kinetochore-associated protein 2 [OS=Homo sapiens]	0.766	0.010032268
Transmembrane emp24 domain-containing protein 3 [OS=Homo sapiens]	0.766	0.074288576
Laminin subunit alpha-5 [OS=Homo sapiens]	0.766	0.881890785
Swi5-dependent recombination DNA repair protein 1 homolog [OS=Homo sapie	0.766	0.085592679
Protein enabled homolog [OS=Homo sapiens]	0.767	0.013327233
Voltage-dependent anion-selective channel protein 2 [OS=Homo sapiens]	0.767	0.753617704
Cleavage and polyadenylation specificity factor subunit 1 [OS=Homo sapiens]	0.767	0.581265756
Protein Wiz [OS=Homo sapiens]	0.767	0.206384025
PHD finger protein 6 [OS=Homo sapiens]	0.767	0.000718212
Mitochondrial inner membrane protein OXA1L [OS=Homo sapiens]	0.767	0.720277819
Deoxycytidylate deaminase [OS=Homo sapiens]	0.767	0.09208155
Interferon-stimulated 20 kDa exonuclease-like 2 [OS=Homo sapiens]	0.767	0.508583453
NF-kappa-B-repressing factor [OS=Homo sapiens]	0.768	0.7117595
Olfactory receptor 5AC2 [OS=Homo sapiens]	0.768	0.961570347
Integrator complex subunit 3 [OS=Homo sapiens]	0.769	0.082026625
Zinc transporter SLC39A7 [OS=Homo sapiens]	0.769	0.745107111
Zinc finger protein 777 [OS=Homo sapiens]	0.769	0.295491101
Cytochrome b-c1 complex subunit Rieske, mitochondrial [OS=Homo sapiens]	0.77	0.47488933
NADH dehydrogenase [ubiquinone] 1 beta subcomplex subunit 5, mitochondrial	0.771	0.676783581

1-acylglycerol-3-phosphate O-acyltransferase ABHD5 [OS=Homo sapiens]	0.771	0.589370246
Tetratricopeptide repeat protein 33 [OS=Homo sapiens]	0.771	0.578242941
Leucine zipper putative tumor suppressor 2 [OS=Homo sapiens]	0.771	0.605401622
Testin [OS=Homo sapiens]	0.772	1.87779E-05
Torsin-1A-interacting protein 2 [OS=Homo sapiens]	0.772	0.14707373
NADH dehydrogenase [ubiquinone] iron-sulfur protein 8, mitochondrial [OS=Hor	0.772	0.554272386
Nucleolar protein 14 [OS=Homo sapiens]	0.772	0.677470577
Inositol polyphosphate-5-phosphatase A [OS=Homo sapiens]	0.772	0.847992836
Protein disulfide-isomerase TMX3 [OS=Homo sapiens]	0.773	0.006656939
Symplekin [OS=Homo sapiens]	0.773	0.277774108
Twinkle mtDNA helicase [OS=Homo sapiens]	0.773	0.255997366
Cytochrome c oxidase subunit 7A-related protein, mitochondrial [OS=Homo sap	0.773	0.908912252
DNA repair protein RAD51 homolog 1 [OS=Homo sapiens]	0.773	0.197148366
MAP kinase-activated protein kinase 3 [OS=Homo sapiens]	0.773	0.005953261
MORC family CW-type zinc finger protein 3 [OS=Homo sapiens]	0.774	0.359062992
U4/U6 small nuclear ribonucleoprotein Prp31 [OS=Homo sapiens]	0.774	0.120493624
DnaJ homolog subfamily C member 30, mitochondrial [OS=Homo sapiens]	0.774	0.674813038
KAT8 regulatory NSL complex subunit 3 [OS=Homo sapiens]	0.774	0.562151005
Mitochondrial carrier homolog 2 [OS=Homo sapiens]	0.775	0.645876653
Probable cytosolic iron-sulfur protein assembly protein CIAO1 [OS=Homo sapie	0.775	0.001567834
THO complex subunit 7 homolog [OS=Homo sapiens]	0.775	0.364825877
BRCA2-interacting transcriptional repressor EMSY [OS=Homo sapiens]	0.775	0.108534144
Heterogeneous nuclear ribonucleoprotein H [OS=Homo sapiens]	0.776	0.194826352
RNA-binding protein 25 [OS=Homo sapiens]	0.776	0.316274388
Maternal embryonic leucine zipper kinase [OS=Homo sapiens]	0.776	0.00569783
IQ calmodulin-binding motif-containing protein 1 [OS=Homo sapiens]	0.776	0.001025109
Smoothelin [OS=Homo sapiens]	0.778	2.41576E-05
Glypican-1 [OS=Homo sapiens]	0.778	0.718049002
Cytoskeleton-associated protein 2-like [OS=Homo sapiens]	0.778	0.000288755
Serine/arginine repetitive matrix protein 1 [OS=Homo sapiens]	0.779	0.046452352
Caveolae-associated protein 1 [OS=Homo sapiens]	0.779	0.113728972
GRIP and coiled-coil domain-containing protein 2 [OS=Homo sapiens]	0.779	0.00242367
Solute carrier family 2, facilitated glucose transporter member 1 [OS=Homo sap	0.779	0.316508726
Activator of basal transcription 1 [OS=Homo sapiens]	0.779	0.992829553
Probable ATP-dependent RNA helicase DDX23 [OS=Homo sapiens]	0.78	0.350931896
Nuclear pore complex protein Nup85 [OS=Homo sapiens]	0.78	0.813315015
Kinesin-like protein KIF23 [OS=Homo sapiens]	0.78	0.018554013
AF4/FMR2 family member 4 [OS=Homo sapiens]	0.78	0.599531902
Transmembrane protein 87A [OS=Homo sapiens]	0.78	0.073676899
Copper homeostasis protein cutC homolog [OS=Homo sapiens]	0.78	0.695622438
Bystin [OS=Homo sapiens]	0.781	0.014522862
NADH dehydrogenase [ubiquinone] flavoprotein 1, mitochondrial [OS=Homo sa	0.781	0.490368999
Occludin [OS=Homo sapiens]	0.781	0.617164933
Upstream-binding protein 1 [OS=Homo sapiens]	0.781	0.477381444
p21-activated protein kinase-interacting protein 1 [OS=Homo sapiens]	0.781	0.191405824
E3 ubiquitin-protein ligase RING2 [OS=Homo sapiens]	0.781	0.130680716
Ribosomal biogenesis factor [OS=Homo sapiens]	0.781	0.253736636
ATP synthase subunit ATP5MJ, mitochondrial [OS=Homo sapiens]	0.782	0.05526699
Parafibromin [OS=Homo sapiens]	0.783	0.001745271
Phosphate carrier protein, mitochondrial [OS=Homo sapiens]	0.783	0.753554113
Zinc finger protein 185 [OS=Homo sapiens]	0.783	0.138455872
Mitochondrial import inner membrane translocase subunit Tim17-B [OS=Homo	0.783	0.967481059
F-box-like/WD repeat-containing protein TBL1XR1 [OS=Homo sapiens]	0.784	0.002702543
Nucleoporin NDC1 [OS=Homo sapiens]	0.784	0.527742806
E3 ubiquitin-protein ligase AMFR [OS=Homo sapiens]	0.784	0.022263029
Serine beta-lactamase-like protein LACTB, mitochondrial [OS=Homo sapiens]	0.784	0.863859771
PHD finger protein 19 [OS=Homo sapiens]	0.784	0.07514417
Cyclin-dependent kinase 12 [OS=Homo sapiens]	0.785	0.13150335
Guanine nucleotide-binding protein G(s) subunit alpha isoforms short [OS=Hom	0.785	0.408617664
Zinc finger MYM-type protein 1 [OS=Homo sapiens]	0.785	0.08490944
Transcription initiation factor TFIID subunit 4 [OS=Homo sapiens]	0.785	0.598104503
Brain-enriched guanylate kinase-associated protein [OS=Homo sapiens]	0.785	0.71485852
Asparagine synthetase [glutamine-hydrolyzing] [OS=Homo sapiens]	0.786	0.000167581
Mesencephalic astrocyte-derived neurotrophic factor [OS=Homo sapiens]	0.786	4.60488E-05
60S ribosomal protein L38 [OS=Homo sapiens]	0.786	0.292063868
Ribonuclease H2 subunit B [OS=Homo sapiens]	0.786	0.02677411
SPATS2-like protein [OS=Homo sapiens]	0.786	0.015494636
F-box only protein 28 [OS=Homo sapiens]	0.786	0.478082423
BET1-like protein [OS=Homo sapiens]	0.786	0.151973318
C-1-tetrahydrofolate synthase, cytoplasmic [OS=Homo sapiens]	0.787	0.000300472
Histone RNA hairpin-binding protein [OS=Homo sapiens]	0.787	0.002144249
OCIA domain-containing protein 1 [OS=Homo sapiens]	0.787	0.930870418
Heterogeneous nuclear ribonucleoprotein A1 [OS=Homo sapiens]	0.788	0.361518798
Sister chromatid cohesion protein PDS5 homolog B [OS=Homo sapiens]	0.788	0.076301681
ADP/ATP translocase 2 [OS=Homo sapiens]	0.788	0.636038891

Remodeling and spacing factor 1 [OS=Homo sapiens]	0.788	0.86963523
Cytochrome c1, heme protein, mitochondrial [OS=Homo sapiens]	0.788	0.766507273
POC1 centriolar protein homolog A [OS=Homo sapiens]	0.788	0.002103302
LIM domain and actin-binding protein 1 [OS=Homo sapiens]	0.789	0.875555095
Acylglycerol kinase, mitochondrial [OS=Homo sapiens]	0.789	0.879362688
Nucleoporin Nup37 [OS=Homo sapiens]	0.789	0.495173566
NADH dehydrogenase [ubiquinone] 1 alpha subcomplex subunit 7 [OS=Homo sa	0.789	0.823580902
Histone-lysine N-methyltransferase NSD3 [OS=Homo sapiens]	0.789	0.438766305
Dedicator of cytokinesis protein 5 [OS=Homo sapiens]	0.789	0.40597688
Splicing factor 3B subunit 2 [OS=Homo sapiens]	0.79	0.157018504
Set1/Ash2 histone methyltransferase complex subunit ASH2 [OS=Homo sapiens	0.79	0.231536063
Histone-lysine N-methyltransferase EHMT2 [OS=Homo sapiens]	0.79	0.223002218
Uncharacterized protein FLJ45252 [OS=Homo sapiens]	0.79	0.037366529
Pre-mRNA-processing-splicing factor 8 [OS=Homo sapiens]	0.792	0.126660235
Armadillo repeat-containing protein 10 [OS=Homo sapiens]	0.792	0.590837526
5'(3')-deoxyribonucleotidase, cytosolic type [OS=Homo sapiens]	0.792	0.077394611
39S ribosomal protein L53, mitochondrial [OS=Homo sapiens]	0.792	0.878671574
Protein Hook homolog 3 [OS=Homo sapiens]	0.793	0.003184316
Zinc finger protein 281 [OS=Homo sapiens]	0.793	0.30646377
DNA repair and recombination protein RAD54B [OS=Homo sapiens]	0.793	0.019637543
Histone-lysine N-methyltransferase 2B [OS=Homo sapiens]	0.794	0.480256996
Polyadenylate-binding protein 2 [OS=Homo sapiens]	0.794	0.379014983
DNA methyltransferase 1-associated protein 1 [OS=Homo sapiens]	0.794	0.76716186
PAX3- and PAX7-binding protein 1 [OS=Homo sapiens]	0.794	0.000721238
Complex I intermediate-associated protein 30, mitochondrial [OS=Homo sapier	0.794	0.527151908
G2 and S phase-expressed protein 1 [OS=Homo sapiens]	0.794	0.999704916
Holliday junction recognition protein [OS=Homo sapiens]	0.794	0.000827386
WD40 repeat-containing protein SMU1 [OS=Homo sapiens]	0.795	0.209015778
Transforming acidic coiled-coil-containing protein 3 [OS=Homo sapiens]	0.795	0.035394927
Nucleolar protein 3 [OS=Homo sapiens]	0.795	0.080322319
Kinesin-like protein KIF2C [OS=Homo sapiens]	0.796	0.0003911
Chromosome alignment-maintaining phosphoprotein 1 [OS=Homo sapiens]	0.796	0.09173697
Flotillin-2 [OS=Homo sapiens]	0.796	0.863067305
Pleckstrin homology-like domain family B member 2 [OS=Homo sapiens]	0.796	0.042629832
Protein Tob2 [OS=Homo sapiens]	0.796	0.281716852
Eukaryotic translation initiation factor 4E-binding protein 1 [OS=Homo sapiens]	0.796	0.346062212
Next to BRCA1 gene 1 protein [OS=Homo sapiens]	0.796	0.999491676
Mitochondrial pyruvate carrier 2 [OS=Homo sapiens]	0.796	0.504679015
Obg-like ATPase 1 [OS=Homo sapiens]	0.797	0.000774909
Mitochondrial fission process protein 1 [OS=Homo sapiens]	0.797	0.750182025
Importin-8 [OS=Homo sapiens]	0.798	0.054645949
Serine/Arginine-related protein 53 [OS=Homo sapiens]	0.798	0.106271681
PCNA-associated factor [OS=Homo sapiens]	0.798	0.021686245
Histone-lysine N-methyltransferase SETD2 [OS=Homo sapiens]	0.798	0.132515922
Putative RNA-binding protein 15B [OS=Homo sapiens]	0.798	0.771121675
Sperm acrosome-associated protein 5 [OS=Homo sapiens]	0.798	0.040364472
Leucine zipper transcription factor-like protein 1 [OS=Homo sapiens]	0.799	0.001650293
DNA topoisomerase 2-beta [OS=Homo sapiens]	0.8	0.957907595
Histone acetyltransferase KAT7 [OS=Homo sapiens]	0.8	0.168348057
HAUS augmin-like complex subunit 4 [OS=Homo sapiens]	0.8	0.031625788
Kinetochore protein NDC80 homolog [OS=Homo sapiens]	0.801	0.000254349
Condensin-2 complex subunit G2 [OS=Homo sapiens]	0.801	0.417704956
HMG domain-containing protein 4 [OS=Homo sapiens]	0.801	0.691201635
ATP-dependent RNA helicase DDX24 [OS=Homo sapiens]	0.802	0.686785274
Upstream stimulatory factor 1 [OS=Homo sapiens]	0.802	0.12648732
Trafficking protein particle complex subunit 6B [OS=Homo sapiens]	0.802	0.013084945
CD2-associated protein [OS=Homo sapiens]	0.803	0.000168679
Transducin beta-like protein 3 [OS=Homo sapiens]	0.803	0.934203354
Guanine nucleotide-binding protein subunit alpha-11 [OS=Homo sapiens]	0.803	0.382688564
Serine/arginine-rich splicing factor 3 [OS=Homo sapiens]	0.803	0.234950199
Putative small nuclear ribonucleoprotein G-like protein 15 [OS=Homo sapiens]	0.803	0.25090403
U4/U6.U5 small nuclear ribonucleoprotein 27 kDa protein [OS=Homo sapiens]	0.803	0.560895817
Sperm-associated antigen 1 [OS=Homo sapiens]	0.803	0.023448863
Nucleolus and neural progenitor protein [OS=Homo sapiens]	0.803	0.576227503
COMM domain-containing protein 6 [OS=Homo sapiens]	0.803	0.296481669
Splicing factor 3A subunit 1 [OS=Homo sapiens]	0.804	0.162838822
Adenosine 5'-monophosphoramidase HINT3 [OS=Homo sapiens]	0.804	0.998140283
Disheveled-associated activator of morphogenesis 1 [OS=Homo sapiens]	0.804	0.38872803
DNA excision repair protein ERCC-6-like [OS=Homo sapiens]	0.805	0.002763726
Thymidylate synthase [OS=Homo sapiens]	0.805	0.008997478
Retinitis pigmentosa 9 protein [OS=Homo sapiens]	0.805	0.008230964
Zinc finger protein 581 [OS=Homo sapiens]	0.805	0.735085111
Stathmin [OS=Homo sapiens]	0.806	0.007410364
Cytochrome c oxidase subunit 4 isoform 1, mitochondrial [OS=Homo sapiens]	0.806	0.749015073
Muscleblind-like protein 1 [OS=Homo sapiens]	0.806	0.000271201

Vesicle transport protein GOT1B [OS=Homo sapiens]	0.806	0.149161603
Zinc finger protein 532 [OS=Homo sapiens]	0.806	0.434062007
ETS-related transcription factor Elf-1 [OS=Homo sapiens]	0.806	0.257413897
Poly(A)-specific ribonuclease PARN [OS=Homo sapiens]	0.807	0.205337127
Cleavage stimulation factor subunit 2 tau variant [OS=Homo sapiens]	0.807	0.19028317
Transcription factor E3 [OS=Homo sapiens]	0.807	0.366421154
Nucleosome assembly protein 1-like 1 [OS=Homo sapiens]	0.808	0.563155099
Thyroid receptor-interacting protein 6 [OS=Homo sapiens]	0.808	0.249670794
A-kinase anchor protein 1, mitochondrial [OS=Homo sapiens]	0.808	0.508209641
Transcriptional activator protein Pur-alpha [OS=Homo sapiens]	0.809	0.099296878
c-Myc-binding protein [OS=Homo sapiens]	0.809	0.006239512
Splicing factor 3B subunit 4 [OS=Homo sapiens]	0.809	0.097661984
Transmembrane protein 126A [OS=Homo sapiens]	0.809	0.75069565
Polypyrimidine tract-binding protein 2 [OS=Homo sapiens]	0.809	0.389083638
Nucleolar and coiled-body phosphoprotein 1 [OS=Homo sapiens]	0.81	0.678394676
Zinc phosphodiesterase ELAC protein 2 [OS=Homo sapiens]	0.81	0.001384928
Fanconi anemia group I protein [OS=Homo sapiens]	0.81	0.001986379
TAR DNA-binding protein 43 [OS=Homo sapiens]	0.81	0.013289153
Centrosomal protein of 97 kDa [OS=Homo sapiens]	0.81	0.311965883
Nuclear autoantigen Sp-100 [OS=Homo sapiens]	0.81	0.84740836
Signal transducer and activator of transcription 5A [OS=Homo sapiens]	0.811	0.918185543
Cyclic GMP-AMP synthase [OS=Homo sapiens]	0.811	0.375570581
NADH dehydrogenase [ubiquinone] iron-sulfur protein 3, mitochondrial [OS=Hor	0.811	0.238865316
Nucleolar GTP-binding protein 2 [OS=Homo sapiens]	0.811	0.248858037
Evolutionarily conserved signaling intermediate in Toll pathway, mitochondrial [(	0.811	0.368668715
RNA-binding protein 3 [OS=Homo sapiens]	0.811	0.338250806
NADH dehydrogenase [ubiquinone] 1 alpha subcomplex subunit 6 [OS=Homo sa	0.811	0.056170319
Complex I assembly factor TIMMDC1, mitochondrial [OS=Homo sapiens]	0.811	0.705660691
Speckle targeted PIP5K1A-regulated poly(A) polymerase [OS=Homo sapiens]	0.811	0.085798614
ALX homeobox protein 1 [OS=Homo sapiens]	0.811	0.213142128
DNA-directed RNA polymerase I subunit RPA1 [OS=Homo sapiens]	0.812	0.260628636
Peptidyl-prolyl cis-trans isomerase-like 4 [OS=Homo sapiens]	0.812	0.002146327
Cytochrome c oxidase assembly protein COX20, mitochondrial [OS=Homo sapi	0.812	0.893144713
Splicing factor 3B subunit 6 [OS=Homo sapiens]	0.812	0.394830479
Protein OSCP1 [OS=Homo sapiens]	0.812	0.865123083
Centrosomal protein of 78 kDa [OS=Homo sapiens]	0.812	0.698017118
Serine/threonine-protein phosphatase 6 regulatory subunit 1 [OS=Homo sapien	0.813	0.236181434
Programmed cell death protein 7 [OS=Homo sapiens]	0.813	0.912282541
Macrophage colony-stimulating factor 1 [OS=Homo sapiens]	0.813	0.617521557
Porphobilinogen deaminase [OS=Homo sapiens]	0.814	0.009627028
Cilia- and flagella-associated protein 20 [OS=Homo sapiens]	0.814	0.000447065
Protein MIS12 homolog [OS=Homo sapiens]	0.814	0.018958392
Dendritic cell-specific transmembrane protein [OS=Homo sapiens]	0.814	0.996464475
Centrin-3 [OS=Homo sapiens]	0.815	0.073051587
Phosphoribosyltransferase domain-containing protein 1 [OS=Homo sapiens]	0.815	0.895446758
SH3 domain-binding protein 1 [OS=Homo sapiens]	0.815	0.675735598
Ribonuclease P protein subunit p25-like protein [OS=Homo sapiens]	0.815	0.40949982
Immunoglobulin superfamily member 3 [OS=Homo sapiens]	0.815	0.765741879
U5 small nuclear ribonucleoprotein 200 kDa helicase [OS=Homo sapiens]	0.816	0.202976637
ATP-dependent RNA helicase DHX15 [OS=Homo sapiens]	0.816	0.422143385
Vesicle-associated membrane protein-associated protein A [OS=Homo sapiens]	0.816	0.089874095
Zinc finger CCCH-type antiviral protein 1-like [OS=Homo sapiens]	0.816	0.003201701
Metal cation symporter ZIP14 [OS=Homo sapiens]	0.816	0.007025148
Protein LTV1 homolog [OS=Homo sapiens]	0.816	0.007677019
DNA polymerase epsilon subunit 2 [OS=Homo sapiens]	0.816	0.030132138
Histone-lysine N-methyltransferase SETD1A [OS=Homo sapiens]	0.816	0.368341211
NADH dehydrogenase [ubiquinone] 1 beta subcomplex subunit 11, mitochondri	0.816	0.897854648
Ankyrin repeat and MYND domain-containing protein 2 [OS=Homo sapiens]	0.816	0.999988117
Zinc transporter ZIP10 [OS=Homo sapiens]	0.816	1
NAD-dependent protein deacylase sirtuin-5, mitochondrial [OS=Homo sapiens]	0.816	0.988387357
Chromosome-associated kinesin KIF4A [OS=Homo sapiens]	0.817	9.52988E-05
Retinoblastoma-binding protein 5 [OS=Homo sapiens]	0.817	0.074178989
Cell division cycle-associated protein 3 [OS=Homo sapiens]	0.817	0.670999426
Nucleolar protein 16 [OS=Homo sapiens]	0.817	0.7804549
Paraneoplastic antigen-like protein 6A [OS=Homo sapiens]	0.817	0.990307198
Disintegrin and metalloproteinase domain-containing protein 9 [OS=Homo sapi	0.818	0.015544851
Spliceosome-associated protein CWC15 homolog [OS=Homo sapiens]	0.818	0.312420267
U3 small nucleolar RNA-associated protein 25 homolog [OS=Homo sapiens]	0.818	0.676847494
Junctional adhesion molecule A [OS=Homo sapiens]	0.818	0.766765005
DNA-directed RNA polymerase II subunit RPB1 [OS=Homo sapiens]	0.819	0.420160663
NADH dehydrogenase [ubiquinone] iron-sulfur protein 2, mitochondrial [OS=Hor	0.819	0.318659017
V-type proton ATPase 116 kDa subunit a 1 [OS=Homo sapiens]	0.819	0.472882427
Cytochrome c oxidase subunit 5A, mitochondrial [OS=Homo sapiens]	0.819	0.642744347
RNA-binding protein 42 [OS=Homo sapiens]	0.819	0.037519949
DnaJ homolog subfamily B member 4 [OS=Homo sapiens]	0.819	0.982003452

Neurogranin [OS=Homo sapiens]	0.819	0.184712104
Eukaryotic initiation factor 4A-III [OS=Homo sapiens]	0.82	0.236453105
Heat shock protein beta-1 [OS=Homo sapiens]	0.82	0.927519271
YEATS domain-containing protein 4 [OS=Homo sapiens]	0.82	0.962347256
Nucleus accumbens-associated protein 2 [OS=Homo sapiens]	0.82	0.679578163
Mitotic spindle assembly checkpoint protein MAD2B [OS=Homo sapiens]	0.82	0.040524497
NADH dehydrogenase [ubiquinone] iron-sulfur protein 6, mitochondrial [OS=Hor	0.821	0.443312522
28S rRNA (cytosine-C(5))-methyltransferase [OS=Homo sapiens]	0.821	0.288007751
Condensin-2 complex subunit D3 [OS=Homo sapiens]	0.821	0.001449132
Structural maintenance of chromosomes protein 6 [OS=Homo sapiens]	0.821	0.982418669
Protein LLP homolog [OS=Homo sapiens]	0.821	0.283131055
Protein FAM76A [OS=Homo sapiens]	0.821	0.187434701
Calcineurin-binding protein cabin-1 [OS=Homo sapiens]	0.821	0.469732538
AF4/FMR2 family member 1 [OS=Homo sapiens]	0.821	0.998622936
DENN domain-containing protein 11 [OS=Homo sapiens]	0.821	0.550124486
NADH-ubiquinone oxidoreductase 75 kDa subunit, mitochondrial [OS=Homo sa	0.822	0.553172794
U2 small nuclear ribonucleoprotein A' [OS=Homo sapiens]	0.822	0.427454646
Ankyrin-1 [OS=Homo sapiens]	0.822	0.013669384
DNA primase large subunit [OS=Homo sapiens]	0.822	0.003354863
E3 ubiquitin-protein ligase ZFP91 [OS=Homo sapiens]	0.822	0.025557841
FAD-dependent oxidoreductase domain-containing protein 2 [OS=Homo sapien	0.822	0.034843174
Protein FAM83H [OS=Homo sapiens]	0.822	0.111110733
Catenin alpha-1 [OS=Homo sapiens]	0.823	0.004640481
U1 small nuclear ribonucleoprotein 70 kDa [OS=Homo sapiens]	0.823	0.255356713
Cytospin-A [OS=Homo sapiens]	0.823	0.736287399
Cyclin-dependent kinase 9 [OS=Homo sapiens]	0.823	0.01620796
Cyclic AMP-dependent transcription factor ATF-6 beta [OS=Homo sapiens]	0.823	0.898322647
Krueppel-like factor 16 [OS=Homo sapiens]	0.823	0.001187459
Protein yippee-like 5 [OS=Homo sapiens]	0.823	0.456247483
Keratin, type II cytoskeletal 1 [OS=Homo sapiens]	0.824	0.999981305
Bcl-2-like protein 13 [OS=Homo sapiens]	0.824	0.501466837
Zinc finger protein 768 [OS=Homo sapiens]	0.824	0.062253454
TBC1 domain family member 2A [OS=Homo sapiens]	0.824	0.24842067
PAS domain-containing serine/threonine-protein kinase [OS=Homo sapiens]	0.824	0.771046954
Multiple myeloma tumor-associated protein 2 [OS=Homo sapiens]	0.825	0.256489045
Essential MCU regulator, mitochondrial [OS=Homo sapiens]	0.825	0.795268355
NADH dehydrogenase [ubiquinone] 1 beta subcomplex subunit 10 [OS=Homo sa	0.826	0.790972042
Actin-related protein 8 [OS=Homo sapiens]	0.826	0.014869317
Biogenesis of lysosome-related organelles complex 1 subunit 3 [OS=Homo sapi	0.826	0.602680812
Zinc finger CCCH domain-containing protein 3 [OS=Homo sapiens]	0.826	0.80037326
ARL14 effector protein [OS=Homo sapiens]	0.826	0.547703273
mRNA turnover protein 4 homolog [OS=Homo sapiens]	0.827	0.008387095
Paralemmin-1 [OS=Homo sapiens]	0.827	0.923609211
Magnesium transporter MRS2 homolog, mitochondrial [OS=Homo sapiens]	0.827	0.980621492
Probable ATP-dependent RNA helicase DDX49 [OS=Homo sapiens]	0.827	0.009319661
Zinc finger and BTB domain-containing protein 1 [OS=Homo sapiens]	0.827	0.491962157
Protein ARV1 [OS=Homo sapiens]	0.827	0.62855749
Phosphatidylinositol 3,4,5-trisphosphate-dependent Rac exchanger 1 protein [C	0.827	0.507430612
UMP-CMP kinase [OS=Homo sapiens]	0.828	0.365363205
Serine/threonine-protein phosphatase PGAM5, mitochondrial [OS=Homo sapier	0.828	0.863495199
Gamma-soluble NSF attachment protein [OS=Homo sapiens]	0.828	0.009341016
WD repeat-containing protein 74 [OS=Homo sapiens]	0.828	0.614009209
CLK4-associating serine/arginine rich protein [OS=Homo sapiens]	0.828	0.000138308
Glutamine synthetase [OS=Homo sapiens]	0.828	0.038932052
Ubiquitin-conjugating enzyme E2 E3 [OS=Homo sapiens]	0.828	0.190643683
Phosphatidylinositol N-acetylglucosaminyltransferase subunit Q [OS=Homo sap	0.828	0.999997992
[Pyruvate dehydrogenase (acetyl-transferring)] kinase isozyme 2, mitochondrial	0.829	0.890452471
116 kDa U5 small nuclear ribonucleoprotein component [OS=Homo sapiens]	0.83	0.294767417
Ribosome biogenesis protein WDR12 [OS=Homo sapiens]	0.83	0.040853947
RCC1 domain-containing protein 1 [OS=Homo sapiens]	0.83	0.062049923
WW domain-binding protein 2 [OS=Homo sapiens]	0.83	0.854775743
Nucleoporin Nup43 [OS=Homo sapiens]	0.83	0.101820056
Multiple coagulation factor deficiency protein 2 [OS=Homo sapiens]	0.83	0.474179694
Complement decay-accelerating factor [OS=Homo sapiens]	0.83	0.799687919
Probable ATP-dependent RNA helicase DDX5 [OS=Homo sapiens]	0.831	0.002468389
MICOS complex subunit MIC13 [OS=Homo sapiens]	0.831	0.965587279
Eukaryotic translation initiation factor 2 subunit 1 [OS=Homo sapiens]	0.832	0.00610831
Golgi integral membrane protein 4 [OS=Homo sapiens]	0.832	0.514875567
NADH dehydrogenase [ubiquinone] 1 alpha subcomplex subunit 5 [OS=Homo sa	0.832	0.58801885
E3 ubiquitin-protein ligase XIAP [OS=Homo sapiens]	0.832	0.539582409
Protein FRG1 [OS=Homo sapiens]	0.832	0.210616348
Beta-galactosidase-1-like protein 2 [OS=Homo sapiens]	0.832	0.815342644
DNA replication licensing factor MCM7 [OS=Homo sapiens]	0.833	0.002011121
DNA replication licensing factor MCM6 [OS=Homo sapiens]	0.833	0.014062008
U4/U6.U5 tri-snRNP-associated protein 2 [OS=Homo sapiens]	0.833	0.348884034

RNA-binding protein 26 [OS=Homo sapiens]	0.833	0.020221383
Condensin complex subunit 3 [OS=Homo sapiens]	0.833	0.660486136
Zinc finger MYM-type protein 4 [OS=Homo sapiens]	0.833	0.452674619
5-aminolevulinate synthase, non-specific, mitochondrial [OS=Homo sapiens]	0.833	0.030962058
Unconventional myosin-XIX [OS=Homo sapiens]	0.833	0.621171099
Structural maintenance of chromosomes protein 4 [OS=Homo sapiens]	0.834	0.000583724
SH2 domain-containing protein 4A [OS=Homo sapiens]	0.834	0.015582818
Phosphatidylinositol 4-kinase alpha [OS=Homo sapiens]	0.834	0.757101655
Mediator of RNA polymerase II transcription subunit 20 [OS=Homo sapiens]	0.834	0.70291397
Thymidine kinase, cytosolic [OS=Homo sapiens]	0.835	0.025524567
NADH dehydrogenase [ubiquinone] 1 alpha subcomplex subunit 10, mitochondı	0.835	0.803438569
Phosphorylated adapter RNA export protein [OS=Homo sapiens]	0.835	0.401853605
Zinc finger MYM-type protein 3 [OS=Homo sapiens]	0.835	0.442980121
CD70 antigen [OS=Homo sapiens]	0.835	0.0293319
Protein ENL [OS=Homo sapiens]	0.835	0.430553168
UPF0711 protein C18orf21 [OS=Homo sapiens]	0.835	0.578400745
Lysine-specific histone demethylase 2 [OS=Homo sapiens]	0.835	0.462111668
Cytochrome c oxidase subunit 7C, mitochondrial [OS=Homo sapiens]	0.835	0.993232419
Alpha-actinin-4 [OS=Homo sapiens]	0.836	0.563151557
7SK snRNA methylphosphate capping enzyme [OS=Homo sapiens]	0.836	0.010519245
Single-stranded DNA-binding protein 3 [OS=Homo sapiens]	0.836	0.100618858
Disco-interacting protein 2 homolog A [OS=Homo sapiens]	0.836	0.472751998
Cell division control protein 45 homolog [OS=Homo sapiens]	0.836	0.397677608
BEN domain-containing protein 3 [OS=Homo sapiens]	0.836	0.395746297
Tetraspanin-14 [OS=Homo sapiens]	0.836	0.895960482
Utrophin [OS=Homo sapiens]	0.837	0.087641243
DNA replication licensing factor MCM4 [OS=Homo sapiens]	0.837	9.99796E-05
Splicing factor, proline- and glutamine-rich [OS=Homo sapiens]	0.837	0.620168808
Pericentriolar material 1 protein [OS=Homo sapiens]	0.837	0.375975859
Double-stranded RNA-specific adenosine deaminase [OS=Homo sapiens]	0.837	0.514531794
Replication termination factor 2 [OS=Homo sapiens]	0.837	0.095004451
V-type proton ATPase 116 kDa subunit a 2 [OS=Homo sapiens]	0.837	0.827451253
PHD finger protein 20 [OS=Homo sapiens]	0.837	0.996870422
Band 4.1-like protein 2 [OS=Homo sapiens]	0.839	0.074413575
RNA helicase aquarius [OS=Homo sapiens]	0.839	0.237287911
Beta-catenin-like protein 1 [OS=Homo sapiens]	0.839	0.0465535
Ras-related protein Rab-21 [OS=Homo sapiens]	0.839	0.636584729
Paraplegin [OS=Homo sapiens]	0.839	0.735225229
Splicing factor, suppressor of white-apricot homolog [OS=Homo sapiens]	0.839	0.544078111
Chromatin assembly factor 1 subunit A [OS=Homo sapiens]	0.839	0.724149467
Kinesin-like protein KIF15 [OS=Homo sapiens]	0.84	0.001493192
Serine/arginine-rich splicing factor 4 [OS=Homo sapiens]	0.84	0.354125506
ZW10 interactor [OS=Homo sapiens]	0.84	0.024567044
Phosphatidylserine synthase 2 [OS=Homo sapiens]	0.84	0.56359889
Rho-associated protein kinase 1 [OS=Homo sapiens]	0.841	0.036259763
Pescadillo homolog [OS=Homo sapiens]	0.841	0.368396176
Centrosome-associated protein CEP250 [OS=Homo sapiens]	0.841	0.036275226
Menin [OS=Homo sapiens]	0.841	0.947199087
Striatin [OS=Homo sapiens]	0.841	0.055040077
Guanine nucleotide-binding protein G(I)/G(S)/G(O) subunit gamma-12 [OS=Hon	0.841	0.858864933
Claudin-3 [OS=Homo sapiens]	0.841	0.582593089
Integrator complex subunit 8 [OS=Homo sapiens]	0.841	0.860132404
PAX-interacting protein 1 [OS=Homo sapiens]	0.841	0.598921926
GON-4-like protein [OS=Homo sapiens]	0.841	0.744546677
Coronin-2A [OS=Homo sapiens]	0.841	0.5460781
Serine/arginine-rich splicing factor 1 [OS=Homo sapiens]	0.842	0.337072526
PIN2/TERF1-interacting telomerase inhibitor 1 [OS=Homo sapiens]	0.842	0.000371244
Protein BUD31 homolog [OS=Homo sapiens]	0.842	0.202924936
DNA replication licensing factor MCM2 [OS=Homo sapiens]	0.843	1.93777E-08
Splicing factor 3A subunit 3 [OS=Homo sapiens]	0.843	0.087884835
Cleavage stimulation factor subunit 2 [OS=Homo sapiens]	0.843	0.386226802
RNA polymerase-associated protein LEO1 [OS=Homo sapiens]	0.843	0.130834055
Chromodomain-helicase-DNA-binding protein 2 [OS=Homo sapiens]	0.843	0.988461961
Fatty acyl-CoA reductase 1 [OS=Homo sapiens]	0.843	0.074555665
Tetratricopeptide repeat protein 27 [OS=Homo sapiens]	0.843	0.648341597
Calcium and integrin-binding protein 1 [OS=Homo sapiens]	0.843	0.502693664
Intraflagellar transport protein 25 homolog [OS=Homo sapiens]	0.843	0.849886951
AT-rich interactive domain-containing protein 4A [OS=Homo sapiens]	0.843	0.390428077
Cyclin-dependent kinase 10 [OS=Homo sapiens]	0.843	0.436162697
Pre-mRNA-splicing factor SLU7 [OS=Homo sapiens]	0.843	0.521229347
RNA polymerase II elongation factor ELL [OS=Homo sapiens]	0.843	0.545719393
BTB/POZ domain-containing adapter for CUL3-mediated RhoA degradation prot	0.843	0.102231424
Eukaryotic translation initiation factor 2 subunit 3 [OS=Homo sapiens]	0.844	5.65337E-05
Sister chromatid cohesion protein PDS5 homolog A [OS=Homo sapiens]	0.844	0.002536833
Ribosome biogenesis protein SLX9 homolog [OS=Homo sapiens]	0.844	0.081417212

TRPM8 channel-associated factor 1 [OS=Homo sapiens]	0.844	0.32795083
GPN-loop GTPase 2 [OS=Homo sapiens]	0.844	0.021720848
Methylcytosine dioxygenase TET2 [OS=Homo sapiens]	0.844	0.967223781
Mitochondrial ornithine transporter 1 [OS=Homo sapiens]	0.844	0.38501216
Serrate RNA effector molecule homolog [OS=Homo sapiens]	0.845	0.001266444
Transcription intermediary factor 1-beta [OS=Homo sapiens]	0.845	0.001815742
Regulator of chromosome condensation [OS=Homo sapiens]	0.845	0.059223984
ADP/ATP translocase 1 [OS=Homo sapiens]	0.845	0.909096234
Myosin phosphatase Rho-interacting protein [OS=Homo sapiens]	0.845	0.532740833
Cip1-interacting zinc finger protein [OS=Homo sapiens]	0.845	0.084577026
Dynamin-binding protein [OS=Homo sapiens]	0.845	0.870730987
Folylpolyglutamate synthase, mitochondrial [OS=Homo sapiens]	0.845	0.146766138
Nuclear receptor corepressor 1 [OS=Homo sapiens]	0.845	0.039929191
DNA-directed RNA polymerase I subunit RPA43 [OS=Homo sapiens]	0.845	0.638029543
E3 ubiquitin-protein ligase DZIP3 [OS=Homo sapiens]	0.845	0.081100422
Nucleolar transcription factor 1 [OS=Homo sapiens]	0.846	0.83457863
Pogo transposable element with ZNF domain [OS=Homo sapiens]	0.846	0.827958262
FERM, ARHGEF and pleckstrin domain-containing protein 1 [OS=Homo sapiens]	0.846	0.771237332
Guanine nucleotide-binding protein-like 3-like protein [OS=Homo sapiens]	0.846	0.051673384
Transmembrane protein 87B [OS=Homo sapiens]	0.846	0.238599564
Origin recognition complex subunit 5 [OS=Homo sapiens]	0.846	0.714931172
DNA-directed primase/polymerase protein [OS=Homo sapiens]	0.846	0.676789457
Uncharacterized protein NKAPD1 [OS=Homo sapiens]	0.846	0.772552212
Bromodomain-containing protein 8 [OS=Homo sapiens]	0.847	0.270120939
Large neutral amino acids transporter small subunit 1 [OS=Homo sapiens]	0.847	0.644745452
Cyclin-T1 [OS=Homo sapiens]	0.847	0.087818152
Monocarboxylate transporter 4 [OS=Homo sapiens]	0.847	0.584530881
Calcium uptake protein 1, mitochondrial [OS=Homo sapiens]	0.847	0.761224829
Protein max [OS=Homo sapiens]	0.847	0.715853625
Phosphotriesterase-related protein [OS=Homo sapiens]	0.847	0.748773399
Ran-binding protein 6 [OS=Homo sapiens]	0.848	0.530566064
Nuclear factor related to kappa-B-binding protein [OS=Homo sapiens]	0.848	0.892583528
Polycomb protein SUZ12 [OS=Homo sapiens]	0.848	0.084664179
RNA-binding motif protein, X-linked 2 [OS=Homo sapiens]	0.848	0.602877577
Mitochondrial inner membrane protease ATP23 homolog [OS=Homo sapiens]	0.848	0.198761559
Dol-P-Man:Man(7)GlcNAc(2)-PP-Dol alpha-1,6-mannosyltransferase [OS=Homo	0.848	0.994593046
Condensin complex subunit 2 [OS=Homo sapiens]	0.849	0.005581643
RNA-binding protein 4 [OS=Homo sapiens]	0.849	0.48268289
Survival of motor neuron-related-splicing factor 30 [OS=Homo sapiens]	0.849	0.16525007
Regulator of nonsense transcripts 3B [OS=Homo sapiens]	0.849	0.725762943
Xaa-Arg dipeptidase [OS=Homo sapiens]	0.849	0.012457608
TATA-box-binding protein [OS=Homo sapiens]	0.849	0.081131171
Sorting nexin-18 [OS=Homo sapiens]	0.849	0.998140745
E3 ubiquitin-protein ligase RNF5 [OS=Homo sapiens]	0.849	0.32470112
Phosphatidate phosphatase LPIN3 [OS=Homo sapiens]	0.849	0.05656315
DNA replication licensing factor MCM5 [OS=Homo sapiens]	0.85	0.001740946
Condensin complex subunit 1 [OS=Homo sapiens]	0.85	0.008972931
Paired amphipathic helix protein Sin3a [OS=Homo sapiens]	0.85	0.118336928
DNA-directed RNA polymerase II subunit RPB2 [OS=Homo sapiens]	0.85	0.1852671
Guanine nucleotide-binding protein G(i) subunit alpha-2 [OS=Homo sapiens]	0.85	0.838957208
Membrane-associated guanylate kinase, WW and PDZ domain-containing protei	0.85	0.986852063
Zinc finger protein OZF [OS=Homo sapiens]	0.85	0.58303465
Asparagine--tRNA ligase, cytoplasmic [OS=Homo sapiens]	0.851	0.123020731
Spliceosome RNA helicase DDX39B [OS=Homo sapiens]	0.851	0.374255204
Nucleoporin NUP188 [OS=Homo sapiens]	0.851	0.24473407
Guanine nucleotide-binding protein G(t) subunit alpha-1 [OS=Homo sapiens]	0.851	0.998342037
Protein ITPRID2 [OS=Homo sapiens]	0.851	0.851574129
DNA replication licensing factor MCM3 [OS=Homo sapiens]	0.852	4.16381E-07
Structural maintenance of chromosomes protein 2 [OS=Homo sapiens]	0.852	0.00334364
D-3-phosphoglycerate dehydrogenase [OS=Homo sapiens]	0.852	0.002050741
Splicing regulatory glutamine/lysine-rich protein 1 [OS=Homo sapiens]	0.852	0.006548871
Matrix remodeling-associated protein 8 [OS=Homo sapiens]	0.852	0.439488155
E3 ubiquitin-protein ligase RAD18 [OS=Homo sapiens]	0.852	0.007062401
Proton-coupled zinc antiporter SLC30A1 [OS=Homo sapiens]	0.852	0.438291904
Ubiquitin carboxyl-terminal hydrolase 40 [OS=Homo sapiens]	0.852	0.999928964
Actin-like protein 6A [OS=Homo sapiens]	0.853	4.0514E-06
Inositol monophosphatase 1 [OS=Homo sapiens]	0.853	0.00223251
Ribonuclease H2 subunit A [OS=Homo sapiens]	0.853	0.401171815
Histone deacetylase complex subunit SAP130 [OS=Homo sapiens]	0.853	0.910789503
Nonsense-mediated mRNA decay factor SMG9 [OS=Homo sapiens]	0.853	0.30814366
Tonsoku-like protein [OS=Homo sapiens]	0.853	0.949517848
Centrosomal protein of 192 kDa [OS=Homo sapiens]	0.853	0.86583967
Splicing factor 3B subunit 1 [OS=Homo sapiens]	0.854	0.396964777
Ras GTPase-activating protein-binding protein 1 [OS=Homo sapiens]	0.854	0.060541142
ATP-dependent RNA helicase DHX8 [OS=Homo sapiens]	0.854	0.316167872

Coronin-1A [OS=Homo sapiens]	0.854	0.106709257
Prostaglandin reductase 2 [OS=Homo sapiens]	0.854	0.571234766
Sarcolemmal membrane-associated protein [OS=Homo sapiens]	0.854	0.125576032
Inhibitor of growth protein 1 [OS=Homo sapiens]	0.854	0.562569684
Exosome RNA helicase MTR4 [OS=Homo sapiens]	0.855	0.045850988
RNA polymerase II-associated factor 1 homolog [OS=Homo sapiens]	0.855	0.046046982
Cytokine-like nuclear factor N-PAC [OS=Homo sapiens]	0.855	0.694953097
Aladin [OS=Homo sapiens]	0.855	0.437492416
Box C/D snoRNA protein 1 [OS=Homo sapiens]	0.855	0.014572004
Serine/threonine-protein kinase N3 [OS=Homo sapiens]	0.855	0.107009
BolA-like protein 1 [OS=Homo sapiens]	0.855	0.323402397
Structural maintenance of chromosomes protein 3 [OS=Homo sapiens]	0.856	0.012840902
TP53-binding protein 1 [OS=Homo sapiens]	0.856	0.830397858
RRP12-like protein [OS=Homo sapiens]	0.856	0.649168383
Activity-dependent neuroprotector homeobox protein [OS=Homo sapiens]	0.856	0.821922866
DNA-binding protein RFX5 [OS=Homo sapiens]	0.856	0.005997223
RNA-binding protein 7 [OS=Homo sapiens]	0.856	0.377034562
Sterol O-acyltransferase 1 [OS=Homo sapiens]	0.856	0.43973376
Muskelin [OS=Homo sapiens]	0.856	0.535781838
Glycosyltransferase 8 domain-containing protein 1 [OS=Homo sapiens]	0.856	0.961103271
Titin [OS=Homo sapiens]	0.856	0.819862422
Myosin-10 [OS=Homo sapiens]	0.857	0.064809169
Guanine nucleotide-binding protein G(I)/G(S)/G(T) subunit beta-2 [OS=Homo sa	0.857	0.776218458
Ubiquitin-conjugating enzyme E2 S [OS=Homo sapiens]	0.857	0.01471102
Protein O-mannosyl-transferase 2 [OS=Homo sapiens]	0.857	0.990714683
FAS-associated factor 2 [OS=Homo sapiens]	0.858	0.111559705
Centrosomal protein of 55 kDa [OS=Homo sapiens]	0.858	0.001313689
Chromatin modification-related protein MEAF6 [OS=Homo sapiens]	0.858	0.910433379
E3 ubiquitin-protein ligase TRIP12 [OS=Homo sapiens]	0.859	0.055977279
Leucyl-cystinyl aminopeptidase [OS=Homo sapiens]	0.859	0.268465106
MAP7 domain-containing protein 3 [OS=Homo sapiens]	0.859	0.0378847
Integrator complex subunit 7 [OS=Homo sapiens]	0.859	0.25935617
Formin-binding protein 4 [OS=Homo sapiens]	0.859	0.096314511
Nuclear transcription factor Y subunit alpha [OS=Homo sapiens]	0.859	0.720025596
Charged multivesicular body protein 1b [OS=Homo sapiens]	0.859	0.010342437
Electrogenic aspartate/glutamate antiporter SLC25A13, mitochondrial [OS=Hon	0.86	0.984306711
Transmembrane protein 43 [OS=Homo sapiens]	0.86	0.878907312
Histone PARylation factor 1 [OS=Homo sapiens]	0.86	0.787908255
Pleckstrin homology-like domain family B member 1 [OS=Homo sapiens]	0.86	0.078793534
Oxysterol-binding protein-related protein 3 [OS=Homo sapiens]	0.86	0.16976001
Equilibrative nucleoside transporter 1 [OS=Homo sapiens]	0.86	0.595828612
Small integral membrane protein 20 [OS=Homo sapiens]	0.86	0.887333161
Nascent polypeptide-associated complex subunit alpha-2 [OS=Homo sapiens]	0.86	0.919990955
DNA-binding protein SATB2 [OS=Homo sapiens]	0.86	0.34644275
cAMP-dependent protein kinase type II-alpha regulatory subunit [OS=Homo sapi	0.861	0.002428644
Nuclear receptor corepressor 2 [OS=Homo sapiens]	0.861	0.087526968
RNA-binding protein NOB1 [OS=Homo sapiens]	0.861	0.005607129
Ras-related protein Rab-3B [OS=Homo sapiens]	0.861	0.981497238
Nuclear factor 1 C-type [OS=Homo sapiens]	0.861	0.659293878
Sentrin-specific protease 6 [OS=Homo sapiens]	0.861	0.75438411
Acireductone dioxygenase [OS=Homo sapiens]	0.861	0.191419364
Splicing factor ESS-2 homolog [OS=Homo sapiens]	0.861	0.443860165
Sodium/myo-inositol cotransporter [OS=Homo sapiens]	0.861	0.881053088
1-acyl-sn-glycerol-3-phosphate acyltransferase delta [OS=Homo sapiens]	0.861	0.134279682
Elongation factor G, mitochondrial [OS=Homo sapiens]	0.862	0.167690899
Integrator complex subunit 5 [OS=Homo sapiens]	0.862	0.5324616
Craniofacial development protein 1 [OS=Homo sapiens]	0.863	0.01568834
Serine/threonine-protein phosphatase 2A 56 kDa regulatory subunit gamma isof	0.863	0.989283473
Zinc finger SWIM domain-containing protein 8 [OS=Homo sapiens]	0.863	0.300991503
Serine incorporator 3 [OS=Homo sapiens]	0.863	0.471934747
Protein FAM234A [OS=Homo sapiens]	0.863	0.592235582
Protein PET100 homolog, mitochondrial [OS=Homo sapiens]	0.863	0.696126381
Sin3 histone deacetylase corepressor complex component SDS3 [OS=Homo sap	0.863	0.479487069
Proliferating cell nuclear antigen [OS=Homo sapiens]	0.864	0.002159531
Kinesin-like protein KIFC1 [OS=Homo sapiens]	0.864	0.002269951
Squamous cell carcinoma antigen recognized by T-cells 3 [OS=Homo sapiens]	0.864	0.067776457
Serine/threonine-protein phosphatase 1 regulatory subunit 10 [OS=Homo sapien	0.864	0.369168553
Ribonucleases P/MRP protein subunit POP1 [OS=Homo sapiens]	0.864	0.00166805
Cyclin-H [OS=Homo sapiens]	0.864	0.250544808
Metalloreductase STEAP3 [OS=Homo sapiens]	0.864	0.525795718
Denticleless protein homolog [OS=Homo sapiens]	0.864	0.219823635
Putative heat shock protein HSP 90-alpha A4 [OS=Homo sapiens]	0.865	0.99528563
Telomere length regulation protein TEL2 homolog [OS=Homo sapiens]	0.865	0.724323303
NADH dehydrogenase [ubiquinone] 1 beta subcomplex subunit 1 [OS=Homo sap	0.865	0.90300586
Proline-rich protein 11 [OS=Homo sapiens]	0.865	0.23569807

Helicase SRCAP [OS=Homo sapiens]	0.865	0.990483087
Tyrosine--tRNA ligase, cytoplasmic [OS=Homo sapiens]	0.866	0.001560914
Leucine zipper protein 1 [OS=Homo sapiens]	0.866	0.615255034
Cancer/testis antigen family 45 member A1 [OS=Homo sapiens]	0.866	0.002962909
FMR1-interacting protein NUFIP2 [OS=Homo sapiens]	0.866	0.001328814
Ankycorbin [OS=Homo sapiens]	0.866	0.340404973
Ribosome biogenesis protein SPATA5 [OS=Homo sapiens]	0.866	0.074098622
Probable C-mannosyltransferase DPY19L1 [OS=Homo sapiens]	0.866	0.679971972
Arginine/serine-rich protein PNISR [OS=Homo sapiens]	0.866	0.351043018
Splicing regulator SDE2 [OS=Homo sapiens]	0.866	0.000217512
Solute carrier family 25 member 33 [OS=Homo sapiens]	0.866	0.881033891
Max-like protein X [OS=Homo sapiens]	0.866	0.715302173
Splicing factor 3B subunit 3 [OS=Homo sapiens]	0.867	0.342085495
Nuclear pore complex protein Nup88 [OS=Homo sapiens]	0.867	0.577344779
La-related protein 7 [OS=Homo sapiens]	0.867	0.0092241
Ubiquitin-conjugating enzyme E2 T [OS=Homo sapiens]	0.867	0.024339872
Nuclear receptor coactivator 7 [OS=Homo sapiens]	0.867	0.052199035
Fanconi anemia group G protein [OS=Homo sapiens]	0.867	0.11402312
39S ribosomal protein L23, mitochondrial [OS=Homo sapiens]	0.867	0.053687303
Phosphomannomutase 1 [OS=Homo sapiens]	0.867	1
Multivesicular body subunit 12B [OS=Homo sapiens]	0.867	0.980267501
Spectrin alpha chain, non-erythrocytic 1 [OS=Homo sapiens]	0.868	0.037807179
Syntaxin-binding protein 1 [OS=Homo sapiens]	0.868	0.289311077
Dihydrofolate reductase [OS=Homo sapiens]	0.868	0.045004167
Tyrosine-protein kinase Yes [OS=Homo sapiens]	0.868	0.920087205
Emerin [OS=Homo sapiens]	0.868	0.823253811
Protein Niban 1 [OS=Homo sapiens]	0.868	0.149370172
Guanine nucleotide-binding protein subunit beta-4 [OS=Homo sapiens]	0.868	0.981481239
Protein phosphatase Slingshot homolog 3 [OS=Homo sapiens]	0.868	0.35431588
Multidrug and toxin extrusion protein 1 [OS=Homo sapiens]	0.868	0.671001733
Exosome complex component RRP46 [OS=Homo sapiens]	0.868	0.547741626
Kinesin-like protein KIF18A [OS=Homo sapiens]	0.868	0.33408354
DNA-directed RNA polymerase II subunit RPB7 [OS=Homo sapiens]	0.868	0.838507411
Nidogen-1 [OS=Homo sapiens]	0.868	0.518479127
Transcription elongation regulator 1 [OS=Homo sapiens]	0.869	0.001127624
Protein scribble homolog [OS=Homo sapiens]	0.869	0.529181977
N-alpha-acetyltransferase 50 [OS=Homo sapiens]	0.869	0.833964593
Protein kinase C and casein kinase substrate in neurons protein 3 [OS=Homo sa	0.869	0.001620364
MAP7 domain-containing protein 1 [OS=Homo sapiens]	0.869	0.004875847
Nucleolar and spindle-associated protein 1 [OS=Homo sapiens]	0.869	0.11550115
Transcription and mRNA export factor ENY2 [OS=Homo sapiens]	0.869	0.43574947
Mediator of RNA polymerase II transcription subunit 18 [OS=Homo sapiens]	0.869	0.00706838
AP-3 complex subunit sigma-2 [OS=Homo sapiens]	0.869	0.520959832
Centriole and centriolar satellite protein OFD1 [OS=Homo sapiens]	0.869	0.483724411
E3 ubiquitin-protein ligase RNF14 [OS=Homo sapiens]	0.87	0.83220882
DnaJ homolog subfamily B member 14 [OS=Homo sapiens]	0.87	0.997894583
Nuclear respiratory factor 1 [OS=Homo sapiens]	0.87	0.345045657
4F2 cell-surface antigen heavy chain [OS=Homo sapiens]	0.871	0.511891133
DNA polymerase alpha catalytic subunit [OS=Homo sapiens]	0.871	0.297582556
Carbonyl reductase [NADPH] 3 [OS=Homo sapiens]	0.871	0.617133104
DNA ligase 1 [OS=Homo sapiens]	0.871	0.086662685
A-kinase anchor protein 8-like [OS=Homo sapiens]	0.871	0.675210685
Neural cell adhesion molecule 1 [OS=Homo sapiens]	0.871	0.611367852
F-box only protein 33 [OS=Homo sapiens]	0.871	0.662676665
(3R)-3-hydroxyacyl-CoA dehydrogenase [OS=Homo sapiens]	0.871	0.983826448
Serine/threonine-protein phosphatase PP1-gamma catalytic subunit [OS=Homo	0.872	0.865970673
Polypyrimidine tract-binding protein 1 [OS=Homo sapiens]	0.872	0.361316633
Tyrosine-protein phosphatase non-receptor type 1 [OS=Homo sapiens]	0.872	0.284033816
Ras-associated and pleckstrin homology domains-containing protein 1 [OS=Hor	0.872	0.102034548
Histone chaperone ASF1B [OS=Homo sapiens]	0.872	0.280642646
Protein regulator of cytokinesis 1 [OS=Homo sapiens]	0.872	0.109483124
Ankyrin repeat domain-containing protein 26 [OS=Homo sapiens]	0.872	0.701630126
U6 snRNA-associated Sm-like protein LSm3 [OS=Homo sapiens]	0.872	0.541197066
TBC1 domain family member 25 [OS=Homo sapiens]	0.872	0.999940571
Double-stranded RNA-specific editase B2 [OS=Homo sapiens]	0.872	0.997355586
Homologous-pairing protein 2 homolog [OS=Homo sapiens]	0.872	0.199585918
Sodium-dependent phosphate transporter 1 [OS=Homo sapiens]	0.872	0.840343091
Guanine nucleotide-binding protein G(i) subunit alpha-3 [OS=Homo sapiens]	0.873	0.894914205
Phostensin [OS=Homo sapiens]	0.873	0.590052774
Dual specificity protein kinase TTK [OS=Homo sapiens]	0.873	0.452543288
Acylphosphatase-1 [OS=Homo sapiens]	0.873	0.051055898
Eukaryotic translation initiation factor 2 subunit 2 [OS=Homo sapiens]	0.874	0.018678363
Pre-mRNA-splicing factor SYF1 [OS=Homo sapiens]	0.874	0.177937957
Glucocorticoid receptor [OS=Homo sapiens]	0.874	0.015462645
DNA primase small subunit [OS=Homo sapiens]	0.874	0.014938528

Protein FAM162A [OS=Homo sapiens]	0.874	0.855686113
E3 ubiquitin-protein ligase RING1 [OS=Homo sapiens]	0.874	0.989715932
Exonuclease 3'-5' domain-containing protein 2 [OS=Homo sapiens]	0.874	0.849031801
Exosome complex component RRP41 [OS=Homo sapiens]	0.874	0.445453314
Transcriptional adapter 3 [OS=Homo sapiens]	0.874	0.074039064
Coiled-coil domain-containing protein 134 [OS=Homo sapiens]	0.874	0.850748259
Activin receptor type-2A [OS=Homo sapiens]	0.874	0.994893658
Small nuclear ribonucleoprotein Sm D2 [OS=Homo sapiens]	0.875	0.665065013
Lysine-specific demethylase 2A [OS=Homo sapiens]	0.875	0.591660316
Integrator complex subunit 6 [OS=Homo sapiens]	0.875	0.265413829
DnaJ homolog subfamily B member 6 [OS=Homo sapiens]	0.875	0.017157184
Cytochrome b-c1 complex subunit 9 [OS=Homo sapiens]	0.875	0.952211376
Aminoacyl tRNA synthase complex-interacting multifunctional protein 1 [OS=Hc	0.876	0.001397969
Zinc finger C3HC-type protein 1 [OS=Homo sapiens]	0.876	0.296242088
Mitochondrial glutamate carrier 1 [OS=Homo sapiens]	0.876	0.951979205
Glucocorticoid modulatory element-binding protein 2 [OS=Homo sapiens]	0.876	0.02401147
NEDD4-like E3 ubiquitin-protein ligase WWP2 [OS=Homo sapiens]	0.876	0.999974522
Bifunctional epoxide hydrolase 2 [OS=Homo sapiens]	0.876	0.999704873
Palmitoyltransferase ZDHHC5 [OS=Homo sapiens]	0.876	0.912617329
GrpE protein homolog 2, mitochondrial [OS=Homo sapiens]	0.876	0.999955624
La-related protein 4 [OS=Homo sapiens]	0.877	0.002209176
Serine/arginine-rich splicing factor 6 [OS=Homo sapiens]	0.877	0.30968095
Proteolipid protein 2 [OS=Homo sapiens]	0.877	0.553315463
DNA-directed RNA polymerase I subunit RPA2 [OS=Homo sapiens]	0.877	0.891760867
Myocyte-specific enhancer factor 2D [OS=Homo sapiens]	0.877	0.413030101
DNA topoisomerase 1 [OS=Homo sapiens]	0.878	0.99888147
Heterogeneous nuclear ribonucleoprotein F [OS=Homo sapiens]	0.878	0.402458699
Splicing factor 3A subunit 2 [OS=Homo sapiens]	0.878	0.430493689
Gem-associated protein 4 [OS=Homo sapiens]	0.878	0.224729442
Ethanolamine-phosphate cytidylyltransferase [OS=Homo sapiens]	0.878	0.743652726
SKI8 subunit of superkiller complex protein [OS=Homo sapiens]	0.878	0.596053542
Vacuolar protein sorting-associated protein 72 homolog [OS=Homo sapiens]	0.878	0.838291041
Translation machinery-associated protein 16 [OS=Homo sapiens]	0.878	0.134531613
Small nuclear ribonucleoprotein Sm D1 [OS=Homo sapiens]	0.878	0.691666183
Smad nuclear-interacting protein 1 [OS=Homo sapiens]	0.878	0.834200138
Guanine nucleotide exchange factor VAV2 [OS=Homo sapiens]	0.879	0.090461445
mRNA-capping enzyme [OS=Homo sapiens]	0.879	0.806894658
U6 snRNA-associated Sm-like protein LSm8 [OS=Homo sapiens]	0.879	0.077713122
Ubiquitin carboxyl-terminal hydrolase 13 [OS=Homo sapiens]	0.88	0.712516071
U1 small nuclear ribonucleoprotein A [OS=Homo sapiens]	0.88	0.462104415
Peptidyl-tRNA hydrolase ICT1, mitochondrial [OS=Homo sapiens]	0.88	0.538964847
Histone-lysine N-methyltransferase SETD7 [OS=Homo sapiens]	0.88	0.026221384
Ankyrin repeat and SAM domain-containing protein 6 [OS=Homo sapiens]	0.88	0.366002969
Frataxin, mitochondrial [OS=Homo sapiens]	0.88	0.685804001
Afadin- and alpha-actinin-binding protein [OS=Homo sapiens]	0.88	0.895668229
Actin-related protein 5 [OS=Homo sapiens]	0.88	0.999977729
Mitochondrial import receptor subunit TOM34 [OS=Homo sapiens]	0.881	0.05043254
tRNA (guanine(6)-N2)-methyltransferase THUMP3 [OS=Homo sapiens]	0.881	0.101805375
Junctional cadherin 5-associated protein [OS=Homo sapiens]	0.881	0.995180556
Protein lin-7 homolog C [OS=Homo sapiens]	0.881	0.471619506
Cyanocobalamin reductase / alkylcobalamin dealkylase [OS=Homo sapiens]	0.881	0.368641555
ATP-dependent DNA helicase Q5 [OS=Homo sapiens]	0.881	0.751387874
Cullin-4A [OS=Homo sapiens]	0.882	0.12457391
Golgin subfamily A member 2 [OS=Homo sapiens]	0.882	0.982679976
High mobility group protein HMG-I/HMG-Y [OS=Homo sapiens]	0.882	0.999805509
Galectin-1 [OS=Homo sapiens]	0.882	0.110217755
Zinc finger protein 609 [OS=Homo sapiens]	0.882	0.54236951
Ribosome biogenesis protein SPATA5L1 [OS=Homo sapiens]	0.882	0.928110106
Nonsense-mediated mRNA decay factor SMG7 [OS=Homo sapiens]	0.882	0.893123747
Coiled-coil domain-containing protein 127 [OS=Homo sapiens]	0.882	0.986808564
Coiled-coil domain-containing protein 82 [OS=Homo sapiens]	0.882	0.22334634
Pentatricopeptide repeat-containing protein 2, mitochondrial [OS=Homo sapien	0.882	0.9352775
Desmoplakin [OS=Homo sapiens]	0.883	0.033305976
Tubulin alpha-1A chain [OS=Homo sapiens]	0.883	0.816116781
NAD(P)H dehydrogenase [quinone] 1 [OS=Homo sapiens]	0.883	0.769498183
Telomerase Cajal body protein 1 [OS=Homo sapiens]	0.883	0.000899504
Methyltransferase-like protein 17, mitochondrial [OS=Homo sapiens]	0.883	0.023570672
IQCJ-SCHIP1 readthrough transcript protein [OS=Homo sapiens]	0.883	0.878526075
Nonsense-mediated mRNA decay factor SMG5 [OS=Homo sapiens]	0.883	0.999993115
Beta-actin-like protein 2 [OS=Homo sapiens]	0.884	0.648175956
KN motif and ankyrin repeat domain-containing protein 2 [OS=Homo sapiens]	0.884	0.02221953
Activating signal cointegrator 1 complex subunit 2 [OS=Homo sapiens]	0.884	0.999714688
Histone deacetylase complex subunit SAP30 [OS=Homo sapiens]	0.884	0.069491733
THO complex subunit 6 homolog [OS=Homo sapiens]	0.884	0.241665869
Cyclin-L2 [OS=Homo sapiens]	0.884	0.931696371

Rho guanine nucleotide exchange factor 17 [OS=Homo sapiens]	0.884	0.9658563
Transcription termination factor 2 [OS=Homo sapiens]	0.885	0.090728453
Probable ATP-dependent RNA helicase DHX37 [OS=Homo sapiens]	0.885	0.108481605
E1A-binding protein p400 [OS=Homo sapiens]	0.885	0.879326776
Geminin [OS=Homo sapiens]	0.885	0.991614355
ATP-binding cassette sub-family C member 4 [OS=Homo sapiens]	0.885	0.847572439
G-protein coupled receptor-associated protein LMBRD2 [OS=Homo sapiens]	0.885	0.893489732
Zinc finger protein with KRAB and SCAN domains 8 [OS=Homo sapiens]	0.885	0.324815929
Cyclin-dependent kinase 4 inhibitor C [OS=Homo sapiens]	0.885	0.688833975
Acetyl-CoA acetyltransferase, cytosolic [OS=Homo sapiens]	0.886	0.962178886
Casein kinase II subunit alpha' [OS=Homo sapiens]	0.886	0.012273482
Flap endonuclease 1 [OS=Homo sapiens]	0.886	0.019235722
Elongator complex protein 4 [OS=Homo sapiens]	0.886	0.319944551
Serine/threonine-protein kinase RIO1 [OS=Homo sapiens]	0.886	0.594433057
Phosphopentomutase [OS=Homo sapiens]	0.886	0.619473383
Transcription intermediary factor 1-alpha [OS=Homo sapiens]	0.886	0.143058141
Aryl hydrocarbon receptor nuclear translocator [OS=Homo sapiens]	0.886	0.255634497
UPF0488 protein C8orf33 [OS=Homo sapiens]	0.886	0.577732097
Carbonic anhydrase-related protein [OS=Homo sapiens]	0.886	0.170188055
Mitogen-activated protein kinase kinase kinase 4 [OS=Homo sapiens]	0.886	0.944810569
AFG3-like protein 2 [OS=Homo sapiens]	0.887	0.791478793
ADP/ATP translocase 3 [OS=Homo sapiens]	0.887	0.67803998
Origin recognition complex subunit 3 [OS=Homo sapiens]	0.887	0.0282451
CTP synthase 2 [OS=Homo sapiens]	0.887	0.823685227
NADH dehydrogenase [ubiquinone] 1 alpha subcomplex assembly factor 4 [OS=	0.887	0.623837783
Serine/threonine-protein phosphatase 6 regulatory ankyrin repeat subunit C [OS	0.887	0.608501026
ER membrane protein complex subunit 7 [OS=Homo sapiens]	0.887	0.796110195
Sodium-coupled neutral amino acid symporter 1 [OS=Homo sapiens]	0.887	0.512120119
RILP-like protein 1 [OS=Homo sapiens]	0.887	0.908082605
Cytochrome b-c1 complex subunit 8 [OS=Homo sapiens]	0.887	0.985217789
Protein FAM241B [OS=Homo sapiens]	0.887	0.90967694
Alpha-1,6-mannosyl-glycoprotein 2-beta-N-acetylglucosaminyltransferase [OS=	0.887	0.965321337
Phosphoenolpyruvate carboxykinase [GTP], mitochondrial [OS=Homo sapiens]	0.888	0.136403313
Hyaluronan mediated motility receptor [OS=Homo sapiens]	0.888	0.188021308
E3 ubiquitin-protein ligase ARIH1 [OS=Homo sapiens]	0.888	0.162990773
Condensin-2 complex subunit H2 [OS=Homo sapiens]	0.888	0.963945355
Lymphocyte function-associated antigen 3 [OS=Homo sapiens]	0.888	0.924525139
Zinc finger CCHC domain-containing protein 8 [OS=Homo sapiens]	0.889	0.499050548
Transgelin [OS=Homo sapiens]	0.889	0.561880447
CGG triplet repeat-binding protein 1 [OS=Homo sapiens]	0.889	0.226765571
Methionine synthase [OS=Homo sapiens]	0.889	0.529683191
UPF0688 protein C1orf174 [OS=Homo sapiens]	0.889	0.885347555
Zinc finger protein AEBP2 [OS=Homo sapiens]	0.889	0.877801869
Treacle protein [OS=Homo sapiens]	0.89	0.176728066
Replication factor C subunit 5 [OS=Homo sapiens]	0.89	0.933241532
Sideroflexin-4 [OS=Homo sapiens]	0.89	0.955837663
NAD-dependent protein deacetylase sirtuin-2 [OS=Homo sapiens]	0.89	0.17229239
MAP kinase-activated protein kinase 2 [OS=Homo sapiens]	0.89	0.462237023
Mitotic spindle assembly checkpoint protein MAD2A [OS=Homo sapiens]	0.89	0.479142899
bMERB domain-containing protein 1 [OS=Homo sapiens]	0.89	0.996778213
Non-POU domain-containing octamer-binding protein [OS=Homo sapiens]	0.891	0.388346256
Pseudouridylate synthase 1 homolog [OS=Homo sapiens]	0.891	0.679367543
LEM domain-containing protein 2 [OS=Homo sapiens]	0.891	0.994700249
CXXC motif containing zinc binding protein [OS=Homo sapiens]	0.891	0.694036058
Phosphatase and actin regulator 4 [OS=Homo sapiens]	0.891	0.064548157
Torsin-3A [OS=Homo sapiens]	0.891	0.998601307
DNA-directed RNA polymerase II subunit RPB4 [OS=Homo sapiens]	0.891	0.84570608
Protein SCO1 homolog, mitochondrial [OS=Homo sapiens]	0.891	0.505795037
Alpha-catulin [OS=Homo sapiens]	0.891	0.719023453
Transcriptional repressor NF-X1 [OS=Homo sapiens]	0.891	0.993759794
Replication factor C subunit 2 [OS=Homo sapiens]	0.892	0.59246111
Phosphatidylinositol 5-phosphate 4-kinase type-2 beta [OS=Homo sapiens]	0.892	0.912704521
Double-stranded RNA-binding protein Staufen homolog 2 [OS=Homo sapiens]	0.892	0.09176284
Heparan sulfate 2-O-sulfotransferase 1 [OS=Homo sapiens]	0.892	0.952595044
Kinesin-like protein KIF21B [OS=Homo sapiens]	0.892	0.975025191
LIM domain-containing protein ajuba [OS=Homo sapiens]	0.892	0.06194635
Cell cycle checkpoint control protein RAD9A [OS=Homo sapiens]	0.892	0.999930903
Uracil-DNA glycosylase [OS=Homo sapiens]	0.892	0.992411266
Zinc finger protein 556 [OS=Homo sapiens]	0.892	0.278554341
ATP synthase subunit C lysine N-methyltransferase [OS=Homo sapiens]	0.892	0.999736522
DNA (cytosine-5)-methyltransferase 1 [OS=Homo sapiens]	0.893	0.054259731
Afadin [OS=Homo sapiens]	0.893	0.003626116
Non-histone chromosomal protein HMG-14 [OS=Homo sapiens]	0.893	0.950053424
Calcium-binding and coiled-coil domain-containing protein 2 [OS=Homo sapien	0.893	0.410348206
BLOC-2 complex member HPS3 [OS=Homo sapiens]	0.893	0.999657929

Serine/threonine-protein kinase ULK3 [OS=Homo sapiens]	0.893	0.731723194
Zinc transporter 7 [OS=Homo sapiens]	0.893	0.999999992
Butyrophilin subfamily 2 member A1 [OS=Homo sapiens]	0.893	0.663782418
Small subunit processome component 20 homolog [OS=Homo sapiens]	0.894	0.994066827
Rho GTPase-activating protein 1 [OS=Homo sapiens]	0.894	0.32794284
ATPase MORC2 [OS=Homo sapiens]	0.894	0.02346688
FAS-associated factor 1 [OS=Homo sapiens]	0.894	0.553144161
Transmembrane protein 115 [OS=Homo sapiens]	0.894	0.845141191
Protein CMSS1 [OS=Homo sapiens]	0.894	0.892650372
Protein MAK16 homolog [OS=Homo sapiens]	0.894	0.988778768
RecQ-mediated genome instability protein 1 [OS=Homo sapiens]	0.894	0.760959914
Transmembrane channel-like protein 1 [OS=Homo sapiens]	0.894	0.995341315
ETS domain-containing transcription factor ERF [OS=Homo sapiens]	0.894	0.998257611
Probable cysteine--tRNA ligase, mitochondrial [OS=Homo sapiens]	0.894	0.545929889
Rho GTPase-activating protein 10 [OS=Homo sapiens]	0.894	0.998241978
BRI3-binding protein [OS=Homo sapiens]	0.894	0.986609391
Importin subunit alpha-3 [OS=Homo sapiens]	0.895	0.797324478
Histone deacetylase 3 [OS=Homo sapiens]	0.895	0.961831826
Protein Mis18-beta [OS=Homo sapiens]	0.895	0.708444321
Protein N-lysine methyltransferase METTL21D [OS=Homo sapiens]	0.895	0.987125169
Heterogeneous nuclear ribonucleoprotein K [OS=Homo sapiens]	0.896	0.012692695
Nucleophosmin [OS=Homo sapiens]	0.896	0.589310498
Nucleoporin SEH1 [OS=Homo sapiens]	0.896	0.685162722
Very-long-chain enoyl-CoA reductase [OS=Homo sapiens]	0.896	0.629091332
Zinc finger protein 330 [OS=Homo sapiens]	0.896	0.685855566
ER membrane protein complex subunit 5 [OS=Homo sapiens]	0.896	0.742336077
Coiled-coil domain-containing protein 102A [OS=Homo sapiens]	0.896	0.071007753
tRNA wybutosine-synthesizing protein 5 [OS=Homo sapiens]	0.896	0.973133197
Protein-S-isoprenylcysteine O-methyltransferase [OS=Homo sapiens]	0.896	0.933924479
Mirror-image polydactyly gene 1 protein [OS=Homo sapiens]	0.896	0.999999982
Thyroid transcription factor 1-associated protein 26 [OS=Homo sapiens]	0.896	0.49976121
39S ribosomal protein L47, mitochondrial [OS=Homo sapiens]	0.897	0.638213234
Mediator of RNA polymerase II transcription subunit 23 [OS=Homo sapiens]	0.897	0.999993677
Proline-rich protein 14 [OS=Homo sapiens]	0.897	0.762064546
Polyamine-modulated factor 1 [OS=Homo sapiens]	0.897	0.009451282
Spindlin-1 [OS=Homo sapiens]	0.897	0.330103037
G patch domain-containing protein 4 [OS=Homo sapiens]	0.897	0.314321857
THAP domain-containing protein 5 [OS=Homo sapiens]	0.897	0.24745965
Tastin [OS=Homo sapiens]	0.897	0.976755288
Protein CDV3 homolog [OS=Homo sapiens]	0.898	0.673016528
Ribosomal RNA small subunit methyltransferase NEP1 [OS=Homo sapiens]	0.898	0.817089553
AT-rich interactive domain-containing protein 2 [OS=Homo sapiens]	0.898	0.027321311
Ribonuclease P protein subunit p38 [OS=Homo sapiens]	0.898	0.151056377
Mitochondrial fission regulator 1 [OS=Homo sapiens]	0.898	0.926458019
E3 ubiquitin-protein ligase PPP1R11 [OS=Homo sapiens]	0.898	0.555191939
Transcription factor SOX-9 [OS=Homo sapiens]	0.898	0.677210962
NADH-ubiquinone oxidoreductase chain 5 [OS=Homo sapiens]	0.898	0.72060135
Alpha-taxilin [OS=Homo sapiens]	0.899	0.033800734
Putative RNA-binding protein Luc7-like 2 [OS=Homo sapiens]	0.899	0.002191606
Cyclin-dependent kinase 1 [OS=Homo sapiens]	0.899	0.093864593
Small acidic protein [OS=Homo sapiens]	0.899	0.089903763
5'-3' exoribonuclease 2 [OS=Homo sapiens]	0.899	0.015399742
Pre-mRNA-splicing factor ISY1 homolog [OS=Homo sapiens]	0.899	0.83102659
U1 small nuclear ribonucleoprotein C [OS=Homo sapiens]	0.899	0.512862814
RelA-associated inhibitor [OS=Homo sapiens]	0.899	0.404050615
Coiled-coil domain-containing protein 90B, mitochondrial [OS=Homo sapiens]	0.899	0.832777339
N-alpha-acetyltransferase 40 [OS=Homo sapiens]	0.899	0.550792738
Lysine-specific demethylase 4B [OS=Homo sapiens]	0.899	0.264884133
Contactin-3 [OS=Homo sapiens]	0.899	0.714869616
Spectrin beta chain, non-erythrocytic 1 [OS=Homo sapiens]	0.9	0.160756936
mRNA export factor RAE1 [OS=Homo sapiens]	0.9	0.69508373
Zinc finger protein 318 [OS=Homo sapiens]	0.9	0.940750384
WD repeat-containing protein 26 [OS=Homo sapiens]	0.9	0.26966083
Scavenger receptor class B member 1 [OS=Homo sapiens]	0.9	0.791201004
Myb-binding protein 1A [OS=Homo sapiens]	0.901	0.779370404
Death-inducer obliterator 1 [OS=Homo sapiens]	0.901	0.817784506
Signal transducer and activator of transcription 5B [OS=Homo sapiens]	0.901	0.775907331
Cyclin-dependent kinase 2 [OS=Homo sapiens]	0.901	0.012538533
DNA-directed RNA polymerase II subunit RPB3 [OS=Homo sapiens]	0.901	0.477594037
FSD1-like protein [OS=Homo sapiens]	0.901	1
Autophagy-related protein 2 homolog B [OS=Homo sapiens]	0.901	0.999638284
Protein JTB [OS=Homo sapiens]	0.901	0.959098645
Mediator of RNA polymerase II transcription subunit 27 [OS=Homo sapiens]	0.902	0.884792343
CXXC-type zinc finger protein 1 [OS=Homo sapiens]	0.902	0.796365481
Unconventional myosin-Va [OS=Homo sapiens]	0.903	0.68111677

Serine/threonine-protein kinase PAK 4 [OS=Homo sapiens]	0.903	0.200752217
39S ribosomal protein L41, mitochondrial [OS=Homo sapiens]	0.903	0.404100032
Polycomb group RING finger protein 5 [OS=Homo sapiens]	0.903	0.98533105
Protein lifeguard 4 [OS=Homo sapiens]	0.903	0.996434849
Spindle and centriole-associated protein 1 [OS=Homo sapiens]	0.903	0.326504696
Ribonuclease P protein subunit p20 [OS=Homo sapiens]	0.903	0.661302358
Heterogeneous nuclear ribonucleoprotein Q [OS=Homo sapiens]	0.904	0.18318552
Small nuclear ribonucleoprotein E [OS=Homo sapiens]	0.904	0.905463626
Diphosphoinositol polyphosphate phosphohydrolase 1 [OS=Homo sapiens]	0.904	0.706776446
Methylthioribulose-1-phosphate dehydratase [OS=Homo sapiens]	0.904	0.916871206
Nucleosome-remodeling factor subunit BPTF [OS=Homo sapiens]	0.904	0.998053272
Mitochondrial import inner membrane translocase subunit Tim10 [OS=Homo sa	0.904	0.558850123
E3 ubiquitin-protein ligase MARCHF6 [OS=Homo sapiens]	0.904	0.999999791
MAPK regulated corepressor interacting protein 2 [OS=Homo sapiens]	0.904	0.919524357
Homeobox protein SIX5 [OS=Homo sapiens]	0.904	0.637877142
Integrator complex subunit 2 [OS=Homo sapiens]	0.904	0.905391399
Tumor necrosis factor receptor superfamily member 10B [OS=Homo sapiens]	0.904	0.961275007
28S ribosomal protein S27, mitochondrial [OS=Homo sapiens]	0.905	0.135808746
Sperm-associated antigen 5 [OS=Homo sapiens]	0.905	0.108202595
Density-regulated protein [OS=Homo sapiens]	0.905	0.226773712
Abasic site processing protein HMCES [OS=Homo sapiens]	0.905	0.199464665
COP9 signalosome complex subunit 7b [OS=Homo sapiens]	0.905	0.4037009
Mitochondrial tRNA-specific 2-thiouridylase 1 [OS=Homo sapiens]	0.905	0.277135496
Integrator complex subunit 12 [OS=Homo sapiens]	0.905	0.901285018
Kin of IRRE-like protein 1 [OS=Homo sapiens]	0.905	0.998763033
PC4 and SFRS1-interacting protein [OS=Homo sapiens]	0.906	0.773036345
WD repeat and HMG-box DNA-binding protein 1 [OS=Homo sapiens]	0.906	0.63223754
Luc7-like protein 3 [OS=Homo sapiens]	0.906	0.638035233
RNA-binding protein PNO1 [OS=Homo sapiens]	0.906	0.057480947
DnaJ homolog subfamily C member 11 [OS=Homo sapiens]	0.906	0.836827957
Myc-associated zinc finger protein [OS=Homo sapiens]	0.906	0.105351459
Histone deacetylase 7 [OS=Homo sapiens]	0.906	1
CCR4-NOT transcription complex subunit 4 [OS=Homo sapiens]	0.906	0.999999994
Motile sperm domain-containing protein 1 [OS=Homo sapiens]	0.906	0.999999345
G patch domain-containing protein 11 [OS=Homo sapiens]	0.906	0.99901599
Putative ATP-dependent RNA helicase TDRD12 [OS=Homo sapiens]	0.907	0.443337549
Unconventional myosin-VI [OS=Homo sapiens]	0.907	0.040289529
Ubiquilin-2 [OS=Homo sapiens]	0.907	0.982827212
cAMP-dependent protein kinase type I-alpha regulatory subunit [OS=Homo sapi	0.907	0.409007251
V-type proton ATPase subunit d 1 [OS=Homo sapiens]	0.907	0.945494555
Ribosomal oxygenase 2 [OS=Homo sapiens]	0.907	0.160333454
Ribonucleoside-diphosphate reductase subunit M2 B [OS=Homo sapiens]	0.907	0.248588655
NADH-cytochrome b5 reductase 2 [OS=Homo sapiens]	0.907	0.647914288
SAGA-associated factor 29 [OS=Homo sapiens]	0.907	0.737514032
E3 ubiquitin-protein ligase RNF126 [OS=Homo sapiens]	0.907	0.706667208
Major prion protein [OS=Homo sapiens]	0.907	0.875621951
Kinetochore-associated protein 1 [OS=Homo sapiens]	0.908	0.423895156
Biorientation of chromosomes in cell division protein 1-like 1 [OS=Homo sapiens	0.908	0.992318039
Acyl-CoA-binding domain-containing protein 5 [OS=Homo sapiens]	0.908	0.84117574
tRNA-splicing endonuclease subunit Sen34 [OS=Homo sapiens]	0.908	0.664445184
Required for meiotic nuclear division protein 1 homolog [OS=Homo sapiens]	0.908	0.838086936
CD44 antigen [OS=Homo sapiens]	0.909	0.505879404
Protein mago nashi homolog 2 [OS=Homo sapiens]	0.909	0.442663992
Guanine nucleotide-binding protein subunit alpha-13 [OS=Homo sapiens]	0.909	0.93179219
Exosome complex component RRP43 [OS=Homo sapiens]	0.909	0.48606954
EPM2A-interacting protein 1 [OS=Homo sapiens]	0.909	0.993275759
Mitotic deacetylase-associated SANT domain protein [OS=Homo sapiens]	0.909	0.996915925
DNA-directed RNA polymerases I, II, and III subunit RPABC5 [OS=Homo sapiens]	0.909	0.22619679
Peroxisomal membrane protein 4 [OS=Homo sapiens]	0.909	0.885053275
Glycoprotein-N-acetylgalactosamine 3-beta-galactosyltransferase 1 [OS=Homo	0.909	0.910967324
Probable methyltransferase TARBP1 [OS=Homo sapiens]	0.909	0.660124813
Riboflavin kinase [OS=Homo sapiens]	0.909	0.999992417
Nuclear transcription factor Y subunit beta [OS=Homo sapiens]	0.909	0.814477064
Two pore channel protein 1 [OS=Homo sapiens]	0.909	0.926750171
Heat shock 70 kDa protein 4L [OS=Homo sapiens]	0.91	0.092009551
Mitotic spindle assembly checkpoint protein MAD1 [OS=Homo sapiens]	0.91	0.107991891
4-hydroxyphenylpyruvate dioxygenase-like protein [OS=Homo sapiens]	0.91	0.778354123
Protein FAM83D [OS=Homo sapiens]	0.91	0.213763431
Forkhead box protein K1 [OS=Homo sapiens]	0.91	0.23645228
Replication factor C subunit 1 [OS=Homo sapiens]	0.91	0.348059546
DNA polymerase delta subunit 2 [OS=Homo sapiens]	0.91	0.999929959
Na(+)/H(+) exchange regulatory cofactor NHE-RF2 [OS=Homo sapiens]	0.91	0.901683842
18S rRNA aminocarboxypropyltransferase [OS=Homo sapiens]	0.91	0.956495667
Cyclin-dependent kinase 7 [OS=Homo sapiens]	0.91	0.546321205
Biogenesis of lysosome-related organelles complex 1 subunit 4 [OS=Homo sapi	0.91	1

BRCA1-A complex subunit RAP80 [OS=Homo sapiens]	0.91	0.811743563
Thrombospondin type-1 domain-containing protein 1 [OS=Homo sapiens]	0.91	0.998608524
General transcription factor 3C polypeptide 1 [OS=Homo sapiens]	0.911	0.065247775
Mitochondrial proton/calcium exchanger protein [OS=Homo sapiens]	0.911	0.650479233
Ras-related protein R-Ras [OS=Homo sapiens]	0.911	0.842668372
Zinc finger MYM-type protein 2 [OS=Homo sapiens]	0.911	0.977939265
RNA-binding protein 8A [OS=Homo sapiens]	0.911	0.316193374
Myc box-dependent-interacting protein 1 [OS=Homo sapiens]	0.911	0.85415016
M-phase inducer phosphatase 3 [OS=Homo sapiens]	0.911	0.198358352
Ribonuclease P protein subunit p25 [OS=Homo sapiens]	0.911	0.423824803
NOP protein chaperone 1 [OS=Homo sapiens]	0.911	0.549645978
WD repeat-containing protein 73 [OS=Homo sapiens]	0.911	0.746401638
Vasorin [OS=Homo sapiens]	0.911	0.996459467
Transmembrane protein 94 [OS=Homo sapiens]	0.911	0.99997003
Vacuolar protein sorting-associated protein 37C [OS=Homo sapiens]	0.911	0.631708469
FMR1-interacting protein NUFIP1 [OS=Homo sapiens]	0.911	0.880910666
Kinesin-like protein KIF11 [OS=Homo sapiens]	0.912	0.133036975
Ribosomal protein S6 kinase alpha-5 [OS=Homo sapiens]	0.912	0.998086912
ATP-binding cassette sub-family D member 1 [OS=Homo sapiens]	0.912	0.692092115
AP2-associated protein kinase 1 [OS=Homo sapiens]	0.912	0.605970066
Cdc42 effector protein 1 [OS=Homo sapiens]	0.912	0.653446308
Protein SDA1 homolog [OS=Homo sapiens]	0.912	0.679715431
Dephospho-CoA kinase domain-containing protein [OS=Homo sapiens]	0.912	0.314392083
E3 ubiquitin-protein ligase MIB1 [OS=Homo sapiens]	0.912	0.987577594
Chromosome transmission fidelity protein 8 homolog [OS=Homo sapiens]	0.912	0.485875848
Mediator of RNA polymerase II transcription subunit 7 [OS=Homo sapiens]	0.912	0.890668461
Zinc finger protein 346 [OS=Homo sapiens]	0.912	0.998019114
Transmembrane protein 51 [OS=Homo sapiens]	0.912	0.957500075
Nuclear nucleic acid-binding protein C1D [OS=Homo sapiens]	0.912	0.86305277
Son of sevenless homolog 1 [OS=Homo sapiens]	0.912	0.948536801
Heat shock protein HSP 90-alpha [OS=Homo sapiens]	0.913	0.464128424
Aspartate aminotransferase, cytoplasmic [OS=Homo sapiens]	0.913	0.141606532
TBC1 domain family member 5 [OS=Homo sapiens]	0.913	0.201950122
Deoxyuridine 5'-triphosphate nucleotidohydrolase, mitochondrial [OS=Homo sa	0.913	0.906952064
Ras GTPase-activating-like protein IQGAP3 [OS=Homo sapiens]	0.913	0.424793357
Integrator complex subunit 4 [OS=Homo sapiens]	0.913	0.144954949
Proto-oncogene tyrosine-protein kinase Src [OS=Homo sapiens]	0.913	0.962306776
Small nuclear ribonucleoprotein Sm D3 [OS=Homo sapiens]	0.913	0.491992669
General transcription factor IIH subunit 2 [OS=Homo sapiens]	0.913	0.999641927
Iron-sulfur cluster assembly 1 homolog, mitochondrial [OS=Homo sapiens]	0.913	0.939049447
Histone acetyltransferase KAT2A [OS=Homo sapiens]	0.914	0.335924483
Pyrroline-5-carboxylate reductase 3 [OS=Homo sapiens]	0.914	0.9995217
CDKN2A-interacting protein [OS=Homo sapiens]	0.914	0.912859564
Leucine-rich repeat-containing protein 42 [OS=Homo sapiens]	0.914	0.192155277
Pogo transposable element with KRAB domain [OS=Homo sapiens]	0.914	0.999736886
Transport and Golgi organization protein 6 homolog [OS=Homo sapiens]	0.914	1
EKC/KEOPS complex subunit GON7 [OS=Homo sapiens]	0.914	0.7915321
Zinc finger CCHC domain-containing protein 9 [OS=Homo sapiens]	0.914	0.948814266
Golgi apparatus protein 1 [OS=Homo sapiens]	0.915	0.949123667
Nuclear pore complex protein Nup214 [OS=Homo sapiens]	0.915	0.653630287
Transformation/transcription domain-associated protein [OS=Homo sapiens]	0.915	0.887637765
Erlin-1 [OS=Homo sapiens]	0.915	0.986261213
NudC domain-containing protein 3 [OS=Homo sapiens]	0.915	0.3184828
T-complex protein 11-like protein 1 [OS=Homo sapiens]	0.915	0.961259343
Protein LZIC [OS=Homo sapiens]	0.915	0.701766965
Exosome complex component RRP45 [OS=Homo sapiens]	0.915	0.496605593
Beta-1,4-galactosyltransferase 1 [OS=Homo sapiens]	0.915	0.232539641
Histone deacetylase 4 [OS=Homo sapiens]	0.915	0.999899545
DNA-directed RNA polymerases I, II, and III subunit RPABC2 [OS=Homo sapiens]	0.915	0.998559481
Protein LIAT1 [OS=Homo sapiens]	0.915	0.971431586
TRAF3-interacting protein 1 [OS=Homo sapiens]	0.915	0.676510002
Protein-glutamine gamma-glutamyltransferase 2 [OS=Homo sapiens]	0.916	0.395866516
Farnesyl pyrophosphate synthase [OS=Homo sapiens]	0.916	0.024568309
Chromobox protein homolog 1 [OS=Homo sapiens]	0.916	0.985859659
Codanin-1 [OS=Homo sapiens]	0.916	0.636859782
TNF receptor-associated factor 2 [OS=Homo sapiens]	0.916	0.81136333
Tetratricopeptide repeat protein 19, mitochondrial [OS=Homo sapiens]	0.916	0.987983365
Zinc finger protein 24 [OS=Homo sapiens]	0.916	0.584492129
Transmembrane protein 33 [OS=Homo sapiens]	0.916	0.999976588
Patatin-like phospholipase domain-containing protein 2 [OS=Homo sapiens]	0.916	0.834467677
KICSTOR complex protein ITFG2 [OS=Homo sapiens]	0.916	0.998825793
Schlafen family member 11 [OS=Homo sapiens]	0.916	0.81632628
Mesoderm induction early response protein 3 [OS=Homo sapiens]	0.916	0.997454412
Protein phosphatase 1 regulatory subunit 12A [OS=Homo sapiens]	0.917	0.306493497
Hydroxymethylglutaryl-CoA synthase, cytoplasmic [OS=Homo sapiens]	0.917	0.044569897

Large subunit GTPase 1 homolog [OS=Homo sapiens]	0.917	0.04854666
Armadillo repeat-containing protein 6 [OS=Homo sapiens]	0.917	0.283472954
DNA polymerase epsilon catalytic subunit A [OS=Homo sapiens]	0.917	0.251435005
14-3-3 protein sigma [OS=Homo sapiens]	0.917	0.573304313
Ribonuclease P protein subunit p30 [OS=Homo sapiens]	0.917	0.019206233
Protein zwilch homolog [OS=Homo sapiens]	0.917	0.801677253
DCN1-like protein 5 [OS=Homo sapiens]	0.917	0.999699848
Zinc finger protein 691 [OS=Homo sapiens]	0.917	0.591902593
COX assembly mitochondrial protein homolog [OS=Homo sapiens]	0.917	0.830305403
Deubiquitinase OTUD6B [OS=Homo sapiens]	0.917	0.278223323
Transcriptional-regulating factor 1 [OS=Homo sapiens]	0.917	0.990078682
Inactive Ufm1-specific protease 1 [OS=Homo sapiens]	0.917	0.850831499
Heterogeneous nuclear ribonucleoprotein U [OS=Homo sapiens]	0.918	0.767541808
STE20-like serine/threonine-protein kinase [OS=Homo sapiens]	0.918	0.187228865
Zinc finger Ran-binding domain-containing protein 2 [OS=Homo sapiens]	0.918	0.746263265
CDK-activating kinase assembly factor MAT1 [OS=Homo sapiens]	0.918	0.056665643
DNA/RNA-binding protein KIN17 [OS=Homo sapiens]	0.918	0.356480648
Peptidyl-prolyl cis-trans isomerase H [OS=Homo sapiens]	0.918	0.43720151
39S ribosomal protein L28, mitochondrial [OS=Homo sapiens]	0.918	0.085273252
Signal transducing adapter molecule 2 [OS=Homo sapiens]	0.918	0.992567232
Zinc finger CCCH domain-containing protein 8 [OS=Homo sapiens]	0.918	0.957172022
Pre-mRNA-splicing factor 38A [OS=Homo sapiens]	0.918	0.940735661
Transcription initiation factor TFIID subunit 7 [OS=Homo sapiens]	0.918	0.214256052
Diphthine methyltransferase [OS=Homo sapiens]	0.918	0.907768783
Casein kinase I isoform gamma-3 [OS=Homo sapiens]	0.918	0.992482363
TLE family member 5 [OS=Homo sapiens]	0.918	0.932449925
UDP-N-acetylhexosamine pyrophosphorylase [OS=Homo sapiens]	0.919	0.005295199
Heterogeneous nuclear ribonucleoprotein D0 [OS=Homo sapiens]	0.919	0.520068742
Quinone oxidoreductase-like protein 1 [OS=Homo sapiens]	0.919	0.818773927
Nucleolar MIF4G domain-containing protein 1 [OS=Homo sapiens]	0.919	0.34830128
Lanosterol 14-alpha demethylase [OS=Homo sapiens]	0.919	0.724512287
E3 SUMO-protein ligase ZNF451 [OS=Homo sapiens]	0.919	0.676410405
Nuclear factor 1 X-type [OS=Homo sapiens]	0.919	0.978241138
Protein HEXIM2 [OS=Homo sapiens]	0.919	0.7769948
39S ribosomal protein L55, mitochondrial [OS=Homo sapiens]	0.919	0.999380558
RWD domain-containing protein 4 [OS=Homo sapiens]	0.919	0.759964283
Tumor necrosis factor receptor superfamily member 12A [OS=Homo sapiens]	0.919	0.999882706
GPI ethanolamine phosphate transferase 1 [OS=Homo sapiens]	0.919	0.996330754
F-box/SPRY domain-containing protein 1 [OS=Homo sapiens]	0.919	0.992442931
Rho guanine nucleotide exchange factor 3 [OS=Homo sapiens]	0.919	0.732269817
Mitotic checkpoint protein BUB3 [OS=Homo sapiens]	0.92	0.320543261
Translin [OS=Homo sapiens]	0.92	0.976334526
Protein SCO2 homolog, mitochondrial [OS=Homo sapiens]	0.92	0.737975687
tRNA wybutosine-synthesizing protein 3 homolog [OS=Homo sapiens]	0.92	0.80520778
COUP transcription factor 1 [OS=Homo sapiens]	0.92	0.927373549
Snurportin-1 [OS=Homo sapiens]	0.92	0.901236041
Gamma-adducin [OS=Homo sapiens]	0.92	0.511580475
Terminal nucleotidyltransferase 4B [OS=Homo sapiens]	0.92	0.999975774
Serine-threonine kinase receptor-associated protein [OS=Homo sapiens]	0.921	0.725598177
Mitogen-activated protein kinase 14 [OS=Homo sapiens]	0.921	0.993078316
Protein FAM210A [OS=Homo sapiens]	0.921	0.981754587
ATP-dependent RNA helicase DHX33 [OS=Homo sapiens]	0.921	0.606323001
Putative ribosome-binding factor A, mitochondrial [OS=Homo sapiens]	0.921	0.183345359
Nuclear receptor coactivator 6 [OS=Homo sapiens]	0.921	0.875963372
Zinc finger protein 624 [OS=Homo sapiens]	0.921	0.099231282
LIM domain-binding protein 1 [OS=Homo sapiens]	0.921	0.768639597
Chromodomain-helicase-DNA-binding protein 4 [OS=Homo sapiens]	0.922	0.67457444
Leucine-rich repeat flightless-interacting protein 1 [OS=Homo sapiens]	0.922	0.316027626
Metastasis-associated protein MTA2 [OS=Homo sapiens]	0.922	0.004527704
Histone H1.10 [OS=Homo sapiens]	0.922	0.843239248
REST corepressor 1 [OS=Homo sapiens]	0.922	0.0996945
Cytochrome c oxidase subunit 6B1 [OS=Homo sapiens]	0.922	0.649337092
Exosome complex component RRP4 [OS=Homo sapiens]	0.922	0.459653572
TBC1 domain family member 10B [OS=Homo sapiens]	0.922	0.980852032
OCIA domain-containing protein 2 [OS=Homo sapiens]	0.922	0.998705624
Hepatoma-derived growth factor [OS=Homo sapiens]	0.923	0.516765279
Ephrin type-A receptor 2 [OS=Homo sapiens]	0.923	0.519275562
Helicase-like transcription factor [OS=Homo sapiens]	0.923	0.438079888
Uridine 5'-monophosphate synthase [OS=Homo sapiens]	0.923	0.999987696
28S ribosomal protein S6, mitochondrial [OS=Homo sapiens]	0.923	0.677624583
Coordinator of PRMT5 and differentiation stimulator [OS=Homo sapiens]	0.923	0.971279694
PRKR-interacting protein 1 [OS=Homo sapiens]	0.923	0.92470276
Geranylgeranyl transferase type-2 subunit beta [OS=Homo sapiens]	0.923	0.633052655
39S ribosomal protein L27, mitochondrial [OS=Homo sapiens]	0.923	0.953034829
Peroxisomal bifunctional enzyme [OS=Homo sapiens]	0.923	0.94096972

Adenine DNA glycosylase [OS=Homo sapiens]	0.923	0.911056617
43 kDa receptor-associated protein of the synapse [OS=Homo sapiens]	0.923	0.829158007
Inositol 1,4,5-trisphosphate receptor type 3 [OS=Homo sapiens]	0.924	0.935948752
SUN domain-containing protein 2 [OS=Homo sapiens]	0.924	0.999107112
Choline dehydrogenase, mitochondrial [OS=Homo sapiens]	0.924	0.916966352
General transcription factor 3C polypeptide 6 [OS=Homo sapiens]	0.924	0.541946671
Transmembrane protein 70, mitochondrial [OS=Homo sapiens]	0.924	0.94188292
DnaJ homolog subfamily C member 25 [OS=Homo sapiens]	0.924	0.999964464
Tumor protein D54 [OS=Homo sapiens]	0.925	0.471412676
ATP-dependent RNA helicase DDX19B [OS=Homo sapiens]	0.925	0.999550121
Peripheral plasma membrane protein CASK [OS=Homo sapiens]	0.925	0.965474568
Cell division cycle protein 23 homolog [OS=Homo sapiens]	0.925	0.998664396
Serine/threonine-protein kinase Chk2 [OS=Homo sapiens]	0.925	0.98578648
Ecotropic viral integration site 5 protein homolog [OS=Homo sapiens]	0.925	0.571795879
G2/mitotic-specific cyclin-B2 [OS=Homo sapiens]	0.925	0.087534122
Activating signal cointegrator 1 [OS=Homo sapiens]	0.925	0.562528808
Retinal rod rhodopsin-sensitive cGMP 3',5'-cyclic phosphodiesterase subunit de	0.925	0.999999892
Chromatin complexes subunit BAP18 [OS=Homo sapiens]	0.925	0.868533619
Nocturnin [OS=Homo sapiens]	0.925	0.999837826
Alkylated DNA repair protein alkB homolog 8 [OS=Homo sapiens]	0.925	0.850618909
Solute carrier family 12 member 2 [OS=Homo sapiens]	0.926	0.992505142
Ras-related protein Rab-35 [OS=Homo sapiens]	0.926	0.675955971
DnaJ homolog subfamily A member 3, mitochondrial [OS=Homo sapiens]	0.926	0.017783573
Glutamate-rich WD repeat-containing protein 1 [OS=Homo sapiens]	0.926	0.353795671
Serine/threonine-protein kinase ATR [OS=Homo sapiens]	0.926	0.358430497
Interferon regulatory factor 2-binding protein 1 [OS=Homo sapiens]	0.926	0.459785311
Tumor protein p53-inducible protein 11 [OS=Homo sapiens]	0.926	0.578137961
YY1-associated protein 1 [OS=Homo sapiens]	0.926	0.582326979
Mitochondrial assembly of ribosomal large subunit protein 1 [OS=Homo sapiens	0.926	0.999564001
CKLF-like MARVEL transmembrane domain-containing protein 4 [OS=Homo sap	0.926	0.999998116
E3 ubiquitin-protein ligase E3D [OS=Homo sapiens]	0.926	0.984185888
DNA repair protein RAD50 [OS=Homo sapiens]	0.927	0.425018619
Importin subunit alpha-1 [OS=Homo sapiens]	0.927	0.082865152
Anamorsin [OS=Homo sapiens]	0.927	0.915107989
Disks large-associated protein 5 [OS=Homo sapiens]	0.927	0.981598625
Cysteine protease ATG4B [OS=Homo sapiens]	0.927	0.97898264
Phospholipid phosphatase 1 [OS=Homo sapiens]	0.927	0.999320174
Alanine aminotransferase 2 [OS=Homo sapiens]	0.927	0.984124397
Bridging integrator 3 [OS=Homo sapiens]	0.927	0.816553038
RNA polymerase-associated protein CTR9 homolog [OS=Homo sapiens]	0.928	0.370766586
Ribose-phosphate pyrophosphokinase 1 [OS=Homo sapiens]	0.928	0.907616172
DnaJ homolog subfamily C member 21 [OS=Homo sapiens]	0.928	0.872489669
Methyl-CpG-binding domain protein 3 [OS=Homo sapiens]	0.928	0.126183522
Ras-related protein Rab-4A [OS=Homo sapiens]	0.928	0.999852458
28S ribosomal protein S18b, mitochondrial [OS=Homo sapiens]	0.928	0.816518015
Serine/arginine-rich splicing factor 5 [OS=Homo sapiens]	0.928	0.724128685
Serine/threonine-protein kinase VRK1 [OS=Homo sapiens]	0.928	0.42164686
Putative methyltransferase C9orf114 [OS=Homo sapiens]	0.928	0.969881031
Bromodomain-containing protein 7 [OS=Homo sapiens]	0.928	0.160809765
Myeloid differentiation primary response protein MyD88 [OS=Homo sapiens]	0.928	0.990399585
Phosphatidylserine synthase 1 [OS=Homo sapiens]	0.928	0.589209554
NADH dehydrogenase [ubiquinone] 1 subunit C1, mitochondrial [OS=Homo sap	0.928	0.999541875
Dehydrogenase/reductase SDR family member 1 [OS=Homo sapiens]	0.928	0.99878162
N-alpha-acetyltransferase 15, NatA auxiliary subunit [OS=Homo sapiens]	0.929	0.997040264
Ras-related protein Rap-1b [OS=Homo sapiens]	0.929	0.58250934
Serine/threonine-protein kinase MARK2 [OS=Homo sapiens]	0.929	0.570623891
Cyclin-K [OS=Homo sapiens]	0.929	0.348994632
Protein LSM14 homolog A [OS=Homo sapiens]	0.929	0.288264458
Succinyl-CoA:3-ketoacid coenzyme A transferase 1, mitochondrial [OS=Homo s	0.929	0.425462056
Zinc finger protein 687 [OS=Homo sapiens]	0.929	0.992758032
Integrator complex subunit 10 [OS=Homo sapiens]	0.929	0.550886375
RuvB-like 1 [OS=Homo sapiens]	0.93	0.005085234
Cystathionine beta-synthase [OS=Homo sapiens]	0.93	0.455623761
Gamma-tubulin complex component 3 [OS=Homo sapiens]	0.93	0.968330981
Transforming acidic coiled-coil-containing protein 1 [OS=Homo sapiens]	0.93	0.553300416
Kanadaptin [OS=Homo sapiens]	0.93	0.237192698
NADH dehydrogenase [ubiquinone] 1 alpha subcomplex assembly factor 3 [OS=	0.93	0.904044125
Forkhead box protein J2 [OS=Homo sapiens]	0.93	0.963687133
PABIR family member 2 [OS=Homo sapiens]	0.93	0.996782436
Nuclear body protein SP140 [OS=Homo sapiens]	0.93	0.999953073
Regulator of nonsense transcripts 1 [OS=Homo sapiens]	0.931	0.258708081
Nuclear ubiquitous casein and cyclin-dependent kinase substrate 1 [OS=Homo	0.931	0.562131804
Integrator complex subunit 1 [OS=Homo sapiens]	0.931	0.376769521
Small kinetochore-associated protein [OS=Homo sapiens]	0.931	0.508153286
Kinetochore protein Spc25 [OS=Homo sapiens]	0.931	0.67416352

High mobility group nucleosome-binding domain-containing protein 4 [OS=Hom	0.931	0.991439212
Probable ATP-dependent RNA helicase DHX40 [OS=Homo sapiens]	0.931	0.988331326
Trafficking protein particle complex subunit 12 [OS=Homo sapiens]	0.931	0.99999583
SH3KBP1-binding protein 1 [OS=Homo sapiens]	0.931	0.984644918
TGF-beta-activated kinase 1 and MAP3K7-binding protein 2 [OS=Homo sapiens]	0.931	0.999713454
Programmed cell death protein 2 [OS=Homo sapiens]	0.931	0.866538093
Mitochondrial fission regulator 2 [OS=Homo sapiens]	0.931	0.998256201
Complex I assembly factor ACAD9, mitochondrial [OS=Homo sapiens]	0.932	0.999953791
Histone-binding protein RBBP4 [OS=Homo sapiens]	0.932	0.615004597
Centrosomal protein of 41 kDa [OS=Homo sapiens]	0.932	0.247692304
Chloride channel CLIC-like protein 1 [OS=Homo sapiens]	0.932	0.584930901
RNA-binding protein 6 [OS=Homo sapiens]	0.932	0.80044061
Reticulophagy regulator 2 [OS=Homo sapiens]	0.932	0.448272897
EH domain-binding protein 1-like protein 1 [OS=Homo sapiens]	0.932	0.740307437
Rhotekin [OS=Homo sapiens]	0.932	0.816373384
Mitochondrial 10-formyltetrahydrofolate dehydrogenase [OS=Homo sapiens]	0.932	0.605707758
Protein CLEC16A [OS=Homo sapiens]	0.932	0.99999993
Cyclin-dependent kinases regulatory subunit 2 [OS=Homo sapiens]	0.932	0.998677672
SWI/SNF-related matrix-associated actin-dependent regulator of chromatin sub	0.933	0.800587844
Calcyclin-binding protein [OS=Homo sapiens]	0.933	0.71107164
Phosphoserine phosphatase [OS=Homo sapiens]	0.933	0.830023918
DnaJ homolog subfamily A member 2 [OS=Homo sapiens]	0.933	0.999763166
Ubiquitin thioesterase otulin [OS=Homo sapiens]	0.933	0.999993045
Mitochondrial Rho GTPase 2 [OS=Homo sapiens]	0.933	0.982156626
RNA demethylase ALKBH5 [OS=Homo sapiens]	0.933	0.024072143
Cytochrome c oxidase assembly factor 6 homolog [OS=Homo sapiens]	0.933	0.485159169
Deoxycytidine kinase [OS=Homo sapiens]	0.933	0.910358954
39S ribosomal protein L48, mitochondrial [OS=Homo sapiens]	0.933	0.783167024
Arginine and glutamate-rich protein 1 [OS=Homo sapiens]	0.933	0.808687273
Death-associated protein kinase 3 [OS=Homo sapiens]	0.933	0.979153664
UBX domain-containing protein 4 [OS=Homo sapiens]	0.933	0.955316772
Poly [ADP-ribose] polymerase 2 [OS=Homo sapiens]	0.933	0.405659326
Endonuclease III-like protein 1 [OS=Homo sapiens]	0.933	0.961636821
Lysine-specific histone demethylase 1A [OS=Homo sapiens]	0.934	0.006620016
Mismatch repair endonuclease PMS2 [OS=Homo sapiens]	0.934	0.966060859
Protein timeless homolog [OS=Homo sapiens]	0.934	0.274838973
NmrA-like family domain-containing protein 1 [OS=Homo sapiens]	0.934	0.999999911
Serine/threonine-protein kinase TAO3 [OS=Homo sapiens]	0.934	0.466025485
TIMELESS-interacting protein [OS=Homo sapiens]	0.934	0.747390412
Zinc finger protein 629 [OS=Homo sapiens]	0.934	0.999826121
Sigma non-opioid intracellular receptor 1 [OS=Homo sapiens]	0.934	0.999995588
Anaphase-promoting complex subunit 4 [OS=Homo sapiens]	0.934	0.953079285
Mitochondrial import receptor subunit TOM70 [OS=Homo sapiens]	0.935	0.965522182
39S ribosomal protein L38, mitochondrial [OS=Homo sapiens]	0.935	0.715131441
Actin-binding protein WASF2 [OS=Homo sapiens]	0.935	0.907407402
Splicing factor 3B subunit 5 [OS=Homo sapiens]	0.935	0.779416934
60S ribosomal protein L35 [OS=Homo sapiens]	0.935	0.997611559
Cytochrome c oxidase assembly factor 3 homolog, mitochondrial [OS=Homo sa	0.935	0.999998962
FERM domain-containing protein 8 [OS=Homo sapiens]	0.935	0.988436191
Transmembrane protein 237 [OS=Homo sapiens]	0.935	0.999743739
Oxysterol-binding protein-related protein 6 [OS=Homo sapiens]	0.935	0.796288801
F-box/LRR-repeat protein 3 [OS=Homo sapiens]	0.935	0.530010148
T-complex protein 1 subunit zeta [OS=Homo sapiens]	0.936	0.999784547
Ubiquitin carboxyl-terminal hydrolase isozyme L5 [OS=Homo sapiens]	0.936	0.234977489
Protein CIP2A [OS=Homo sapiens]	0.936	0.110197857
Coiled-coil-helix-coiled-coil-helix domain-containing protein 5 [OS=Homo sapie	0.936	0.518276044
Protein MTO1 homolog, mitochondrial [OS=Homo sapiens]	0.936	0.550742861
tRNA (guanine-N(7)-)-methyltransferase [OS=Homo sapiens]	0.936	0.999999991
Pleckstrin homology domain-containing family O member 2 [OS=Homo sapiens]	0.936	0.755099653
EF-hand domain-containing protein D2 [OS=Homo sapiens]	0.936	0.989343261
Synaptophysin-like protein 1 [OS=Homo sapiens]	0.936	0.446338536
Translation initiation factor IF-3, mitochondrial [OS=Homo sapiens]	0.936	0.975656504
Mitochondrial carnitine/acylcarnitine carrier protein [OS=Homo sapiens]	0.936	0.703127693
Protein DEK [OS=Homo sapiens]	0.937	0.868723611
Serine/threonine-protein kinase greatwall [OS=Homo sapiens]	0.937	0.968610355
Zinc finger C3H1 domain-containing protein [OS=Homo sapiens]	0.937	0.991286844
ERBB receptor feedback inhibitor 1 [OS=Homo sapiens]	0.937	0.697062265
Translocon-associated protein subunit gamma [OS=Homo sapiens]	0.937	0.990771222
Cyclin-A2 [OS=Homo sapiens]	0.937	0.722485991
Cytoskeleton-associated protein 4 [OS=Homo sapiens]	0.938	0.94198277
Transcription elongation factor SPT6 [OS=Homo sapiens]	0.938	0.967685432
Histone deacetylase 1 [OS=Homo sapiens]	0.938	0.230975397
Retinoblastoma-associated protein [OS=Homo sapiens]	0.938	0.769305312
Chromobox protein homolog 3 [OS=Homo sapiens]	0.938	0.871207881
Rab GTPase-activating protein 1 [OS=Homo sapiens]	0.938	0.155158442

Periodic tryptophan protein 1 homolog [OS=Homo sapiens]	0.938	0.63830409
Sodium-coupled neutral amino acid symporter 2 [OS=Homo sapiens]	0.938	0.944697164
Transcription initiation factor TFIID subunit 2 [OS=Homo sapiens]	0.938	0.999584139
U6 snRNA-associated Sm-like protein LSm5 [OS=Homo sapiens]	0.938	0.997272089
Vacuole membrane protein 1 [OS=Homo sapiens]	0.938	0.928740143
BTB/POZ domain-containing protein KCTD18 [OS=Homo sapiens]	0.938	0.921936793
Mitochondrial amidoxime-reducing component 1 [OS=Homo sapiens]	0.938	0.950723119
Peptidyl-prolyl cis-trans isomerase FKBP5 [OS=Homo sapiens]	0.939	0.970930381
Ribose-phosphate pyrophosphokinase 2 [OS=Homo sapiens]	0.939	0.123013498
Tubulin gamma-1 chain [OS=Homo sapiens]	0.939	0.820422199
28S ribosomal protein S22, mitochondrial [OS=Homo sapiens]	0.939	0.684931054
DNA-directed RNA polymerases I, II, and III subunit RPABC1 [OS=Homo sapiens	0.939	0.519911255
Integrator complex subunit 13 [OS=Homo sapiens]	0.939	0.745470489
Dr1-associated corepressor [OS=Homo sapiens]	0.939	0.78803096
NADH dehydrogenase [ubiquinone] flavoprotein 2, mitochondrial [OS=Homo sa	0.939	0.631802158
RalBP1-associated Eps domain-containing protein 1 [OS=Homo sapiens]	0.939	0.991394515
39S ribosomal protein L15, mitochondrial [OS=Homo sapiens]	0.939	0.225965949
LRP chaperone MESD [OS=Homo sapiens]	0.939	0.950849602
General transcription factor IIH subunit 4 [OS=Homo sapiens]	0.939	0.164152837
E3 ubiquitin-protein ligase KCMF1 [OS=Homo sapiens]	0.939	0.96791439
Macoilin [OS=Homo sapiens]	0.939	0.847148583
C2 calcium-dependent domain-containing protein 4B [OS=Homo sapiens]	0.939	0.997907422
BOS complex subunit NOMO1 [OS=Homo sapiens]	0.94	0.99998164
RuvB-like 2 [OS=Homo sapiens]	0.94	0.071602919
Thyroid receptor-interacting protein 11 [OS=Homo sapiens]	0.94	0.998418829
Cohesin subunit SA-2 [OS=Homo sapiens]	0.94	0.627743655
Protein IWS1 homolog [OS=Homo sapiens]	0.94	0.50038692
Kinetochore protein Spc24 [OS=Homo sapiens]	0.94	0.629369407
Tyrosine-protein phosphatase non-receptor type 2 [OS=Homo sapiens]	0.94	0.999999687
DEP domain-containing mTOR-interacting protein [OS=Homo sapiens]	0.94	0.999328507
B-cell lymphoma/leukemia 10 [OS=Homo sapiens]	0.94	0.35161288
Disks large homolog 1 [OS=Homo sapiens]	0.94	0.985615883
Protein O-linked-mannose beta-1,2-N-acetylglucosaminyltransferase 1 [OS=Ho	0.94	0.999932361
Protein ZNRD2 [OS=Homo sapiens]	0.94	0.998937267
Transcription activator BRG1 [OS=Homo sapiens]	0.94	0.999067997
CUE domain-containing protein 2 [OS=Homo sapiens]	0.94	0.276381552
Serine/threonine-protein kinase PAK 2 [OS=Homo sapiens]	0.941	0.180494244
Tuftelin-interacting protein 11 [OS=Homo sapiens]	0.941	0.36765555
Guanine nucleotide-binding protein G(I)/G(S)/G(T) subunit beta-1 [OS=Homo sa	0.941	0.891649325
Mitochondrial tRNA methylthiotransferase CDK5RAP1 [OS=Homo sapiens]	0.941	0.968587763
Integrator complex subunit 14 [OS=Homo sapiens]	0.941	0.871798477
Interferon-induced protein with tetratricopeptide repeats 5 [OS=Homo sapiens]	0.941	0.946886841
Glomulin [OS=Homo sapiens]	0.941	0.709105601
Splicing factor YJU2 [OS=Homo sapiens]	0.941	0.468135552
Cysteine-rich protein 1 [OS=Homo sapiens]	0.941	0.787965863
5'-nucleotidase domain-containing protein 3 [OS=Homo sapiens]	0.941	0.39151841
Pre-mRNA-splicing factor 38B [OS=Homo sapiens]	0.941	0.989541495
FK506-binding protein-like [OS=Homo sapiens]	0.941	0.960646039
Replication factor C subunit 4 [OS=Homo sapiens]	0.942	0.542528379
Ras-related protein Rab-8A [OS=Homo sapiens]	0.942	0.028436866
DNA mismatch repair protein Mlh1 [OS=Homo sapiens]	0.942	0.963950901
Anaphase-promoting complex subunit 7 [OS=Homo sapiens]	0.942	0.427802998
Intracellular hyaluronan-binding protein 4 [OS=Homo sapiens]	0.942	0.999991811
CCR4-NOT transcription complex subunit 9 [OS=Homo sapiens]	0.942	0.999203956
28S ribosomal protein S34, mitochondrial [OS=Homo sapiens]	0.942	0.997214946
Ubiquitin carboxyl-terminal hydrolase 22 [OS=Homo sapiens]	0.942	0.984050325
Splicing factor Cactin [OS=Homo sapiens]	0.942	0.917436353
TBC1 domain family member 24 [OS=Homo sapiens]	0.942	0.957939404
tRNA dimethylallyltransferase [OS=Homo sapiens]	0.942	0.949208336
Actin, cytoplasmic 1 [OS=Homo sapiens]	0.943	0.969892143
Tubulin beta chain [OS=Homo sapiens]	0.943	0.999985661
Creatine kinase B-type [OS=Homo sapiens]	0.943	0.522949853
High mobility group protein B1 [OS=Homo sapiens]	0.943	0.999769898
Cytoplasmic dynein 1 intermediate chain 2 [OS=Homo sapiens]	0.943	0.976934109
Transducin beta-like protein 2 [OS=Homo sapiens]	0.943	0.697810612
Hexokinase-2 [OS=Homo sapiens]	0.943	0.889817761
Choline-phosphate cytidylyltransferase A [OS=Homo sapiens]	0.943	0.97977693
Death domain-associated protein 6 [OS=Homo sapiens]	0.943	0.224722213
Beta-2-syntrophin [OS=Homo sapiens]	0.943	0.949551864
39S ribosomal protein L24, mitochondrial [OS=Homo sapiens]	0.943	0.6556637
Arpin [OS=Homo sapiens]	0.943	0.972099383
Activating signal cointegrator 1 complex subunit 1 [OS=Homo sapiens]	0.943	0.718861171
Zinc finger and SCAN domain-containing protein 18 [OS=Homo sapiens]	0.943	0.972332433
Interferon regulatory factor 2 [OS=Homo sapiens]	0.943	0.996066279
Vesicle-fusing ATPase [OS=Homo sapiens]	0.944	0.527192119

Nucleoredoxin [OS=Homo sapiens]	0.944	0.940594707
Centromere/kinetochore protein zw10 homolog [OS=Homo sapiens]	0.944	0.99982539
Nibrin [OS=Homo sapiens]	0.944	0.80524562
G patch domain-containing protein 8 [OS=Homo sapiens]	0.944	0.435939002
NAD-dependent protein deacetylase sirtuin-1 [OS=Homo sapiens]	0.944	0.631405706
dCTP pyrophosphatase 1 [OS=Homo sapiens]	0.944	0.420411749
39S ribosomal protein L21, mitochondrial [OS=Homo sapiens]	0.944	0.999888564
Up-regulator of cell proliferation [OS=Homo sapiens]	0.944	0.777175061
Endophilin-B1 [OS=Homo sapiens]	0.944	0.9908419
G2/mitotic-specific cyclin-B1 [OS=Homo sapiens]	0.945	0.158424544
HAUS augmin-like complex subunit 1 [OS=Homo sapiens]	0.945	0.64044222
B-cell CLL/lymphoma 7 protein family member C [OS=Homo sapiens]	0.945	0.27271159
RAB7A-interacting MON1-CCZ1 complex subunit 1 [OS=Homo sapiens]	0.945	0.996378714
tRNA-uridine aminocarboxypropyltransferase 1 [OS=Homo sapiens]	0.945	0.992198757
Transcription factor TFIIIB component B'' homolog [OS=Homo sapiens]	0.945	0.9976988
Stress-70 protein, mitochondrial [OS=Homo sapiens]	0.946	0.499142097
Tight junction protein ZO-2 [OS=Homo sapiens]	0.946	0.347689412
Unconventional myosin-Ib [OS=Homo sapiens]	0.946	0.948387745
Succinate--CoA ligase [GDP-forming] subunit beta, mitochondrial [OS=Homo sa	0.946	0.915637906
Prothymosin alpha [OS=Homo sapiens]	0.946	0.398116532
Nitric oxide-associated protein 1 [OS=Homo sapiens]	0.946	0.957935762
Metaxin-1 [OS=Homo sapiens]	0.946	0.995338575
E3 ubiquitin-protein ligase TRIM21 [OS=Homo sapiens]	0.946	1
DNA polymerase alpha subunit B [OS=Homo sapiens]	0.946	0.720128523
Ubiquitin-associated domain-containing protein 2 [OS=Homo sapiens]	0.946	0.929409904
RNA-binding protein MEX3D [OS=Homo sapiens]	0.946	0.4409275
Lysophospholipid acyltransferase 5 [OS=Homo sapiens]	0.946	0.999999204
F-box only protein 38 [OS=Homo sapiens]	0.946	0.982539369
Motor neuron and pancreas homeobox protein 1 [OS=Homo sapiens]	0.946	0.988505303
Splicing factor U2AF 65 kDa subunit [OS=Homo sapiens]	0.947	0.769788492
Optineurin [OS=Homo sapiens]	0.947	0.996685357
Cell division cycle protein 20 homolog [OS=Homo sapiens]	0.947	0.618552622
Protein SAAL1 [OS=Homo sapiens]	0.947	0.834720436
Neural cell adhesion molecule L1 [OS=Homo sapiens]	0.947	0.800451086
Zinc finger protein 136 [OS=Homo sapiens]	0.947	0.965145989
Recombining binding protein suppressor of hairless [OS=Homo sapiens]	0.947	0.692735143
Filamin-A [OS=Homo sapiens]	0.948	0.603284737
Kinesin light chain 1 [OS=Homo sapiens]	0.948	0.290874065
Serine/threonine-protein phosphatase PP1-beta catalytic subunit [OS=Homo sa	0.948	0.810937527
Parkinson disease protein 7 [OS=Homo sapiens]	0.948	0.200702106
Vasodilator-stimulated phosphoprotein [OS=Homo sapiens]	0.948	0.999993686
Heterogeneous nuclear ribonucleoprotein L-like [OS=Homo sapiens]	0.948	0.571846244
Ena/VASP-like protein [OS=Homo sapiens]	0.948	0.999998614
Pleckstrin homology domain-containing family G member 2 [OS=Homo sapiens]	0.948	0.999219014
Ubiquitin-conjugating enzyme E2 A [OS=Homo sapiens]	0.948	0.999056546
Importin-7 [OS=Homo sapiens]	0.949	0.775464779
DNA-(apurinic or apyrimidinic site) endonuclease [OS=Homo sapiens]	0.949	0.719373039
General transcription factor 3C polypeptide 5 [OS=Homo sapiens]	0.949	0.482262277
Mevalonate kinase [OS=Homo sapiens]	0.949	0.999999893
CDGSH iron-sulfur domain-containing protein 1 [OS=Homo sapiens]	0.949	0.998813729
Golgin subfamily A member 7 [OS=Homo sapiens]	0.949	0.997847043
Latexin [OS=Homo sapiens]	0.949	0.995773915
NF-X1-type zinc finger protein NFXL1 [OS=Homo sapiens]	0.949	0.999996028
Nipped-B-like protein [OS=Homo sapiens]	0.95	0.752843193
60S ribosomal protein L18 [OS=Homo sapiens]	0.95	0.972422571
1,4-alpha-glucan-branching enzyme [OS=Homo sapiens]	0.95	0.871802225
Kelch-like ECH-associated protein 1 [OS=Homo sapiens]	0.95	0.098158147
Mitogen-activated protein kinase kinase kinase 7 [OS=Homo sapiens]	0.95	0.97298401
Transmembrane protein 165 [OS=Homo sapiens]	0.95	0.946831325
Transcriptional activator protein Pur-beta [OS=Homo sapiens]	0.95	0.21955479
R3H domain-containing protein 4 [OS=Homo sapiens]	0.95	0.975864839
Ribonuclease H1 [OS=Homo sapiens]	0.95	0.999471642
GDP-mannose 4,6 dehydratase [OS=Homo sapiens]	0.95	0.947335834
Transcription initiation factor TFIID subunit 5 [OS=Homo sapiens]	0.95	0.999993819
Vesicle transport protein SFT2B [OS=Homo sapiens]	0.95	0.945043066
Peroxisomal trans-2-enoyl-CoA reductase [OS=Homo sapiens]	0.95	0.998577871
Histone H1.2 [OS=Homo sapiens]	0.951	0.969354934
Protein phosphatase 1G [OS=Homo sapiens]	0.951	0.505373549
C-terminal-binding protein 2 [OS=Homo sapiens]	0.951	0.114717652
LIM domain-containing protein 1 [OS=Homo sapiens]	0.951	0.999746453
Replication factor C subunit 3 [OS=Homo sapiens]	0.951	0.681511654
Methylosome subunit pICln [OS=Homo sapiens]	0.951	0.750514387
Transcriptional repressor p66-beta [OS=Homo sapiens]	0.951	0.370727648
Alpha-adducin [OS=Homo sapiens]	0.951	0.853652173
Rho GTPase-activating protein 29 [OS=Homo sapiens]	0.951	0.997207971

Acyl-CoA (8-3)-desaturase [OS=Homo sapiens]	0.951	0.989609713
60S ribosomal protein L37 [OS=Homo sapiens]	0.951	0.999999668
Kinase D-interacting substrate of 220 kDa [OS=Homo sapiens]	0.951	0.999933463
Cytohesin-1 [OS=Homo sapiens]	0.951	0.999586287
Jupiter microtubule associated homolog 2 [OS=Homo sapiens]	0.952	0.986287695
SURP and G-patch domain-containing protein 1 [OS=Homo sapiens]	0.952	0.942047091
Serine/arginine-rich splicing factor 2 [OS=Homo sapiens]	0.952	0.847149151
Cold-inducible RNA-binding protein [OS=Homo sapiens]	0.952	0.533921755
S-adenosyl-L-methionine-dependent tRNA 4-demethylwyosine synthase TYW1 [	0.952	0.999997223
Threonine synthase-like 1 [OS=Homo sapiens]	0.952	0.998505468
Meiosis-specific with OB domain-containing protein [OS=Homo sapiens]	0.952	0.768689746
Putative Polycomb group protein ASXL2 [OS=Homo sapiens]	0.952	0.886937513
Zinc finger protein 469 [OS=Homo sapiens]	0.952	0.961885159
Myosin-14 [OS=Homo sapiens]	0.953	0.993934198
Cell cycle and apoptosis regulator protein 2 [OS=Homo sapiens]	0.953	0.879910321
Tropomodulin-3 [OS=Homo sapiens]	0.953	0.927074184
TATA-binding protein-associated factor 2N [OS=Homo sapiens]	0.953	0.976476983
HAUS augmin-like complex subunit 6 [OS=Homo sapiens]	0.953	0.727354554
NADH dehydrogenase [ubiquinone] flavoprotein 3, mitochondrial [OS=Homo sa	0.953	0.999995809
Enoyl-CoA hydratase domain-containing protein 3, mitochondrial [OS=Homo sa	0.953	0.992003888
HAUS augmin-like complex subunit 3 [OS=Homo sapiens]	0.953	0.952981933
Chromodomain Y-like protein [OS=Homo sapiens]	0.953	0.999999543
Protein FAM177A1 [OS=Homo sapiens]	0.953	0.990817969
TATA box-binding protein-associated factor RNA polymerase I subunit C [OS=Ho	0.953	0.81666357
Coiled-coil domain-containing protein 174 [OS=Homo sapiens]	0.953	0.866424181
Ceramide-1-phosphate transfer protein [OS=Homo sapiens]	0.953	0.995919803
Rho GTPase-activating protein 4 [OS=Homo sapiens]	0.953	0.999290518
39S ribosomal protein L52, mitochondrial [OS=Homo sapiens]	0.953	0.983538821
Uncharacterized protein C12orf29 [OS=Homo sapiens]	0.953	0.935601828
Ran guanine nucleotide release factor [OS=Homo sapiens]	0.953	0.997983478
Brain-specific angiogenesis inhibitor 1-associated protein 2 [OS=Homo sapiens]	0.954	0.551513365
Mitochondrial intermembrane space import and assembly protein 40 [OS=Homo	0.954	0.965603258
Uncharacterized protein KIAA1143 [OS=Homo sapiens]	0.954	0.809821797
Mitochondrial genome maintenance exonuclease 1 [OS=Homo sapiens]	0.954	0.999975133
Mediator of RNA polymerase II transcription subunit 15 [OS=Homo sapiens]	0.954	0.95390449
Biotin--protein ligase [OS=Homo sapiens]	0.954	0.755492573
Transcription initiation factor TFIID subunit 8 [OS=Homo sapiens]	0.954	0.922835479
Protein CASC3 [OS=Homo sapiens]	0.954	0.999887765
Cyclin-D1-binding protein 1 [OS=Homo sapiens]	0.954	0.995507741
Arf-GAP with Rho-GAP domain, ANK repeat and PH domain-containing protein 1	0.954	0.998729532
tRNA selenocysteine 1-associated protein 1 [OS=Homo sapiens]	0.954	0.999728106
26S proteasome non-ATPase regulatory subunit 14 [OS=Homo sapiens]	0.955	0.97082115
Alanine--tRNA ligase, mitochondrial [OS=Homo sapiens]	0.955	0.986868313
Protein O-glucosyltransferase 3 [OS=Homo sapiens]	0.955	0.999213473
DNA polymerase delta subunit 3 [OS=Homo sapiens]	0.955	0.973674218
Malectin [OS=Homo sapiens]	0.955	0.907744396
ADP-ribosylation factor-like protein 6-interacting protein 4 [OS=Homo sapiens]	0.955	0.776495716
E3 ubiquitin-protein ligase UBR2 [OS=Homo sapiens]	0.955	1
40S ribosomal protein S27-like [OS=Homo sapiens]	0.955	0.999565425
Testis-expressed protein 264 [OS=Homo sapiens]	0.955	0.981883191
Abscission/NoCut checkpoint regulator [OS=Homo sapiens]	0.955	0.999494342
Ashwin [OS=Homo sapiens]	0.955	0.993558928
Protein DPCD [OS=Homo sapiens]	0.955	0.999690983
28S ribosomal protein S33, mitochondrial [OS=Homo sapiens]	0.955	0.374824771
Kinesin-1 heavy chain [OS=Homo sapiens]	0.956	0.507001002
Double-strand break repair protein MRE11 [OS=Homo sapiens]	0.956	0.36831739
Septin-2 [OS=Homo sapiens]	0.956	0.996528239
N-alpha-acetyltransferase 25, NatB auxiliary subunit [OS=Homo sapiens]	0.956	0.996715631
Activating signal cointegrator 1 complex subunit 3 [OS=Homo sapiens]	0.956	0.996122466
STAM-binding protein [OS=Homo sapiens]	0.956	0.865251879
ER membrane protein complex subunit 2 [OS=Homo sapiens]	0.956	0.555131175
ER membrane protein complex subunit 4 [OS=Homo sapiens]	0.956	0.907858085
Aurora kinase A [OS=Homo sapiens]	0.956	0.931542736
12S rRNA N4-methylcytidine (m4C) methyltransferase [OS=Homo sapiens]	0.956	0.951712148
Sideroflexin-5 [OS=Homo sapiens]	0.956	0.99991366
Transcription factor E2-alpha [OS=Homo sapiens]	0.956	0.994873694
DNA mismatch repair protein Msh6 [OS=Homo sapiens]	0.957	0.885910866
Serine--tRNA ligase, cytoplasmic [OS=Homo sapiens]	0.957	0.649614264
Histone deacetylase 2 [OS=Homo sapiens]	0.957	0.295593612
Erbin [OS=Homo sapiens]	0.957	0.9967794
E3 ubiquitin-protein ligase TRIM47 [OS=Homo sapiens]	0.957	0.975664213
Mitochondrial chaperone BCS1 [OS=Homo sapiens]	0.957	0.977356107
Probable helicase senataxin [OS=Homo sapiens]	0.957	0.829313386
Protein numb homolog [OS=Homo sapiens]	0.957	0.985621486
eEF1A lysine and N-terminal methyltransferase [OS=Homo sapiens]	0.957	0.999998703

Zinc finger MIZ domain-containing protein 1 [OS=Homo sapiens]	0.957	1
Palmitoyltransferase ZDHHC17 [OS=Homo sapiens]	0.957	0.99999995
Pleckstrin homology domain-containing family A member 7 [OS=Homo sapiens]	0.957	0.940098708
Obscurin [OS=Homo sapiens]	0.957	0.877209562
Putative RNA-binding protein Luc7-like 1 [OS=Homo sapiens]	0.958	0.999993463
Telomeric repeat-binding factor 2-interacting protein 1 [OS=Homo sapiens]	0.958	0.759095253
Origin recognition complex subunit 4 [OS=Homo sapiens]	0.958	0.996637393
B-cell receptor-associated protein 29 [OS=Homo sapiens]	0.958	0.999998714
Origin recognition complex subunit 2 [OS=Homo sapiens]	0.958	0.999881755
Ubiquitin carboxyl-terminal hydrolase 28 [OS=Homo sapiens]	0.958	0.998979028
Sterile alpha motif domain-containing protein 11 [OS=Homo sapiens]	0.958	0.588927317
Nuclear transcription factor Y subunit gamma [OS=Homo sapiens]	0.958	0.765973928
Myotubularin-related protein 3 [OS=Homo sapiens]	0.958	0.999965508
Cobalamin trafficking protein CblD [OS=Homo sapiens]	0.958	0.983799829
Tubulin beta-3 chain [OS=Homo sapiens]	0.959	0.999999997
Rab GTPase-binding effector protein 1 [OS=Homo sapiens]	0.959	0.999969262
Proteasome activator complex subunit 3 [OS=Homo sapiens]	0.959	0.990619551
ATP-binding cassette sub-family F member 3 [OS=Homo sapiens]	0.959	0.869185822
Inositol hexakisphosphate and diphosphoinositol-pentakisphosphate kinase 2 [(	0.959	0.799884082
BRO1 domain-containing protein BROX [OS=Homo sapiens]	0.959	0.994313054
Serine/threonine-protein kinase PAK 1 [OS=Homo sapiens]	0.959	1
Ankyrin repeat and LEM domain-containing protein 2 [OS=Homo sapiens]	0.959	0.993980363
Zinc finger and BTB domain-containing protein 21 [OS=Homo sapiens]	0.959	0.998951906
N-alpha-acetyltransferase 20 [OS=Homo sapiens]	0.959	0.989781266
THO complex subunit 3 [OS=Homo sapiens]	0.959	0.509279793
Protein zer-1 homolog [OS=Homo sapiens]	0.959	0.847699374
Glycerol-3-phosphate acyltransferase 4 [OS=Homo sapiens]	0.959	0.96235989
Fas apoptotic inhibitory molecule 1 [OS=Homo sapiens]	0.959	0.823398129
Bcl-2 homologous antagonist/killer [OS=Homo sapiens]	0.959	0.999973191
HEAT repeat-containing protein 5A [OS=Homo sapiens]	0.959	0.999794877
Arachidonate 12-lipoxygenase, 12R-type [OS=Homo sapiens]	0.959	0.824344131
Eukaryotic translation initiation factor 4B [OS=Homo sapiens]	0.96	0.607716778
Rootletin [OS=Homo sapiens]	0.96	0.159137933
Liprin-alpha-1 [OS=Homo sapiens]	0.96	0.880053441
NudC domain-containing protein 1 [OS=Homo sapiens]	0.96	0.992224028
39S ribosomal protein L45, mitochondrial [OS=Homo sapiens]	0.96	0.998605081
Kinetochore-associated protein NSL1 homolog [OS=Homo sapiens]	0.96	0.955114021
G patch domain-containing protein 1 [OS=Homo sapiens]	0.96	0.998394083
Dimethyladenosine transferase 1, mitochondrial [OS=Homo sapiens]	0.96	0.969153616
Threonylcarbamoyl-AMP synthase [OS=Homo sapiens]	0.96	0.992808168
SUN domain-containing ossification factor [OS=Homo sapiens]	0.96	0.999999976
NADH dehydrogenase [ubiquinone] 1 beta subcomplex subunit 3 [OS=Homo sap	0.96	0.999995512
Probable ATP-dependent RNA helicase DHX34 [OS=Homo sapiens]	0.96	0.978782362
Probable ATP-dependent RNA helicase DDX17 [OS=Homo sapiens]	0.961	0.670361636
Putative heat shock protein HSP 90-beta 4 [OS=Homo sapiens]	0.961	0.959627548
Serine/threonine-protein kinase SMG1 [OS=Homo sapiens]	0.961	0.992040991
Tetratricopeptide repeat protein 4 [OS=Homo sapiens]	0.961	0.999997156
Pseudouridylate synthase TRUB1 [OS=Homo sapiens]	0.961	0.69669564
SWI/SNF-related matrix-associated actin-dependent regulator of chromatin sub	0.961	0.999949598
Quinone oxidoreductase PIG3 [OS=Homo sapiens]	0.961	0.999999151
Protein diaphanous homolog 2 [OS=Homo sapiens]	0.961	0.999986525
Proline-rich AKT1 substrate 1 [OS=Homo sapiens]	0.961	0.928005903
Transmembrane protein 41B [OS=Homo sapiens]	0.961	0.999999201
Intraflagellar transport protein 22 homolog [OS=Homo sapiens]	0.961	0.991950932
Pre-mRNA-splicing factor 18 [OS=Homo sapiens]	0.961	0.960698256
ATP-binding cassette sub-family A member 2 [OS=Homo sapiens]	0.961	0.999935286
tRNA pseudouridine(38/39) synthase [OS=Homo sapiens]	0.961	0.999967925
Neuron navigator 1 [OS=Homo sapiens]	0.961	0.999910922
Mini-chromosome maintenance complex-binding protein [OS=Homo sapiens]	0.962	0.789697731
40S ribosomal protein S7 [OS=Homo sapiens]	0.962	0.997560182
Protein PML [OS=Homo sapiens]	0.962	0.999995393
LanC-like protein 2 [OS=Homo sapiens]	0.962	0.968350348
V-type proton ATPase subunit H [OS=Homo sapiens]	0.962	0.999998232
Lysocardiolipin acyltransferase 1 [OS=Homo sapiens]	0.962	0.94856556
DDRGK domain-containing protein 1 [OS=Homo sapiens]	0.962	0.976930931
Golgi SNAP receptor complex member 1 [OS=Homo sapiens]	0.962	0.969476069
Gephyrin [OS=Homo sapiens]	0.962	0.999990557
Actin, alpha cardiac muscle 1 [OS=Homo sapiens]	0.963	0.964569933
Eukaryotic translation initiation factor 3 subunit H [OS=Homo sapiens]	0.963	0.999908162
DnaJ homolog subfamily B member 1 [OS=Homo sapiens]	0.963	0.3528932
Protein arginine N-methyltransferase 5 [OS=Homo sapiens]	0.963	0.999003626
Protein FAM114A2 [OS=Homo sapiens]	0.963	0.998975444
Serine/threonine-protein kinase MRCK alpha [OS=Homo sapiens]	0.963	0.608483249
Exosome complex component MTR3 [OS=Homo sapiens]	0.963	0.952548124
General transcription factor 3C polypeptide 2 [OS=Homo sapiens]	0.963	0.983636415

SEC14-like protein 2 [OS=Homo sapiens]	0.963	0.953660658
MOB kinase activator 1B [OS=Homo sapiens]	0.963	0.99987911
Endoplasmic reticulum metallopeptidase 1 [OS=Homo sapiens]	0.963	0.984984872
Microspherule protein 1 [OS=Homo sapiens]	0.963	0.985390929
Ribonucleoprotein PTB-binding 2 [OS=Homo sapiens]	0.963	0.939301646
Putative transferase CAF17, mitochondrial [OS=Homo sapiens]	0.963	0.994629551
CSC1-like protein 1 [OS=Homo sapiens]	0.963	0.966842758
Serine/threonine-protein kinase Sgk3 [OS=Homo sapiens]	0.963	0.999997873
Vinculin [OS=Homo sapiens]	0.964	0.987269005
Poly [ADP-ribose] polymerase 1 [OS=Homo sapiens]	0.964	0.999934624
Nucleolin [OS=Homo sapiens]	0.964	0.696153812
T-complex protein 1 subunit theta [OS=Homo sapiens]	0.964	0.99819597
X-ray repair cross-complementing protein 6 [OS=Homo sapiens]	0.964	0.313091024
RNA-binding protein FUS [OS=Homo sapiens]	0.964	0.669285047
Protein RCC2 [OS=Homo sapiens]	0.964	0.999048707
Histone-binding protein RBBP7 [OS=Homo sapiens]	0.964	0.882736528
Phosducin-like protein 3 [OS=Homo sapiens]	0.964	0.888622473
Squalene synthase [OS=Homo sapiens]	0.964	0.951757706
Retinol dehydrogenase 11 [OS=Homo sapiens]	0.964	0.994034799
Oxidized purine nucleoside triphosphate hydrolase [OS=Homo sapiens]	0.964	0.998552623
Cytosolic iron-sulfur assembly component 3 [OS=Homo sapiens]	0.964	0.887128339
Vinexin [OS=Homo sapiens]	0.964	0.996608249
TNF receptor-associated factor 5 [OS=Homo sapiens]	0.964	0.991945267
Vacuolar fusion protein MON1 homolog B [OS=Homo sapiens]	0.964	0.866000079
Acyl-coenzyme A diphosphatase NUDT19 [OS=Homo sapiens]	0.964	0.990271205
Early endosome antigen 1 [OS=Homo sapiens]	0.965	0.830754205
Elongation factor 1-delta [OS=Homo sapiens]	0.965	0.993859972
Four and a half LIM domains protein 2 [OS=Homo sapiens]	0.965	0.883461812
General transcription factor 3C polypeptide 3 [OS=Homo sapiens]	0.965	0.635332839
Transforming acidic coiled-coil-containing protein 2 [OS=Homo sapiens]	0.965	0.991930115
YTH domain-containing family protein 1 [OS=Homo sapiens]	0.965	0.973152663
28S ribosomal protein S14, mitochondrial [OS=Homo sapiens]	0.965	0.999764234
CD81 antigen [OS=Homo sapiens]	0.965	0.862510808
DNA repair protein XRCC4 [OS=Homo sapiens]	0.965	0.939656917
KATNB1-like protein 1 [OS=Homo sapiens]	0.965	0.982454493
Serine/threonine-protein kinase RIO2 [OS=Homo sapiens]	0.965	0.942045591
6-pyruvoyl tetrahydrobiopterin synthase [OS=Homo sapiens]	0.965	0.999500269
Zinc finger HIT domain-containing protein 3 [OS=Homo sapiens]	0.965	0.9953
GSK3B-interacting protein [OS=Homo sapiens]	0.965	0.982536077
Kinesin light chain 2 [OS=Homo sapiens]	0.966	0.999960326
WD repeat-containing protein 62 [OS=Homo sapiens]	0.966	0.979026808
5'-3' exoribonuclease 1 [OS=Homo sapiens]	0.966	0.526733647
V-type proton ATPase subunit E 1 [OS=Homo sapiens]	0.966	0.983517813
Rho-related GTP-binding protein RhoG [OS=Homo sapiens]	0.966	0.999933317
Volume-regulated anion channel subunit LRRC8C [OS=Homo sapiens]	0.966	0.993702854
6-phosphofructo-2-kinase/fructose-2,6-bisphosphatase 3 [OS=Homo sapiens]	0.966	0.999923307
WD repeat-containing protein 6 [OS=Homo sapiens]	0.966	0.999998468
39S ribosomal protein L46, mitochondrial [OS=Homo sapiens]	0.966	0.967196355
Putative tRNA (cytidine(32)/guanosine(34)-2'-O)-methyltransferase [OS=Homo s	0.966	0.994085744
E3 ubiquitin-protein ligase TRAF7 [OS=Homo sapiens]	0.966	0.996101714
Signal transducer and activator of transcription 3 [OS=Homo sapiens]	0.967	0.999738648
Serine/threonine-protein phosphatase 6 regulatory subunit 3 [OS=Homo sapiens	0.967	0.999973878
V-type proton ATPase subunit B, brain isoform [OS=Homo sapiens]	0.967	0.999999999
Heme oxygenase 2 [OS=Homo sapiens]	0.967	1
28S ribosomal protein S25, mitochondrial [OS=Homo sapiens]	0.967	0.999964202
Adenosine 5'-monophosphoramidase HINT1 [OS=Homo sapiens]	0.967	0.999999999
Solute carrier family 12 member 7 [OS=Homo sapiens]	0.967	0.992107983
Protein HGH1 homolog [OS=Homo sapiens]	0.967	0.447964415
Programmed cell death protein 2-like [OS=Homo sapiens]	0.967	0.978956402
tRNA (guanine-N(7)-)-methyltransferase non-catalytic subunit WDR4 [OS=Homo	0.967	0.972325305
Ragulator complex protein LAMTOR3 [OS=Homo sapiens]	0.967	0.999999426
D-dopachrome decarboxylase [OS=Homo sapiens]	0.967	0.999153847
Ceramide kinase [OS=Homo sapiens]	0.967	0.99950316
Rho GTPase-activating protein 21 [OS=Homo sapiens]	0.967	0.999855238
Protein GPR108 [OS=Homo sapiens]	0.967	0.612148992
Putative transmembrane protein INAFM2 [OS=Homo sapiens]	0.967	0.962618664
Protein diaphanous homolog 1 [OS=Homo sapiens]	0.968	0.999978305
Phosphatidylinositol 4-phosphate 5-kinase type-1 alpha [OS=Homo sapiens]	0.968	0.88146994
Enhancer of rudimentary homolog [OS=Homo sapiens]	0.968	0.837163784
39S ribosomal protein L37, mitochondrial [OS=Homo sapiens]	0.968	0.810358867
N6-adenosine-methyltransferase catalytic subunit [OS=Homo sapiens]	0.968	0.715368208
Pentatricopeptide repeat-containing protein 1, mitochondrial [OS=Homo sapien	0.968	0.572135286
ATP synthase subunit delta, mitochondrial [OS=Homo sapiens]	0.968	0.999803621
Ceroid-lipofuscinosis neuronal protein 6 [OS=Homo sapiens]	0.968	0.998696684
Alanine--tRNA ligase, cytoplasmic [OS=Homo sapiens]	0.969	0.871454127

Eukaryotic initiation factor 4A-I [OS=Homo sapiens]	0.969	0.963351626
T-complex protein 1 subunit delta [OS=Homo sapiens]	0.969	0.999917743
Transportin-1 [OS=Homo sapiens]	0.969	0.834273054
Trans-Golgi network integral membrane protein 2 [OS=Homo sapiens]	0.969	0.926741528
Transcriptional regulator QRICH1 [OS=Homo sapiens]	0.969	0.998516786
Uncharacterized protein C11orf98 [OS=Homo sapiens]	0.969	0.852137555
Histone deacetylase 6 [OS=Homo sapiens]	0.969	0.99999736
N-alpha-acetyltransferase 35, NatC auxiliary subunit [OS=Homo sapiens]	0.969	0.96605558
Peroxisomal 2,4-dienoyl-CoA reductase [(3E)-enoyl-CoA-producing] [OS=Homo	0.969	0.471272603
Formin-like protein 2 [OS=Homo sapiens]	0.969	0.987349461
Myomegalin [OS=Homo sapiens]	0.969	0.998128877
Heat shock-related 70 kDa protein 2 [OS=Homo sapiens]	0.97	0.999715186
Myosin regulatory light chain 12B [OS=Homo sapiens]	0.97	0.936429839
G protein-regulated inducer of neurite outgrowth 1 [OS=Homo sapiens]	0.97	0.997130687
Argininosuccinate synthase [OS=Homo sapiens]	0.97	0.998734135
Partitioning defective 3 homolog [OS=Homo sapiens]	0.97	0.953702214
DNA polymerase delta catalytic subunit [OS=Homo sapiens]	0.97	0.962425668
F-box only protein 6 [OS=Homo sapiens]	0.97	0.999998501
NHP2-like protein 1 [OS=Homo sapiens]	0.97	0.982551323
Tumor necrosis factor alpha-induced protein 2 [OS=Homo sapiens]	0.97	0.999999783
DNA polymerase subunit gamma-1 [OS=Homo sapiens]	0.97	0.999999987
Probable glutathione peroxidase 8 [OS=Homo sapiens]	0.97	0.999712906
Dedicator of cytokinesis protein 11 [OS=Homo sapiens]	0.97	0.999999718
DNA dC->dU-editing enzyme APOBEC-3C [OS=Homo sapiens]	0.97	0.86193754
Serine/threonine-protein phosphatase 6 catalytic subunit [OS=Homo sapiens]	0.971	0.936092009
Mediator of RNA polymerase II transcription subunit 12 [OS=Homo sapiens]	0.971	0.999296738
HIV Tat-specific factor 1 [OS=Homo sapiens]	0.971	0.996878195
Tripartite motif-containing protein 26 [OS=Homo sapiens]	0.971	0.999999081
Synembryn-A [OS=Homo sapiens]	0.971	0.999987844
Vacuolar fusion protein CCZ1 homolog [OS=Homo sapiens]	0.971	0.999993429
Metastasis-associated protein MTA3 [OS=Homo sapiens]	0.971	0.724834613
NEDD8-conjugating enzyme Ubc12 [OS=Homo sapiens]	0.971	0.849537195
Protein LSM14 homolog B [OS=Homo sapiens]	0.971	0.999995324
Mitotic checkpoint serine/threonine-protein kinase BUB1 [OS=Homo sapiens]	0.971	0.870337867
Tuftelin [OS=Homo sapiens]	0.971	0.95537466
Deoxynucleotidyltransferase terminal-interacting protein 1 [OS=Homo sapiens]	0.971	0.999999539
Transmembrane protease serine 13 [OS=Homo sapiens]	0.971	0.838629641
ATP synthase subunit beta, mitochondrial [OS=Homo sapiens]	0.972	0.998707844
Tubulin alpha-1C chain [OS=Homo sapiens]	0.972	1
Eukaryotic translation initiation factor 5 [OS=Homo sapiens]	0.972	0.993580759
Arf-GAP with coiled-coil, ANK repeat and PH domain-containing protein 2 [OS=H	0.972	0.999586079
Cytoplasmic 60S subunit biogenesis factor ZNF622 [OS=Homo sapiens]	0.972	0.999801164
Segment polarity protein dishevelled homolog DVL-2 [OS=Homo sapiens]	0.972	0.940466259
Coiled-coil domain-containing protein 9 [OS=Homo sapiens]	0.972	0.768920798
Zinc finger protein 800 [OS=Homo sapiens]	0.972	0.993731432
Glutathione synthetase [OS=Homo sapiens]	0.972	0.999999994
Signal-induced proliferation-associated 1-like protein 3 [OS=Homo sapiens]	0.972	0.922443516
Pterin-4-alpha-carbinolamine dehydratase [OS=Homo sapiens]	0.972	0.999779821
Mediator of RNA polymerase II transcription subunit 10 [OS=Homo sapiens]	0.972	0.993309079
Kelch-like protein 11 [OS=Homo sapiens]	0.972	0.999996896
Pre-mRNA-splicing factor SYF2 [OS=Homo sapiens]	0.972	0.940700359
ATP synthase F(0) complex subunit C3, mitochondrial [OS=Homo sapiens]	0.972	0.998447628
Kinectin [OS=Homo sapiens]	0.973	0.997322901
Heat shock protein 105 kDa [OS=Homo sapiens]	0.973	0.92580722
Ubiquitin carboxyl-terminal hydrolase 7 [OS=Homo sapiens]	0.973	0.987901254
Copine-3 [OS=Homo sapiens]	0.973	0.999999993
S-methyl-5'-thioadenosine phosphorylase [OS=Homo sapiens]	0.973	0.996298897
60S ribosomal protein L35a [OS=Homo sapiens]	0.973	0.999999687
28S ribosomal protein S17, mitochondrial [OS=Homo sapiens]	0.973	0.87845604
tRNA-splicing endonuclease subunit Sen2 [OS=Homo sapiens]	0.973	0.999999891
MARVEL domain-containing protein 2 [OS=Homo sapiens]	0.973	0.999988619
39S ribosomal protein L30, mitochondrial [OS=Homo sapiens]	0.973	0.998841825
Cullin-4B [OS=Homo sapiens]	0.974	1
Aspartate aminotransferase, mitochondrial [OS=Homo sapiens]	0.974	0.991526474
FAD-dependent oxidoreductase domain-containing protein 1 [OS=Homo sapien	0.974	0.999982418
Heme-binding protein 1 [OS=Homo sapiens]	0.974	0.979922093
TELO2-interacting protein 2 [OS=Homo sapiens]	0.974	0.999997101
F-box-like/WD repeat-containing protein TBL1X [OS=Homo sapiens]	0.974	0.866465856
General transcription factor IIH subunit 1 [OS=Homo sapiens]	0.974	0.998192494
Small ubiquitin-related modifier 1 [OS=Homo sapiens]	0.974	0.969780694
Alpha-ketoglutarate-dependent dioxygenase alkB homolog 3 [OS=Homo sapiens	0.974	0.931293352
T-complex protein 1 subunit beta [OS=Homo sapiens]	0.975	0.999804681
BAG family molecular chaperone regulator 3 [OS=Homo sapiens]	0.975	0.822641576
Calmodulin-1 [OS=Homo sapiens]	0.975	0.988544719
General transcription factor 3C polypeptide 4 [OS=Homo sapiens]	0.975	0.991158306

Sphingosine-1-phosphate lyase 1 [OS=Homo sapiens]	0.975	0.999408571
Caspase-8 [OS=Homo sapiens]	0.975	0.999800536
Partner of Y14 and mago [OS=Homo sapiens]	0.975	0.999436654
Ribosyldihydronicotinamide dehydrogenase [quinone] [OS=Homo sapiens]	0.975	0.999982588
CCR4-NOT transcription complex subunit 2 [OS=Homo sapiens]	0.975	0.997779223
28S ribosomal protein S16, mitochondrial [OS=Homo sapiens]	0.975	0.974427533
39S ribosomal protein L50, mitochondrial [OS=Homo sapiens]	0.975	0.841910606
Pleckstrin homology domain-containing family G member 1 [OS=Homo sapiens]	0.975	0.999999998
Pre-B-cell leukemia transcription factor 2 [OS=Homo sapiens]	0.975	0.998992368
Xin actin-binding repeat-containing protein 2 [OS=Homo sapiens]	0.975	0.974986817
Synaptojanin-2 [OS=Homo sapiens]	0.976	0.998779302
40S ribosomal protein SA [OS=Homo sapiens]	0.976	0.999998732
KH domain-containing, RNA-binding, signal transduction-associated protein 1 [(	0.976	0.465905906
ADP-ribosylation factor 4 [OS=Homo sapiens]	0.976	0.999826573
Replication initiator 1 [OS=Homo sapiens]	0.976	0.95203643
Transcription factor Sp4 [OS=Homo sapiens]	0.976	0.999925228
Intraflagellar transport protein 81 homolog [OS=Homo sapiens]	0.976	0.952190065
Retinoid-binding protein 7 [OS=Homo sapiens]	0.976	0.999919229
tRNA (uracil-5-)-methyltransferase homolog A [OS=Homo sapiens]	0.976	0.999998174
GDP-Man:Man(3)GlcNAc(2)-PP-Dol alpha-1,2-mannosyltransferase [OS=Homo	0.976	0.999986855
Prostamide/prostaglandin F synthase [OS=Homo sapiens]	0.976	0.996155656
Putative RNA polymerase II subunit B1 CTD phosphatase RPAP2 [OS=Homo sap	0.976	0.999165205
39S ribosomal protein L34, mitochondrial [OS=Homo sapiens]	0.976	0.995441575
Dynein light chain Tctex-type 4 [OS=Homo sapiens]	0.976	0.855025518
Myosin-9 [OS=Homo sapiens]	0.977	0.959368988
Protein SGT1 homolog [OS=Homo sapiens]	0.977	0.997682058
40S ribosomal protein S6 [OS=Homo sapiens]	0.977	0.999914755
Prostaglandin E synthase 3 [OS=Homo sapiens]	0.977	0.999863325
40S ribosomal protein S4, X isoform [OS=Homo sapiens]	0.977	0.999992201
SKI3 subunit of superkiller complex protein [OS=Homo sapiens]	0.977	0.999346004
RB1-inducible coiled-coil protein 1 [OS=Homo sapiens]	0.977	0.999999969
Polycomb protein EED [OS=Homo sapiens]	0.977	0.879405824
V-type proton ATPase subunit D [OS=Homo sapiens]	0.977	0.959465288
Zinc finger CCCH domain-containing protein 7B [OS=Homo sapiens]	0.977	0.999576319
E3 ubiquitin-protein transferase MAEA [OS=Homo sapiens]	0.977	0.999144191
CD82 antigen [OS=Homo sapiens]	0.977	0.982647181
Probable helicase with zinc finger domain [OS=Homo sapiens]	0.977	0.994765381
Peptide-N(4)-(N-acetyl-beta-glucosaminyl)asparagine amidase [OS=Homo sapi	0.977	0.998878839
Pterin-4-alpha-carbinolamine dehydratase 2 [OS=Homo sapiens]	0.977	0.950321804
E3 ubiquitin-protein ligase RNF138 [OS=Homo sapiens]	0.977	0.999711584
Probable E3 ubiquitin-protein ligase HERC3 [OS=Homo sapiens]	0.977	0.999932472
39S ribosomal protein L51, mitochondrial [OS=Homo sapiens]	0.977	0.995203613
Far upstream element-binding protein 1 [OS=Homo sapiens]	0.978	0.999941627
Nuclear inhibitor of protein phosphatase 1 [OS=Homo sapiens]	0.978	0.557258182
Thioredoxin domain-containing protein 9 [OS=Homo sapiens]	0.978	0.993649983
Atlastin-1 [OS=Homo sapiens]	0.978	0.997924764
Cysteine-rich protein 2 [OS=Homo sapiens]	0.978	0.968792981
Transient receptor potential cation channel subfamily M member 4 [OS=Homo s	0.978	0.999997954
CWF19-like protein 2 [OS=Homo sapiens]	0.978	0.949830129
Chromatin accessibility complex protein 1 [OS=Homo sapiens]	0.978	0.999999337
Centrosome and spindle pole-associated protein 1 [OS=Homo sapiens]	0.978	0.963409122
Uncharacterized protein C9orf85 [OS=Homo sapiens]	0.978	0.999874103
F-box only protein 44 [OS=Homo sapiens]	0.978	0.999965854
T-complex protein 1 subunit eta [OS=Homo sapiens]	0.979	0.981133998
Protein unc-45 homolog A [OS=Homo sapiens]	0.979	0.999682533
Golgi-specific brefeldin A-resistance guanine nucleotide exchange factor 1 [OS=	0.979	0.998754103
Voltage-dependent anion-selective channel protein 1 [OS=Homo sapiens]	0.979	0.999681296
Heterogeneous nuclear ribonucleoprotein H2 [OS=Homo sapiens]	0.979	0.999869649
Glutathione S-transferase omega-1 [OS=Homo sapiens]	0.979	0.989355353
Probable ATP-dependent RNA helicase DDX20 [OS=Homo sapiens]	0.979	0.999993548
Ras-related protein Ral-B [OS=Homo sapiens]	0.979	0.901320979
Telomeric repeat-binding factor 2 [OS=Homo sapiens]	0.979	0.981085957
Phosducin-like protein [OS=Homo sapiens]	0.979	0.989775527
MICAL-like protein 2 [OS=Homo sapiens]	0.979	0.986954164
PHD finger-like domain-containing protein 5A [OS=Homo sapiens]	0.979	0.902322122
Intraflagellar transport protein 74 homolog [OS=Homo sapiens]	0.979	0.999819868
Exosome complex component CSL4 [OS=Homo sapiens]	0.979	0.999325407
tRNA-splicing endonuclease subunit Sen54 [OS=Homo sapiens]	0.979	0.998661
Uncharacterized protein C9orf40 [OS=Homo sapiens]	0.979	0.999034518
Alpha-actinin-1 [OS=Homo sapiens]	0.98	0.937020831
Na(+)/H(+) exchange regulatory cofactor NHE-RF1 [OS=Homo sapiens]	0.98	0.968531749
60S ribosomal protein L19 [OS=Homo sapiens]	0.98	0.99076656
60S ribosomal protein L26 [OS=Homo sapiens]	0.98	0.995060849
V-type proton ATPase 116 kDa subunit a 3 [OS=Homo sapiens]	0.98	0.99980674
Nucleolysin TIAR [OS=Homo sapiens]	0.98	0.839075335

GRIP and coiled-coil domain-containing protein 1 [OS=Homo sapiens]	0.98	0.999489333
Casein kinase I isoform epsilon [OS=Homo sapiens]	0.98	0.999813485
Phosphorylase b kinase gamma catalytic chain, liver/testis isoform [OS=Homo s	0.98	0.992299937
Queuine tRNA-ribosyltransferase accessory subunit 2 [OS=Homo sapiens]	0.98	0.999477557
Anaphase-promoting complex subunit 16 [OS=Homo sapiens]	0.98	0.998549537
Transcription initiation factor TFIID subunit 10 [OS=Homo sapiens]	0.98	0.999157141
Adrenodoxin, mitochondrial [OS=Homo sapiens]	0.98	0.999736324
Olfactory receptor 1M1 [OS=Homo sapiens]	0.98	0.877044712
Protein flightless-1 homolog [OS=Homo sapiens]	0.981	0.997937874
Prolyl endopeptidase-like [OS=Homo sapiens]	0.981	0.862343762
GH3 domain-containing protein [OS=Homo sapiens]	0.981	0.999098983
Nitric oxide synthase-interacting protein [OS=Homo sapiens]	0.981	0.963371536
Phosphofurin acidic cluster sorting protein 1 [OS=Homo sapiens]	0.981	0.958087368
NADH-cytochrome b5 reductase 1 [OS=Homo sapiens]	0.981	0.999995536
LETM1 domain-containing protein 1 [OS=Homo sapiens]	0.981	0.999703507
Non-homologous end-joining factor 1 [OS=Homo sapiens]	0.981	0.999661507
Polyribonucleotide 5'-hydroxyl-kinase Clp1 [OS=Homo sapiens]	0.981	0.99719351
DNA-binding protein SMUBP-2 [OS=Homo sapiens]	0.981	0.999925192
Ubiquitin domain-containing protein 2 [OS=Homo sapiens]	0.981	0.999503673
Gamma-tubulin complex component 5 [OS=Homo sapiens]	0.981	0.993275159
Cytochrome b [OS=Homo sapiens]	0.981	0.931088973
SCL-interrupting locus protein [OS=Homo sapiens]	0.981	0.993713489
Proton-coupled zinc antiporter SLC30A5 [OS=Homo sapiens]	0.981	0.991661634
Noelin [OS=Homo sapiens]	0.981	0.996589772
V-type proton ATPase catalytic subunit A [OS=Homo sapiens]	0.982	0.887498951
FK506-binding protein 15 [OS=Homo sapiens]	0.982	0.998974112
Oxysterol-binding protein 1 [OS=Homo sapiens]	0.982	0.998930025
Dynein light chain 1, cytoplasmic [OS=Homo sapiens]	0.982	0.939067495
Eukaryotic translation initiation factor 3 subunit J [OS=Homo sapiens]	0.982	0.935793324
TATA element modulatory factor [OS=Homo sapiens]	0.982	0.99959854
Eukaryotic translation initiation factor 3 subunit F [OS=Homo sapiens]	0.982	0.999999708
High mobility group protein B3 [OS=Homo sapiens]	0.982	0.993858474
Long-chain-fatty-acid--CoA ligase 1 [OS=Homo sapiens]	0.982	0.999997403
28S ribosomal protein S21, mitochondrial [OS=Homo sapiens]	0.982	0.998997457
Lipase maturation factor 2 [OS=Homo sapiens]	0.982	0.999579275
Interleukin-1 receptor-associated kinase 1 [OS=Homo sapiens]	0.982	0.999967623
Iron-sulfur cluster co-chaperone protein HscB [OS=Homo sapiens]	0.982	0.823039556
MAU2 chromatid cohesion factor homolog [OS=Homo sapiens]	0.982	0.887507518
AN1-type zinc finger protein 5 [OS=Homo sapiens]	0.982	0.874876987
Syntaxin-18 [OS=Homo sapiens]	0.982	0.999999707
Extended synaptotagmin-1 [OS=Homo sapiens]	0.983	0.973017783
Kinesin-like protein KIF1C [OS=Homo sapiens]	0.983	0.937213545
SR-related and CTD-associated factor 4 [OS=Homo sapiens]	0.983	0.992434931
Antigen peptide transporter 2 [OS=Homo sapiens]	0.983	0.999959235
Hepatoma-derived growth factor-related protein 2 [OS=Homo sapiens]	0.983	0.997310667
SWI/SNF-related matrix-associated actin-dependent regulator of chromatin sub	0.983	0.999999998
Ras-related protein Ral-A [OS=Homo sapiens]	0.983	0.999999959
Peroxisomal ATPase PEX6 [OS=Homo sapiens]	0.983	0.999994598
Dysferlin [OS=Homo sapiens]	0.983	0.996110402
Serine/threonine-protein kinase WNK2 [OS=Homo sapiens]	0.983	0.999640568
Ubiquitin-like protein 5 [OS=Homo sapiens]	0.983	0.999964413
39S ribosomal protein S30, mitochondrial [OS=Homo sapiens]	0.983	0.990035955
Cell division cycle protein 16 homolog [OS=Homo sapiens]	0.983	0.999008143
Inositol-tetrakisphosphate 1-kinase [OS=Homo sapiens]	0.983	0.999999875
Fucose-1-phosphate guanylyltransferase [OS=Homo sapiens]	0.983	0.998002937
Palmitoyltransferase ZDHHC4 [OS=Homo sapiens]	0.983	0.999998749
Threonine--tRNA ligase 1, cytoplasmic [OS=Homo sapiens]	0.984	0.946980817
Purine nucleoside phosphorylase [OS=Homo sapiens]	0.984	0.995699765
Brain-specific angiogenesis inhibitor 1-associated protein 2-like protein 1 [OS=H	0.984	1
28S ribosomal protein S23, mitochondrial [OS=Homo sapiens]	0.984	0.999998541
Inositol 1,4,5-trisphosphate receptor type 2 [OS=Homo sapiens]	0.984	0.889944587
Cell division cycle protein 27 homolog [OS=Homo sapiens]	0.984	0.936873755
Transcription initiation factor TFIID subunit 6 [OS=Homo sapiens]	0.984	0.999999999
60S ribosomal protein L15 [OS=Homo sapiens]	0.984	0.99999787
39S ribosomal protein S18a, mitochondrial [OS=Homo sapiens]	0.984	0.984274475
SAP30-binding protein [OS=Homo sapiens]	0.984	0.99711968
Cilia- and flagella-associated protein 298 [OS=Homo sapiens]	0.984	1
Paired amphipathic helix protein Sin3b [OS=Homo sapiens]	0.984	0.841228252
28S ribosomal protein S12, mitochondrial [OS=Homo sapiens]	0.984	0.955192342
Terminal uridylyltransferase 7 [OS=Homo sapiens]	0.984	0.994577967
3-ketodihydrosphingosine reductase [OS=Homo sapiens]	0.984	0.999997376
Nestin [OS=Homo sapiens]	0.984	0.970325057
Activator of 90 kDa heat shock protein ATPase homolog 1 [OS=Homo sapiens]	0.985	0.985808475
40S ribosomal protein S21 [OS=Homo sapiens]	0.985	0.999952823
Interferon regulatory factor 2-binding protein 2 [OS=Homo sapiens]	0.985	0.890937876

Adenosine kinase [OS=Homo sapiens]	0.985	0.99986158
Leucine-rich repeat flightless-interacting protein 2 [OS=Homo sapiens]	0.985	0.998942885
Mortality factor 4-like protein 1 [OS=Homo sapiens]	0.985	0.992844782
cAMP-dependent protein kinase catalytic subunit alpha [OS=Homo sapiens]	0.985	0.990205186
Serine/threonine-protein kinase A-Raf [OS=Homo sapiens]	0.985	0.999999992
Coiled-coil domain-containing protein 12 [OS=Homo sapiens]	0.985	0.987718747
Kinesin-like protein KIF14 [OS=Homo sapiens]	0.985	0.999912549
Diphosphoinositol polyphosphate phosphohydrolase 2 [OS=Homo sapiens]	0.985	0.999963415
Sharpin [OS=Homo sapiens]	0.985	0.984528435
RNA-binding protein Musashi homolog 2 [OS=Homo sapiens]	0.985	0.99593305
Protein FAM3A [OS=Homo sapiens]	0.985	0.999985927
Bifunctional glutamate/proline--tRNA ligase [OS=Homo sapiens]	0.986	0.738321871
General transcription factor II-I [OS=Homo sapiens]	0.986	0.998945175
Eukaryotic translation initiation factor 2D [OS=Homo sapiens]	0.986	0.998267181
General transcription and DNA repair factor IIH helicase subunit XPB [OS=Homo	0.986	0.995221786
TraB domain-containing protein [OS=Homo sapiens]	0.986	0.998750764
Serine dehydratase-like [OS=Homo sapiens]	0.986	0.999976967
Antiviral innate immune response receptor RIG-I [OS=Homo sapiens]	0.986	0.999860386
60S ribosomal protein L37a [OS=Homo sapiens]	0.986	0.980254802
Bifunctional 3'-5' exonuclease/ATP-dependent helicase WRN [OS=Homo sapien	0.986	0.848610294
Segment polarity protein dishevelled homolog DVL-1 [OS=Homo sapiens]	0.986	0.991058189
DNA-directed RNA polymerase II subunit RPB11-a [OS=Homo sapiens]	0.986	0.892220334
Dual specificity protein phosphatase 12 [OS=Homo sapiens]	0.986	0.999999996
Secernin-3 [OS=Homo sapiens]	0.986	0.934588487
Leucine--tRNA ligase, cytoplasmic [OS=Homo sapiens]	0.987	0.981422789
Signal recognition particle subunit SRP68 [OS=Homo sapiens]	0.987	0.999999986
Cyclin-dependent kinase 11B [OS=Homo sapiens]	0.987	0.99999754
Destrin [OS=Homo sapiens]	0.987	0.99996287
40S ribosomal protein S23 [OS=Homo sapiens]	0.987	0.955350618
Very-long-chain (3R)-3-hydroxyacyl-CoA dehydratase 3 [OS=Homo sapiens]	0.987	0.931639053
Ras-related C3 botulinum toxin substrate 1 [OS=Homo sapiens]	0.987	0.999523071
Serine/threonine-protein phosphatase 2A 55 kDa regulatory subunit B alpha isof	0.987	0.979345249
Actin-related protein 2/3 complex subunit 5-like protein [OS=Homo sapiens]	0.987	0.999989021
Deoxyhypusine synthase [OS=Homo sapiens]	0.987	0.999984358
Alpha-ketoglutarate-dependent dioxygenase FTO [OS=Homo sapiens]	0.987	0.999654737
ER membrane protein complex subunit 3 [OS=Homo sapiens]	0.987	0.978097575
Phosphorylase b kinase regulatory subunit alpha, liver isoform [OS=Homo sapie	0.987	0.999999415
E3 ubiquitin-protein ligase TRIM11 [OS=Homo sapiens]	0.987	0.98257052
Coiled-coil domain-containing protein 9B [OS=Homo sapiens]	0.987	0.999997902
Caytaxin [OS=Homo sapiens]	0.987	0.944796048
CCR4-NOT transcription complex subunit 1 [OS=Homo sapiens]	0.988	0.998548097
Cyclin-dependent kinase 13 [OS=Homo sapiens]	0.988	0.995274988
Protein NEDD1 [OS=Homo sapiens]	0.988	0.999950088
CCA tRNA nucleotidyltransferase 1, mitochondrial [OS=Homo sapiens]	0.988	0.999985757
Probable hydrolase PNKD [OS=Homo sapiens]	0.988	0.999556247
Tripartite motif-containing protein 59 [OS=Homo sapiens]	0.988	0.817704693
Transcription factor Sp1 [OS=Homo sapiens]	0.988	0.999932292
Ubiquitin carboxyl-terminal hydrolase 1 [OS=Homo sapiens]	0.988	0.998985577
Carbohydrate sulfotransferase 14 [OS=Homo sapiens]	0.988	0.999999982
Transcription factor Dp family member 3 [OS=Homo sapiens]	0.988	0.99998679
Importin subunit beta-1 [OS=Homo sapiens]	0.989	0.999326901
Importin-9 [OS=Homo sapiens]	0.989	0.826300397
PDZ and LIM domain protein 1 [OS=Homo sapiens]	0.989	0.999996268
Prefoldin subunit 5 [OS=Homo sapiens]	0.989	0.995293011
PEST proteolytic signal-containing nuclear protein [OS=Homo sapiens]	0.989	0.991226698
Synaptosomal-associated protein 23 [OS=Homo sapiens]	0.989	0.986817149
ATP synthase subunit g, mitochondrial [OS=Homo sapiens]	0.989	0.999500846
Splicing factor C9orf78 [OS=Homo sapiens]	0.989	0.999950568
Notchless protein homolog 1 [OS=Homo sapiens]	0.989	0.999999998
AT-rich interactive domain-containing protein 4B [OS=Homo sapiens]	0.989	0.993905323
Putative GTP-binding protein 6 [OS=Homo sapiens]	0.989	0.999111269
Sequestosome-1 [OS=Homo sapiens]	0.989	0.925407032
tRNA N(3)-methylcytidine methyltransferase METTL2B [OS=Homo sapiens]	0.989	0.999265368
Uncharacterized protein C1orf122 [OS=Homo sapiens]	0.989	0.999998931
ATP-dependent DNA helicase Q4 [OS=Homo sapiens]	0.989	0.999951726
Ankyrin repeat domain-containing protein 13A [OS=Homo sapiens]	0.989	0.999999969
Tyrosine-protein kinase JAK2 [OS=Homo sapiens]	0.989	0.999989844
Glycine--tRNA ligase [OS=Homo sapiens]	0.99	0.697001773
GMP synthase [glutamine-hydrolyzing] [OS=Homo sapiens]	0.99	0.998580272
E3 ubiquitin-protein ligase LRSAM1 [OS=Homo sapiens]	0.99	0.973215291
Vesicle-associated membrane protein-associated protein B/C [OS=Homo sapie	0.99	0.987815828
60S ribosomal protein L17 [OS=Homo sapiens]	0.99	0.998297585
Coiled-coil and C2 domain-containing protein 1A [OS=Homo sapiens]	0.99	0.990723136
60S ribosomal protein L29 [OS=Homo sapiens]	0.99	0.991905135
RAF proto-oncogene serine/threonine-protein kinase [OS=Homo sapiens]	0.99	0.894262875

Alpha-parvin [OS=Homo sapiens]	0.99	0.998924451
Arginine/serine-rich coiled-coil protein 2 [OS=Homo sapiens]	0.99	0.997587445
CD151 antigen [OS=Homo sapiens]	0.99	0.999999371
RNA-binding motif, single-stranded-interacting protein 2 [OS=Homo sapiens]	0.99	0.999990217
Peptidyl-prolyl cis-trans isomerase E [OS=Homo sapiens]	0.99	0.993531916
Roquin-1 [OS=Homo sapiens]	0.99	0.999999924
ASNSD1 upstream open reading frame protein [OS=Homo sapiens]	0.99	0.999997654
Acylphosphatase-2 [OS=Homo sapiens]	0.99	0.999999717
Centrosome-associated protein ALMS1 [OS=Homo sapiens]	0.99	0.982369485
Mitogen-activated protein kinase 9 [OS=Homo sapiens]	0.99	0.988452277
Protein FAM53C [OS=Homo sapiens]	0.99	0.99000212
Unconventional myosin-Ic [OS=Homo sapiens]	0.991	0.996801072
Bifunctional phosphoribosylaminoimidazole carboxylase/phosphoribosylamino	0.991	0.999636656
Drebrin-like protein [OS=Homo sapiens]	0.991	0.999469333
Protein Smaug homolog 2 [OS=Homo sapiens]	0.991	0.923299724
Pseudouridylate synthase RPUSD2 [OS=Homo sapiens]	0.991	0.999563399
Probable asparagine--tRNA ligase, mitochondrial [OS=Homo sapiens]	0.991	0.779843733
Prolyl 3-hydroxylase OGFOD1 [OS=Homo sapiens]	0.991	0.998812753
Bcl-2-like protein 1 [OS=Homo sapiens]	0.991	0.999909288
Zinc finger E-box-binding homeobox 1 [OS=Homo sapiens]	0.991	0.999999992
Uncharacterized protein C1orf112 [OS=Homo sapiens]	0.991	0.978595605
Eukaryotic translation initiation factor 3 subunit A [OS=Homo sapiens]	0.992	0.999666258
T-complex protein 1 subunit alpha [OS=Homo sapiens]	0.992	0.999970211
DNA mismatch repair protein Msh2 [OS=Homo sapiens]	0.992	0.999999996
Cysteine--tRNA ligase, cytoplasmic [OS=Homo sapiens]	0.992	0.999999182
Eukaryotic translation initiation factor 3 subunit L [OS=Homo sapiens]	0.992	0.999999835
Mediator of RNA polymerase II transcription subunit 1 [OS=Homo sapiens]	0.992	0.864979057
60S ribosomal protein L18a [OS=Homo sapiens]	0.992	0.999182477
Phosphatidylinositol 3-kinase catalytic subunit type 3 [OS=Homo sapiens]	0.992	0.995907833
Zinc finger HIT domain-containing protein 2 [OS=Homo sapiens]	0.992	0.999975536
Nucleotide triphosphate diphosphatase NUDT15 [OS=Homo sapiens]	0.992	0.999981413
Phospholipase DDHD2 [OS=Homo sapiens]	0.992	0.999874122
Zinc finger protein 511 [OS=Homo sapiens]	0.992	0.9987028
Phosphatidylinositol 4-phosphate 5-kinase type-1 gamma [OS=Homo sapiens]	0.992	0.999892972
HAUS augmin-like complex subunit 8 [OS=Homo sapiens]	0.992	0.999835532
Mitochondrial fission regulator 1-like [OS=Homo sapiens]	0.992	0.997968253
Inositol-trisphosphate 3-kinase B [OS=Homo sapiens]	0.992	0.988265425
Protein phosphatase 1E [OS=Homo sapiens]	0.992	0.903656425
Mixed lineage kinase domain-like protein [OS=Homo sapiens]	0.992	0.998736772
NHL repeat-containing protein 3 [OS=Homo sapiens]	0.992	0.985666254
Cell cycle exit and neuronal differentiation protein 1 [OS=Homo sapiens]	0.992	0.993943165
Exportin-2 [OS=Homo sapiens]	0.993	0.994392703
S-adenosylmethionine synthase isoform type-2 [OS=Homo sapiens]	0.993	0.994645882
Acyl-CoA-binding protein [OS=Homo sapiens]	0.993	0.999824269
Queuosine salvage protein [OS=Homo sapiens]	0.993	0.92563922
Protein phosphatase 1B [OS=Homo sapiens]	0.993	0.996661918
Ubiquitin carboxyl-terminal hydrolase 48 [OS=Homo sapiens]	0.993	0.999985292
40S ribosomal protein S25 [OS=Homo sapiens]	0.993	0.991355937
Probable tRNA(His) guanylyltransferase [OS=Homo sapiens]	0.993	0.999599435
RalA-binding protein 1 [OS=Homo sapiens]	0.993	0.999713305
Ubiquitin carboxyl-terminal hydrolase 34 [OS=Homo sapiens]	0.993	0.979569583
Geranylgeranyl pyrophosphate synthase [OS=Homo sapiens]	0.993	0.999997776
Dickkopf-related protein 1 [OS=Homo sapiens]	0.993	0.993903779
Aurora kinase A-interacting protein [OS=Homo sapiens]	0.993	1
Protein Mis18-alpha [OS=Homo sapiens]	0.993	0.924248991
Protein FAM98B [OS=Homo sapiens]	0.994	0.999999876
Probable E3 ubiquitin-protein ligase IRF2BPL [OS=Homo sapiens]	0.994	0.999999999
39S ribosomal protein L11, mitochondrial [OS=Homo sapiens]	0.994	0.999993848
Lariat debranching enzyme [OS=Homo sapiens]	0.994	0.983166795
Methylosome protein 50 [OS=Homo sapiens]	0.994	0.999998744
Centrin-2 [OS=Homo sapiens]	0.994	0.999652813
Programmed cell death protein 10 [OS=Homo sapiens]	0.994	0.850182407
39S ribosomal protein L4, mitochondrial [OS=Homo sapiens]	0.994	0.999998658
Collagen alpha-2(I) chain [OS=Homo sapiens]	0.994	0.973389414
Mimecan [OS=Homo sapiens]	0.994	0.917021683
Golgi reassembly-stacking protein 1 [OS=Homo sapiens]	0.994	0.993368762
Constitutive coactivator of PPAR-gamma-like protein 1 [OS=Homo sapiens]	0.995	0.999999957
Golgi-associated PDZ and coiled-coil motif-containing protein [OS=Homo sapiei	0.995	0.997341219
G-patch domain and KOW motifs-containing protein [OS=Homo sapiens]	0.995	0.980980935
Wings apart-like protein homolog [OS=Homo sapiens]	0.995	0.996310279
Thioredoxin-related transmembrane protein 1 [OS=Homo sapiens]	0.995	0.990223878
Prefoldin subunit 4 [OS=Homo sapiens]	0.995	0.98288145
Protein phosphatase 1A [OS=Homo sapiens]	0.995	0.892587719
Protein HEXIM1 [OS=Homo sapiens]	0.995	0.999824352
Tyrosyl-DNA phosphodiesterase 2 [OS=Homo sapiens]	0.995	0.996436572

60S ribosomal protein L31 [OS=Homo sapiens]	0.995	0.999788816
Dystroglycan 1 [OS=Homo sapiens]	0.995	0.95336183
General transcription factor IIH subunit 3 [OS=Homo sapiens]	0.995	0.989956922
Proteasomal ATPase-associated factor 1 [OS=Homo sapiens]	0.995	0.999926907
Signal peptidase complex subunit 3 [OS=Homo sapiens]	0.995	0.999917671
E3 ubiquitin-protein ligase Praja-2 [OS=Homo sapiens]	0.995	0.999980889
Zinc finger CCCH domain-containing protein 7A [OS=Homo sapiens]	0.995	0.942910392
Charged multivesicular body protein 6 [OS=Homo sapiens]	0.995	0.999858991
Heat shock protein HSP 90-beta [OS=Homo sapiens]	0.996	0.98820443
Sarcoplasmic/endoplasmic reticulum calcium ATPase 2 [OS=Homo sapiens]	0.996	0.997425763
Y-box-binding protein 1 [OS=Homo sapiens]	0.996	0.946028384
Eukaryotic peptide chain release factor subunit 1 [OS=Homo sapiens]	0.996	0.999997265
Apoptosis inhibitor 5 [OS=Homo sapiens]	0.996	0.952225024
Malignant T-cell-amplified sequence 1 [OS=Homo sapiens]	0.996	0.976880097
Erlin-2 [OS=Homo sapiens]	0.996	0.999999928
Small nuclear ribonucleoprotein-associated proteins B and B' [OS=Homo sapier	0.996	0.784927266
Ran-specific GTPase-activating protein [OS=Homo sapiens]	0.996	0.999999969
DNA mismatch repair protein Msh3 [OS=Homo sapiens]	0.996	0.99827794
28S ribosomal protein S26, mitochondrial [OS=Homo sapiens]	0.996	0.999999936
DNA-3-methyladenine glycosylase [OS=Homo sapiens]	0.996	0.999750098
Uridine-cytidine kinase 2 [OS=Homo sapiens]	0.996	0.999999984
CCR4-NOT transcription complex subunit 11 [OS=Homo sapiens]	0.996	0.99999998
Ubiquitin-like protein 4A [OS=Homo sapiens]	0.996	0.995281179
Ragulator complex protein LAMTOR2 [OS=Homo sapiens]	0.996	0.998992054
Dehydrogenase/reductase SDR family member 4 [OS=Homo sapiens]	0.996	0.999851377
Ubiquinol-cytochrome-c reductase complex assembly factor 1 [OS=Homo sapie	0.996	0.999423059
Vacuolar protein sorting-associated protein 45 [OS=Homo sapiens]	0.996	0.999922734
Forkhead box protein J3 [OS=Homo sapiens]	0.996	0.999997727
GATOR complex protein NPRL3 [OS=Homo sapiens]	0.996	1
Arfaptin-1 [OS=Homo sapiens]	0.996	0.97611855
Retinoic acid-induced protein 1 [OS=Homo sapiens]	0.996	0.871791856
V-type immunoglobulin domain-containing suppressor of T-cell activation [OS=H	0.996	0.957302236
T-complex protein 1 subunit epsilon [OS=Homo sapiens]	0.997	0.955263205
Arginine--tRNA ligase, cytoplasmic [OS=Homo sapiens]	0.997	0.987209924
Nuclear pore complex protein Nup155 [OS=Homo sapiens]	0.997	0.998548203
Tensin-3 [OS=Homo sapiens]	0.997	0.988745789
Dihydrolipoyl dehydrogenase, mitochondrial [OS=Homo sapiens]	0.997	0.995538164
Exportin-T [OS=Homo sapiens]	0.997	1
40S ribosomal protein S13 [OS=Homo sapiens]	0.997	0.965032997
Succinate dehydrogenase [ubiquinone] iron-sulfur subunit, mitochondrial [OS=H	0.997	0.999825754
Ribosome quality control complex subunit NEMF [OS=Homo sapiens]	0.997	0.890921598
Hsp70-binding protein 1 [OS=Homo sapiens]	0.997	0.999782561
UDP-glucose 6-dehydrogenase [OS=Homo sapiens]	0.997	0.999999983
TIP41-like protein [OS=Homo sapiens]	0.997	0.999999872
Serine/threonine-protein kinase Nek9 [OS=Homo sapiens]	0.997	0.92749637
28S ribosomal protein S5, mitochondrial [OS=Homo sapiens]	0.997	0.999952358
Intersectin-1 [OS=Homo sapiens]	0.997	0.99830742
FH1/FH2 domain-containing protein 1 [OS=Homo sapiens]	0.997	0.999977169
Armadillo repeat-containing protein 1 [OS=Homo sapiens]	0.997	0.999947816
tRNA modification GTPase GTPBP3, mitochondrial [OS=Homo sapiens]	0.997	0.994932522
Caspase-2 [OS=Homo sapiens]	0.997	0.999838205
Nucleolar protein 9 [OS=Homo sapiens]	0.997	0.950733094
RanBP-type and C3HC4-type zinc finger-containing protein 1 [OS=Homo sapiens	0.997	0.999984632
Zinc transporter ZIP6 [OS=Homo sapiens]	0.997	0.999967047
Small ubiquitin-related modifier 3 [OS=Homo sapiens]	0.997	0.959082129
Cytoplasmic dynein 1 light intermediate chain 1 [OS=Homo sapiens]	0.998	0.976705426
Tight junction protein ZO-1 [OS=Homo sapiens]	0.998	0.985304416
Aldo-keto reductase family 1 member B1 [OS=Homo sapiens]	0.998	0.999998923
E3 UFM1-protein ligase 1 [OS=Homo sapiens]	0.998	1
Kinesin light chain 4 [OS=Homo sapiens]	0.998	0.969308898
Ras-related protein Rap-1A [OS=Homo sapiens]	0.998	0.999999994
Inhibitor of nuclear factor kappa-B kinase-interacting protein [OS=Homo sapiens	0.998	0.999656197
PSME3-interacting protein [OS=Homo sapiens]	0.998	0.999999852
40S ribosomal protein S24 [OS=Homo sapiens]	0.998	0.977120864
Serine/threonine-protein phosphatase 6 regulatory ankyrin repeat subunit A [OS	0.998	0.998770915
Uridine-cytidine kinase-like 1 [OS=Homo sapiens]	0.998	0.891912752
Gamma-tubulin complex component 6 [OS=Homo sapiens]	0.998	0.99891008
Cytochrome b5 [OS=Homo sapiens]	0.998	0.986729295
tRNA methyltransferase 10 homolog A [OS=Homo sapiens]	0.998	0.999875906
tRNA (cytosine(72)-C(5))-methyltransferase NSUN6 [OS=Homo sapiens]	0.998	0.988159186
Rabankyrin-5 [OS=Homo sapiens]	0.999	0.880326581
60S ribosomal protein L27a [OS=Homo sapiens]	0.999	0.995780511
Cytosolic Fe-S cluster assembly factor NUBP1 [OS=Homo sapiens]	0.999	0.998276909
RING-type E3 ubiquitin-protein ligase PPIL2 [OS=Homo sapiens]	0.999	0.981563447
Glycosaminoglycan xylosylkinase [OS=Homo sapiens]	0.999	0.992287377

Normal mucosa of esophagus-specific gene 1 protein [OS=Homo sapiens]	0.999	0.977725249
3'-5' exoribonuclease 1 [OS=Homo sapiens]	0.999	0.975191061
Pituitary tumor-transforming gene 1 protein-interacting protein [OS=Homo sapie	0.999	0.930230004
UPF0415 protein C7orf25 [OS=Homo sapiens]	0.999	0.999646929
ATP synthase subunit f, mitochondrial [OS=Homo sapiens]	0.999	0.984982856
Putative deoxyribonuclease TATDN3 [OS=Homo sapiens]	0.999	0.883397933
Cotranscriptional regulator FAM172A [OS=Homo sapiens]	0.999	1
Proteasome adapter and scaffold protein ECM29 [OS=Homo sapiens]	1	0.999559629
Bifunctional purine biosynthesis protein ATIC [OS=Homo sapiens]	1	0.971848934
BRCA1-associated ATM activator 1 [OS=Homo sapiens]	1	0.999976723
Serine/threonine-protein kinase OSR1 [OS=Homo sapiens]	1	0.999871499
Diacylglycerol lipase-beta [OS=Homo sapiens]	1	0.962643269
HAUS augmin-like complex subunit 7 [OS=Homo sapiens]	1	0.943058918
Protein dpy-30 homolog [OS=Homo sapiens]	1	0.997097492
ER membrane protein complex subunit 9 [OS=Homo sapiens]	1	0.999999999
Kelch domain-containing protein 4 [OS=Homo sapiens]	1	0.999989113
Putative cytochrome P450 2D7 [OS=Homo sapiens]	1	0.997391869
Putative glycine N-acyltransferase-like protein 1B [OS=Homo sapiens]	1	0.999959583
Serine-protein kinase ATM [OS=Homo sapiens]	1.001	1
3-ketoacyl-CoA thiolase, peroxisomal [OS=Homo sapiens]	1.001	0.846265406
Protein kinase C alpha type [OS=Homo sapiens]	1.001	0.999756439
Peptidyl-prolyl cis-trans isomerase-like 3 [OS=Homo sapiens]	1.001	0.978443845
Pseudouridylate synthase TRUB2, mitochondrial [OS=Homo sapiens]	1.001	0.898310597
Mitochondrial peptide methionine sulfoxide reductase [OS=Homo sapiens]	1.001	0.999217976
V-type proton ATPase subunit F [OS=Homo sapiens]	1.001	0.982840845
Acetyl-coenzyme A synthetase, cytoplasmic [OS=Homo sapiens]	1.001	0.945859833
Transcriptional regulator Kaiso [OS=Homo sapiens]	1.001	0.994510401
Histone acetyltransferase KAT8 [OS=Homo sapiens]	1.001	0.998379321
Mpv17-like protein 2 [OS=Homo sapiens]	1.001	0.997050719
Probable proline--tRNA ligase, mitochondrial [OS=Homo sapiens]	1.001	0.999977139
Folliculin-interacting protein 1 [OS=Homo sapiens]	1.001	0.95305977
Ornithine aminotransferase, mitochondrial [OS=Homo sapiens]	1.002	0.998043898
Src substrate cortactin [OS=Homo sapiens]	1.002	0.989898961
60S acidic ribosomal protein P2 [OS=Homo sapiens]	1.002	0.999876282
Mucosa-associated lymphoid tissue lymphoma translocation protein 1 [OS=Hor	1.002	0.99318256
KIF-binding protein [OS=Homo sapiens]	1.002	0.999249878
Serine/threonine-protein kinase TBK1 [OS=Homo sapiens]	1.002	0.998748156
Ubiquitin carboxyl-terminal hydrolase 47 [OS=Homo sapiens]	1.002	0.999998538
40S ribosomal protein S18 [OS=Homo sapiens]	1.002	0.999802286
Retinoic acid receptor RXR-beta [OS=Homo sapiens]	1.002	0.993730317
Mediator of RNA polymerase II transcription subunit 24 [OS=Homo sapiens]	1.002	0.999999133
Exosome complex component RRP42 [OS=Homo sapiens]	1.002	0.999062785
Protein SOGA1 [OS=Homo sapiens]	1.002	0.995148634
39S ribosomal protein L16, mitochondrial [OS=Homo sapiens]	1.002	0.883136968
Melanoma-associated antigen 2 [OS=Homo sapiens]	1.002	0.999273972
Homeobox protein Hox-B5 [OS=Homo sapiens]	1.002	0.857687655
Hepatocyte growth factor receptor [OS=Homo sapiens]	1.002	0.999288927
60S ribosomal protein L4 [OS=Homo sapiens]	1.003	0.988737613
Host cell factor 1 [OS=Homo sapiens]	1.003	0.999997676
Ubiquilin-1 [OS=Homo sapiens]	1.003	0.97667535
Malignant T-cell-amplified sequence 2 [OS=Homo sapiens]	1.003	0.999788608
Geranylgeranyl transferase type-2 subunit alpha [OS=Homo sapiens]	1.003	0.997298471
Lipoma-preferred partner [OS=Homo sapiens]	1.003	0.998610046
Protein CutA [OS=Homo sapiens]	1.003	0.99275239
RAD50-interacting protein 1 [OS=Homo sapiens]	1.003	0.955765256
Mitogen-activated protein kinase kinase kinase 20 [OS=Homo sapiens]	1.003	0.998041443
39S ribosomal protein L17, mitochondrial [OS=Homo sapiens]	1.003	0.999999993
Protein farnesyltransferase subunit beta [OS=Homo sapiens]	1.003	0.996140485
HLA class I histocompatibility antigen, alpha chain E [OS=Homo sapiens]	1.003	0.926944414
Charged multivesicular body protein 7 [OS=Homo sapiens]	1.003	0.999097141
Proline-rich protein PRCC [OS=Homo sapiens]	1.003	0.999998795
Zinc finger protein 528 [OS=Homo sapiens]	1.003	0.990247595
Formin-1 [OS=Homo sapiens]	1.003	0.996707342
Protein GOLM2 [OS=Homo sapiens]	1.003	0.839716854
Glucose-6-phosphate isomerase [OS=Homo sapiens]	1.004	0.98395771
Eukaryotic translation initiation factor 3 subunit C-like protein [OS=Homo sapier	1.004	0.998726383
Sodium/potassium-transporting ATPase subunit alpha-2 [OS=Homo sapiens]	1.004	0.98458402
40S ribosomal protein S14 [OS=Homo sapiens]	1.004	0.987278773
60S ribosomal protein L23 [OS=Homo sapiens]	1.004	0.938193893
Elongation factor-like GTPase 1 [OS=Homo sapiens]	1.004	0.934449822
Translin-associated protein X [OS=Homo sapiens]	1.004	0.999859471
ATP-dependent RNA helicase DDX55 [OS=Homo sapiens]	1.004	0.999928293
Ras-related protein Rab-6B [OS=Homo sapiens]	1.004	0.999612371
Centrosomal protein 43 [OS=Homo sapiens]	1.004	0.993409267
Protein bicaudal D homolog 1 [OS=Homo sapiens]	1.004	0.995772164

Centrosomal protein of 76 kDa [OS=Homo sapiens]	1.004	0.999998144
Arf-GAP with SH3 domain, ANK repeat and PH domain-containing protein 3 [OS=	1.004	0.89284233
Protein adenylyltransferase SelO, mitochondrial [OS=Homo sapiens]	1.004	0.762287321
Probable phospholipid-transporting ATPase IIB [OS=Homo sapiens]	1.004	1
DNA excision repair protein ERCC-8 [OS=Homo sapiens]	1.004	0.999892536
Filamin-B [OS=Homo sapiens]	1.005	0.845679128
Ras GTPase-activating-like protein IQGAP1 [OS=Homo sapiens]	1.005	1
Heat shock cognate 71 kDa protein [OS=Homo sapiens]	1.005	0.949515414
T-complex protein 1 subunit gamma [OS=Homo sapiens]	1.005	0.999998861
CAP-Gly domain-containing linker protein 2 [OS=Homo sapiens]	1.005	0.999341893
26S proteasome non-ATPase regulatory subunit 7 [OS=Homo sapiens]	1.005	0.997752747
E3 ubiquitin-protein ligase TRIM33 [OS=Homo sapiens]	1.005	0.991826675
Casein kinase I isoform alpha [OS=Homo sapiens]	1.005	0.988833682
Zinc finger CCCH domain-containing protein 4 [OS=Homo sapiens]	1.005	0.99980166
Alpha-soluble NSF attachment protein [OS=Homo sapiens]	1.005	0.995962144
Transcriptional repressor protein YY1 [OS=Homo sapiens]	1.005	0.999911316
SLIT-ROBO Rho GTPase-activating protein 2 [OS=Homo sapiens]	1.005	0.812372229
Thyroid adenoma-associated protein [OS=Homo sapiens]	1.005	0.998593726
Probable 18S rRNA (guanine-N(7))-methyltransferase [OS=Homo sapiens]	1.005	0.992482371
Migration and invasion enhancer 1 [OS=Homo sapiens]	1.005	0.999962529
Cyclic AMP-dependent transcription factor ATF-7 [OS=Homo sapiens]	1.005	0.990646795
Epidermal growth factor receptor kinase substrate 8 [OS=Homo sapiens]	1.005	0.999999986
Securin [OS=Homo sapiens]	1.005	0.99999354
Tetratricopeptide repeat protein 21B [OS=Homo sapiens]	1.005	0.999711199
ER degradation-enhancing alpha-mannosidase-like protein 2 [OS=Homo sapien	1.005	0.999984699
Carboxy-terminal domain RNA polymerase II polypeptide A small phosphatase 1	1.005	0.968275497
Glycogen phosphorylase, liver form [OS=Homo sapiens]	1.006	0.962382969
Peptidyl-prolyl cis-trans isomerase FKBP4 [OS=Homo sapiens]	1.006	0.998792252
Centrosomal protein of 170 kDa [OS=Homo sapiens]	1.006	0.671791089
Transcription factor BTF3 [OS=Homo sapiens]	1.006	0.992930458
Anaphase-promoting complex subunit 1 [OS=Homo sapiens]	1.006	0.886603845
Regulation of nuclear pre-mRNA domain-containing protein 1B [OS=Homo sapie	1.006	0.962056688
TRMT1-like protein [OS=Homo sapiens]	1.006	0.975418026
Tubulin-specific chaperone A [OS=Homo sapiens]	1.006	0.999995363
Epidermal growth factor receptor kinase substrate 8-like protein 2 [OS=Homo sa	1.006	0.996726653
Bromodomain-containing protein 2 [OS=Homo sapiens]	1.006	0.999883336
Ensconsin [OS=Homo sapiens]	1.006	0.998958473
Protein MIX23 [OS=Homo sapiens]	1.006	0.981734653
Isoaspartyl peptidase/L-asparaginase [OS=Homo sapiens]	1.006	0.9916953
Tubulin beta-6 chain [OS=Homo sapiens]	1.007	0.99388123
AP-3 complex subunit beta-1 [OS=Homo sapiens]	1.007	0.691562708
2-oxoglutarate dehydrogenase complex component E1 [OS=Homo sapiens]	1.007	0.999753321
26S proteasome non-ATPase regulatory subunit 3 [OS=Homo sapiens]	1.007	0.981158699
Probable ATP-dependent RNA helicase DDX6 [OS=Homo sapiens]	1.007	0.999999931
28S ribosomal protein S29, mitochondrial [OS=Homo sapiens]	1.007	0.999116271
Synapse-associated protein 1 [OS=Homo sapiens]	1.007	0.986933017
Bromodomain-containing protein 4 [OS=Homo sapiens]	1.007	0.999981548
Protein PRRC2B [OS=Homo sapiens]	1.007	0.988293239
Protein phosphatase 1 regulatory subunit 14A [OS=Homo sapiens]	1.007	0.971869258
Cell division cycle protein 123 homolog [OS=Homo sapiens]	1.007	0.999688272
Probable E3 ubiquitin-protein ligase HERC1 [OS=Homo sapiens]	1.007	0.942019489
Katanin p60 ATPase-containing subunit A1 [OS=Homo sapiens]	1.007	0.999958982
Pre-mRNA cleavage complex 2 protein Pcf11 [OS=Homo sapiens]	1.007	0.999999885
Proteasome assembly chaperone 3 [OS=Homo sapiens]	1.007	0.999987657
Ketosamine-3-kinase [OS=Homo sapiens]	1.007	0.968097087
39S ribosomal protein L40, mitochondrial [OS=Homo sapiens]	1.007	0.990197637
E3 ubiquitin-protein ligase DTX3L [OS=Homo sapiens]	1.007	0.979362514
Phospholipase D1 [OS=Homo sapiens]	1.007	0.967742151
Protein spire homolog 1 [OS=Homo sapiens]	1.007	0.999157718
Mediator of RNA polymerase II transcription subunit 22 [OS=Homo sapiens]	1.007	0.999999004
Large neutral amino acids transporter small subunit 4 [OS=Homo sapiens]	1.007	0.997424705
Mediator of RNA polymerase II transcription subunit 30 [OS=Homo sapiens]	1.007	0.999706207
Trypsin-3 [OS=Homo sapiens]	1.007	0.998973479
Meiosis-specific coiled-coil domain-containing protein MEIOC [OS=Homo sapie	1.007	0.998580562
Cytochrome c oxidase subunit 7B, mitochondrial [OS=Homo sapiens]	1.007	0.996476675
Torsin-1A-interacting protein 1 [OS=Homo sapiens]	1.008	0.999986061
Ribosome maturation protein SBDS [OS=Homo sapiens]	1.008	0.999430879
Beta-enolase [OS=Homo sapiens]	1.008	0.933800617
S-formylglutathione hydrolase [OS=Homo sapiens]	1.008	0.987832045
Abl interactor 1 [OS=Homo sapiens]	1.008	0.999651033
GTPase Era, mitochondrial [OS=Homo sapiens]	1.008	1
Enhancer of mRNA-decapping protein 3 [OS=Homo sapiens]	1.008	0.999999523
Serine/arginine-rich splicing factor 11 [OS=Homo sapiens]	1.008	0.969636213
NEDD8 ultimate buster 1 [OS=Homo sapiens]	1.008	0.997432519
Leukocyte receptor cluster member 8 [OS=Homo sapiens]	1.008	0.998922191

Copine-8 [OS=Homo sapiens]	1.008	0.640686216
SWI/SNF-related matrix-associated actin-dependent regulator of chromatin sub	1.008	0.999999987
Succinate dehydrogenase assembly factor 4, mitochondrial [OS=Homo sapiens]	1.008	0.999999839
Optic atrophy 3 protein [OS=Homo sapiens]	1.008	0.999991524
Signal transducer and activator of transcription 6 [OS=Homo sapiens]	1.008	0.999979811
Ganglioside-induced differentiation-associated protein 2 [OS=Homo sapiens]	1.008	0.999568516
AP-1 complex subunit sigma-3 [OS=Homo sapiens]	1.008	0.99999395
Glutathione S-transferase theta-2 [OS=Homo sapiens]	1.008	0.95643508
Succinate dehydrogenase [ubiquinone] flavoprotein subunit, mitochondrial [OS=	1.009	0.999999995
Malate dehydrogenase, cytoplasmic [OS=Homo sapiens]	1.009	0.999105694
Pre-mRNA-splicing factor ATP-dependent RNA helicase DHX16 [OS=Homo sapie	1.009	0.999763796
Caprin-1 [OS=Homo sapiens]	1.009	0.998153819
eIF5-mimic protein 2 [OS=Homo sapiens]	1.009	0.967233182
60S ribosomal protein L6 [OS=Homo sapiens]	1.009	0.999975478
G-rich sequence factor 1 [OS=Homo sapiens]	1.009	0.99677008
CREB-binding protein [OS=Homo sapiens]	1.009	0.833856092
CDK5 regulatory subunit-associated protein 3 [OS=Homo sapiens]	1.009	0.99998887
NACHT, LRR and PYD domains-containing protein 2 [OS=Homo sapiens]	1.009	0.995498566
Torsin-1A [OS=Homo sapiens]	1.009	0.99996728
Immunoglobulin-binding protein 1 [OS=Homo sapiens]	1.009	0.992836433
DNA-directed RNA polymerase II subunit RPB9 [OS=Homo sapiens]	1.009	0.948879397
Calcineurin subunit B type 1 [OS=Homo sapiens]	1.009	0.953906852
Interferon-induced transmembrane protein 3 [OS=Homo sapiens]	1.009	0.999965571
Zinc finger protein 467 [OS=Homo sapiens]	1.009	0.999765336
Zinc finger protein 787 [OS=Homo sapiens]	1.009	0.933576789
Sodium channel modifier 1 [OS=Homo sapiens]	1.009	1
Peroxisomal membrane protein PEX13 [OS=Homo sapiens]	1.009	1
Rab GDP dissociation inhibitor alpha [OS=Homo sapiens]	1.01	0.999984638
Polyadenylate-binding protein 4 [OS=Homo sapiens]	1.01	0.667840703
RNA polymerase-associated protein RTF1 homolog [OS=Homo sapiens]	1.01	0.999994646
DnaJ homolog subfamily C member 8 [OS=Homo sapiens]	1.01	0.995946234
Importin-11 [OS=Homo sapiens]	1.01	0.875117875
Methionine aminopeptidase 2 [OS=Homo sapiens]	1.01	0.989159556
CAAX prenyl protease 1 homolog [OS=Homo sapiens]	1.01	0.970368426
Glycerol-3-phosphate dehydrogenase, mitochondrial [OS=Homo sapiens]	1.01	0.999924787
Syndetin [OS=Homo sapiens]	1.01	0.999849109
Adhesion G protein-coupled receptor E5 [OS=Homo sapiens]	1.01	0.999985791
HCLS1-associated protein X-1 [OS=Homo sapiens]	1.01	0.999999882
Glutamyl-tRNA(Gln) amidotransferase subunit A, mitochondrial [OS=Homo sapi	1.01	0.992112837
39S ribosomal protein L32, mitochondrial [OS=Homo sapiens]	1.01	0.999989335
Glutaredoxin-related protein 5, mitochondrial [OS=Homo sapiens]	1.01	0.945769738
Neuronal-specific septin-3 [OS=Homo sapiens]	1.01	0.940454581
Vacuolar fusion protein MON1 homolog A [OS=Homo sapiens]	1.01	0.999999998
FAST kinase domain-containing protein 1, mitochondrial [OS=Homo sapiens]	1.01	0.999979246
ATP synthase subunit d, mitochondrial [OS=Homo sapiens]	1.011	0.999995817
Girdin [OS=Homo sapiens]	1.011	0.952976408
eIF5-mimic protein 1 [OS=Homo sapiens]	1.011	0.993002657
CWF19-like protein 1 [OS=Homo sapiens]	1.011	0.999131046
Ubiquitin carboxyl-terminal hydrolase 4 [OS=Homo sapiens]	1.011	0.937464801
26S proteasome non-ATPase regulatory subunit 10 [OS=Homo sapiens]	1.011	0.980793027
Phosphoacetylglucosamine mutase [OS=Homo sapiens]	1.011	0.992467632
Serine palmitoyltransferase 1 [OS=Homo sapiens]	1.011	0.997390349
Growth arrest and DNA damage-inducible proteins-interacting protein 1 [OS=Ho	1.011	0.999870663
Gamma-tubulin complex component 4 [OS=Homo sapiens]	1.011	0.999996949
TNF receptor-associated factor 6 [OS=Homo sapiens]	1.011	0.999841342
Rab GTPase-activating protein 1-like [OS=Homo sapiens]	1.011	0.999978191
Proteasome assembly chaperone 4 [OS=Homo sapiens]	1.011	0.998730957
Cyclin-C [OS=Homo sapiens]	1.011	0.819425399
Signal transducer and activator of transcription 1-alpha/beta [OS=Homo sapiens	1.012	0.902971456
Splicing factor 1 [OS=Homo sapiens]	1.012	0.998153657
Ran-binding protein 3 [OS=Homo sapiens]	1.012	0.977424819
Pre-mRNA-splicing factor ATP-dependent RNA helicase PRP16 [OS=Homo sapie	1.012	0.996938586
A-kinase anchor protein 12 [OS=Homo sapiens]	1.012	0.99999999
Rab11 family-interacting protein 1 [OS=Homo sapiens]	1.012	0.999991423
Ras-related protein Rab-22A [OS=Homo sapiens]	1.012	0.999999979
Transmembrane protein 109 [OS=Homo sapiens]	1.012	0.99982914
RWD domain-containing protein 1 [OS=Homo sapiens]	1.012	0.990190165
Vacuolar protein sorting-associated protein 52 homolog [OS=Homo sapiens]	1.012	0.999841486
Surfeit locus protein 2 [OS=Homo sapiens]	1.012	0.999999923
Integrator complex subunit 9 [OS=Homo sapiens]	1.012	0.756148217
Syntenin-1 [OS=Homo sapiens]	1.012	0.99978437
Mitotic-spindle organizing protein 2B [OS=Homo sapiens]	1.012	0.902474454
Kelch domain-containing protein 2 [OS=Homo sapiens]	1.012	0.999998964
Syndecan-2 [OS=Homo sapiens]	1.012	0.999987119
Guanine nucleotide-binding protein G(I)/G(S)/G(O) subunit gamma-5 [OS=Homo	1.012	0.999999974

Calcium uptake protein 2, mitochondrial [OS=Homo sapiens]	1.012	0.999977429
L-lactate dehydrogenase A chain [OS=Homo sapiens]	1.013	0.999999364
Probable ATP-dependent RNA helicase DDX46 [OS=Homo sapiens]	1.013	0.766434152
14-3-3 protein beta/alpha [OS=Homo sapiens]	1.013	0.98774518
ATP-binding cassette sub-family F member 2 [OS=Homo sapiens]	1.013	0.962097058
Nuclear factor NF-kappa-B p100 subunit [OS=Homo sapiens]	1.013	0.935964127
Eukaryotic translation initiation factor 2A [OS=Homo sapiens]	1.013	0.995107463
28S ribosomal protein S9, mitochondrial [OS=Homo sapiens]	1.013	0.991262807
RNA-binding protein FXR1 [OS=Homo sapiens]	1.013	0.847791068
Eukaryotic translation initiation factor 6 [OS=Homo sapiens]	1.013	0.999980171
ADP-ribosylation factor 6 [OS=Homo sapiens]	1.013	0.991980176
CUB domain-containing protein 1 [OS=Homo sapiens]	1.013	1
28S ribosomal protein S7, mitochondrial [OS=Homo sapiens]	1.013	0.996928601
Tyrosine-protein kinase ABL1 [OS=Homo sapiens]	1.013	0.983655277
RNA N6-adenosine-methyltransferase METTL16 [OS=Homo sapiens]	1.013	0.997634598
Target of EGR1 protein 1 [OS=Homo sapiens]	1.013	0.999999754
Tether containing UBX domain for GLUT4 [OS=Homo sapiens]	1.013	0.999999995
Inactive rhomboid protein 2 [OS=Homo sapiens]	1.013	0.999987385
Transmembrane protein 104 [OS=Homo sapiens]	1.013	0.999996261
STING ER exit protein [OS=Homo sapiens]	1.013	0.925228684
DnaJ homolog subfamily B member 5 [OS=Homo sapiens]	1.013	0.999617041
60 kDa heat shock protein, mitochondrial [OS=Homo sapiens]	1.014	0.926721897
Talin-2 [OS=Homo sapiens]	1.014	0.961617896
RNA polymerase II-associated protein 3 [OS=Homo sapiens]	1.014	0.955895096
von Willebrand factor A domain-containing protein 8 [OS=Homo sapiens]	1.014	0.910297324
Translation initiation factor eIF-2B subunit epsilon [OS=Homo sapiens]	1.014	0.903260624
HEAT repeat-containing protein 6 [OS=Homo sapiens]	1.014	0.999433853
E3 ubiquitin-protein ligase TRIM32 [OS=Homo sapiens]	1.014	0.999999968
Osteopetrosis-associated transmembrane protein 1 [OS=Homo sapiens]	1.014	0.999983517
DNA topoisomerase 3-beta-1 [OS=Homo sapiens]	1.014	0.977635918
IQ motif and SEC7 domain-containing protein 1 [OS=Homo sapiens]	1.014	0.990162343
F-box/LRR-repeat protein 20 [OS=Homo sapiens]	1.014	0.89110418
RAB6-interacting golgin [OS=Homo sapiens]	1.014	0.997401588
E3 ubiquitin-protein ligase TRIM13 [OS=Homo sapiens]	1.014	0.999999155
Leucine-zipper-like transcriptional regulator 1 [OS=Homo sapiens]	1.014	0.97647198
DNA-dependent protein kinase catalytic subunit [OS=Homo sapiens]	1.015	0.906709536
Fatty acid synthase [OS=Homo sapiens]	1.015	0.987498499
ATP-citrate synthase [OS=Homo sapiens]	1.015	0.964842314
ATP-binding cassette sub-family F member 1 [OS=Homo sapiens]	1.015	0.999005007
Methionine adenosyltransferase 2 subunit beta [OS=Homo sapiens]	1.015	0.973774062
Cleavage and polyadenylation specificity factor subunit 7 [OS=Homo sapiens]	1.015	0.988939074
Uncharacterized protein C7orf50 [OS=Homo sapiens]	1.015	0.957287271
Sorting nexin-17 [OS=Homo sapiens]	1.015	0.999727865
Melanoma-associated antigen 10 [OS=Homo sapiens]	1.015	0.996857067
28S ribosomal protein S28, mitochondrial [OS=Homo sapiens]	1.015	0.999999735
Mitochondrial potassium channel [OS=Homo sapiens]	1.015	0.995385479
Acyl-coenzyme A thioesterase THEM4 [OS=Homo sapiens]	1.015	0.80341795
Telomerase-binding protein EST1A [OS=Homo sapiens]	1.015	0.570978026
Peroxisomal membrane protein PMP34 [OS=Homo sapiens]	1.015	0.999999996
FACT complex subunit SPT16 [OS=Homo sapiens]	1.016	0.940174279
Signal recognition particle subunit SRP72 [OS=Homo sapiens]	1.016	0.99881975
Clathrin interactor 1 [OS=Homo sapiens]	1.016	0.863375474
Endothelin-converting enzyme 1 [OS=Homo sapiens]	1.016	0.980968832
Alcohol dehydrogenase class-3 [OS=Homo sapiens]	1.016	0.999853054
L-aminoadipate-semialdehyde dehydrogenase-phosphopantetheinyl transferas	1.016	0.940228211
Translocation protein SEC62 [OS=Homo sapiens]	1.016	0.98200424
Thioredoxin-like protein 4B [OS=Homo sapiens]	1.016	0.999979144
Tripartite motif-containing protein 65 [OS=Homo sapiens]	1.016	0.999922298
Pantothenate kinase 3 [OS=Homo sapiens]	1.016	0.970165731
Syntaxin-binding protein 4 [OS=Homo sapiens]	1.016	0.998747238
Transmembrane protein 59 [OS=Homo sapiens]	1.016	0.996285087
Integrin alpha-E [OS=Homo sapiens]	1.016	0.995095503
Multimerin-2 [OS=Homo sapiens]	1.016	0.94597161
Golgin subfamily A member 3 [OS=Homo sapiens]	1.017	0.886200257
Poly(U)-binding-splicing factor PUF60 [OS=Homo sapiens]	1.017	0.925209034
Shootin-1 [OS=Homo sapiens]	1.017	0.968779128
Paxillin [OS=Homo sapiens]	1.017	0.904872174
60S ribosomal protein L7 [OS=Homo sapiens]	1.017	0.953146365
Inverted formin-2 [OS=Homo sapiens]	1.017	0.999997357
Protein phosphatase methylesterase 1 [OS=Homo sapiens]	1.017	0.930655148
Syntaxin-binding protein 3 [OS=Homo sapiens]	1.017	0.999998731
Leucine-rich repeat-containing protein 47 [OS=Homo sapiens]	1.017	0.999999959
Hepatocyte growth factor-regulated tyrosine kinase substrate [OS=Homo sapier	1.017	0.998612637
U6 snRNA-associated Sm-like protein LSm7 [OS=Homo sapiens]	1.017	0.887393776
Protein phosphatase 3 catalytic subunit alpha [OS=Homo sapiens]	1.017	0.913337223

DNA-directed RNA polymerase III subunit RPC2 [OS=Homo sapiens]	1.017	0.958604598
SLC35A4 upstream open reading frame protein [OS=Homo sapiens]	1.017	0.89284264
Probable RNA-binding protein EIF1AD [OS=Homo sapiens]	1.017	0.999999982
Dual specificity protein phosphatase 23 [OS=Homo sapiens]	1.017	0.999995291
Ferroptosis suppressor protein 1 [OS=Homo sapiens]	1.017	0.965860258
Methyl-CpG-binding domain protein 2 [OS=Homo sapiens]	1.017	0.940801597
Cell surface glycoprotein MUC18 [OS=Homo sapiens]	1.017	0.999999995
Growth factor receptor-bound protein 10 [OS=Homo sapiens]	1.017	0.985766793
KAT8 regulatory NSL complex subunit 2 [OS=Homo sapiens]	1.017	0.999991839
M-phase inducer phosphatase 2 [OS=Homo sapiens]	1.017	0.990615293
Protein AHNAK2 [OS=Homo sapiens]	1.018	0.998709275
60S ribosomal protein L3 [OS=Homo sapiens]	1.018	0.830027407
60S ribosomal protein L10a [OS=Homo sapiens]	1.018	0.979169909
Caseinolytic peptidase B protein homolog [OS=Homo sapiens]	1.018	0.982152417
Dolichol-phosphate mannosyltransferase subunit 1 [OS=Homo sapiens]	1.018	0.999433254
Serine hydroxymethyltransferase, cytosolic [OS=Homo sapiens]	1.018	0.999998179
Serine/threonine-protein kinase 38 [OS=Homo sapiens]	1.018	0.878116741
NFU1 iron-sulfur cluster scaffold homolog, mitochondrial [OS=Homo sapiens]	1.018	0.910664507
Microtubule-associated protein 1A [OS=Homo sapiens]	1.018	0.999966651
39S ribosomal protein L2, mitochondrial [OS=Homo sapiens]	1.018	0.999999656
Protein THEMIS2 [OS=Homo sapiens]	1.018	0.999999562
39S ribosomal protein L20, mitochondrial [OS=Homo sapiens]	1.018	0.998844732
Stromal membrane-associated protein 2 [OS=Homo sapiens]	1.018	0.999967407
mRNA-decapping enzyme 1A [OS=Homo sapiens]	1.018	0.99992947
STE20-related kinase adapter protein alpha [OS=Homo sapiens]	1.018	0.951388602
Nuclear migration protein nudC [OS=Homo sapiens]	1.019	0.998761506
Eukaryotic translation initiation factor 3 subunit E [OS=Homo sapiens]	1.019	0.999980537
26S proteasome regulatory subunit 6B [OS=Homo sapiens]	1.019	0.730313141
Prefoldin subunit 3 [OS=Homo sapiens]	1.019	0.99939269
Pachytene checkpoint protein 2 homolog [OS=Homo sapiens]	1.019	0.999998892
Phosphoribosyl pyrophosphate synthase-associated protein 1 [OS=Homo sapie	1.019	0.927492477
Zinc finger protein ubi-d4 [OS=Homo sapiens]	1.019	0.843827726
UPF0696 protein C11orf68 [OS=Homo sapiens]	1.019	0.999988011
Alpha-globin transcription factor CP2 [OS=Homo sapiens]	1.019	0.99957374
Checkpoint protein HUS1 [OS=Homo sapiens]	1.019	0.926184306
CBP80/20-dependent translation initiation factor [OS=Homo sapiens]	1.019	0.997924644
Vesicle transport through interaction with t-SNAREs homolog 1A [OS=Homo sap	1.019	0.996608767
EP300-interacting inhibitor of differentiation 2 [OS=Homo sapiens]	1.019	0.9232345
Cytoplasmic dynein 1 heavy chain 1 [OS=Homo sapiens]	1.02	0.999994629
Tryptophan--tRNA ligase, cytoplasmic [OS=Homo sapiens]	1.02	0.975385694
SKI2 subunit of superkiller complex protein [OS=Homo sapiens]	1.02	0.824134859
Histidine--tRNA ligase, mitochondrial [OS=Homo sapiens]	1.02	0.977560207
40S ribosomal protein S15a [OS=Homo sapiens]	1.02	0.99770043
Transcriptional repressor p66-alpha [OS=Homo sapiens]	1.02	0.998134487
Prefoldin subunit 6 [OS=Homo sapiens]	1.02	0.996415704
60S ribosomal protein L28 [OS=Homo sapiens]	1.02	0.978692114
Ras-related protein Rab-31 [OS=Homo sapiens]	1.02	0.917283676
Transducin-like enhancer protein 3 [OS=Homo sapiens]	1.02	0.875437029
Striatin-interacting protein 1 [OS=Homo sapiens]	1.02	0.846637513
Melanoma-associated antigen 3 [OS=Homo sapiens]	1.02	0.999998746
DIS3-like exonuclease 2 [OS=Homo sapiens]	1.02	0.916626231
Regulatory-associated protein of mTOR [OS=Homo sapiens]	1.02	0.963946731
Netrin-1 [OS=Homo sapiens]	1.02	0.766278489
Elongation factor 2 [OS=Homo sapiens]	1.021	0.978203621
Rho-associated protein kinase 2 [OS=Homo sapiens]	1.021	0.841626051
Catalase [OS=Homo sapiens]	1.021	0.874780714
Chromodomain-helicase-DNA-binding protein 1-like [OS=Homo sapiens]	1.021	0.83385052
Deubiquitinating protein VCPIP1 [OS=Homo sapiens]	1.021	0.912867266
Gamma-tubulin complex component 2 [OS=Homo sapiens]	1.021	0.847496023
Protein Spindly [OS=Homo sapiens]	1.021	0.983654267
Dynein light chain roadblock-type 1 [OS=Homo sapiens]	1.021	0.915053938
Glutamate--cysteine ligase regulatory subunit [OS=Homo sapiens]	1.021	0.733048122
WD repeat-containing protein 82 [OS=Homo sapiens]	1.021	0.993064776
Heme-binding protein 2 [OS=Homo sapiens]	1.021	0.999618315
28S ribosomal protein S15, mitochondrial [OS=Homo sapiens]	1.021	0.997492869
ATP synthase-coupling factor 6, mitochondrial [OS=Homo sapiens]	1.021	0.999280926
Ubiquitin-conjugating enzyme E2 Z [OS=Homo sapiens]	1.021	0.999859797
Testis-specific Y-encoded-like protein 4 [OS=Homo sapiens]	1.021	0.999997943
Mth938 domain-containing protein [OS=Homo sapiens]	1.021	0.930284446
Bis(5'-nucleosyl)-tetraphosphatase [asymmetrical] [OS=Homo sapiens]	1.021	0.999997214
Retrotransposon-derived protein PEG10 [OS=Homo sapiens]	1.021	0.999945939
ER membrane protein complex subunit 10 [OS=Homo sapiens]	1.021	0.999996836
Phosphatidate phosphatase LPIN1 [OS=Homo sapiens]	1.021	0.932504489
Zinc finger protein 608 [OS=Homo sapiens]	1.021	0.999954899
Fibronectin type III and SPRY domain-containing protein 1 [OS=Homo sapiens]	1.021	0.983822745

Exocyst complex component 3-like protein 4 [OS=Homo sapiens]	1.021	0.935564662
Vacuolar protein sorting-associated protein 35 [OS=Homo sapiens]	1.022	0.685915713
DnaJ homolog subfamily A member 1 [OS=Homo sapiens]	1.022	0.998496508
Signal recognition particle subunit SRP54 [OS=Homo sapiens]	1.022	0.85827552
Transportin-2 [OS=Homo sapiens]	1.022	0.597659611
Importin subunit alpha-5 [OS=Homo sapiens]	1.022	0.978351973
Clathrin light chain B [OS=Homo sapiens]	1.022	0.935284465
Ubiquitin carboxyl-terminal hydrolase 16 [OS=Homo sapiens]	1.022	0.999325804
Caspase-7 [OS=Homo sapiens]	1.022	0.803790083
Peroxisomal biogenesis factor 3 [OS=Homo sapiens]	1.022	0.999999596
Tripartite motif-containing protein 5 [OS=Homo sapiens]	1.022	0.989944621
Mediator of RNA polymerase II transcription subunit 21 [OS=Homo sapiens]	1.022	0.945312893
Cytochrome c oxidase copper chaperone [OS=Homo sapiens]	1.022	0.996211465
Transmembrane and ubiquitin-like domain-containing protein 1 [OS=Homo sapi	1.022	0.999999999
Centrosome-associated protein 350 [OS=Homo sapiens]	1.022	0.999972997
Protein zyg-11 homolog B [OS=Homo sapiens]	1.022	0.999979474
Phospholipid phosphatase 2 [OS=Homo sapiens]	1.022	0.999971397
Nischarin [OS=Homo sapiens]	1.022	0.999769396
Alpha-enolase [OS=Homo sapiens]	1.023	0.901529422
26S proteasome regulatory subunit 7 [OS=Homo sapiens]	1.023	0.835581872
Dynamin-like 120 kDa protein, mitochondrial [OS=Homo sapiens]	1.023	0.999997041
Monofunctional C1-tetrahydrofolate synthase, mitochondrial [OS=Homo sapien	1.023	0.975843522
Switch-associated protein 70 [OS=Homo sapiens]	1.023	0.999067549
SRSF protein kinase 1 [OS=Homo sapiens]	1.023	0.664598163
Eukaryotic translation initiation factor 3 subunit K [OS=Homo sapiens]	1.023	0.991502662
Small glutamine-rich tetratricopeptide repeat-containing protein alpha [OS=Hor	1.023	0.996804318
ATP synthase F(0) complex subunit B1, mitochondrial [OS=Homo sapiens]	1.023	0.999995809
Fatty acid CoA ligase Acsl3 [OS=Homo sapiens]	1.023	0.802049298
60S ribosomal protein L13 [OS=Homo sapiens]	1.023	0.991971909
60S ribosomal protein L10 [OS=Homo sapiens]	1.023	0.843117003
60S ribosomal protein L9 [OS=Homo sapiens]	1.023	0.970438688
Kinesin-like protein KIF2A [OS=Homo sapiens]	1.023	0.808283005
Elongin-A [OS=Homo sapiens]	1.023	0.626353916
Ubiquitin carboxyl-terminal hydrolase 19 [OS=Homo sapiens]	1.023	0.918585309
Sepiapterin reductase [OS=Homo sapiens]	1.023	0.912418784
Mortality factor 4-like protein 2 [OS=Homo sapiens]	1.023	0.999391562
CCR4-NOT transcription complex subunit 10 [OS=Homo sapiens]	1.023	0.995904964
Enoyl-CoA delta isomerase 2 [OS=Homo sapiens]	1.023	0.907542881
AP-4 complex subunit epsilon-1 [OS=Homo sapiens]	1.023	0.976193118
4'-phosphopantetheine phosphatase [OS=Homo sapiens]	1.023	0.999973148
Zinc finger FYVE domain-containing protein 1 [OS=Homo sapiens]	1.023	0.977846141
Distal membrane-arm assembly complex protein 2 [OS=Homo sapiens]	1.023	0.999899004
Xyloside xylosyltransferase 1 [OS=Homo sapiens]	1.023	0.998189288
Xenotropic and polytropic retrovirus receptor 1 [OS=Homo sapiens]	1.023	0.999994749
Exportin-1 [OS=Homo sapiens]	1.024	0.97756669
Poly(rC)-binding protein 2 [OS=Homo sapiens]	1.024	0.324926246
Keratin, type I cytoskeletal 10 [OS=Homo sapiens]	1.024	0.99998181
E3 ubiquitin-protein ligase ZNF598 [OS=Homo sapiens]	1.024	0.794381382
PAT complex subunit CCDC47 [OS=Homo sapiens]	1.024	0.998361086
Sodium/potassium-transporting ATPase subunit beta-1 [OS=Homo sapiens]	1.024	0.999900404
Prefoldin subunit 2 [OS=Homo sapiens]	1.024	0.987482203
Ribosome-recycling factor, mitochondrial [OS=Homo sapiens]	1.024	0.982264252
CD9 antigen [OS=Homo sapiens]	1.024	0.999993573
39S ribosomal protein L10, mitochondrial [OS=Homo sapiens]	1.024	0.992217231
Protein lin-37 homolog [OS=Homo sapiens]	1.024	0.999727618
Palladin [OS=Homo sapiens]	1.024	0.99918523
Programmed cell death 6-interacting protein [OS=Homo sapiens]	1.025	0.962664048
Protein PRRC2C [OS=Homo sapiens]	1.025	0.72057028
Aspartate--tRNA ligase, cytoplasmic [OS=Homo sapiens]	1.025	0.995566039
Inosine-5'-monophosphate dehydrogenase 2 [OS=Homo sapiens]	1.025	0.99997188
ATP-dependent RNA helicase DDX1 [OS=Homo sapiens]	1.025	0.925878903
Transcription elongation factor A protein 1 [OS=Homo sapiens]	1.025	0.993594407
GTP-binding nuclear protein Ran [OS=Homo sapiens]	1.025	0.705199592
Myosin light polypeptide 6 [OS=Homo sapiens]	1.025	0.998714581
Regulator of G-protein signaling 10 [OS=Homo sapiens]	1.025	0.999999999
Sperm-associated antigen 7 [OS=Homo sapiens]	1.025	0.986271374
Ubiquitin-40S ribosomal protein S27a [OS=Homo sapiens]	1.025	0.999999238
Eukaryotic translation initiation factor 1 [OS=Homo sapiens]	1.025	0.937998816
26S proteasome non-ATPase regulatory subunit 8 [OS=Homo sapiens]	1.025	0.979535271
NAD(+) hydrolase SARM1 [OS=Homo sapiens]	1.025	0.999385176
Transcription elongation factor A protein-like 3 [OS=Homo sapiens]	1.025	0.842855651
Transient receptor potential cation channel subfamily V member 2 [OS=Homo sa	1.025	0.999998691
Regulator of MON1-CCZ1 complex [OS=Homo sapiens]	1.025	0.742729906
Gem-associated protein 2 [OS=Homo sapiens]	1.025	0.641576675
Zinc finger protein 40 [OS=Homo sapiens]	1.025	0.992395395

X-ray repair cross-complementing protein 5 [OS=Homo sapiens]	1.026	0.987734205
Septin-9 [OS=Homo sapiens]	1.026	0.567574757
Nuclear cap-binding protein subunit 1 [OS=Homo sapiens]	1.026	0.993627659
SR-related and CTD-associated factor 8 [OS=Homo sapiens]	1.026	0.406329329
60S ribosomal protein L12 [OS=Homo sapiens]	1.026	0.844253631
Proteasome assembly chaperone 1 [OS=Homo sapiens]	1.026	0.871431206
Protein ILRUN [OS=Homo sapiens]	1.026	0.998757516
Ras-related protein Rap-2c [OS=Homo sapiens]	1.026	0.999999999
mRNA decay activator protein ZFP36L2 [OS=Homo sapiens]	1.026	0.999967571
NCK-interacting protein with SH3 domain [OS=Homo sapiens]	1.026	0.99222959
Ornithine decarboxylase [OS=Homo sapiens]	1.026	0.975047789
Cytochrome c oxidase assembly protein COX14 [OS=Homo sapiens]	1.026	0.99863481
N-glycosylase/DNA lyase [OS=Homo sapiens]	1.026	0.999990641
26S proteasome regulatory subunit 6A [OS=Homo sapiens]	1.027	0.711684397
Dual specificity protein phosphatase 3 [OS=Homo sapiens]	1.027	0.828441315
Succinate--CoA ligase [ADP/GDP-forming] subunit alpha, mitochondrial [OS=Ho	1.027	0.87565151
Ribosomal protein S6 kinase beta-1 [OS=Homo sapiens]	1.027	0.942951139
Peroxiredoxin-2 [OS=Homo sapiens]	1.027	0.997122676
Pumilio homolog 2 [OS=Homo sapiens]	1.027	0.999989908
Dual specificity tyrosine-phosphorylation-regulated kinase 1A [OS=Homo sapien	1.027	0.627924202
Coiled-coil domain-containing protein 93 [OS=Homo sapiens]	1.027	0.544325573
FHF complex subunit HOOK interacting protein 2A [OS=Homo sapiens]	1.027	0.9888842
Arginyl-tRNA--protein transferase 1 [OS=Homo sapiens]	1.027	0.991598496
Tax1-binding protein 3 [OS=Homo sapiens]	1.027	0.94767464
Dystonin [OS=Homo sapiens]	1.028	0.439139965
Heterogeneous nuclear ribonucleoprotein U-like protein 1 [OS=Homo sapiens]	1.028	0.796306078
Adenylate kinase 2, mitochondrial [OS=Homo sapiens]	1.028	0.835370271
Nascent polypeptide-associated complex subunit alpha, muscle-specific form [	1.028	0.999999934
Myosin regulatory light polypeptide 9 [OS=Homo sapiens]	1.028	0.882510997
Biliverdin reductase A [OS=Homo sapiens]	1.028	0.999998392
Cysteine and glycine-rich protein 1 [OS=Homo sapiens]	1.028	0.729737898
Transmembrane protein 214 [OS=Homo sapiens]	1.028	0.990791895
BUB3-interacting and GLEBS motif-containing protein ZNF207 [OS=Homo sapien	1.028	0.997402118
Probable glutamate--tRNA ligase, mitochondrial [OS=Homo sapiens]	1.028	0.345156048
Transcription elongation factor A protein-like 4 [OS=Homo sapiens]	1.028	0.997549598
Dynein light chain 2, cytoplasmic [OS=Homo sapiens]	1.028	0.946177866
Protein strawberry notch homolog 1 [OS=Homo sapiens]	1.028	0.83230099
Guanine nucleotide-binding protein G(q) subunit alpha [OS=Homo sapiens]	1.028	0.999998351
Cellular retinoic acid-binding protein 2 [OS=Homo sapiens]	1.028	0.734599922
Protein bicaudal D homolog 2 [OS=Homo sapiens]	1.028	0.99540399
Immortalization up-regulated protein [OS=Homo sapiens]	1.028	0.859187709
COP9 signalosome complex subunit 9 [OS=Homo sapiens]	1.028	0.974019987
39S ribosomal protein L18, mitochondrial [OS=Homo sapiens]	1.028	0.805483389
E3 ubiquitin-protein transferase RMND5A [OS=Homo sapiens]	1.028	0.999866052
DnaJ homolog subfamily C member 15 [OS=Homo sapiens]	1.028	0.994907128
LYR motif-containing protein 2 [OS=Homo sapiens]	1.028	0.996945599
Tyrosine--tRNA ligase, mitochondrial [OS=Homo sapiens]	1.029	0.929605499
39S ribosomal protein L39, mitochondrial [OS=Homo sapiens]	1.029	0.999999869
Actin-histidine N-methyltransferase [OS=Homo sapiens]	1.029	0.838109622
IST1 homolog [OS=Homo sapiens]	1.029	0.993175785
Protein THEM6 [OS=Homo sapiens]	1.029	0.804628423
Cleavage and polyadenylation specificity factor subunit 5 [OS=Homo sapiens]	1.029	0.991226123
GTP-binding protein 10 [OS=Homo sapiens]	1.029	0.995344622
Retinol dehydrogenase 14 [OS=Homo sapiens]	1.029	0.999308331
2-(3-amino-3-carboxypropyl)histidine synthase subunit 1 [OS=Homo sapiens]	1.029	0.987861048
Serine/threonine-protein phosphatase 2A 55 kDa regulatory subunit B delta isofo	1.029	0.99768913
Protein jagunal homolog 1 [OS=Homo sapiens]	1.029	0.999999687
Protein mono-ADP-ribosyltransferase PARP9 [OS=Homo sapiens]	1.029	0.999928853
Protein TANC2 [OS=Homo sapiens]	1.029	0.638227032
Monocarboxylate transporter 8 [OS=Homo sapiens]	1.029	0.976993839
Intraflagellar transport protein 56 [OS=Homo sapiens]	1.029	0.998791569
Ubiquitin carboxyl-terminal hydrolase 14 [OS=Homo sapiens]	1.03	0.99442629
Transcription elongation factor SPT5 [OS=Homo sapiens]	1.03	0.718525585
Glutaminase kidney isoform, mitochondrial [OS=Homo sapiens]	1.03	0.982011476
N-alpha-acetyltransferase 10 [OS=Homo sapiens]	1.03	0.999684294
Pyrroline-5-carboxylate reductase 2 [OS=Homo sapiens]	1.03	0.870727206
Protein phosphatase 1F [OS=Homo sapiens]	1.03	0.976956119
Protein PBDC1 [OS=Homo sapiens]	1.03	0.871087526
Rho guanine nucleotide exchange factor 1 [OS=Homo sapiens]	1.03	0.994169956
DNA fragmentation factor subunit alpha [OS=Homo sapiens]	1.03	0.999999698
GTP-binding protein SAR1b [OS=Homo sapiens]	1.03	0.485918069
Ribonuclease P protein subunit p40 [OS=Homo sapiens]	1.03	0.994414693
Merlin [OS=Homo sapiens]	1.03	0.78855717
CD166 antigen [OS=Homo sapiens]	1.03	0.952114788
Regulation of nuclear pre-mRNA domain-containing protein 1A [OS=Homo sapie	1.03	0.999742172

Transmembrane protein 199 [OS=Homo sapiens]	1.03	0.994631958
7-dehydrocholesterol reductase [OS=Homo sapiens]	1.03	0.999291701
Methylcrotonoyl-CoA carboxylase subunit alpha, mitochondrial [OS=Homo sapi	1.03	0.739094428
5'-AMP-activated protein kinase subunit beta-1 [OS=Homo sapiens]	1.03	0.979983153
Mediator of RNA polymerase II transcription subunit 16 [OS=Homo sapiens]	1.03	0.784512058
Chromosome transmission fidelity protein 18 homolog [OS=Homo sapiens]	1.03	0.949537137
Phosphatidylcholine translocator ABCB4 [OS=Homo sapiens]	1.03	0.795039158
Serine/threonine-protein kinase Nek6 [OS=Homo sapiens]	1.03	0.879532472
Nicotinamide/nicotinic acid mononucleotide adenylyltransferase 1 [OS=Homo s	1.03	0.998257238
Glyceraldehyde-3-phosphate dehydrogenase [OS=Homo sapiens]	1.031	0.980700787
ATP synthase subunit alpha, mitochondrial [OS=Homo sapiens]	1.031	0.999745032
Nuclear autoantigenic sperm protein [OS=Homo sapiens]	1.031	0.997905797
Eukaryotic translation initiation factor 4 gamma 2 [OS=Homo sapiens]	1.031	0.916409792
Poly(rC)-binding protein 1 [OS=Homo sapiens]	1.031	0.967580844
Eukaryotic translation initiation factor 3 subunit G [OS=Homo sapiens]	1.031	0.914931767
F-actin-capping protein subunit alpha-1 [OS=Homo sapiens]	1.031	0.625223988
Pyridoxal kinase [OS=Homo sapiens]	1.031	0.996214892
Actin-related protein 2 [OS=Homo sapiens]	1.031	0.848684419
Atlastin-2 [OS=Homo sapiens]	1.031	0.994924597
Glutathione S-transferase LANCL1 [OS=Homo sapiens]	1.031	0.999211172
Serine/threonine-protein phosphatase 4 regulatory subunit 1 [OS=Homo sapiens	1.031	0.997891
60S ribosomal protein L34 [OS=Homo sapiens]	1.031	0.589075614
Sterol-4-alpha-carboxylate 3-dehydrogenase, decarboxylating [OS=Homo sapie	1.031	0.839353893
Translation initiation factor eIF-2B subunit beta [OS=Homo sapiens]	1.031	0.490514868
Eukaryotic translation initiation factor 4E transporter [OS=Homo sapiens]	1.031	0.998747816
cAMP-regulated phosphoprotein 19 [OS=Homo sapiens]	1.031	0.667618521
Histone chaperone ASF1A [OS=Homo sapiens]	1.031	0.959186305
Thioredoxin, mitochondrial [OS=Homo sapiens]	1.031	0.999991297
Vacuolar protein sorting-associated protein 33A [OS=Homo sapiens]	1.031	0.7473004
Guanidinoacetate N-methyltransferase [OS=Homo sapiens]	1.031	0.999999886
General transcription factor IIH subunit 5 [OS=Homo sapiens]	1.031	0.999990059
Polyadenylate-binding protein 1 [OS=Homo sapiens]	1.032	0.766031
Polymerase delta-interacting protein 2 [OS=Homo sapiens]	1.032	0.887268088
Rho guanine nucleotide exchange factor 2 [OS=Homo sapiens]	1.032	0.784732841
40S ribosomal protein S9 [OS=Homo sapiens]	1.032	0.826579155
Eukaryotic translation initiation factor 1A, X-chromosomal [OS=Homo sapiens]	1.032	0.862663678
Translation initiation factor IF-2, mitochondrial [OS=Homo sapiens]	1.032	0.935852454
Rho GDP-dissociation inhibitor 1 [OS=Homo sapiens]	1.032	0.848450738
Vesicle-associated membrane protein 3 [OS=Homo sapiens]	1.032	0.999999992
Adenosine 5'-monophosphoramidase HINT2 [OS=Homo sapiens]	1.032	0.999999744
Vacuolar protein sorting-associated protein 29 [OS=Homo sapiens]	1.032	0.986650815
Huntingtin-interacting protein K [OS=Homo sapiens]	1.032	0.838506061
Eukaryotic translation initiation factor 4H [OS=Homo sapiens]	1.032	0.98862228
Poly(ADP-ribose) glycohydrolase [OS=Homo sapiens]	1.032	0.968424158
Golgi-resident adenosine 3',5'-bisphosphate 3'-phosphatase [OS=Homo sapiens	1.032	0.977099394
Pyruvate kinase PKLR [OS=Homo sapiens]	1.032	0.999525838
Transmembrane 9 superfamily member 1 [OS=Homo sapiens]	1.032	0.999965955
Phenylalanine--tRNA ligase, mitochondrial [OS=Homo sapiens]	1.032	0.874176522
Tsukushi [OS=Homo sapiens]	1.032	0.923313903
Centrosomal protein of 120 kDa [OS=Homo sapiens]	1.032	0.86341187
Glutamine--tRNA ligase [OS=Homo sapiens]	1.033	0.853718374
Calnexin [OS=Homo sapiens]	1.033	0.992863492
Cap-specific mRNA (nucleoside-2'-O-)-methyltransferase 1 [OS=Homo sapiens]	1.033	0.947741149
E3 ubiquitin-protein ligase HERC2 [OS=Homo sapiens]	1.033	0.522766788
Protein transport protein Sec23B [OS=Homo sapiens]	1.033	0.92180768
Dihydrolipoyllysine-residue succinyltransferase component of 2-oxoglutarate de	1.033	0.93915265
Actin-related protein 2/3 complex subunit 4 [OS=Homo sapiens]	1.033	0.797690484
UV excision repair protein RAD23 homolog A [OS=Homo sapiens]	1.033	0.871085261
C-terminal-binding protein 1 [OS=Homo sapiens]	1.033	0.999997189
Mediator of RNA polymerase II transcription subunit 14 [OS=Homo sapiens]	1.033	0.968633326
Pseudouridylate synthase RPUSD4, mitochondrial [OS=Homo sapiens]	1.033	0.987962154
UDP-glucuronic acid decarboxylase 1 [OS=Homo sapiens]	1.033	0.999727695
FAST kinase domain-containing protein 2, mitochondrial [OS=Homo sapiens]	1.033	0.977199732
Spastin [OS=Homo sapiens]	1.033	0.82191054
DNA topoisomerase 2-binding protein 1 [OS=Homo sapiens]	1.033	0.999839346
Protein O-mannose kinase [OS=Homo sapiens]	1.033	0.999348186
Signal peptidase complex catalytic subunit SEC11A [OS=Homo sapiens]	1.033	0.99999966
2-aminoethanethiol dioxygenase [OS=Homo sapiens]	1.033	0.992625224
ATP synthase mitochondrial F1 complex assembly factor 2 [OS=Homo sapiens]	1.033	0.788016049
Golgi SNAP receptor complex member 2 [OS=Homo sapiens]	1.033	0.999986967
Cell death regulator Aven [OS=Homo sapiens]	1.033	0.395633782
Ubiquinol-cytochrome-c reductase complex assembly factor 2 [OS=Homo sapie	1.033	1
Choline transporter-like protein 1 [OS=Homo sapiens]	1.033	0.999999455
Arginine-glutamic acid dipeptide repeats protein [OS=Homo sapiens]	1.033	0.956029558
Cyclin-dependent kinase 4 inhibitor B [OS=Homo sapiens]	1.033	0.999996005

Leucine-rich PPR motif-containing protein, mitochondrial [OS=Homo sapiens]	1.034	0.891547356
Atlastin-3 [OS=Homo sapiens]	1.034	0.836641614
ELKS/Rab6-interacting/CAST family member 1 [OS=Homo sapiens]	1.034	0.852897243
CTP synthase 1 [OS=Homo sapiens]	1.034	0.998110842
Thymidylate kinase [OS=Homo sapiens]	1.034	0.997752951
COP9 signalosome complex subunit 1 [OS=Homo sapiens]	1.034	0.835523508
Protein pelota homolog [OS=Homo sapiens]	1.034	0.862856158
Gamma-glutamyl hydrolase [OS=Homo sapiens]	1.034	0.697093833
2-oxoadipate dehydrogenase complex component E1 [OS=Homo sapiens]	1.034	0.825396523
ATP synthase subunit epsilon, mitochondrial [OS=Homo sapiens]	1.034	0.999912397
Intraflagellar transport protein 140 homolog [OS=Homo sapiens]	1.034	0.771162708
Rho-related GTP-binding protein RhoF [OS=Homo sapiens]	1.034	0.999986148
Transcription factor HIVEP2 [OS=Homo sapiens]	1.034	0.983338503
RNA cytosine C(5)-methyltransferase NSUN2 [OS=Homo sapiens]	1.035	0.998033246
Histidine--tRNA ligase, cytoplasmic [OS=Homo sapiens]	1.035	0.802619276
Cysteine and histidine-rich domain-containing protein 1 [OS=Homo sapiens]	1.035	0.999799353
Programmed cell death protein 5 [OS=Homo sapiens]	1.035	0.951079961
5'-nucleotidase domain-containing protein 1 [OS=Homo sapiens]	1.035	0.940788541
Ras-related protein Rab-5C [OS=Homo sapiens]	1.035	0.45130366
40S ribosomal protein S20 [OS=Homo sapiens]	1.035	0.965551485
Bifunctional coenzyme A synthase [OS=Homo sapiens]	1.035	0.80125184
Septin-8 [OS=Homo sapiens]	1.035	0.999990604
[Pyruvate dehydrogenase (acetyl-transferring)] kinase isozyme 3, mitochondrial	1.035	0.977203447
Exportin-4 [OS=Homo sapiens]	1.035	0.929080591
Zinc finger protein 143 [OS=Homo sapiens]	1.035	0.999953103
Suppressor of tumorigenicity 7 protein [OS=Homo sapiens]	1.035	0.999997807
26S proteasome non-ATPase regulatory subunit 1 [OS=Homo sapiens]	1.036	0.492718621
40S ribosomal protein S2 [OS=Homo sapiens]	1.036	0.991440484
Actin-related protein 2/3 complex subunit 2 [OS=Homo sapiens]	1.036	0.940561856
60S ribosomal protein L24 [OS=Homo sapiens]	1.036	0.988171874
La-related protein 4B [OS=Homo sapiens]	1.036	0.522529052
Signal recognition particle 14 kDa protein [OS=Homo sapiens]	1.036	0.499812719
Potassium-transporting ATPase alpha chain 1 [OS=Homo sapiens]	1.036	0.998905435
Queuine tRNA-ribosyltransferase catalytic subunit 1 [OS=Homo sapiens]	1.036	0.900596932
COMM domain-containing protein 4 [OS=Homo sapiens]	1.036	0.817484384
Protein TMED8 [OS=Homo sapiens]	1.036	1
28S ribosomal protein S2, mitochondrial [OS=Homo sapiens]	1.036	0.997614705
Sigma intracellular receptor 2 [OS=Homo sapiens]	1.036	0.968484426
Nuclear receptor 2C2-associated protein [OS=Homo sapiens]	1.036	0.97855654
U6 snRNA-associated Sm-like protein LSm4 [OS=Homo sapiens]	1.036	0.999809419
Very large A-kinase anchor protein [OS=Homo sapiens]	1.036	0.283678223
Pleckstrin homology domain-containing family A member 1 [OS=Homo sapiens]	1.036	0.999992572
TSC22 domain family protein 2 [OS=Homo sapiens]	1.036	0.996705819
Intraflagellar transport protein 122 homolog [OS=Homo sapiens]	1.036	0.999999045
Cilia- and flagella-associated protein 100 [OS=Homo sapiens]	1.036	0.991117625
E3 ubiquitin-protein ligase RNF181 [OS=Homo sapiens]	1.036	0.79115435
Sister chromatid cohesion protein DCC1 [OS=Homo sapiens]	1.036	0.954198787
Lysine--tRNA ligase [OS=Homo sapiens]	1.037	0.51578877
Serine/threonine-protein phosphatase PP1-alpha catalytic subunit [OS=Homo s	1.037	0.999999996
Far upstream element-binding protein 3 [OS=Homo sapiens]	1.037	0.148023851
Fatty acid-binding protein 5 [OS=Homo sapiens]	1.037	0.999359761
Protein SET [OS=Homo sapiens]	1.037	0.898268906
Dynein axonemal assembly factor 5 [OS=Homo sapiens]	1.037	0.644145434
Hypoxanthine-guanine phosphoribosyltransferase [OS=Homo sapiens]	1.037	0.957590405
39S ribosomal protein L22, mitochondrial [OS=Homo sapiens]	1.037	0.980732377
39S ribosomal protein L12, mitochondrial [OS=Homo sapiens]	1.037	0.999996782
Exportin-6 [OS=Homo sapiens]	1.037	0.831590594
Protein C18orf25 [OS=Homo sapiens]	1.037	0.999365821
Unconventional myosin-Ie [OS=Homo sapiens]	1.037	0.42958279
Fragile X messenger ribonucleoprotein 1 [OS=Homo sapiens]	1.037	0.978489242
Leydig cell tumor 10 kDa protein homolog [OS=Homo sapiens]	1.037	0.998225553
TELO2-interacting protein 1 homolog [OS=Homo sapiens]	1.037	0.997789086
Protein unc-119 homolog A [OS=Homo sapiens]	1.037	0.913564461
Trimethylguanosine synthase [OS=Homo sapiens]	1.037	0.997266227
ADP-ribose glycohydrolase OARD1 [OS=Homo sapiens]	1.037	0.83646377
Kelch-like protein 26 [OS=Homo sapiens]	1.037	0.951958122
SWI/SNF complex subunit SMARCC1 [OS=Homo sapiens]	1.038	0.760198844
AP-3 complex subunit delta-1 [OS=Homo sapiens]	1.038	0.568537273
26S proteasome non-ATPase regulatory subunit 11 [OS=Homo sapiens]	1.038	0.396382512
Eukaryotic translation initiation factor 3 subunit I [OS=Homo sapiens]	1.038	0.971467977
40S ribosomal protein S5 [OS=Homo sapiens]	1.038	0.785953784
Bifunctional 3'-phosphoadenosine 5'-phosphosulfate synthase 1 [OS=Homo sap	1.038	0.815381929
Probable ATP-dependent RNA helicase DDX28 [OS=Homo sapiens]	1.038	0.959015081
Nuclear pore complex protein Nup50 [OS=Homo sapiens]	1.038	1
BLOC-2 complex member HPS5 [OS=Homo sapiens]	1.038	0.74094248

Tubulin-specific chaperone cofactor E-like protein [OS=Homo sapiens]	1.038	0.999999951
Inositol polyphosphate-4-phosphatase type I A [OS=Homo sapiens]	1.038	0.526414801
Myoferlin [OS=Homo sapiens]	1.039	0.981101393
Acyl-coenzyme A thioesterase 9, mitochondrial [OS=Homo sapiens]	1.039	0.914069961
Serine/threonine-protein phosphatase 5 [OS=Homo sapiens]	1.039	0.758286602
Oxysterol-binding protein-related protein 8 [OS=Homo sapiens]	1.039	0.999835218
Pyrroline-5-carboxylate reductase 1, mitochondrial [OS=Homo sapiens]	1.039	0.786727134
DnaJ homolog subfamily C member 9 [OS=Homo sapiens]	1.039	0.8075541
Probable dimethyladenosine transferase [OS=Homo sapiens]	1.039	0.860290231
60S ribosomal protein L21 [OS=Homo sapiens]	1.039	0.990991931
Beta-arrestin-2 [OS=Homo sapiens]	1.039	0.993401384
Secretory carrier-associated membrane protein 3 [OS=Homo sapiens]	1.039	0.93146002
General transcription factor IIF subunit 2 [OS=Homo sapiens]	1.039	0.786526665
Regulator of nonsense transcripts 2 [OS=Homo sapiens]	1.039	0.988881311
60S ribosomal protein L36a [OS=Homo sapiens]	1.039	0.967935803
Target of Myb1 membrane trafficking protein [OS=Homo sapiens]	1.039	0.995630797
Presenilin-1 [OS=Homo sapiens]	1.039	0.991775647
Translocating chain-associated membrane protein 1 [OS=Homo sapiens]	1.039	0.998945416
DnaJ homolog subfamily C member 7 [OS=Homo sapiens]	1.04	0.948892005
60S ribosomal protein L7a [OS=Homo sapiens]	1.04	0.998843394
Ubiquitin-protein ligase E3A [OS=Homo sapiens]	1.04	0.67122044
SWI/SNF-related matrix-associated actin-dependent regulator of chromatin sub	1.04	0.277221348
ATP synthase subunit gamma, mitochondrial [OS=Homo sapiens]	1.04	0.999996665
Casein kinase II subunit beta [OS=Homo sapiens]	1.04	0.187241106
RNA-binding protein 33 [OS=Homo sapiens]	1.04	0.845216404
60S ribosomal protein L13a [OS=Homo sapiens]	1.04	0.898156069
Nucleoplasmin-3 [OS=Homo sapiens]	1.04	0.998155241
Protein furry homolog-like [OS=Homo sapiens]	1.04	0.994667893
60S ribosomal protein L27 [OS=Homo sapiens]	1.04	0.788523876
Ras-related protein Rab-34, isoform NARR [OS=Homo sapiens]	1.04	0.988735747
Huntingtin-interacting protein 1 [OS=Homo sapiens]	1.04	0.993976736
Probable ATP-dependent RNA helicase DHX35 [OS=Homo sapiens]	1.04	0.999977136
Cyclin-dependent kinase 2-associated protein 1 [OS=Homo sapiens]	1.04	0.982806924
Adenylyltransferase and sulfurtransferase MOCS3 [OS=Homo sapiens]	1.04	0.90873061
E3 ubiquitin-protein ligase UHRF2 [OS=Homo sapiens]	1.04	0.98328429
Plasma membrane ascorbate-dependent reductase CYBRD1 [OS=Homo sapien	1.04	0.98466097
Thioredoxin domain-containing protein 16 [OS=Homo sapiens]	1.04	0.999745939
Protein mono-ADP-ribosyltransferase PARP12 [OS=Homo sapiens]	1.04	0.999900377
Pleckstrin homology-like domain family A member 2 [OS=Homo sapiens]	1.04	0.761869791
Adenylyl cyclase-associated protein 2 [OS=Homo sapiens]	1.041	0.803282725
Actin-related protein 2/3 complex subunit 5 [OS=Homo sapiens]	1.041	0.336268229
Developmentally-regulated GTP-binding protein 2 [OS=Homo sapiens]	1.041	0.989128992
Conserved oligomeric Golgi complex subunit 7 [OS=Homo sapiens]	1.041	0.757856566
39S ribosomal protein L13, mitochondrial [OS=Homo sapiens]	1.041	0.99552729
Zinc finger protein 830 [OS=Homo sapiens]	1.041	0.999765353
Lysine-specific demethylase 3A [OS=Homo sapiens]	1.041	0.853811348
Leucine-rich repeat-containing protein 1 [OS=Homo sapiens]	1.041	0.999363672
Histone-lysine N-methyltransferase SMYD3 [OS=Homo sapiens]	1.041	0.997552377
DNA excision repair protein ERCC-1 [OS=Homo sapiens]	1.041	0.898634375
BRISC and BRCA1-A complex member 2 [OS=Homo sapiens]	1.041	0.980617608
MRG/MORF4L-binding protein [OS=Homo sapiens]	1.041	0.952536371
Transcription factor SPT20 homolog [OS=Homo sapiens]	1.041	0.999999997
AN1-type zinc finger protein 2B [OS=Homo sapiens]	1.041	0.999961589
Transgelin-2 [OS=Homo sapiens]	1.042	0.901727771
Serine hydroxymethyltransferase, mitochondrial [OS=Homo sapiens]	1.042	0.969770836
Prostaglandin reductase 1 [OS=Homo sapiens]	1.042	0.937484237
Cingulin [OS=Homo sapiens]	1.042	0.8599225
40S ribosomal protein S15 [OS=Homo sapiens]	1.042	0.997918339
Proteasome subunit alpha type-5 [OS=Homo sapiens]	1.042	0.991616508
Negative elongation factor E [OS=Homo sapiens]	1.042	0.904832991
Long-chain fatty acid transport protein 4 [OS=Homo sapiens]	1.042	0.998819444
PRKC apoptosis WT1 regulator protein [OS=Homo sapiens]	1.042	0.951494399
60S ribosomal protein L36 [OS=Homo sapiens]	1.042	0.497042143
NF-kappa-B essential modulator [OS=Homo sapiens]	1.042	0.954324291
Multifunctional methyltransferase subunit TRM112-like protein [OS=Homo sapie	1.042	0.999725164
ADP-ribosylation factor-like protein 2 [OS=Homo sapiens]	1.042	0.476399554
Zinc finger CCCH-type with G patch domain-containing protein [OS=Homo sapie	1.042	0.999980436
Rho GTPase-activating protein 5 [OS=Homo sapiens]	1.042	0.989451784
Squalene monooxygenase [OS=Homo sapiens]	1.042	0.85748326
Arfaptin-2 [OS=Homo sapiens]	1.042	0.983990241
Tropomodulin-2 [OS=Homo sapiens]	1.042	0.743724984
Nucleoside diphosphate kinase 6 [OS=Homo sapiens]	1.042	0.936118533
Tetratricopeptide repeat protein 14 [OS=Homo sapiens]	1.042	0.945954638
Elongation factor 1-alpha 1 [OS=Homo sapiens]	1.043	0.772722245
Eukaryotic translation initiation factor 3 subunit B [OS=Homo sapiens]	1.043	0.997597654

Thymidine phosphorylase [OS=Homo sapiens]	1.043	0.213573876
Ferrochelatase, mitochondrial [OS=Homo sapiens]	1.043	0.791571841
Transforming protein RhoA [OS=Homo sapiens]	1.043	0.991178485
Myocardin-related transcription factor B [OS=Homo sapiens]	1.043	0.714879819
Inositol-3-phosphate synthase 1 [OS=Homo sapiens]	1.043	0.58512465
DNA polymerase epsilon subunit 3 [OS=Homo sapiens]	1.043	0.99999103
V-type proton ATPase subunit G 1 [OS=Homo sapiens]	1.043	0.999997187
Protein FAM98C [OS=Homo sapiens]	1.043	0.998054458
Cytochrome c oxidase assembly protein COX11, mitochondrial [OS=Homo sapie	1.043	0.992815743
Ubiquitin carboxyl-terminal hydrolase BAP1 [OS=Homo sapiens]	1.043	0.339383461
FAST kinase domain-containing protein 3, mitochondrial [OS=Homo sapiens]	1.043	0.897303897
HUWE1-associated protein modifying stress responses [OS=Homo sapiens]	1.043	0.999812959
Methionine--tRNA ligase, cytoplasmic [OS=Homo sapiens]	1.044	0.906317556
Dolichyl-diphosphooligosaccharide--protein glycosyltransferase subunit 1 [OS=	1.044	0.999897754
Alpha-aminoadipic semialdehyde dehydrogenase [OS=Homo sapiens]	1.044	0.689955782
RUN and FYVE domain-containing protein 1 [OS=Homo sapiens]	1.044	0.415165239
Lactoylglutathione lyase [OS=Homo sapiens]	1.044	0.999964228
Vacuolar protein sorting-associated protein 51 homolog [OS=Homo sapiens]	1.044	0.534432374
NIF3-like protein 1 [OS=Homo sapiens]	1.044	0.924425792
Translation initiation factor eIF-2B subunit gamma [OS=Homo sapiens]	1.044	0.548190864
Multidrug resistance-associated protein 1 [OS=Homo sapiens]	1.044	0.999960699
Transcription termination factor 3, mitochondrial [OS=Homo sapiens]	1.044	0.985575622
WAS protein family homolog 6 [OS=Homo sapiens]	1.044	0.967913986
Ribosomal oxygenase 1 [OS=Homo sapiens]	1.044	0.999994513
Ubiquitin-conjugating enzyme E2 G2 [OS=Homo sapiens]	1.044	0.912831277
Beta-adrenergic receptor kinase 1 [OS=Homo sapiens]	1.044	0.688130391
1-acyl-sn-glycerol-3-phosphate acyltransferase gamma [OS=Homo sapiens]	1.044	0.563418885
Putative coiled-coil-helix-coiled-coil-helix domain-containing protein CHCHD2F	1.044	0.411675495
Tyrosine-protein kinase transmembrane receptor ROR2 [OS=Homo sapiens]	1.044	0.973093631
DNA polymerase beta [OS=Homo sapiens]	1.044	0.999320254
CAP-Gly domain-containing linker protein 1 [OS=Homo sapiens]	1.045	0.821516382
26S proteasome regulatory subunit 4 [OS=Homo sapiens]	1.045	0.639777886
Plastin-3 [OS=Homo sapiens]	1.045	0.806641451
26S proteasome regulatory subunit 8 [OS=Homo sapiens]	1.045	0.610619062
Ubiquitin-like modifier-activating enzyme 6 [OS=Homo sapiens]	1.045	0.807715798
2',3'-cyclic-nucleotide 3'-phosphodiesterase [OS=Homo sapiens]	1.045	0.956088344
Ubiquitin-conjugating enzyme E2 C [OS=Homo sapiens]	1.045	0.882153849
Methylthioribose-1-phosphate isomerase [OS=Homo sapiens]	1.045	0.75333978
Histone-lysine N-trimethyltransferase SMYD5 [OS=Homo sapiens]	1.045	0.995223716
Disabled homolog 2-interacting protein [OS=Homo sapiens]	1.045	0.991938463
Mediator of RNA polymerase II transcription subunit 25 [OS=Homo sapiens]	1.045	0.953710252
Leucine-rich repeat-containing protein 14 [OS=Homo sapiens]	1.045	0.622562157
D-aminoacyl-tRNA deacylase 1 [OS=Homo sapiens]	1.045	0.946329819
ADP-ribose pyrophosphatase, mitochondrial [OS=Homo sapiens]	1.045	0.997583471
Protein-tyrosine sulfotransferase 1 [OS=Homo sapiens]	1.045	0.999954263
14-3-3 protein epsilon [OS=Homo sapiens]	1.046	0.390757807
N-myc-interactor [OS=Homo sapiens]	1.046	0.589073536
60S ribosomal protein L32 [OS=Homo sapiens]	1.046	0.988366062
60S acidic ribosomal protein P0 [OS=Homo sapiens]	1.046	0.54258619
Mitotic checkpoint serine/threonine-protein kinase BUB1 beta [OS=Homo sapier	1.046	0.997900729
Protein ABHD11 [OS=Homo sapiens]	1.046	0.998600036
E3 ubiquitin-protein ligase makorin-2 [OS=Homo sapiens]	1.046	0.267935734
tRNA N6-adenosine threonylcarbamoyltransferase [OS=Homo sapiens]	1.046	0.503714705
Glucocorticoid modulatory element-binding protein 1 [OS=Homo sapiens]	1.046	0.985254705
Exocyst complex component 6B [OS=Homo sapiens]	1.046	0.514760333
Stearoyl-CoA desaturase [OS=Homo sapiens]	1.046	1
Growth hormone-inducible transmembrane protein [OS=Homo sapiens]	1.046	0.99991331
Calretinin [OS=Homo sapiens]	1.046	0.804866878
Protein monoglycylase TTLL8 [OS=Homo sapiens]	1.046	0.836384242
Ribonucleoside-diphosphate reductase large subunit [OS=Homo sapiens]	1.047	0.757378757
Proteasome activator complex subunit 2 [OS=Homo sapiens]	1.047	0.56920356
DNA ligase 3 [OS=Homo sapiens]	1.047	0.90974806
Chromodomain-helicase-DNA-binding protein 3 [OS=Homo sapiens]	1.047	0.999998848
Disco-interacting protein 2 homolog B [OS=Homo sapiens]	1.047	0.193983396
Cell division control protein 42 homolog [OS=Homo sapiens]	1.047	0.845350764
Calcineurin B homologous protein 1 [OS=Homo sapiens]	1.047	0.730442268
Spermatogenesis-associated serine-rich protein 2 [OS=Homo sapiens]	1.047	0.648694139
39S ribosomal protein L49, mitochondrial [OS=Homo sapiens]	1.047	0.586792112
MTOR-associated protein MEAK7 [OS=Homo sapiens]	1.047	0.944672344
Alanyl-tRNA editing protein Aarsd1 [OS=Homo sapiens]	1.047	0.744687081
Integrator complex subunit 11 [OS=Homo sapiens]	1.047	0.799934418
Phosphorylase b kinase regulatory subunit beta [OS=Homo sapiens]	1.047	0.849991491
Propionyl-CoA carboxylase beta chain, mitochondrial [OS=Homo sapiens]	1.047	0.988910506
Pre-mRNA-processing factor 39 [OS=Homo sapiens]	1.047	0.998487895
40S ribosomal protein S3 [OS=Homo sapiens]	1.048	0.907905431

Pumilio homolog 1 [OS=Homo sapiens]	1.048	0.51610049
Elongator complex protein 1 [OS=Homo sapiens]	1.048	0.918275377
DAZ-associated protein 1 [OS=Homo sapiens]	1.048	0.999707405
Actin-related protein 2/3 complex subunit 1B [OS=Homo sapiens]	1.048	0.291604209
Wolframin [OS=Homo sapiens]	1.048	0.999999661
SUMO-conjugating enzyme UBC9 [OS=Homo sapiens]	1.048	0.85288615
Oxysterol-binding protein-related protein 11 [OS=Homo sapiens]	1.048	0.992892391
PDZ and LIM domain protein 5 [OS=Homo sapiens]	1.048	0.479253804
[F-actin]-monooxygenase MICAL3 [OS=Homo sapiens]	1.048	0.494808969
HMG box transcription factor BBX [OS=Homo sapiens]	1.048	0.998903602
Serine/threonine-protein kinase 25 [OS=Homo sapiens]	1.048	0.789124076
Transmembrane protein 230 [OS=Homo sapiens]	1.048	0.898846428
Protein cornichon homolog 4 [OS=Homo sapiens]	1.048	0.999999399
PDZ and LIM domain protein 2 [OS=Homo sapiens]	1.048	0.875106889
BolA-like protein 3 [OS=Homo sapiens]	1.048	0.917964538
Cytosolic carboxypeptidase 1 [OS=Homo sapiens]	1.048	0.701073222
26S proteasome non-ATPase regulatory subunit 12 [OS=Homo sapiens]	1.049	0.457268009
Elongation factor 1-gamma [OS=Homo sapiens]	1.049	0.818840435
Putative nucleoside diphosphate kinase [OS=Homo sapiens]	1.049	0.999999985
Protein PAT1 homolog 1 [OS=Homo sapiens]	1.049	0.1955682
Pyridoxal-dependent decarboxylase domain-containing protein 1 [OS=Homo sa	1.049	0.790617938
Dynein axonemal assembly factor 10 [OS=Homo sapiens]	1.049	0.669619535
ADP-ribosylation factor-like protein 6 [OS=Homo sapiens]	1.049	0.624370213
Peflin [OS=Homo sapiens]	1.049	0.545566525
WD repeat-containing protein 35 [OS=Homo sapiens]	1.049	0.999999999
Galactose mutarotase [OS=Homo sapiens]	1.049	1
Protein AMN1 homolog [OS=Homo sapiens]	1.049	0.998838815
Pro-cathepsin H [OS=Homo sapiens]	1.049	0.966549386
60S ribosomal protein L5 [OS=Homo sapiens]	1.05	0.444091156
WASH complex subunit 2C [OS=Homo sapiens]	1.05	0.97187996
E3 ubiquitin-protein ligase BRE1A [OS=Homo sapiens]	1.05	0.341630322
BRCA2 and CDKN1A-interacting protein [OS=Homo sapiens]	1.05	0.58703752
Rab3 GTPase-activating protein non-catalytic subunit [OS=Homo sapiens]	1.05	0.647449658
Mitofusin-2 [OS=Homo sapiens]	1.05	0.85646258
E3 ubiquitin-protein ligase ARIH2 [OS=Homo sapiens]	1.05	0.656280513
AP-2 complex subunit mu [OS=Homo sapiens]	1.05	0.620570656
Cysteine and glycine-rich protein 2 [OS=Homo sapiens]	1.05	0.999499014
F-actin-uncapping protein LRRC16A [OS=Homo sapiens]	1.05	0.719929015
Autophagy-related protein 9A [OS=Homo sapiens]	1.05	0.640650668
Delta-aminolevulinic acid dehydratase [OS=Homo sapiens]	1.05	0.546010816
TBC1 domain family member 23 [OS=Homo sapiens]	1.05	0.93197295
Protein FAM210B, mitochondrial [OS=Homo sapiens]	1.05	0.999839399
Cyclic AMP-responsive element-binding protein 1 [OS=Homo sapiens]	1.05	0.988834248
Transmembrane protein 14C [OS=Homo sapiens]	1.05	0.999991219
Lysophosphatidylcholine acyltransferase 2 [OS=Homo sapiens]	1.05	0.564091508
CMP-N-acetylneuraminate-beta-galactosamide-alpha-2,3-sialyltransferase 2 [C	1.05	0.994007354
Phosphatidylinositol-glycan biosynthesis class W protein [OS=Homo sapiens]	1.05	0.994562557
Eukaryotic initiation factor 4A-II [OS=Homo sapiens]	1.051	0.998603393
Cullin-1 [OS=Homo sapiens]	1.051	0.535284432
B-cell receptor-associated protein 31 [OS=Homo sapiens]	1.051	0.722552756
Kinesin-like protein KIF3B [OS=Homo sapiens]	1.051	0.460628414
Nucleobindin-2 [OS=Homo sapiens]	1.051	0.505301663
40S ribosomal protein S8 [OS=Homo sapiens]	1.051	0.592572367
Focadhesin [OS=Homo sapiens]	1.051	0.866545994
Tapasin [OS=Homo sapiens]	1.051	0.856534956
Protein ELFN1 [OS=Homo sapiens]	1.051	0.993465257
Syntaxin-8 [OS=Homo sapiens]	1.051	0.963325816
CDP-diacylglycerol--inositol 3-phosphatidyltransferase [OS=Homo sapiens]	1.051	0.843807304
Nucleoside diphosphate kinase 7 [OS=Homo sapiens]	1.051	0.999999901
Vacuolar protein sorting-associated protein 26C [OS=Homo sapiens]	1.051	0.99999803
Protein CC2D2B [OS=Homo sapiens]	1.051	0.889733103
Spectrin alpha chain, erythrocytic 1 [OS=Homo sapiens]	1.051	0.999999986
Trifunctional purine biosynthetic protein adenosine-3 [OS=Homo sapiens]	1.052	0.9919705
Ran GTPase-activating protein 1 [OS=Homo sapiens]	1.052	0.999930573
Phospholipase A-2-activating protein [OS=Homo sapiens]	1.052	0.478698793
tRNA methyltransferase 10 homolog C [OS=Homo sapiens]	1.052	0.603846478
General transcription factor IIF subunit 1 [OS=Homo sapiens]	1.052	0.767543138
2',5'-phosphodiesterase 12 [OS=Homo sapiens]	1.052	0.893811609
Serine/threonine-protein kinase TAO1 [OS=Homo sapiens]	1.052	0.297611133
SRA stem-loop-interacting RNA-binding protein, mitochondrial [OS=Homo sapie	1.052	0.852266498
RNA-binding protein 27 [OS=Homo sapiens]	1.052	0.470283921
Elongator complex protein 3 [OS=Homo sapiens]	1.052	0.993334617
Vacuolar protein sorting-associated protein 37B [OS=Homo sapiens]	1.052	0.5156982
39S ribosomal protein L9, mitochondrial [OS=Homo sapiens]	1.052	0.999999952
Myotrophin [OS=Homo sapiens]	1.052	0.814212063

Metal transporter CNNM3 [OS=Homo sapiens]	1.052	0.999260047
Putative monooxygenase p33MONOX [OS=Homo sapiens]	1.052	0.435716648
Protein XRP2 [OS=Homo sapiens]	1.052	0.994942714
WD repeat-containing protein 7 [OS=Homo sapiens]	1.052	0.50142856
Serine/threonine-protein kinase tousled-like 2 [OS=Homo sapiens]	1.052	0.992645283
Acetyl-coenzyme A transporter 1 [OS=Homo sapiens]	1.052	0.999999479
Delta(14)-sterol reductase LBR [OS=Homo sapiens]	1.052	0.999853632
Ankyrin repeat domain-containing protein 54 [OS=Homo sapiens]	1.052	0.996721258
Phosphatase and actin regulator 2 [OS=Homo sapiens]	1.052	0.710386748
Ubiquitin-associated domain-containing protein 1 [OS=Homo sapiens]	1.052	0.446688092
Integral membrane protein GPR180 [OS=Homo sapiens]	1.052	0.973165123
Histone deacetylase 8 [OS=Homo sapiens]	1.052	0.667686896
ATP-dependent 6-phosphofructokinase, platelet type [OS=Homo sapiens]	1.053	0.885773297
Eukaryotic translation initiation factor 3 subunit D [OS=Homo sapiens]	1.053	0.984722378
26S proteasome non-ATPase regulatory subunit 4 [OS=Homo sapiens]	1.053	0.870291508
28S ribosomal protein S31, mitochondrial [OS=Homo sapiens]	1.053	0.976273354
Splicing factor U2AF 35 kDa subunit [OS=Homo sapiens]	1.053	0.999906686
Dual specificity mitogen-activated protein kinase kinase 2 [OS=Homo sapiens]	1.053	0.113813042
DCN1-like protein 1 [OS=Homo sapiens]	1.053	0.914013479
Telomerase RNA component interacting RNase [OS=Homo sapiens]	1.053	0.989179806
Cytoplasmic protein NCK1 [OS=Homo sapiens]	1.053	0.618267085
DnaJ homolog subfamily C member 17 [OS=Homo sapiens]	1.053	0.976973095
COP9 signalosome complex subunit 8 [OS=Homo sapiens]	1.053	0.683683628
Sperm acrosome developmental regulator [OS=Homo sapiens]	1.053	0.238489698
Serine/threonine-protein kinase RIO3 [OS=Homo sapiens]	1.053	0.851854227
Transmembrane protein 131 [OS=Homo sapiens]	1.053	0.998848379
Serine/threonine-protein kinase ULK1 [OS=Homo sapiens]	1.053	0.997312452
Rho GTPase-activating protein 18 [OS=Homo sapiens]	1.053	0.970957848
Isoleucine--tRNA ligase, cytoplasmic [OS=Homo sapiens]	1.054	0.486563411
Peptidyl-prolyl cis-trans isomerase A [OS=Homo sapiens]	1.054	0.220962405
Ankyrin repeat and KH domain-containing protein 1 [OS=Homo sapiens]	1.054	0.745549699
ATP-dependent RNA helicase DDX42 [OS=Homo sapiens]	1.054	0.535441713
28S ribosomal protein S35, mitochondrial [OS=Homo sapiens]	1.054	0.971904087
Transcription initiation factor IIB [OS=Homo sapiens]	1.054	0.997049712
REST corepressor 3 [OS=Homo sapiens]	1.054	0.996834953
WW domain-binding protein 4 [OS=Homo sapiens]	1.054	0.969490957
Cathepsin Z [OS=Homo sapiens]	1.054	0.761013326
Rab proteins geranylgeranyltransferase component A 1 [OS=Homo sapiens]	1.054	0.927652153
DNA-directed RNA polymerase III subunit RPC8 [OS=Homo sapiens]	1.054	0.976990789
Polypeptide N-acetylgalactosaminyltransferase 11 [OS=Homo sapiens]	1.054	0.99977305
Biogenesis of lysosome-related organelles complex 1 subunit 5 [OS=Homo sapi	1.054	0.373678465
Dysbindin domain-containing protein 1 [OS=Homo sapiens]	1.054	0.977651915
Protein PRRC2A [OS=Homo sapiens]	1.055	0.375337142
Enoyl-CoA hydratase, mitochondrial [OS=Homo sapiens]	1.055	0.563046258
Sulfide:quinone oxidoreductase, mitochondrial [OS=Homo sapiens]	1.055	0.897775921
Tropomyosin alpha-3 chain [OS=Homo sapiens]	1.055	0.698858759
A-kinase anchor protein 9 [OS=Homo sapiens]	1.055	0.966274897
40S ribosomal protein S12 [OS=Homo sapiens]	1.055	0.600379492
Endoribonuclease LACTB2 [OS=Homo sapiens]	1.055	0.310777442
39S ribosomal protein L44, mitochondrial [OS=Homo sapiens]	1.055	0.880658049
Opioid growth factor receptor [OS=Homo sapiens]	1.055	0.734263132
Phosphoinositide 3-kinase regulatory subunit 4 [OS=Homo sapiens]	1.055	0.935133382
Protein PAXX [OS=Homo sapiens]	1.055	0.203860048
Deoxyribonuclease TATDN1 [OS=Homo sapiens]	1.055	0.983617045
2-(3-amino-3-carboxypropyl)histidine synthase subunit 2 [OS=Homo sapiens]	1.055	0.387842707
Nonsense-mediated mRNA decay factor SMG8 [OS=Homo sapiens]	1.055	0.922779124
Glutamyl-tRNA(Gln) amidotransferase subunit C, mitochondrial [OS=Homo sap	1.055	0.543335049
Soluble calcium-activated nucleotidase 1 [OS=Homo sapiens]	1.055	0.983719096
Kinesin-like protein KIF1A [OS=Homo sapiens]	1.056	0.498808315
Perilipin-3 [OS=Homo sapiens]	1.056	0.953047879
AP-1 complex subunit beta-1 [OS=Homo sapiens]	1.056	0.337648987
Proliferation-associated protein 2G4 [OS=Homo sapiens]	1.056	0.870746401
Serine/threonine-protein kinase WNK1 [OS=Homo sapiens]	1.056	0.294643962
Developmentally-regulated GTP-binding protein 1 [OS=Homo sapiens]	1.056	0.641383882
Xaa-Pro aminopeptidase 1 [OS=Homo sapiens]	1.056	0.368977499
Exosome complex exonuclease RRP44 [OS=Homo sapiens]	1.056	0.52175515
Phosphomevalonate kinase [OS=Homo sapiens]	1.056	0.997845952
CCR4-NOT transcription complex subunit 3 [OS=Homo sapiens]	1.056	0.056484429
EKC/KEOPS complex subunit TP53RK [OS=Homo sapiens]	1.056	0.558521316
Lysine-specific demethylase RSBN1L [OS=Homo sapiens]	1.056	0.913309825
Phosphatidylglycerophosphatase and protein-tyrosine phosphatase 1 [OS=Hom	1.056	0.628158784
Mediator of RNA polymerase II transcription subunit 4 [OS=Homo sapiens]	1.056	0.98851422
CDGSH iron-sulfur domain-containing protein 3, mitochondrial [OS=Homo sapie	1.056	0.991108149
C-Maf-inducing protein [OS=Homo sapiens]	1.056	1
Receptor expression-enhancing protein 6 [OS=Homo sapiens]	1.056	0.550636055

Protein lin-9 homolog [OS=Homo sapiens]	1.056	1
Ribosomal protein S6 kinase beta-2 [OS=Homo sapiens]	1.056	0.999894232
Serine/threonine-protein phosphatase CPPED1 [OS=Homo sapiens]	1.057	0.855535118
Stromal interaction molecule 1 [OS=Homo sapiens]	1.057	0.965165681
Anaphase-promoting complex subunit CDC26 [OS=Homo sapiens]	1.057	0.559542237
Apoptosis-stimulating of p53 protein 2 [OS=Homo sapiens]	1.057	0.129065364
Epithelial discoidin domain-containing receptor 1 [OS=Homo sapiens]	1.057	0.315506903
H(+)/Cl(-) exchange transporter 7 [OS=Homo sapiens]	1.057	0.999999709
E3 ubiquitin-protein ligase RNF115 [OS=Homo sapiens]	1.057	0.999999957
Mitotic-spindle organizing protein 1 [OS=Homo sapiens]	1.057	0.998633812
DNA polymerase subunit gamma-2, mitochondrial [OS=Homo sapiens]	1.057	0.927685455
Microtubule-actin cross-linking factor 1, isoforms 1/2/3/4/5 [OS=Homo sapiens]	1.058	0.275525054
CAD protein [OS=Homo sapiens]	1.058	0.783397443
Stress-induced-phosphoprotein 1 [OS=Homo sapiens]	1.058	0.655764275
Phosphoglycerate kinase 1 [OS=Homo sapiens]	1.058	0.516717095
NAD-dependent malic enzyme, mitochondrial [OS=Homo sapiens]	1.058	0.755266289
Importin subunit alpha-7 [OS=Homo sapiens]	1.058	0.025496348
AP-1 complex subunit mu-1 [OS=Homo sapiens]	1.058	0.483985095
Coiled-coil domain-containing protein 124 [OS=Homo sapiens]	1.058	0.779587402
Transportin-3 [OS=Homo sapiens]	1.058	0.46653377
Leukocyte elastase inhibitor [OS=Homo sapiens]	1.058	0.714394434
Exocyst complex component 3 [OS=Homo sapiens]	1.058	0.265410052
40S ribosomal protein S27 [OS=Homo sapiens]	1.058	0.532558415
Thiosulfate sulfurtransferase [OS=Homo sapiens]	1.058	0.989357838
Centrosomal protein of 85 kDa [OS=Homo sapiens]	1.058	0.998542135
Conserved oligomeric Golgi complex subunit 5 [OS=Homo sapiens]	1.058	0.447560314
von Hippel-Lindau disease tumor suppressor [OS=Homo sapiens]	1.058	0.735088619
AP-4 complex subunit beta-1 [OS=Homo sapiens]	1.058	0.99993176
Stonin-2 [OS=Homo sapiens]	1.058	0.396474765
Battenin [OS=Homo sapiens]	1.058	0.999899907
Leucine-rich repeat-containing protein 36 [OS=Homo sapiens]	1.058	0.203981164
Endoplasmin [OS=Homo sapiens]	1.059	0.666701689
E3 ubiquitin/ISG15 ligase TRIM25 [OS=Homo sapiens]	1.059	0.452838174
Pentatricopeptide repeat domain-containing protein 3, mitochondrial [OS=Hom	1.059	0.858429407
Extended synaptotagmin-2 [OS=Homo sapiens]	1.059	0.495715728
A-kinase anchor protein 13 [OS=Homo sapiens]	1.059	0.340531578
DNA-directed RNA polymerase III subunit RPC1 [OS=Homo sapiens]	1.059	0.52098877
THUMP domain-containing protein 1 [OS=Homo sapiens]	1.059	0.724865914
Neurochondrin [OS=Homo sapiens]	1.059	0.648910082
Anaphase-promoting complex subunit 5 [OS=Homo sapiens]	1.059	0.969002757
Oxysterol-binding protein-related protein 10 [OS=Homo sapiens]	1.059	0.986777692
Synaptojanin-1 [OS=Homo sapiens]	1.059	0.478115471
Centrosomal protein of 170 kDa protein B [OS=Homo sapiens]	1.059	0.99999972
SH3 domain-binding protein 5 [OS=Homo sapiens]	1.059	0.999999961
E3 SUMO-protein ligase ZBED1 [OS=Homo sapiens]	1.059	0.945468059
Volume-regulated anion channel subunit LRRC8A [OS=Homo sapiens]	1.059	0.999073737
Probable JmjC domain-containing histone demethylation protein 2C [OS=Homo	1.059	0.999064133
3-phosphoinositide-dependent protein kinase 1 [OS=Homo sapiens]	1.059	0.999999995
Protein FAM107B [OS=Homo sapiens]	1.059	0.711207768
G protein-coupled receptor kinase 6 [OS=Homo sapiens]	1.059	0.455328397
Mediator of RNA polymerase II transcription subunit 8 [OS=Homo sapiens]	1.059	0.965106514
Pyruvate kinase PKM [OS=Homo sapiens]	1.06	0.865995158
Dynactin subunit 1 [OS=Homo sapiens]	1.06	0.299009793
Lupus La protein [OS=Homo sapiens]	1.06	0.631807562
ATP-binding cassette sub-family E member 1 [OS=Homo sapiens]	1.06	0.287859233
E3 ubiquitin-protein ligase BRE1B [OS=Homo sapiens]	1.06	0.327821167
COP9 signalosome complex subunit 3 [OS=Homo sapiens]	1.06	0.38505168
Conserved oligomeric Golgi complex subunit 4 [OS=Homo sapiens]	1.06	0.654466318
Antigen peptide transporter 1 [OS=Homo sapiens]	1.06	0.486842651
60S ribosomal protein L23a [OS=Homo sapiens]	1.06	0.800666674
Sex comb on midleg-like protein 2 [OS=Homo sapiens]	1.06	0.989290203
Ubiquitin-conjugating enzyme E2 E1 [OS=Homo sapiens]	1.06	0.689841365
Egl nine homolog 1 [OS=Homo sapiens]	1.06	0.669876902
Peptidylprolyl isomerase domain and WD repeat-containing protein 1 [OS=Hom	1.06	0.985349078
Myelin protein zero-like protein 1 [OS=Homo sapiens]	1.06	0.977023962
UPF0449 protein C19orf25 [OS=Homo sapiens]	1.06	0.909739521
COX assembly mitochondrial protein 2 homolog [OS=Homo sapiens]	1.06	0.959156449
Protein angel homolog 2 [OS=Homo sapiens]	1.06	0.321933267
Testis-expressed protein 30 [OS=Homo sapiens]	1.06	0.99769902
Inorganic pyrophosphatase [OS=Homo sapiens]	1.061	0.752203187
TBC1 domain family member 15 [OS=Homo sapiens]	1.061	0.64062278
NADH-cytochrome b5 reductase 3 [OS=Homo sapiens]	1.061	0.885170933
Oxygen-dependent coproporphyrinogen-III oxidase, mitochondrial [OS=Homo sa	1.061	0.595043068
YTH domain-containing family protein 2 [OS=Homo sapiens]	1.061	0.52333139
Enoyl-[acyl-carrier-protein] reductase, mitochondrial [OS=Homo sapiens]	1.061	0.572236357

DnaJ homolog subfamily C member 5 [OS=Homo sapiens]	1.061	0.292047028
Trafficking protein particle complex subunit 5 [OS=Homo sapiens]	1.061	0.829733423
Kinesin-associated protein 3 [OS=Homo sapiens]	1.061	0.959780264
Isoamyl acetate-hydrolyzing esterase 1 homolog [OS=Homo sapiens]	1.061	0.809809423
Protein CUSTOS [OS=Homo sapiens]	1.061	0.99999993
A-kinase anchor protein 10, mitochondrial [OS=Homo sapiens]	1.061	0.590799882
Lysophospholipid acyltransferase 7 [OS=Homo sapiens]	1.061	0.999990764
S1 RNA-binding domain-containing protein 1 [OS=Homo sapiens]	1.061	0.989924958
Ketohexokinase [OS=Homo sapiens]	1.061	0.961886096
Periplakin [OS=Homo sapiens]	1.062	0.789178407
Peroxiredoxin-6 [OS=Homo sapiens]	1.062	0.249721006
Plexin-B2 [OS=Homo sapiens]	1.062	0.968848711
Leucine-rich repeat-containing protein 59 [OS=Homo sapiens]	1.062	0.915183322
Proteasome subunit beta type-1 [OS=Homo sapiens]	1.062	0.925295081
Barrier-to-autointegration factor [OS=Homo sapiens]	1.062	0.995360758
Alpha-endosulfine [OS=Homo sapiens]	1.062	0.291777991
Rhophilin-2 [OS=Homo sapiens]	1.062	0.916151196
Ras-related protein Rab-5A [OS=Homo sapiens]	1.062	0.418146324
MHC class II regulatory factor RFX1 [OS=Homo sapiens]	1.062	0.976627608
Thioredoxin domain-containing protein 17 [OS=Homo sapiens]	1.062	0.556002355
EKC/KEOPS complex subunit TPRKB [OS=Homo sapiens]	1.062	0.983809043
DnaJ homolog subfamily C member 1 [OS=Homo sapiens]	1.062	0.993684022
Insulin receptor [OS=Homo sapiens]	1.062	0.973416825
Heat shock 70 kDa protein 1B [OS=Homo sapiens]	1.063	0.672367397
Fructose-bisphosphate aldolase A [OS=Homo sapiens]	1.063	0.933311476
Annexin A7 [OS=Homo sapiens]	1.063	0.490568372
Septin-7 [OS=Homo sapiens]	1.063	0.591040781
26S proteasome non-ATPase regulatory subunit 5 [OS=Homo sapiens]	1.063	0.680060808
Nicotinate phosphoribosyltransferase [OS=Homo sapiens]	1.063	0.164333763
Eukaryotic translation initiation factor 5A-1 [OS=Homo sapiens]	1.063	0.180529125
Interferon-inducible double-stranded RNA-dependent protein kinase activator A	1.063	0.795755063
Protein phosphatase 1 regulatory subunit 7 [OS=Homo sapiens]	1.063	0.466433567
Dual specificity mitogen-activated protein kinase kinase 1 [OS=Homo sapiens]	1.063	0.731619907
Coiled-coil domain-containing protein 22 [OS=Homo sapiens]	1.063	0.464521081
Calcium/calmodulin-dependent protein kinase type II subunit gamma [OS=Hom	1.063	0.296155952
Hydroxyacyl-coenzyme A dehydrogenase, mitochondrial [OS=Homo sapiens]	1.063	0.738346383
Proteasome assembly chaperone 2 [OS=Homo sapiens]	1.063	0.563524729
Cytoplasmic dynein 2 intermediate chain 2 [OS=Homo sapiens]	1.063	0.915517977
WD repeat-containing protein 5 [OS=Homo sapiens]	1.063	0.994751198
tRNA (guanine(10)-N2)-methyltransferase homolog [OS=Homo sapiens]	1.063	0.884277167
Probable ATP-dependent RNA helicase DDX59 [OS=Homo sapiens]	1.063	0.959668863
Cell cycle checkpoint protein RAD1 [OS=Homo sapiens]	1.063	0.999146941
DNA repair protein XRCC1 [OS=Homo sapiens]	1.063	0.996757892
ADP-ribosylation factor-like protein 13B [OS=Homo sapiens]	1.063	0.999940163
Bridge-like lipid transfer protein family member 2 [OS=Homo sapiens]	1.063	0.993984955
N-acetyllactosaminide beta-1,3-N-acetylglucosaminyltransferase 2 [OS=Homo	1.063	1
Cleavage and polyadenylation specificity factor subunit 6 [OS=Homo sapiens]	1.064	0.656257798
Four and a half LIM domains protein 1 [OS=Homo sapiens]	1.064	0.807055412
ATP synthase subunit O, mitochondrial [OS=Homo sapiens]	1.064	0.999993937
Actin-related protein 2/3 complex subunit 3 [OS=Homo sapiens]	1.064	0.793846892
Tight junction-associated protein 1 [OS=Homo sapiens]	1.064	0.790010392
Geranylgeranyl transferase type-1 subunit beta [OS=Homo sapiens]	1.064	0.20851521
Small glutamine-rich tetratricopeptide repeat-containing protein beta [OS=Hom	1.064	0.943740148
Coiled-coil domain-containing protein 91 [OS=Homo sapiens]	1.064	0.854475778
GPN-loop GTPase 1 [OS=Homo sapiens]	1.064	0.948082484
SH3 domain-binding protein 4 [OS=Homo sapiens]	1.064	0.672495228
HAUS augmin-like complex subunit 5 [OS=Homo sapiens]	1.064	0.984478932
LisH domain-containing protein ARMC9 [OS=Homo sapiens]	1.064	0.805125185
39S ribosomal protein L35, mitochondrial [OS=Homo sapiens]	1.064	0.999998667
EKC/KEOPS complex subunit LAGE3 [OS=Homo sapiens]	1.064	0.828799348
Rho GTPase-activating protein 27 [OS=Homo sapiens]	1.064	0.836453858
Adenosylhomocysteinase [OS=Homo sapiens]	1.065	0.524740972
RNA-splicing ligase RtcB homolog [OS=Homo sapiens]	1.065	0.110618114
Coatomer subunit gamma-2 [OS=Homo sapiens]	1.065	0.699666798
Glutaredoxin-3 [OS=Homo sapiens]	1.065	0.763014415
Exocyst complex component 2 [OS=Homo sapiens]	1.065	0.802551308
Tax1-binding protein 1 [OS=Homo sapiens]	1.065	0.264998642
Oxysterol-binding protein-related protein 9 [OS=Homo sapiens]	1.065	0.67355242
Platelet-activating factor acetylhydrolase IB subunit alpha2 [OS=Homo sapiens]	1.065	0.997483347
Cytochrome b5 type B [OS=Homo sapiens]	1.065	0.999997672
Actin-related protein 10 [OS=Homo sapiens]	1.065	0.995940428
Ribonuclease 3 [OS=Homo sapiens]	1.065	0.721235728
Protein BRICK1 [OS=Homo sapiens]	1.065	0.948865924
Magnesium transporter protein 1 [OS=Homo sapiens]	1.065	0.99936875
Copine-7 [OS=Homo sapiens]	1.065	0.720876334

tRNA N6-adenosine threonylcarbamoyltransferase, mitochondrial [OS=Homo sa	1.065	0.744307307
WD repeat, SAM and U-box domain-containing protein 1 [OS=Homo sapiens]	1.065	0.78157232
TBC1 domain family member 31 [OS=Homo sapiens]	1.065	0.812436866
Starch-binding domain-containing protein 1 [OS=Homo sapiens]	1.065	0.928993893
AT-rich interactive domain-containing protein 1A [OS=Homo sapiens]	1.066	0.233281007
Chondroitin sulfate proteoglycan 4 [OS=Homo sapiens]	1.066	0.809122813
Galactokinase [OS=Homo sapiens]	1.066	0.226650523
60S ribosomal protein L11 [OS=Homo sapiens]	1.066	0.461132846
Aldehyde dehydrogenase family 16 member A1 [OS=Homo sapiens]	1.066	0.92930293
Transcription elongation factor SPT4 [OS=Homo sapiens]	1.066	0.79994311
Ribulose-phosphate 3-epimerase [OS=Homo sapiens]	1.066	0.575602752
Mannosyl-oligosaccharide 1,2-alpha-mannosidase IB [OS=Homo sapiens]	1.066	0.874774918
L-lactate dehydrogenase B chain [OS=Homo sapiens]	1.067	0.916358762
Tubulin--tyrosine ligase-like protein 12 [OS=Homo sapiens]	1.067	0.947682215
Actin-related protein 3 [OS=Homo sapiens]	1.067	0.338536627
Endoplasmic reticulum resident protein 29 [OS=Homo sapiens]	1.067	0.559999379
SWI/SNF-related matrix-associated actin-dependent regulator of chromatin sub	1.067	0.236728833
Breast cancer anti-estrogen resistance protein 1 [OS=Homo sapiens]	1.067	0.696816762
2-methoxy-6-polyprenyl-1,4-benzoquinol methylase, mitochondrial [OS=Homo s	1.067	0.779949769
Cytochrome P450 20A1 [OS=Homo sapiens]	1.067	0.849864544
Neurobeachin-like protein 1 [OS=Homo sapiens]	1.067	0.506840323
28S ribosomal protein S11, mitochondrial [OS=Homo sapiens]	1.067	0.938504585
Intermembrane lipid transfer protein VPS13D [OS=Homo sapiens]	1.067	0.999953157
TAF6-like RNA polymerase II p300/CBP-associated factor-associated factor 65 k	1.067	0.999445597
Zinc finger CCHC-type and RNA-binding motif-containing protein 1 [OS=Homo s	1.067	0.993499165
Immunoglobulin superfamily member 8 [OS=Homo sapiens]	1.067	1
Testis-specific Y-encoded-like protein 2 [OS=Homo sapiens]	1.067	0.568577531
Ezrin [OS=Homo sapiens]	1.068	0.277449075
Eukaryotic translation initiation factor 5B [OS=Homo sapiens]	1.068	0.308059587
Sodium/potassium-transporting ATPase subunit alpha-1 [OS=Homo sapiens]	1.068	0.99999577
14-3-3 protein eta [OS=Homo sapiens]	1.068	0.390724387
Eukaryotic peptide chain release factor GTP-binding subunit ERF3A [OS=Homo s	1.068	0.964253507
Ubiquitin carboxyl-terminal hydrolase 10 [OS=Homo sapiens]	1.068	0.075938668
TBC1 domain family member 9B [OS=Homo sapiens]	1.068	0.148879964
Mitochondrial Rho GTPase 1 [OS=Homo sapiens]	1.068	0.933698662
Soluble lamin-associated protein of 75 kDa [OS=Homo sapiens]	1.068	0.999589355
Oxysterol-binding protein-related protein 2 [OS=Homo sapiens]	1.068	0.967742735
WAS protein family homolog 2 [OS=Homo sapiens]	1.068	0.842846932
Dolichyl-diphosphooligosaccharide--protein glycosyltransferase subunit DAD1 [	1.068	1
Ubiquitin-fold modifier-conjugating enzyme 1 [OS=Homo sapiens]	1.068	0.930648483
Sulfite oxidase, mitochondrial [OS=Homo sapiens]	1.068	0.747815926
Bublin coiled-coil protein [OS=Homo sapiens]	1.068	0.999976379
KxDL motif-containing protein 1 [OS=Homo sapiens]	1.068	0.677640787
Zinc finger and BTB domain-containing protein 10 [OS=Homo sapiens]	1.068	0.933330202
Nuclear pore complex-interacting protein family member B11 [OS=Homo sapier	1.068	0.999998652
26S proteasome non-ATPase regulatory subunit 2 [OS=Homo sapiens]	1.069	0.149006287
26S proteasome regulatory subunit 10B [OS=Homo sapiens]	1.069	0.355895417
40S ribosomal protein S19 [OS=Homo sapiens]	1.069	0.917616557
Cullin-7 [OS=Homo sapiens]	1.069	0.12146946
40S ribosomal protein S11 [OS=Homo sapiens]	1.069	0.811505866
Peroxisomal biogenesis factor 19 [OS=Homo sapiens]	1.069	0.677641913
40S ribosomal protein S28 [OS=Homo sapiens]	1.069	0.33542418
28S ribosomal protein S10, mitochondrial [OS=Homo sapiens]	1.069	0.586209678
Ras-related protein Rab-33B [OS=Homo sapiens]	1.069	0.97461872
General transcription and DNA repair factor IIH helicase subunit XPD [OS=Homo	1.069	0.392393609
Terminal uridylyltransferase 4 [OS=Homo sapiens]	1.069	0.318209624
EGF domain-specific O-linked N-acetylglucosamine transferase [OS=Homo sap	1.069	0.767717645
Mannose-P-dolichol utilization defect 1 protein [OS=Homo sapiens]	1.069	0.881589631
Transmembrane protein 11, mitochondrial [OS=Homo sapiens]	1.069	1
Ataxin-10 [OS=Homo sapiens]	1.07	0.398178908
Apolipoprotein L2 [OS=Homo sapiens]	1.07	0.332310882
U6 snRNA-associated Sm-like protein LSm2 [OS=Homo sapiens]	1.07	0.942619693
Charged multivesicular body protein 5 [OS=Homo sapiens]	1.07	0.498536248
Inosine-5'-monophosphate dehydrogenase 1 [OS=Homo sapiens]	1.07	0.417133724
39S ribosomal protein L43, mitochondrial [OS=Homo sapiens]	1.07	0.915579458
TBC1 domain family member 10A [OS=Homo sapiens]	1.07	0.844785657
Tudor domain-containing protein 7 [OS=Homo sapiens]	1.07	0.226987808
Zinc finger protein 64 [OS=Homo sapiens]	1.07	0.941397286
BTB/POZ domain-containing protein KCTD3 [OS=Homo sapiens]	1.07	0.99962618
Proline and serine-rich protein 2 [OS=Homo sapiens]	1.07	0.631156821
Zinc finger MYM-type protein 6 [OS=Homo sapiens]	1.07	0.425043767
E3 ubiquitin-protein ligase UBR4 [OS=Homo sapiens]	1.071	0.317002727
Tubulin beta-4A chain [OS=Homo sapiens]	1.071	0.348781987
COP9 signalosome complex subunit 4 [OS=Homo sapiens]	1.071	0.27243129
ATP-dependent RNA helicase DDX19A [OS=Homo sapiens]	1.071	0.057692394

40S ribosomal protein S10 [OS=Homo sapiens]	1.071	0.563201042
Macrophage migration inhibitory factor [OS=Homo sapiens]	1.071	0.986365321
Methionine synthase reductase [OS=Homo sapiens]	1.071	0.646931756
Sedoheptulokinase [OS=Homo sapiens]	1.071	0.976113964
2,4-dienoyl-CoA reductase [(3E)-enoyl-CoA-producing], mitochondrial [OS=Hom	1.071	0.111426748
Tyrosine-protein phosphatase non-receptor type 14 [OS=Homo sapiens]	1.071	0.644684234
Baculoviral IAP repeat-containing protein 2 [OS=Homo sapiens]	1.071	0.99188814
39S ribosomal protein L54, mitochondrial [OS=Homo sapiens]	1.071	0.782013549
Ephrin-B1 [OS=Homo sapiens]	1.071	0.937847971
Alpha-tubulin N-acetyltransferase 1 [OS=Homo sapiens]	1.071	0.960837518
Midasin [OS=Homo sapiens]	1.072	0.876657147
Transketolase [OS=Homo sapiens]	1.072	0.680386386
Exocyst complex component 4 [OS=Homo sapiens]	1.072	0.189762397
Charged multivesicular body protein 4a [OS=Homo sapiens]	1.072	0.161872176
FAS-associated death domain protein [OS=Homo sapiens]	1.072	0.741049106
Rab9 effector protein with kelch motifs [OS=Homo sapiens]	1.072	0.987370349
Acyl-coenzyme A thioesterase MBLAC2 [OS=Homo sapiens]	1.072	0.793930673
Inactive tyrosine-protein kinase PEAK1 [OS=Homo sapiens]	1.072	0.997582325
Major facilitator superfamily domain-containing protein 10 [OS=Homo sapiens]	1.072	0.985351539
Cellular tumor antigen p53 [OS=Homo sapiens]	1.073	0.583914341
45 kDa calcium-binding protein [OS=Homo sapiens]	1.073	0.667246618
Thioredoxin [OS=Homo sapiens]	1.073	0.806333718
Coronin-1B [OS=Homo sapiens]	1.073	0.15549862
AH receptor-interacting protein [OS=Homo sapiens]	1.073	0.097000894
Elongation factor 1-beta [OS=Homo sapiens]	1.073	0.340260631
Translation initiation factor eIF-2B subunit alpha [OS=Homo sapiens]	1.073	0.121230143
Glucose-induced degradation protein 8 homolog [OS=Homo sapiens]	1.073	0.193308319
Endoplasmic reticulum transmembrane helix translocase [OS=Homo sapiens]	1.073	0.950751413
Synaptosomal-associated protein 29 [OS=Homo sapiens]	1.073	0.608373611
39S ribosomal protein L3, mitochondrial [OS=Homo sapiens]	1.073	0.999728968
Inositol polyphosphate 5-phosphatase K [OS=Homo sapiens]	1.073	0.843979613
Lysosomal-trafficking regulator [OS=Homo sapiens]	1.073	0.169040496
Ras GTPase-activating protein nGAP [OS=Homo sapiens]	1.073	0.673272899
Tetratricopeptide repeat protein 9C [OS=Homo sapiens]	1.073	0.682274601
Mediator of RNA polymerase II transcription subunit 17 [OS=Homo sapiens]	1.073	0.963895192
Succinate dehydrogenase [ubiquinone] cytochrome b small subunit, mitochond	1.073	0.991819953
Ras and Rab interactor 1 [OS=Homo sapiens]	1.073	0.671819328
Protein YIF1B [OS=Homo sapiens]	1.073	0.5937832
Phenazine biosynthesis-like domain-containing protein [OS=Homo sapiens]	1.073	0.999982013
Serine/threonine-protein kinase 17A [OS=Homo sapiens]	1.073	0.986300123
TIR domain-containing adapter molecule 1 [OS=Homo sapiens]	1.073	0.517075726
Formin-like protein 1 [OS=Homo sapiens]	1.073	0.999932896
Calpastatin [OS=Homo sapiens]	1.074	0.918180751
40S ribosomal protein S3a [OS=Homo sapiens]	1.074	0.18636724
Eukaryotic translation initiation factor 3 subunit M [OS=Homo sapiens]	1.074	0.992125146
Nucleosome assembly protein 1-like 4 [OS=Homo sapiens]	1.074	0.754065943
40S ribosomal protein S17 [OS=Homo sapiens]	1.074	0.975534417
Ras-related protein Rab-2B [OS=Homo sapiens]	1.074	0.881863365
Cytosolic 5'-nucleotidase 3A [OS=Homo sapiens]	1.074	0.978709295
Tudor and KH domain-containing protein [OS=Homo sapiens]	1.074	0.903767641
Ran-binding protein 9 [OS=Homo sapiens]	1.074	0.660837171
Selenocysteine-specific elongation factor [OS=Homo sapiens]	1.074	0.144071633
Protein kinase C delta type [OS=Homo sapiens]	1.074	0.901797638
Translocon-associated protein subunit alpha [OS=Homo sapiens]	1.074	0.995682405
Ribosomal protein 63, mitochondrial [OS=Homo sapiens]	1.074	0.908572264
Triggering receptor expressed on myeloid cells 1 [OS=Homo sapiens]	1.074	0.992618488
Ubiquitin-associated protein 2-like [OS=Homo sapiens]	1.075	0.030529781
Importin-4 [OS=Homo sapiens]	1.075	0.806963763
Lysine-specific demethylase 3B [OS=Homo sapiens]	1.075	0.363431711
tRNA (guanine(26)-N(2))-dimethyltransferase [OS=Homo sapiens]	1.075	0.491903535
Pyridoxine-5'-phosphate oxidase [OS=Homo sapiens]	1.075	0.810592059
Arf-GAP domain and FG repeat-containing protein 1 [OS=Homo sapiens]	1.075	0.204164875
Exocyst complex component 8 [OS=Homo sapiens]	1.075	0.093271966
Translational activator of cytochrome c oxidase 1 [OS=Homo sapiens]	1.075	0.449713908
Core-binding factor subunit beta [OS=Homo sapiens]	1.075	0.40034076
Oxysterol-binding protein-related protein 5 [OS=Homo sapiens]	1.075	0.89624178
TBC1 domain family member 22A [OS=Homo sapiens]	1.075	0.444392738
Nucleic acid dioxygenase ALKBH1 [OS=Homo sapiens]	1.075	0.631317589
Acyl-CoA-binding domain-containing protein 6 [OS=Homo sapiens]	1.075	0.438357629
E3 ubiquitin-protein ligase MGRN1 [OS=Homo sapiens]	1.075	0.461277077
Translocator protein [OS=Homo sapiens]	1.075	0.983883818
SEC14-like protein 4 [OS=Homo sapiens]	1.075	0.897383587
Krev interaction trapped protein 1 [OS=Homo sapiens]	1.075	0.999999142
Trafficking protein particle complex subunit 9 [OS=Homo sapiens]	1.075	0.391648421
Cyclin-dependent kinase 19 [OS=Homo sapiens]	1.075	0.944568061

E3 ubiquitin-protein ligase HUWE1 [OS=Homo sapiens]	1.076	0.239773454
Tubulin alpha-4A chain [OS=Homo sapiens]	1.076	0.216390126
CLIP-associating protein 1 [OS=Homo sapiens]	1.076	0.351464044
14-3-3 protein theta [OS=Homo sapiens]	1.076	0.361791736
DENN domain-containing protein 4C [OS=Homo sapiens]	1.076	0.235136348
Phenylalanine--tRNA ligase alpha subunit [OS=Homo sapiens]	1.076	0.377519934
NBAS subunit of NRZ tethering complex [OS=Homo sapiens]	1.076	0.164831223
All-trans-retinol 13,14-reductase [OS=Homo sapiens]	1.076	0.072455443
Ubiquitin recognition factor in ER-associated degradation protein 1 [OS=Homo s	1.076	0.482578642
2'-deoxynucleoside 5'-phosphate N-hydrolase 1 [OS=Homo sapiens]	1.076	0.444030546
N6-adenosine-methyltransferase non-catalytic subunit [OS=Homo sapiens]	1.076	0.992298427
3'-5' RNA helicase YTHDC2 [OS=Homo sapiens]	1.076	0.904553193
DNA damage-binding protein 2 [OS=Homo sapiens]	1.076	0.631183981
Cyclin-T2 [OS=Homo sapiens]	1.076	0.999153191
NEDD8-conjugating enzyme UBE2F [OS=Homo sapiens]	1.076	0.980081824
Cofilin-1 [OS=Homo sapiens]	1.077	0.094378493
Twinfilin-2 [OS=Homo sapiens]	1.077	0.341592
Protein LYRIC [OS=Homo sapiens]	1.077	0.365334249
Leucine-rich repeat and WD repeat-containing protein 1 [OS=Homo sapiens]	1.077	0.736342612
Myeloid leukemia factor 2 [OS=Homo sapiens]	1.077	0.999999999
Actin-binding LIM protein 1 [OS=Homo sapiens]	1.077	0.485074985
ER membrane protein complex subunit 8 [OS=Homo sapiens]	1.077	0.965980254
Ergosterol biosynthetic protein 28 homolog [OS=Homo sapiens]	1.077	0.99997929
Transcription factor IIIA [OS=Homo sapiens]	1.077	0.998732389
Glycogen phosphorylase, brain form [OS=Homo sapiens]	1.078	0.342112547
Clustered mitochondria protein homolog [OS=Homo sapiens]	1.078	0.234359412
Delta-1-pyrroline-5-carboxylate synthase [OS=Homo sapiens]	1.078	0.02555484
Protein kinase C and casein kinase substrate in neurons protein 2 [OS=Homo sa	1.078	0.150876282
Tubulin-specific chaperone D [OS=Homo sapiens]	1.078	0.254751529
DnaJ homolog subfamily B member 11 [OS=Homo sapiens]	1.078	0.026621063
UTP--glucose-1-phosphate uridylyltransferase [OS=Homo sapiens]	1.078	0.224916351
Epidermal growth factor receptor substrate 15-like 1 [OS=Homo sapiens]	1.078	0.270770076
WD repeat-containing protein 70 [OS=Homo sapiens]	1.078	0.616198986
39S ribosomal protein L19, mitochondrial [OS=Homo sapiens]	1.078	0.9155825
RCC1-like G exchanging factor-like protein [OS=Homo sapiens]	1.078	0.314281279
Signal recognition particle 9 kDa protein [OS=Homo sapiens]	1.078	0.051443016
Prefoldin subunit 1 [OS=Homo sapiens]	1.078	0.290251376
Non-histone chromosomal protein HMG-17 [OS=Homo sapiens]	1.078	0.999999963
1-acyl-sn-glycerol-3-phosphate acyltransferase alpha [OS=Homo sapiens]	1.078	0.276228511
WD repeat-containing protein WRAP73 [OS=Homo sapiens]	1.078	0.915045227
KH homology domain-containing protein 4 [OS=Homo sapiens]	1.078	0.676153703
Protein 4.1 [OS=Homo sapiens]	1.078	0.985467349
Rab11 family-interacting protein 5 [OS=Homo sapiens]	1.078	0.978744125
ATP synthase protein 8 [OS=Homo sapiens]	1.078	0.999999451
Cytoskeleton-associated protein 5 [OS=Homo sapiens]	1.079	0.436089406
Transport and Golgi organization protein 1 homolog [OS=Homo sapiens]	1.079	0.03812325
POTE ankyrin domain family member E [OS=Homo sapiens]	1.079	0.865774915
Rab3 GTPase-activating protein catalytic subunit [OS=Homo sapiens]	1.079	0.778294812
Cullin-3 [OS=Homo sapiens]	1.079	0.054319574
Protein ecdysoneless homolog [OS=Homo sapiens]	1.079	0.58035876
Xylulose kinase [OS=Homo sapiens]	1.079	0.125384548
Inhibitor of nuclear factor kappa-B kinase subunit beta [OS=Homo sapiens]	1.079	0.410984754
tRNA (guanine(37)-N1)-methyltransferase [OS=Homo sapiens]	1.079	0.840214005
ADP-ribosylation factor-binding protein GGA1 [OS=Homo sapiens]	1.079	0.396746733
Syntaxin-5 [OS=Homo sapiens]	1.079	0.188559496
Bifunctional arginine demethylase and lysyl-hydroxylase JMJD6 [OS=Homo sapie	1.079	0.961691199
Phytanoyl-CoA dioxygenase, peroxisomal [OS=Homo sapiens]	1.079	0.462101644
GATOR complex protein WDR59 [OS=Homo sapiens]	1.079	0.158892627
2-oxoisovalerate dehydrogenase subunit beta, mitochondrial [OS=Homo sapien	1.079	0.346452352
Centriolin [OS=Homo sapiens]	1.079	0.231170306
E3 ubiquitin-protein ligase TRIM4 [OS=Homo sapiens]	1.079	0.999937035
Ankyrin repeat and SOCS box protein 13 [OS=Homo sapiens]	1.079	0.320158436
Transcription initiation factor TFIID subunit 9B [OS=Homo sapiens]	1.079	0.999421978
ATP-dependent RNA helicase DHX30 [OS=Homo sapiens]	1.08	0.045960195
RNA transcription, translation and transport factor protein [OS=Homo sapiens]	1.08	0.024342347
DnaJ homolog subfamily C member 3 [OS=Homo sapiens]	1.08	0.219775115
Atypical kinase COQ8A, mitochondrial [OS=Homo sapiens]	1.08	0.656187972
GTP-binding protein Rheb [OS=Homo sapiens]	1.08	0.160846497
RING finger protein 214 [OS=Homo sapiens]	1.08	0.089014837
Coiled-coil domain-containing protein 6 [OS=Homo sapiens]	1.08	0.717166851
Translocon-associated protein subunit delta [OS=Homo sapiens]	1.08	0.936666231
GA-binding protein alpha chain [OS=Homo sapiens]	1.08	0.910628293
Endoplasmic reticulum junction formation protein lunapark [OS=Homo sapiens]	1.08	0.06195875
Endonuclease 8-like 2 [OS=Homo sapiens]	1.08	0.755381442
Phosphatidylinositol 4-kinase beta [OS=Homo sapiens]	1.08	0.315205569

Nuclear receptor-binding protein [OS=Homo sapiens]	1.08	0.449969009
Rho GTPase-activating protein 35 [OS=Homo sapiens]	1.08	0.805357143
Dynein axonemal heavy chain 10 [OS=Homo sapiens]	1.08	0.9936531
SWI/SNF complex subunit SMARCC2 [OS=Homo sapiens]	1.081	0.755879686
Cytoplasmic FMR1-interacting protein 1 [OS=Homo sapiens]	1.081	0.77099406
Protein arginine N-methyltransferase 1 [OS=Homo sapiens]	1.081	0.439158571
Probable E3 ubiquitin-protein ligase HERC4 [OS=Homo sapiens]	1.081	0.361800774
26S proteasome non-ATPase regulatory subunit 9 [OS=Homo sapiens]	1.081	0.197218694
Tetratricopeptide repeat protein 1 [OS=Homo sapiens]	1.081	0.778668714
Protein FAM50A [OS=Homo sapiens]	1.081	0.334863634
Ubiquitin-conjugating enzyme E2 variant 2 [OS=Homo sapiens]	1.081	0.813648837
Calcium-regulated heat-stable protein 1 [OS=Homo sapiens]	1.081	0.622510278
Heat shock factor protein 1 [OS=Homo sapiens]	1.081	0.266638723
Xaa-Pro aminopeptidase 3 [OS=Homo sapiens]	1.081	0.469728265
Ubiquinone biosynthesis monooxygenase COQ6, mitochondrial [OS=Homo sapi	1.081	0.989552154
39S ribosomal protein L14, mitochondrial [OS=Homo sapiens]	1.081	0.997157662
Tudor domain-containing protein 3 [OS=Homo sapiens]	1.081	0.974854391
Mediator of RNA polymerase II transcription subunit 29 [OS=Homo sapiens]	1.081	0.854370026
Acyl-coenzyme A thioesterase 8 [OS=Homo sapiens]	1.081	0.991180322
Phosphatidylinositol 4,5-bisphosphate 3-kinase catalytic subunit beta isoform [(	1.081	0.204512752
Guanine nucleotide exchange factor MSS4 [OS=Homo sapiens]	1.081	0.916916434
MOB kinase activator 2 [OS=Homo sapiens]	1.081	0.455303296
SUMO-activating enzyme subunit 2 [OS=Homo sapiens]	1.082	0.497077171
Double-stranded RNA-binding protein Staufen homolog 1 [OS=Homo sapiens]	1.082	0.00916754
Phosphatidylinositol transfer protein beta isoform [OS=Homo sapiens]	1.082	0.448650868
Triple functional domain protein [OS=Homo sapiens]	1.082	0.646457766
Inactive C-alpha-formylglycine-generating enzyme 2 [OS=Homo sapiens]	1.082	0.19780956
Zinc finger ZZ-type and EF-hand domain-containing protein 1 [OS=Homo sapiens	1.082	0.528465823
GTPase NRas [OS=Homo sapiens]	1.082	0.68805617
Charged multivesicular body protein 3 [OS=Homo sapiens]	1.082	0.397417778
Enolase-phosphatase E1 [OS=Homo sapiens]	1.082	0.262191334
Heat shock 70 kDa protein 12A [OS=Homo sapiens]	1.082	0.55532084
Putative nascent polypeptide-associated complex subunit alpha-like protein [OS	1.082	0.834146824
Conserved oligomeric Golgi complex subunit 6 [OS=Homo sapiens]	1.082	0.741241275
RELT-like protein 1 [OS=Homo sapiens]	1.082	0.610959936
Receptor of activated protein C kinase 1 [OS=Homo sapiens]	1.083	0.698920406
Calponin-3 [OS=Homo sapiens]	1.083	0.232121889
6-phosphogluconate dehydrogenase, decarboxylating [OS=Homo sapiens]	1.083	0.259205785
26S proteasome non-ATPase regulatory subunit 13 [OS=Homo sapiens]	1.083	0.19288241
Interferon-induced 35 kDa protein [OS=Homo sapiens]	1.083	0.806809874
Eukaryotic translation elongation factor 1 epsilon-1 [OS=Homo sapiens]	1.083	0.189230902
PDZ and LIM domain protein 7 [OS=Homo sapiens]	1.083	0.21057566
Ubiquitin-conjugating enzyme E2 R1 [OS=Homo sapiens]	1.083	0.292424655
Inositol hexakisphosphate kinase 1 [OS=Homo sapiens]	1.083	0.694072185
Tumor necrosis factor receptor superfamily member 6 [OS=Homo sapiens]	1.083	0.886928564
Hypoxia-inducible factor 1-alpha inhibitor [OS=Homo sapiens]	1.083	0.984139619
Histone-lysine N-methyltransferase SETDB1 [OS=Homo sapiens]	1.083	0.999901755
Proteasome activator complex subunit 4 [OS=Homo sapiens]	1.084	0.073830448
E3 ubiquitin-protein ligase MYCBP2 [OS=Homo sapiens]	1.084	0.212946807
Receptor expression-enhancing protein 5 [OS=Homo sapiens]	1.084	0.037940369
Peroxisomal ATPase PEX1 [OS=Homo sapiens]	1.084	0.23828987
Negative elongation factor A [OS=Homo sapiens]	1.084	0.293796561
Seizure 6-like protein 2 [OS=Homo sapiens]	1.084	0.759121527
tRNA-specific adenosine deaminase 2 [OS=Homo sapiens]	1.084	0.693618505
Nck-associated protein 5-like [OS=Homo sapiens]	1.084	0.52668544
GAS2-like protein 3 [OS=Homo sapiens]	1.084	0.999517936
Importin-5 [OS=Homo sapiens]	1.085	0.677153148
AP-2 complex subunit alpha-2 [OS=Homo sapiens]	1.085	0.576144591
Lipopolysaccharide-responsive and beige-like anchor protein [OS=Homo sapien	1.085	0.467512781
Zyxin [OS=Homo sapiens]	1.085	0.079414098
Dihydroorotate dehydrogenase (quinone), mitochondrial [OS=Homo sapiens]	1.085	0.999321001
Vacuolar protein sorting-associated protein 26A [OS=Homo sapiens]	1.085	0.238023753
E3 ubiquitin-protein ligase CBL [OS=Homo sapiens]	1.085	0.673855898
Actin filament-associated protein 1-like 2 [OS=Homo sapiens]	1.085	0.752187453
Ubiquitin-conjugating enzyme E2 variant 1 [OS=Homo sapiens]	1.085	0.966338501
Peroxisomal acyl-coenzyme A oxidase 1 [OS=Homo sapiens]	1.085	0.29215537
Nicotinamide N-methyltransferase [OS=Homo sapiens]	1.085	0.444088207
Mothers against decapentaplegic homolog 4 [OS=Homo sapiens]	1.085	0.165489474
Mitochondrial antiviral-signaling protein [OS=Homo sapiens]	1.085	0.999889171
tRNA (adenine(58)-N(1))-methyltransferase, mitochondrial [OS=Homo sapiens]	1.085	0.919639076
DNA-directed RNA polymerase III subunit RPC3 [OS=Homo sapiens]	1.085	0.265340532
BTB/POZ domain-containing protein KCTD9 [OS=Homo sapiens]	1.085	0.99999591
Sphingosine-1-phosphate phosphatase 1 [OS=Homo sapiens]	1.085	0.957735178
Tubulin beta-4B chain [OS=Homo sapiens]	1.086	0.662811988
Nck-associated protein 1 [OS=Homo sapiens]	1.086	0.257237083

ATP-dependent RNA helicase DHX29 [OS=Homo sapiens]	1.086	0.057450441
CLIP-associating protein 2 [OS=Homo sapiens]	1.086	0.245930309
Importin subunit alpha-4 [OS=Homo sapiens]	1.086	0.038629237
Endothelial differentiation-related factor 1 [OS=Homo sapiens]	1.086	0.428693519
Twinfilin-1 [OS=Homo sapiens]	1.086	0.088592015
Syntaxin-4 [OS=Homo sapiens]	1.086	0.998753189
Paladin [OS=Homo sapiens]	1.086	0.068192707
Succinate dehydrogenase assembly factor 2, mitochondrial [OS=Homo sapiens]	1.086	0.493089753
Tyrosine-protein phosphatase non-receptor type 6 [OS=Homo sapiens]	1.086	0.617604738
Elongin-B [OS=Homo sapiens]	1.086	0.042166569
Nuclear receptor subfamily 2 group C member 2 [OS=Homo sapiens]	1.086	0.967380614
Rho guanine nucleotide exchange factor 28 [OS=Homo sapiens]	1.086	0.999800146
Enhancer of mRNA-decapping protein 4 [OS=Homo sapiens]	1.087	0.007509726
Alkyldihydroxyacetonephosphate synthase, peroxisomal [OS=Homo sapiens]	1.087	0.087788052
Tumor susceptibility gene 101 protein [OS=Homo sapiens]	1.087	0.364746917
Negative elongation factor B [OS=Homo sapiens]	1.087	0.396303328
V-type proton ATPase subunit C 1 [OS=Homo sapiens]	1.087	0.31409274
Ubiquitin carboxyl-terminal hydrolase 11 [OS=Homo sapiens]	1.087	0.641314789
Electron transfer flavoprotein-ubiquinone oxidoreductase, mitochondrial [OS=H	1.087	0.88997865
Phosphopantothenate--cysteine ligase [OS=Homo sapiens]	1.087	0.284505646
GMP reductase 2 [OS=Homo sapiens]	1.087	0.333318607
Proteasome inhibitor PI31 subunit [OS=Homo sapiens]	1.087	0.594612225
Receptor expression-enhancing protein 4 [OS=Homo sapiens]	1.087	0.630258455
Aprataxin [OS=Homo sapiens]	1.087	0.686841017
Chloride intracellular channel protein 4 [OS=Homo sapiens]	1.088	0.155892878
Helicase MOV-10 [OS=Homo sapiens]	1.088	0.35134202
Protein UXT [OS=Homo sapiens]	1.088	0.451130682
Phosphatidylinositide phosphatase SAC2 [OS=Homo sapiens]	1.088	0.816339613
Conserved oligomeric Golgi complex subunit 2 [OS=Homo sapiens]	1.088	0.129106228
Signal recognition particle 19 kDa protein [OS=Homo sapiens]	1.088	0.85726152
CCR4-NOT transcription complex subunit 6 [OS=Homo sapiens]	1.088	1
Refilin-B [OS=Homo sapiens]	1.088	0.999998164
Chronophin [OS=Homo sapiens]	1.088	0.705371004
Pleckstrin homology domain-containing family F member 1 [OS=Homo sapiens]	1.088	0.800296379
VPS35 endosomal protein-sorting factor-like [OS=Homo sapiens]	1.088	0.99995313
Protein sel-1 homolog 1 [OS=Homo sapiens]	1.088	0.893214208
Biogenesis of lysosome-related organelles complex 1 subunit 1 [OS=Homo sapi	1.088	0.536097197
Uncharacterized protein C2orf42 [OS=Homo sapiens]	1.088	0.927396725
CAP-Gly domain-containing linker protein 4 [OS=Homo sapiens]	1.088	0.758045343
Plasminogen activator inhibitor 1 RNA-binding protein [OS=Homo sapiens]	1.089	0.195298931
HBS1-like protein [OS=Homo sapiens]	1.089	0.377593194
GTPase-activating protein and VPS9 domain-containing protein 1 [OS=Homo sa	1.089	0.186079072
F-actin-capping protein subunit beta [OS=Homo sapiens]	1.089	0.267497203
Creatine kinase U-type, mitochondrial [OS=Homo sapiens]	1.089	0.667898346
Dihydrolipoyllysine-residue acetyltransferase component of pyruvate dehydroge	1.089	0.99999994
Elongator complex protein 2 [OS=Homo sapiens]	1.089	0.804997881
ADP-ribosylation factor GTPase-activating protein 2 [OS=Homo sapiens]	1.089	0.228554469
Calcium-transporting ATPase type 2C member 1 [OS=Homo sapiens]	1.089	0.986359973
BAG family molecular chaperone regulator 5 [OS=Homo sapiens]	1.089	0.978962356
Trafficking protein particle complex subunit 8 [OS=Homo sapiens]	1.089	0.25539135
Constitutive coactivator of peroxisome proliferator-activated receptor gamma [(	1.089	0.26947408
E3 ubiquitin-protein ligase RNF31 [OS=Homo sapiens]	1.089	0.867232641
F-box/LRR-repeat protein 15 [OS=Homo sapiens]	1.089	0.984493996
Citrate synthase, mitochondrial [OS=Homo sapiens]	1.09	0.688444244
COP9 signalosome complex subunit 2 [OS=Homo sapiens]	1.09	0.365955066
Lactadherin [OS=Homo sapiens]	1.09	0.635917726
E3 ubiquitin-protein ligase listerin [OS=Homo sapiens]	1.09	0.391104023
Vacuolar protein sorting-associated protein 4A [OS=Homo sapiens]	1.09	0.612681471
CD2 antigen cytoplasmic tail-binding protein 2 [OS=Homo sapiens]	1.09	0.343180628
Ras-related protein Rab-34 [OS=Homo sapiens]	1.09	0.423364966
Acyl-CoA dehydrogenase family member 11 [OS=Homo sapiens]	1.09	0.134337352
Kinesin-like protein KIF7 [OS=Homo sapiens]	1.09	0.645168602
Dermcidin [OS=Homo sapiens]	1.09	0.998443741
Neutral alpha-glucosidase AB [OS=Homo sapiens]	1.091	0.451434813
Pseudouridylate synthase 7 homolog [OS=Homo sapiens]	1.091	0.336947823
Cdc42-interacting protein 4 [OS=Homo sapiens]	1.091	0.210458516
WD repeat-containing protein 44 [OS=Homo sapiens]	1.091	0.274684932
Threonine--tRNA ligase, mitochondrial [OS=Homo sapiens]	1.091	0.300705655
Protein kinase C iota type [OS=Homo sapiens]	1.091	0.124782207
ARF GTPase-activating protein GIT2 [OS=Homo sapiens]	1.091	0.568165511
Minor histocompatibility antigen H13 [OS=Homo sapiens]	1.091	0.999709808
39S ribosomal protein L1, mitochondrial [OS=Homo sapiens]	1.091	0.855498914
Rap guanine nucleotide exchange factor 6 [OS=Homo sapiens]	1.091	0.054765663
Proteasome subunit beta type-8 [OS=Homo sapiens]	1.091	0.935691118
5-formyltetrahydrofolate cyclo-ligase [OS=Homo sapiens]	1.091	0.320601278

Mitofusin-1 [OS=Homo sapiens]	1.091	0.515308336
Vesicle transport protein USE1 [OS=Homo sapiens]	1.091	0.692646787
Sorting nexin-10 [OS=Homo sapiens]	1.091	0.693776635
MYG1 exonuclease [OS=Homo sapiens]	1.092	0.48020284
CUGBP Elav-like family member 1 [OS=Homo sapiens]	1.092	0.606108039
Heat shock 70 kDa protein 14 [OS=Homo sapiens]	1.092	0.115208366
Thioredoxin domain-containing protein 12 [OS=Homo sapiens]	1.092	0.878823241
Serine/threonine-protein phosphatase 2A catalytic subunit beta isoform [OS=Hc	1.092	0.182236268
RNA polymerase II-associated protein 1 [OS=Homo sapiens]	1.092	0.325600407
Probable aminopeptidase NPEPL1 [OS=Homo sapiens]	1.092	0.236821082
Acyl carrier protein, mitochondrial [OS=Homo sapiens]	1.092	0.369906306
Endophilin-B2 [OS=Homo sapiens]	1.092	0.126195603
Numb-like protein [OS=Homo sapiens]	1.092	0.398225359
Platelet-activating factor acetylhydrolase 2, cytoplasmic [OS=Homo sapiens]	1.092	0.341713483
Chondroitin sulfate glucuronyltransferase [OS=Homo sapiens]	1.092	0.984552986
Annexin A2 [OS=Homo sapiens]	1.093	0.207884893
Neutral cholesterol ester hydrolase 1 [OS=Homo sapiens]	1.093	0.55455292
Ras-related protein Rab-10 [OS=Homo sapiens]	1.093	0.671401052
BRISC and BRCA1-A complex member 1 [OS=Homo sapiens]	1.093	0.159091232
60S acidic ribosomal protein P1 [OS=Homo sapiens]	1.093	0.809571752
Survival motor neuron protein [OS=Homo sapiens]	1.093	0.000479479
DNA-directed RNA polymerases I, II, and III subunit RPABC3 [OS=Homo sapiens]	1.093	0.6025363
Proteasome subunit beta type-9 [OS=Homo sapiens]	1.093	0.838458919
1-phosphatidylinositol 4,5-bisphosphate phosphodiesterase gamma-2 [OS=Hor	1.093	0.475436804
DNA repair endonuclease XPF [OS=Homo sapiens]	1.093	0.483708205
Peroxisomal targeting signal 1 receptor [OS=Homo sapiens]	1.093	0.393297516
Protein NATD1 [OS=Homo sapiens]	1.093	0.82828958
3-oxo-5-alpha-steroid 4-dehydrogenase 1 [OS=Homo sapiens]	1.093	0.578108209
Hypoxia up-regulated protein 1 [OS=Homo sapiens]	1.094	0.188581167
Cytosol aminopeptidase [OS=Homo sapiens]	1.094	0.782036752
60S ribosomal protein L8 [OS=Homo sapiens]	1.094	0.866876202
Protein transport protein Sec24B [OS=Homo sapiens]	1.094	0.236733529
2'-5'-oligoadenylate synthase 3 [OS=Homo sapiens]	1.094	0.175020134
Mitochondrial import inner membrane translocase subunit Tim8 B [OS=Homo sa	1.094	0.045726474
Acyl-protein thioesterase 1 [OS=Homo sapiens]	1.094	0.088254917
Cytochrome c oxidase subunit NDUFA4 [OS=Homo sapiens]	1.094	0.95603907
Methylmalonate-semialdehyde dehydrogenase [acylating], mitochondrial [OS=H	1.094	0.430093748
RING1 and YY1-binding protein [OS=Homo sapiens]	1.094	0.994469889
Transcription elongation factor 1 homolog [OS=Homo sapiens]	1.094	0.876628179
Proton-activated chloride channel [OS=Homo sapiens]	1.094	0.540312545
ORM1-like protein 3 [OS=Homo sapiens]	1.094	0.902993512
Protein FMC1 homolog [OS=Homo sapiens]	1.094	0.986717526
FACT complex subunit SSRP1 [OS=Homo sapiens]	1.095	0.972960972
Aminopeptidase B [OS=Homo sapiens]	1.095	0.185443701
Endophilin-A2 [OS=Homo sapiens]	1.095	0.296078513
Protein MON2 homolog [OS=Homo sapiens]	1.095	0.908513932
Golgi reassembly-stacking protein 2 [OS=Homo sapiens]	1.095	0.201352346
Histone acetyltransferase type B catalytic subunit [OS=Homo sapiens]	1.095	0.646190463
Glycerol-3-phosphate phosphatase [OS=Homo sapiens]	1.095	0.176407439
Cysteine-rich PDZ-binding protein [OS=Homo sapiens]	1.095	0.740762902
MIF4G domain-containing protein [OS=Homo sapiens]	1.095	0.096237574
Dihydroxyacetone phosphate acyltransferase [OS=Homo sapiens]	1.095	0.378437402
Immediate early response 3-interacting protein 1 [OS=Homo sapiens]	1.095	0.864302142
Cell cycle control protein 50A [OS=Homo sapiens]	1.095	0.999994157
Protein transport protein Sec16A [OS=Homo sapiens]	1.096	0.003879405
Formin-binding protein 1 [OS=Homo sapiens]	1.096	0.090011512
26S proteasome non-ATPase regulatory subunit 6 [OS=Homo sapiens]	1.096	0.108433992
5'-AMP-activated protein kinase catalytic subunit alpha-1 [OS=Homo sapiens]	1.096	0.059192188
Ubiquitin-conjugating enzyme E2 L3 [OS=Homo sapiens]	1.096	0.524184626
Coiled-coil domain-containing protein 25 [OS=Homo sapiens]	1.096	0.186350785
Vacuolar protein sorting-associated protein VTA1 homolog [OS=Homo sapiens]	1.096	0.176901114
Calpain-7 [OS=Homo sapiens]	1.096	0.107357442
Bridge-like lipid transfer protein family member 3A [OS=Homo sapiens]	1.096	0.101301116
Paraspeckle component 1 [OS=Homo sapiens]	1.096	0.260210195
Protein NDRG3 [OS=Homo sapiens]	1.096	0.093920299
Nuclear factor NF-kappa-B p105 subunit [OS=Homo sapiens]	1.096	0.420389451
Poly(A) RNA polymerase, mitochondrial [OS=Homo sapiens]	1.096	0.979243641
Vacuolar protein sorting-associated protein 28 homolog [OS=Homo sapiens]	1.096	0.121491293
DNA-directed RNA polymerase III subunit RPC9 [OS=Homo sapiens]	1.096	0.952910744
Glycosylphosphatidylinositol anchor attachment 1 protein [OS=Homo sapiens]	1.096	0.925378695
Pleckstrin-2 [OS=Homo sapiens]	1.096	0.997571376
Solute carrier family 2, facilitated glucose transporter member 6 [OS=Homo sap	1.096	0.996249325
Phosphatidylinositol N-acetylglucosaminyltransferase subunit A [OS=Homo sap	1.096	0.837533506
Selenoprotein S [OS=Homo sapiens]	1.096	0.896304307
Ubiquitin carboxyl-terminal hydrolase 5 [OS=Homo sapiens]	1.097	0.54206866

ATP-dependent 6-phosphofructokinase, liver type [OS=Homo sapiens]	1.097	0.318562174
Platelet-activating factor acetylhydrolase IB subunit beta [OS=Homo sapiens]	1.097	0.387404895
COP9 signalosome complex subunit 5 [OS=Homo sapiens]	1.097	0.229920201
Thioredoxin-like protein 1 [OS=Homo sapiens]	1.097	0.185142236
Ubiquitin-conjugating enzyme E2 J1 [OS=Homo sapiens]	1.097	0.893330232
DDB1- and CUL4-associated factor 7 [OS=Homo sapiens]	1.097	0.171499327
Matrix-remodeling-associated protein 7 [OS=Homo sapiens]	1.097	0.888699735
Succinate dehydrogenase assembly factor 1, mitochondrial [OS=Homo sapiens]	1.097	0.858675055
Protein Z-dependent protease inhibitor [OS=Homo sapiens]	1.097	0.842132344
T-cell immunomodulatory protein [OS=Homo sapiens]	1.097	0.999871696
WD repeat-containing protein 1 [OS=Homo sapiens]	1.098	0.015265202
AT-rich interactive domain-containing protein 1B [OS=Homo sapiens]	1.098	0.378941551
GTP:AMP phosphotransferase AK3, mitochondrial [OS=Homo sapiens]	1.098	0.314949508
Mitogen-activated protein kinase 3 [OS=Homo sapiens]	1.098	0.551127001
Protein AAR2 homolog [OS=Homo sapiens]	1.098	0.330911797
DENN domain-containing protein 10 [OS=Homo sapiens]	1.098	0.451706004
Glutamyl-tRNA(Gln) amidotransferase subunit B, mitochondrial [OS=Homo sapi	1.098	0.251515263
Mitochondrial import inner membrane translocase subunit TIM14 [OS=Homo sa	1.098	0.988110051
Mitochondrial fission factor [OS=Homo sapiens]	1.098	0.931547471
Neugrin [OS=Homo sapiens]	1.098	0.680791299
Intercellular adhesion molecule 5 [OS=Homo sapiens]	1.098	0.976673305
Kazrin [OS=Homo sapiens]	1.098	0.336673992
ATP-dependent RNA helicase DDX3X [OS=Homo sapiens]	1.099	0.000134191
14-3-3 protein gamma [OS=Homo sapiens]	1.099	0.068052189
WAS/WASL-interacting protein family member 2 [OS=Homo sapiens]	1.099	0.326660062
Roundabout homolog 1 [OS=Homo sapiens]	1.099	0.263162398
HCLS1-binding protein 3 [OS=Homo sapiens]	1.099	0.289831354
Diphthine methyl ester synthase [OS=Homo sapiens]	1.099	0.706326523
Beclin-1 [OS=Homo sapiens]	1.099	0.737574572
Ubiquitin-conjugating enzyme E2 Q1 [OS=Homo sapiens]	1.099	0.135739514
Clathrin heavy chain 1 [OS=Homo sapiens]	1.1	0.048830398
Valine--tRNA ligase [OS=Homo sapiens]	1.1	0.009475216
Dolichyl-diphosphooligosaccharide--protein glycosyltransferase subunit 2 [OS=	1.1	0.958732357
Ankyrin repeat domain-containing protein 17 [OS=Homo sapiens]	1.1	0.399902579
Serine/threonine-protein kinase 10 [OS=Homo sapiens]	1.1	0.155015279
UDP-glucose 4-epimerase [OS=Homo sapiens]	1.1	0.061119444
Kynurenine--oxoglutarate transaminase 3 [OS=Homo sapiens]	1.1	0.042777988
5'-AMP-activated protein kinase subunit gamma-1 [OS=Homo sapiens]	1.1	0.14436279
RNA polymerase II subunit A C-terminal domain phosphatase SSU72 [OS=Homo	1.1	0.253165019
ERI1 exoribonuclease 3 [OS=Homo sapiens]	1.1	0.746719093
Gem-associated protein 8 [OS=Homo sapiens]	1.1	0.773415837
Polyribonucleotide nucleotidyltransferase 1, mitochondrial [OS=Homo sapiens]	1.101	0.200198876
E3 ubiquitin-protein ligase HECTD1 [OS=Homo sapiens]	1.101	0.115059932
Proteasome subunit alpha type-6 [OS=Homo sapiens]	1.101	0.254103066
Protein TFG [OS=Homo sapiens]	1.101	0.150385547
Metastasis-associated protein MTA1 [OS=Homo sapiens]	1.101	0.023088812
Ubiquitin carboxyl-terminal hydrolase 15 [OS=Homo sapiens]	1.101	0.150364999
Serine/threonine-protein phosphatase 2A activator [OS=Homo sapiens]	1.101	0.178158087
Collagen alpha-1(VI) chain [OS=Homo sapiens]	1.101	0.193396415
Transcription factor p65 [OS=Homo sapiens]	1.101	0.082160842
General transcription factor IIE subunit 1 [OS=Homo sapiens]	1.101	0.048095532
Signal-induced proliferation-associated protein 1 [OS=Homo sapiens]	1.101	0.214267298
Iron-sulfur clusters transporter ABCB7, mitochondrial [OS=Homo sapiens]	1.101	0.986860095
Coiled-coil domain-containing protein 50 [OS=Homo sapiens]	1.101	0.872640761
TNF receptor-associated factor 1 [OS=Homo sapiens]	1.101	0.092624036
Septin-11 [OS=Homo sapiens]	1.102	0.588940249
Nuclear protein localization protein 4 homolog [OS=Homo sapiens]	1.102	0.074744206
Sulfhydryl oxidase 2 [OS=Homo sapiens]	1.102	0.938941114
Perilipin-2 [OS=Homo sapiens]	1.102	0.590128458
Vacuolar protein-sorting-associated protein 36 [OS=Homo sapiens]	1.102	0.257269096
Protein mono-ADP-ribosyltransferase PARP14 [OS=Homo sapiens]	1.102	0.356677743
Maleylacetoacetate isomerase [OS=Homo sapiens]	1.102	0.428775607
EH domain-containing protein 2 [OS=Homo sapiens]	1.102	0.254433372
N-terminal Xaa-Pro-Lys N-methyltransferase 1 [OS=Homo sapiens]	1.102	0.913370121
Protein C10 [OS=Homo sapiens]	1.102	0.421902484
ATP synthase subunit e, mitochondrial [OS=Homo sapiens]	1.102	0.999999988
GEM-interacting protein [OS=Homo sapiens]	1.102	0.065470564
Trafficking protein particle complex subunit 10 [OS=Homo sapiens]	1.102	0.695586658
Succinate dehydrogenase cytochrome b560 subunit, mitochondrial [OS=Homo	1.102	0.999987698
Apolipoprotein C-I [OS=Homo sapiens]	1.102	0.902872589
Serine/threonine-protein kinase Nek1 [OS=Homo sapiens]	1.102	0.788731386
Serine/threonine-protein phosphatase 2A 65 kDa regulatory subunit A alpha isof	1.103	0.123104696
Triokinase/FMN cyclase [OS=Homo sapiens]	1.103	0.224647782
Proteasome subunit beta type-7 [OS=Homo sapiens]	1.103	0.116165921
60S ribosomal protein L22 [OS=Homo sapiens]	1.103	0.853101225

Integrin-linked kinase-associated serine/threonine phosphatase 2C [OS=Homo	1.103	0.957786607
Heat shock factor-binding protein 1 [OS=Homo sapiens]	1.103	0.369618187
Stromal cell-derived factor 2-like protein 1 [OS=Homo sapiens]	1.103	0.009710362
Polyadenylate-binding protein-interacting protein 1 [OS=Homo sapiens]	1.103	0.093603355
Neurogenic locus notch homolog protein 2 [OS=Homo sapiens]	1.103	0.084751687
Ubiquitin-conjugating enzyme E2 G1 [OS=Homo sapiens]	1.103	0.728617512
RAC-alpha serine/threonine-protein kinase [OS=Homo sapiens]	1.103	0.102858812
E3 ubiquitin-protein ligase NEDD4 [OS=Homo sapiens]	1.103	0.055986744
COP9 signalosome complex subunit 6 [OS=Homo sapiens]	1.103	0.201924637
Nebulette [OS=Homo sapiens]	1.103	0.128482902
Ceramide synthase 2 [OS=Homo sapiens]	1.103	0.966367565
Serine/threonine-protein kinase 3 [OS=Homo sapiens]	1.103	0.167246777
SAC3 domain-containing protein 1 [OS=Homo sapiens]	1.103	0.667678941
Surfeit locus protein 1 [OS=Homo sapiens]	1.103	0.999999998
Signal peptidase complex subunit 2 [OS=Homo sapiens]	1.104	0.947906497
Proteasome subunit beta type-2 [OS=Homo sapiens]	1.104	0.459623926
Monocarboxylate transporter 1 [OS=Homo sapiens]	1.104	0.999882096
Exocyst complex component 7 [OS=Homo sapiens]	1.104	0.405641554
PHD finger protein 23 [OS=Homo sapiens]	1.104	0.184227546
Retrotransposon Gag-like protein 8A [OS=Homo sapiens]	1.104	0.901041477
Ankyrin repeat and SOCS box protein 3 [OS=Homo sapiens]	1.104	0.633694787
Talin-1 [OS=Homo sapiens]	1.105	0.055853269
AP-2 complex subunit beta [OS=Homo sapiens]	1.105	0.037536578
Mitochondrial import inner membrane translocase subunit TIM44 [OS=Homo sa	1.105	0.205770584
Large proline-rich protein BAG6 [OS=Homo sapiens]	1.105	0.035618137
UV excision repair protein RAD23 homolog B [OS=Homo sapiens]	1.105	0.08616643
DnaJ homolog subfamily C member 2 [OS=Homo sapiens]	1.105	0.144840112
BOS complex subunit NCLN [OS=Homo sapiens]	1.105	0.026103779
Mitochondrial import inner membrane translocase subunit TIM50 [OS=Homo sa	1.105	0.110517689
F-actin-capping protein subunit alpha-2 [OS=Homo sapiens]	1.105	0.042052589
N-acetylgalactosamine-6-sulfatase [OS=Homo sapiens]	1.105	0.126154129
Cdc42 effector protein 4 [OS=Homo sapiens]	1.105	0.832390525
EH domain-binding protein 1 [OS=Homo sapiens]	1.105	0.459925816
RING finger protein unkempt homolog [OS=Homo sapiens]	1.105	0.052674389
C2 domain-containing protein 5 [OS=Homo sapiens]	1.105	0.056687825
Putative pre-mRNA-splicing factor ATP-dependent RNA helicase DHX32 [OS=Ho	1.105	0.285756156
Trafficking protein particle complex subunit 2-like protein [OS=Homo sapiens]	1.105	0.313149896
GRB2-associated-binding protein 2 [OS=Homo sapiens]	1.105	0.546241183
Adenylosuccinate lyase [OS=Homo sapiens]	1.106	0.056007808
Helicase with zinc finger domain 2 [OS=Homo sapiens]	1.106	0.00888272
Deoxyhypusine hydroxylase [OS=Homo sapiens]	1.106	0.367353473
Protocadherin Fat 1 [OS=Homo sapiens]	1.106	0.137102096
Acyl-CoA:lysophosphatidylglycerol acyltransferase 1 [OS=Homo sapiens]	1.106	0.999228298
Conserved oligomeric Golgi complex subunit 3 [OS=Homo sapiens]	1.106	0.149729234
Serine/threonine-protein kinase Chk1 [OS=Homo sapiens]	1.106	0.97899402
BTB/POZ domain-containing protein 1 [OS=Homo sapiens]	1.106	0.035393955
Cilia- and flagella-associated protein 410 [OS=Homo sapiens]	1.106	0.443317086
SUMO-activating enzyme subunit 1 [OS=Homo sapiens]	1.107	0.418072174
Reticulocalbin-3 [OS=Homo sapiens]	1.107	0.248196582
ATP-dependent Clp protease ATP-binding subunit clpX-like, mitochondrial [OS=I	1.107	0.410283582
E3 ubiquitin-protein ligase CHIP [OS=Homo sapiens]	1.107	0.02021286
UBX domain-containing protein 7 [OS=Homo sapiens]	1.107	0.032783293
Thymocyte nuclear protein 1 [OS=Homo sapiens]	1.107	0.026854349
SNARE-associated protein Snapin [OS=Homo sapiens]	1.107	0.724785865
Dehydrogenase/reductase SDR family member 11 [OS=Homo sapiens]	1.107	0.626080736
Serine/threonine-protein kinase LATS1 [OS=Homo sapiens]	1.107	0.207266246
Coiled-coil domain-containing protein 97 [OS=Homo sapiens]	1.107	0.328292292
Interleukin-17 receptor A [OS=Homo sapiens]	1.107	0.892748714
Rho GTPase-activating protein 39 [OS=Homo sapiens]	1.107	0.735186915
E3 ubiquitin-protein ligase UBR5 [OS=Homo sapiens]	1.108	0.048778019
Zinc finger CCCH-type antiviral protein 1 [OS=Homo sapiens]	1.108	0.003165445
Ras-related protein Rab-1B [OS=Homo sapiens]	1.108	0.237892242
Annexin A11 [OS=Homo sapiens]	1.108	0.221297287
Protein-L-isoaspartate(D-aspartate) O-methyltransferase [OS=Homo sapiens]	1.108	0.727685258
28 kDa heat- and acid-stable phosphoprotein [OS=Homo sapiens]	1.108	0.020440473
Nucleoside diphosphate kinase A [OS=Homo sapiens]	1.108	0.473526936
Protein dopey-2 [OS=Homo sapiens]	1.108	0.35100269
Putative sodium-coupled neutral amino acid transporter 10 [OS=Homo sapiens]	1.108	0.742253228
60S ribosomal protein L30 [OS=Homo sapiens]	1.108	0.495691936
Protein farnesyltransferase/geranylgeranyltransferase type-1 subunit alpha [OS=	1.108	0.275222958
COMM domain-containing protein 1 [OS=Homo sapiens]	1.108	0.393543297
Urokinase-type plasminogen activator [OS=Homo sapiens]	1.108	0.277255682
5-demethoxyubiquinone hydroxylase, mitochondrial [OS=Homo sapiens]	1.108	0.811697386
Male-enhanced antigen 1 [OS=Homo sapiens]	1.108	0.983129868
Receptor-type tyrosine-protein phosphatase gamma [OS=Homo sapiens]	1.108	0.993232467

DNA-directed RNA polymerase III subunit RPC7 [OS=Homo sapiens]	1.108	0.38299801
UbiA prenyltransferase domain-containing protein 1 [OS=Homo sapiens]	1.108	0.854016239
Homer protein homolog 3 [OS=Homo sapiens]	1.108	0.942085841
Solute carrier family 22 member 18 [OS=Homo sapiens]	1.108	0.159609137
Breast cancer anti-estrogen resistance protein 3 [OS=Homo sapiens]	1.108	0.853873672
Glycerate kinase [OS=Homo sapiens]	1.108	0.999710339
E3 ubiquitin-protein ligase RNF185 [OS=Homo sapiens]	1.108	0.616029751
Enolase 4 [OS=Homo sapiens]	1.108	0.31312775
Serine/threonine-protein kinase N2 [OS=Homo sapiens]	1.109	0.114272989
Tubulin-folding cofactor B [OS=Homo sapiens]	1.109	0.039050747
40S ribosomal protein S16 [OS=Homo sapiens]	1.109	0.603373412
DNA-directed RNA polymerase, mitochondrial [OS=Homo sapiens]	1.109	0.399747091
Dynactin subunit 4 [OS=Homo sapiens]	1.109	0.099578308
Coatomer subunit epsilon [OS=Homo sapiens]	1.109	0.13090899
ATPase WRNIP1 [OS=Homo sapiens]	1.109	0.061951444
WD repeat-containing protein 48 [OS=Homo sapiens]	1.109	0.900206885
Exopolyphosphatase PRUNE1 [OS=Homo sapiens]	1.109	0.267680061
F-box/WD repeat-containing protein 1A [OS=Homo sapiens]	1.109	0.997292319
BOS complex subunit TMEM147 [OS=Homo sapiens]	1.109	0.830793749
Active breakpoint cluster region-related protein [OS=Homo sapiens]	1.11	0.098875522
Exocyst complex component 5 [OS=Homo sapiens]	1.11	0.10742986
Unconventional myosin-XVIIIa [OS=Homo sapiens]	1.11	0.226311994
Macrophage-capping protein [OS=Homo sapiens]	1.11	0.158990525
Protein SEC13 homolog [OS=Homo sapiens]	1.11	0.425582818
Dimethyladenosine transferase 2, mitochondrial [OS=Homo sapiens]	1.11	0.130776074
Mitochondrial ribosome-associated GTPase 1 [OS=Homo sapiens]	1.11	0.916696472
TRAF-type zinc finger domain-containing protein 1 [OS=Homo sapiens]	1.11	0.869755203
E3 ubiquitin-protein ligase ZNRF2 [OS=Homo sapiens]	1.11	0.756975298
U8 snoRNA-decapping enzyme [OS=Homo sapiens]	1.11	0.40975077
Zinc finger protein 131 [OS=Homo sapiens]	1.11	0.995787703
E3 ubiquitin-protein ligase RNF149 [OS=Homo sapiens]	1.11	0.996456318
Atrial natriuretic peptide receptor 2 [OS=Homo sapiens]	1.11	0.71741193
DENN domain-containing protein 2B [OS=Homo sapiens]	1.11	0.966633723
Endoplasmic reticulum chaperone BiP [OS=Homo sapiens]	1.111	0.025582177
Golgin subfamily B member 1 [OS=Homo sapiens]	1.111	0.999985543
Nicotinamide phosphoribosyltransferase [OS=Homo sapiens]	1.111	0.20297748
Adenylosuccinate synthetase isozyme 2 [OS=Homo sapiens]	1.111	0.055217007
Calmodulin-regulated spectrin-associated protein 1 [OS=Homo sapiens]	1.111	0.063996704
Rho guanine nucleotide exchange factor 12 [OS=Homo sapiens]	1.111	0.093016639
Alpha-ketoglutarate dehydrogenase component 4 [OS=Homo sapiens]	1.111	0.718899919
Platelet-activating factor acetylhydrolase IB subunit alpha1 [OS=Homo sapiens]	1.111	0.035413215
rRNA methyltransferase 3, mitochondrial [OS=Homo sapiens]	1.111	0.197291788
Transcription elongation factor A protein-like 1 [OS=Homo sapiens]	1.111	0.500309932
ATP synthase membrane subunit K, mitochondrial [OS=Homo sapiens]	1.111	0.999617071
DNA-directed RNA polymerase III subunit RPC4 [OS=Homo sapiens]	1.111	0.879053779
Protein-L-histidine N-pros-methyltransferase [OS=Homo sapiens]	1.111	0.626344998
Dedicator of cytokinesis protein 9 [OS=Homo sapiens]	1.111	0.959128847
Sialin [OS=Homo sapiens]	1.111	0.741125974
L-xylulose reductase [OS=Homo sapiens]	1.112	0.185044206
Tubulin-specific chaperone C [OS=Homo sapiens]	1.112	0.565205446
Methionine aminopeptidase 1 [OS=Homo sapiens]	1.112	0.063304072
NEDD8 [OS=Homo sapiens]	1.112	0.166013543
Serine/threonine-protein kinase TAO2 [OS=Homo sapiens]	1.112	0.095294647
Proteoglycan 4 [OS=Homo sapiens]	1.112	0.998191772
Profilin-1 [OS=Homo sapiens]	1.113	0.310435392
Protein mono-ADP-ribosyltransferase PARP4 [OS=Homo sapiens]	1.113	0.025775681
Proteasome subunit beta type-3 [OS=Homo sapiens]	1.113	0.73182856
Bifunctional UDP-N-acetylglucosamine 2-epimerase/N-acetylmannosamine kin	1.113	0.168622486
UDP-N-acetylhexosamine pyrophosphorylase-like protein 1 [OS=Homo sapiens]	1.113	0.863300553
Galectin-3 [OS=Homo sapiens]	1.113	0.220167775
HEAT repeat-containing protein 3 [OS=Homo sapiens]	1.113	0.277029553
Forkhead box protein K2 [OS=Homo sapiens]	1.113	0.270842934
Methionine--tRNA ligase, mitochondrial [OS=Homo sapiens]	1.113	0.673479069
Cellular retinoic acid-binding protein 1 [OS=Homo sapiens]	1.113	0.996102098
Testis-expressed protein 2 [OS=Homo sapiens]	1.113	0.698956564
N-alpha-acetyltransferase 30 [OS=Homo sapiens]	1.113	0.989211385
Protein AMBP [OS=Homo sapiens]	1.113	0.843039029
Complement component 1 Q subcomponent-binding protein, mitochondrial [OS	1.114	0.0846089
Translationally-controlled tumor protein [OS=Homo sapiens]	1.114	0.245821949
Prolactin regulatory element-binding protein [OS=Homo sapiens]	1.114	0.004585345
GRB10-interacting GYF protein 1 [OS=Homo sapiens]	1.114	0.25784745
R3H domain-containing protein 1 [OS=Homo sapiens]	1.114	0.322188044
Protein RFT1 homolog [OS=Homo sapiens]	1.114	0.957125893
Signal peptide peptidase-like 2A [OS=Homo sapiens]	1.114	0.687423259
Cancer-related nucleoside-triphosphatase [OS=Homo sapiens]	1.114	0.851744798

WASH complex subunit 3 [OS=Homo sapiens]	1.114	0.514970082
Ferredoxin-2, mitochondrial [OS=Homo sapiens]	1.114	0.64809517
Prostaglandin E synthase 2 [OS=Homo sapiens]	1.115	0.125584153
AMP deaminase 2 [OS=Homo sapiens]	1.115	0.06731778
ATP-dependent DNA/RNA helicase DHX36 [OS=Homo sapiens]	1.115	0.006666493
Bifunctional 3'-phosphoadenosine 5'-phosphosulfate synthase 2 [OS=Homo sap	1.115	0.10128945
Phosphoribosyl pyrophosphate synthase-associated protein 2 [OS=Homo sapie	1.115	0.199865285
Liprin-beta-1 [OS=Homo sapiens]	1.115	0.084670843
Coiled-coil and C2 domain-containing protein 1B [OS=Homo sapiens]	1.115	0.05994472
Carbonic anhydrase 2 [OS=Homo sapiens]	1.115	0.87526702
COMM domain-containing protein 10 [OS=Homo sapiens]	1.115	0.984310271
Type II inositol 1,4,5-trisphosphate 5-phosphatase [OS=Homo sapiens]	1.115	0.079443993
Deaminated glutathione amidase [OS=Homo sapiens]	1.115	0.220600398
Roquin-2 [OS=Homo sapiens]	1.115	0.868319631
Serine/threonine-protein kinase STK11 [OS=Homo sapiens]	1.115	0.252918055
Golgin subfamily A member 8J [OS=Homo sapiens]	1.115	0.658561583
182 kDa tankyrase-1-binding protein [OS=Homo sapiens]	1.116	0.036463069
NADH dehydrogenase [ubiquinone] 1 alpha subcomplex assembly factor 2 [OS=	1.116	0.01446412
Serine/threonine-protein kinase 26 [OS=Homo sapiens]	1.116	0.216335802
Vacuolar protein sorting-associated protein 53 homolog [OS=Homo sapiens]	1.116	0.309552785
Zinc finger C2HC domain-containing protein 1A [OS=Homo sapiens]	1.116	0.017903715
Charged multivesicular body protein 2a [OS=Homo sapiens]	1.116	0.729311866
Complex III assembly factor LYRM7 [OS=Homo sapiens]	1.116	0.059938563
BCAS3 microtubule associated cell migration factor [OS=Homo sapiens]	1.116	0.346397523
LINE-1 retrotransposable element ORF1 protein [OS=Homo sapiens]	1.116	0.99999891
Vesicle transport protein SEC20 [OS=Homo sapiens]	1.116	0.108833928
Endonuclease domain-containing 1 protein [OS=Homo sapiens]	1.116	0.999865787
Mediator of RNA polymerase II transcription subunit 13-like [OS=Homo sapiens]	1.116	0.097971239
F-box/LRR-repeat protein 6 [OS=Homo sapiens]	1.116	0.339493504
Mitochondrial import inner membrane translocase subunit TIM16 [OS=Homo sa	1.116	0.997853164
Cullin-5 [OS=Homo sapiens]	1.117	0.098128481
Hsc70-interacting protein [OS=Homo sapiens]	1.117	0.250531472
Eukaryotic translation initiation factor 4E [OS=Homo sapiens]	1.117	0.807373981
Inner nuclear membrane protein Man1 [OS=Homo sapiens]	1.117	0.99625557
Coatomer subunit zeta-1 [OS=Homo sapiens]	1.117	0.47750961
Tripartite motif-containing protein 2 [OS=Homo sapiens]	1.117	0.18884946
Protein Dr1 [OS=Homo sapiens]	1.117	0.037273225
Separin [OS=Homo sapiens]	1.117	0.853283225
Acidic fibroblast growth factor intracellular-binding protein [OS=Homo sapiens]	1.117	0.079906017
COMM domain-containing protein 5 [OS=Homo sapiens]	1.117	0.914753198
Transcription termination factor 4, mitochondrial [OS=Homo sapiens]	1.117	0.579039171
PHD finger protein 20-like protein 1 [OS=Homo sapiens]	1.117	0.999986318
Phosphatidate cytidylyltransferase 2 [OS=Homo sapiens]	1.117	0.942137503
Ubiquitin carboxyl-terminal hydrolase 24 [OS=Homo sapiens]	1.118	0.047325006
Casein kinase II subunit alpha [OS=Homo sapiens]	1.118	0.10358108
Rab GTPase-binding effector protein 2 [OS=Homo sapiens]	1.118	0.014409437
Signal transducing adapter molecule 1 [OS=Homo sapiens]	1.118	0.530143975
Exocyst complex component 1 [OS=Homo sapiens]	1.118	0.199151682
CCN family member 1 [OS=Homo sapiens]	1.118	0.927985712
Estradiol 17-beta-dehydrogenase 11 [OS=Homo sapiens]	1.118	0.417511986
Mannose-1-phosphate guanyltransferase beta [OS=Homo sapiens]	1.118	0.194533942
ADP-ribosylation factor-like protein 6-interacting protein 1 [OS=Homo sapiens]	1.118	0.560107213
Dynein light chain Tctex-type 1 [OS=Homo sapiens]	1.118	0.383846111
SAM and SH3 domain-containing protein 1 [OS=Homo sapiens]	1.118	0.596767412
Protein FAM25G [OS=Homo sapiens]	1.118	0.715022218
Coiled-coil domain-containing protein 89 [OS=Homo sapiens]	1.118	0.963187065
Myomesin-3 [OS=Homo sapiens]	1.118	0.290520814
Dynamin-2 [OS=Homo sapiens]	1.119	0.130148249
Coatomer subunit beta' [OS=Homo sapiens]	1.119	0.043835948
Proteasomal ubiquitin receptor ADRM1 [OS=Homo sapiens]	1.119	0.143353206
RNA-binding protein FXR2 [OS=Homo sapiens]	1.119	0.038151108
GTP-binding protein SAR1a [OS=Homo sapiens]	1.119	0.669664958
NFATC2-interacting protein [OS=Homo sapiens]	1.119	0.149416986
Pirin [OS=Homo sapiens]	1.119	0.060278779
Serine/threonine-protein kinase Nek3 [OS=Homo sapiens]	1.119	0.014440955
Striatin-4 [OS=Homo sapiens]	1.119	0.311030505
Kunitz-type protease inhibitor 2 [OS=Homo sapiens]	1.119	0.344689731
Ankyrin repeat and SAM domain-containing protein 1A [OS=Homo sapiens]	1.119	0.604772857
E3 ubiquitin-protein ligase synoviolin [OS=Homo sapiens]	1.119	0.651949106
tRNA (adenine(58)-N(1))-methyltransferase non-catalytic subunit TRM6 [OS=Ho	1.119	0.228068666
Anaphase-promoting complex subunit 2 [OS=Homo sapiens]	1.119	0.219868628
Transcription factor HES-1 [OS=Homo sapiens]	1.119	0.991845081
GRB10-interacting GYF protein 2 [OS=Homo sapiens]	1.12	0.230162679
Tyrosine-protein phosphatase non-receptor type 23 [OS=Homo sapiens]	1.12	0.047119911
Glutamine--fructose-6-phosphate aminotransferase [isomerizing] 2 [OS=Homo	1.12	0.112764637

Phosphoglucomutase-1 [OS=Homo sapiens]	1.12	0.38220716
Phosphatidylethanolamine-binding protein 1 [OS=Homo sapiens]	1.12	0.152429303
Ras-related protein Rab-14 [OS=Homo sapiens]	1.12	0.139942989
Septin-10 [OS=Homo sapiens]	1.12	0.208322137
Regulator of microtubule dynamics protein 1 [OS=Homo sapiens]	1.12	0.01300833
Argininosuccinate lyase [OS=Homo sapiens]	1.12	0.063644096
Proto-oncogene c-Rel [OS=Homo sapiens]	1.12	0.068487737
DnaJ homolog subfamily B member 2 [OS=Homo sapiens]	1.12	0.245099952
Mitochondrial mRNA pseudouridine synthase RPUSD3 [OS=Homo sapiens]	1.12	0.002053227
Forkhead box protein G1 [OS=Homo sapiens]	1.12	0.977919811
Methyltransferase N6AMT1 [OS=Homo sapiens]	1.12	0.932250874
cAMP-specific 3',5'-cyclic phosphodiesterase 4A [OS=Homo sapiens]	1.12	0.837306548
Fermitin family homolog 3 [OS=Homo sapiens]	1.12	0.999999997
ATP-dependent RNA helicase SUPV3L1, mitochondrial [OS=Homo sapiens]	1.121	0.149471864
AP-3 complex subunit mu-1 [OS=Homo sapiens]	1.121	0.047754668
WASH complex subunit 4 [OS=Homo sapiens]	1.121	0.05892463
Myosin light chain 6B [OS=Homo sapiens]	1.121	0.034185495
Synergin gamma [OS=Homo sapiens]	1.121	0.065110816
GPI transamidase component PIG-T [OS=Homo sapiens]	1.121	0.079789502
Calcium/calmodulin-dependent protein kinase type 1 [OS=Homo sapiens]	1.121	0.210078042
Protein arginine methyltransferase NDUFAF7, mitochondrial [OS=Homo sapiens	1.121	0.035866532
Peroxiredoxin-like 2A [OS=Homo sapiens]	1.121	0.139002915
Nicotinate-nucleotide pyrophosphorylase [carboxylating] [OS=Homo sapiens]	1.121	0.046079984
Breakpoint cluster region protein [OS=Homo sapiens]	1.121	0.439826246
Bifunctional polynucleotide phosphatase/kinase [OS=Homo sapiens]	1.121	0.57044822
Aldehyde dehydrogenase, mitochondrial [OS=Homo sapiens]	1.121	0.70182536
DNA-directed RNA polymerase III subunit RPC5 [OS=Homo sapiens]	1.121	0.42631952
Protein FRA10AC1 [OS=Homo sapiens]	1.121	0.992029466
Tetraspanin-15 [OS=Homo sapiens]	1.121	0.212675835
Calcium/calmodulin-dependent protein kinase type IV [OS=Homo sapiens]	1.121	0.987806092
Electron transfer flavoprotein subunit beta [OS=Homo sapiens]	1.122	0.00292906
Integrin-linked protein kinase [OS=Homo sapiens]	1.122	0.091509911
Zinc finger protein ZPR1 [OS=Homo sapiens]	1.122	0.145808369
Gamma-taxilin [OS=Homo sapiens]	1.122	0.127602159
RNA-binding protein RO60 [OS=Homo sapiens]	1.122	0.03456636
Endoplasmic reticulum lectin 1 [OS=Homo sapiens]	1.122	0.209781784
Serine/threonine-protein kinase tousled-like 1 [OS=Homo sapiens]	1.122	0.09857604
Methyltransferase-like 26 [OS=Homo sapiens]	1.122	0.200945817
Vitamin K epoxide reductase complex subunit 1-like protein 1 [OS=Homo sapien	1.122	0.964035202
Cerebellar degeneration-related protein 2-like [OS=Homo sapiens]	1.122	0.741827707
N-terminal EF-hand calcium-binding protein 3 [OS=Homo sapiens]	1.122	0.99635014
Transitional endoplasmic reticulum ATPase [OS=Homo sapiens]	1.123	0.21740593
Acylamino-acid-releasing enzyme [OS=Homo sapiens]	1.123	0.357207796
Proteasome subunit beta type-5 [OS=Homo sapiens]	1.123	0.765211291
Fumarate hydratase, mitochondrial [OS=Homo sapiens]	1.123	0.189789967
Intermembrane lipid transfer protein VPS13C [OS=Homo sapiens]	1.123	0.062262655
Kinesin-like protein KIF3A [OS=Homo sapiens]	1.123	0.052003758
Protein S100-A10 [OS=Homo sapiens]	1.123	0.745425676
N-terminal kinase-like protein [OS=Homo sapiens]	1.123	0.066032435
m-AAA protease-interacting protein 1, mitochondrial [OS=Homo sapiens]	1.123	0.901366562
WD repeat-containing protein 13 [OS=Homo sapiens]	1.123	0.540311172
DISP complex protein LRCH3 [OS=Homo sapiens]	1.123	0.544900154
Receptor tyrosine-protein kinase erbB-2 [OS=Homo sapiens]	1.123	0.4889537
Cysteine-rich motor neuron 1 protein [OS=Homo sapiens]	1.123	0.206848515
Peroxisomal membrane protein PEX16 [OS=Homo sapiens]	1.123	0.999999997
Zinc finger CCCH domain-containing protein 15 [OS=Homo sapiens]	1.124	0.20387044
Heat shock 70 kDa protein 13 [OS=Homo sapiens]	1.124	0.066663884
WD repeat-containing protein 91 [OS=Homo sapiens]	1.124	0.799552508
ADP-sugar pyrophosphatase [OS=Homo sapiens]	1.124	0.099327699
Inositol 1,4,5-trisphosphate receptor type 1 [OS=Homo sapiens]	1.124	0.988557651
Lysosome membrane protein 2 [OS=Homo sapiens]	1.124	0.687998314
Esterase OVCA2 [OS=Homo sapiens]	1.124	0.264907114
Cystatin-B [OS=Homo sapiens]	1.124	0.367811032
Adenosine 3'-phospho 5'-phosphosulfate transporter 1 [OS=Homo sapiens]	1.124	0.999936551
Protein FAM168A [OS=Homo sapiens]	1.124	0.992050705
Hexokinase-1 [OS=Homo sapiens]	1.125	0.006645755
Dynamin-1-like protein [OS=Homo sapiens]	1.125	0.39927255
CYFIP-related Rac1 interactor B [OS=Homo sapiens]	1.125	0.062204158
Saccharopine dehydrogenase-like oxidoreductase [OS=Homo sapiens]	1.125	0.008585099
Sodium bicarbonate cotransporter 3 [OS=Homo sapiens]	1.125	0.978320824
L-2-hydroxyglutarate dehydrogenase, mitochondrial [OS=Homo sapiens]	1.125	0.096659286
E3 ubiquitin-protein ligase MARCHF5 [OS=Homo sapiens]	1.125	0.999989546
Dynactin subunit 5 [OS=Homo sapiens]	1.125	0.244459067
Protein unc-13 homolog B [OS=Homo sapiens]	1.125	0.58419527
RNA guanine-N7 methyltransferase-activating subunit-like protein [OS=Homo sa	1.125	0.655300782

Ankyrin repeat domain-containing protein 34C [OS=Homo sapiens]	1.125	0.878502076
Kinesin-like protein KIF1B [OS=Homo sapiens]	1.126	0.012181141
Proteasome activator complex subunit 1 [OS=Homo sapiens]	1.126	0.028571309
3-hydroxyisobutyrate dehydrogenase, mitochondrial [OS=Homo sapiens]	1.126	0.060913778
Golgi phosphoprotein 3 [OS=Homo sapiens]	1.126	0.252893026
Trafficking protein particle complex subunit 3 [OS=Homo sapiens]	1.126	0.126089861
MICAL-like protein 1 [OS=Homo sapiens]	1.126	0.031206424
Biorientation of chromosomes in cell division protein 1 [OS=Homo sapiens]	1.126	0.18326204
Tyrosine-protein kinase receptor UFO [OS=Homo sapiens]	1.126	0.801698974
Neuronal calcium sensor 1 [OS=Homo sapiens]	1.126	0.007244939
Lysine-specific demethylase 4A [OS=Homo sapiens]	1.126	0.999995185
Tryptophan--tRNA ligase, mitochondrial [OS=Homo sapiens]	1.126	0.532949345
Retinoblastoma-like protein 1 [OS=Homo sapiens]	1.126	0.705457619
Armadillo repeat-containing protein 2 [OS=Homo sapiens]	1.126	0.345065306
Glucosidase 2 subunit beta [OS=Homo sapiens]	1.127	0.258440164
Thimet oligopeptidase [OS=Homo sapiens]	1.127	0.05089132
FAST kinase domain-containing protein 4 [OS=Homo sapiens]	1.127	0.336909059
Proteasome subunit alpha type-1 [OS=Homo sapiens]	1.127	0.470156982
Sec1 family domain-containing protein 1 [OS=Homo sapiens]	1.127	0.000693887
Ubiquitin-protein ligase E3C [OS=Homo sapiens]	1.127	0.198823396
Vesicle-associated membrane protein 7 [OS=Homo sapiens]	1.127	0.108201645
Peptidyl-tRNA hydrolase 2, mitochondrial [OS=Homo sapiens]	1.127	0.038647194
Protein odr-4 homolog [OS=Homo sapiens]	1.127	0.040069745
Tyrosyl-DNA phosphodiesterase 1 [OS=Homo sapiens]	1.127	0.116342667
Alpha- and gamma-adaptin-binding protein p34 [OS=Homo sapiens]	1.127	0.682278681
Pyruvate carboxylase, mitochondrial [OS=Homo sapiens]	1.127	0.154476533
Peroxiredoxin-1 [OS=Homo sapiens]	1.128	0.015152843
Dedicator of cytokinesis protein 6 [OS=Homo sapiens]	1.128	0.506849186
60S ribosomal protein L36a-like [OS=Homo sapiens]	1.128	0.609625471
RAB11-binding protein RELCH [OS=Homo sapiens]	1.128	0.063416037
Lysosomal Pro-X carboxypeptidase [OS=Homo sapiens]	1.128	0.174523688
Ribosome-releasing factor 2, mitochondrial [OS=Homo sapiens]	1.128	0.152952996
RUS family member 1 [OS=Homo sapiens]	1.128	0.355372729
NGFI-A-binding protein 2 [OS=Homo sapiens]	1.128	0.3709392
Arylsulfatase K [OS=Homo sapiens]	1.128	0.628322696
Glutaredoxin-1 [OS=Homo sapiens]	1.128	0.843801499
Ataxin-2 [OS=Homo sapiens]	1.129	0.028684423
VIP36-like protein [OS=Homo sapiens]	1.129	0.116636389
Valine--tRNA ligase, mitochondrial [OS=Homo sapiens]	1.129	0.053201029
Putative phospholipase B-like 2 [OS=Homo sapiens]	1.129	0.002301119
Membrane-associated progesterone receptor component 1 [OS=Homo sapiens]	1.129	0.98323338
Serine protease HTRA2, mitochondrial [OS=Homo sapiens]	1.129	0.190893218
Ethanolamine kinase 1 [OS=Homo sapiens]	1.129	0.03577606
SWI/SNF-related matrix-associated actin-dependent regulator of chromatin sub	1.129	0.999299926
Small integral membrane protein 12 [OS=Homo sapiens]	1.129	0.986395794
Very low-density lipoprotein receptor [OS=Homo sapiens]	1.129	0.507920757
Coiled-coil domain-containing protein 85B [OS=Homo sapiens]	1.129	0.728317393
Solute carrier family 35 member E3 [OS=Homo sapiens]	1.129	0.998966634
CREB-regulated transcription coactivator 2 [OS=Homo sapiens]	1.129	0.184031626
Ribosome-binding protein 1 [OS=Homo sapiens]	1.13	0.100649069
Rab GDP dissociation inhibitor beta [OS=Homo sapiens]	1.13	0.33067714
Leucine-rich repeat-containing protein 40 [OS=Homo sapiens]	1.13	0.180753391
Dynactin subunit 2 [OS=Homo sapiens]	1.13	0.11782919
Aflatoxin B1 aldehyde reductase member 2 [OS=Homo sapiens]	1.13	0.11988623
SRSF protein kinase 2 [OS=Homo sapiens]	1.13	0.006897908
TBC1 domain family member 13 [OS=Homo sapiens]	1.13	0.256028715
Poliovirus receptor [OS=Homo sapiens]	1.13	0.452597315
Cyclin-dependent kinase 16 [OS=Homo sapiens]	1.13	0.168206182
Far upstream element-binding protein 2 [OS=Homo sapiens]	1.131	0.010207519
Carbonyl reductase [NADPH] 1 [OS=Homo sapiens]	1.131	0.089360667
S-phase kinase-associated protein 1 [OS=Homo sapiens]	1.131	0.09981935
EH domain-containing protein 3 [OS=Homo sapiens]	1.131	0.222627063
Oxidoreductase HTATIP2 [OS=Homo sapiens]	1.131	0.040703247
Uridine phosphorylase 1 [OS=Homo sapiens]	1.131	0.177418119
NudC domain-containing protein 2 [OS=Homo sapiens]	1.131	0.327096716
CBY1-interacting BAR domain-containing protein 1 [OS=Homo sapiens]	1.131	0.994267319
High mobility group protein HMGI-C [OS=Homo sapiens]	1.131	0.998516091
Guanylate kinase [OS=Homo sapiens]	1.131	0.504369865
Ferritin heavy chain [OS=Homo sapiens]	1.131	0.814589314
Reticulon-3 [OS=Homo sapiens]	1.131	0.383427678
Caspase-6 [OS=Homo sapiens]	1.131	0.852439245
Secretory carrier-associated membrane protein 4 [OS=Homo sapiens]	1.131	0.916252938
Succinate dehydrogenase assembly factor 3, mitochondrial [OS=Homo sapiens]	1.131	0.601524424
Cell growth regulator with EF hand domain protein 1 [OS=Homo sapiens]	1.131	0.572460691
Catenin delta-1 [OS=Homo sapiens]	1.132	0.000188821

BOS complex subunit NOMO2 [OS=Homo sapiens]	1.132	0.110749407
Ras-related protein Rab-7a [OS=Homo sapiens]	1.132	0.029279882
Deoxynucleoside triphosphate triphosphohydrolase SAMHD1 [OS=Homo sapier	1.132	0.062966907
Echinoderm microtubule-associated protein-like 3 [OS=Homo sapiens]	1.132	0.369571415
COMM domain-containing protein 3 [OS=Homo sapiens]	1.132	0.045831034
Filamin-binding LIM protein 1 [OS=Homo sapiens]	1.132	0.158955449
Annexin A3 [OS=Homo sapiens]	1.132	0.897767222
Thiamine-triphosphatase [OS=Homo sapiens]	1.132	0.251558701
Ankyrin repeat and SOCS box protein 6 [OS=Homo sapiens]	1.132	0.998372805
Glucose-6-phosphate 1-dehydrogenase [OS=Homo sapiens]	1.133	0.511464235
Dipeptidyl peptidase 3 [OS=Homo sapiens]	1.133	0.313559146
Glutathione S-transferase P [OS=Homo sapiens]	1.133	0.283427455
Receptor-interacting serine/threonine-protein kinase 1 [OS=Homo sapiens]	1.133	0.086215108
Low molecular weight phosphotyrosine protein phosphatase [OS=Homo sapiens	1.133	0.114742325
Serine/threonine-protein phosphatase 2A 56 kDa regulatory subunit epsilon isof	1.133	0.024068982
Protein canopy homolog 3 [OS=Homo sapiens]	1.133	0.058726277
Glutamine and serine-rich protein 1 [OS=Homo sapiens]	1.133	0.604031746
OTU domain-containing protein 7B [OS=Homo sapiens]	1.133	0.242297647
E3 ubiquitin-protein ligase Itchy homolog [OS=Homo sapiens]	1.134	0.028581786
Aminoacyl tRNA synthase complex-interacting multifunctional protein 2 [OS=Hc	1.134	0.713004212
Ras suppressor protein 1 [OS=Homo sapiens]	1.134	0.223765532
Tyrosine-protein kinase CSK [OS=Homo sapiens]	1.134	0.006313759
Ubiquitin-conjugating enzyme E2 D2 [OS=Homo sapiens]	1.134	0.071784008
WW domain-containing adapter protein with coiled-coil [OS=Homo sapiens]	1.134	0.050412423
Liprin-beta-2 [OS=Homo sapiens]	1.134	0.439329296
BLOC-2 complex member HPS6 [OS=Homo sapiens]	1.134	0.997808112
G antigen 12B/C/D/E [OS=Homo sapiens]	1.134	0.325232041
Isocitrate dehydrogenase [NADP] cytoplasmic [OS=Homo sapiens]	1.135	0.076697588
Sterol carrier protein 2 [OS=Homo sapiens]	1.135	0.124564568
Patatin-like phospholipase domain-containing protein 6 [OS=Homo sapiens]	1.135	0.013931779
TATA-binding protein-associated factor 172 [OS=Homo sapiens]	1.135	0.016856111
Alpha-centractin [OS=Homo sapiens]	1.135	0.158948369
Phosphatidylinositol 4-phosphate 3-kinase C2 domain-containing subunit alpha	1.135	0.04618624
Brefeldin A-inhibited guanine nucleotide-exchange protein 2 [OS=Homo sapiens	1.135	0.073978436
Protein phosphatase 1 regulatory subunit 14B [OS=Homo sapiens]	1.135	0.606477766
F-box only protein 2 [OS=Homo sapiens]	1.135	0.038331938
Acetoacetyl-CoA synthetase [OS=Homo sapiens]	1.135	0.069436625
Importin-13 [OS=Homo sapiens]	1.135	0.106046046
BRISC complex subunit Abraxas 2 [OS=Homo sapiens]	1.135	0.072923051
Ubiquitin carboxyl-terminal hydrolase isozyme L3 [OS=Homo sapiens]	1.135	0.246178898
Inositol-trisphosphate 3-kinase C [OS=Homo sapiens]	1.135	0.309651834
Chloride intracellular channel protein 1 [OS=Homo sapiens]	1.136	0.241120932
Tumor protein D52 [OS=Homo sapiens]	1.136	0.017268509
WD repeat domain-containing protein 83 [OS=Homo sapiens]	1.136	0.821637418
Autophagy-related protein 16-1 [OS=Homo sapiens]	1.136	0.011036975
Tubulin polyglutamylase complex subunit 2 [OS=Homo sapiens]	1.136	0.909138692
F-box/WD repeat-containing protein 11 [OS=Homo sapiens]	1.136	0.430315166
Low-density lipoprotein receptor-related protein 11 [OS=Homo sapiens]	1.136	0.876796351
Protein Niban 2 [OS=Homo sapiens]	1.137	0.052490716
Proteasome subunit beta type-4 [OS=Homo sapiens]	1.137	0.30660333
Proteasome subunit alpha type-4 [OS=Homo sapiens]	1.137	0.803801583
BAG family molecular chaperone regulator 2 [OS=Homo sapiens]	1.137	0.006103732
Intersectin-2 [OS=Homo sapiens]	1.137	0.040029736
Programmed cell death protein 4 [OS=Homo sapiens]	1.137	0.195354812
Peptidyl-prolyl cis-trans isomerase FKBP11 [OS=Homo sapiens]	1.137	0.302324292
Target of rapamycin complex subunit LST8 [OS=Homo sapiens]	1.137	0.395341097
COMM domain-containing protein 8 [OS=Homo sapiens]	1.137	0.637008578
Dolichol-phosphate mannosyltransferase subunit 3 [OS=Homo sapiens]	1.137	0.111701164
Calpain-5 [OS=Homo sapiens]	1.137	0.998116025
Zinc finger protein 804A [OS=Homo sapiens]	1.137	0.859622033
Protein O-GlcNAcase [OS=Homo sapiens]	1.138	0.284889404
Chromodomain-helicase-DNA-binding protein 8 [OS=Homo sapiens]	1.138	0.023485193
Medium-chain specific acyl-CoA dehydrogenase, mitochondrial [OS=Homo sapi	1.138	0.040745425
Serine/threonine-protein phosphatase 4 regulatory subunit 3B [OS=Homo sapie	1.138	0.008074468
ADP-ribosylhydrolase ARH3 [OS=Homo sapiens]	1.138	0.123882795
Myotubularin [OS=Homo sapiens]	1.138	0.15011558
Cytoplasmic protein NCK2 [OS=Homo sapiens]	1.138	0.146515452
Probable E3 ubiquitin-protein ligase HECTD4 [OS=Homo sapiens]	1.138	0.676251449
Cerebellar degeneration-related protein 2 [OS=Homo sapiens]	1.138	0.969085284
Plasma protease C1 inhibitor [OS=Homo sapiens]	1.138	0.188744441
Cytosolic non-specific dipeptidase [OS=Homo sapiens]	1.139	0.333080347
NEDD8-activating enzyme E1 regulatory subunit [OS=Homo sapiens]	1.139	0.02463457
Ras-related protein Rab-11B [OS=Homo sapiens]	1.139	0.043177972
Protein NipSnap homolog 1 [OS=Homo sapiens]	1.139	0.643519024
Striatin-3 [OS=Homo sapiens]	1.139	0.019408865

E3 ubiquitin-protein ligase NEDD4-like [OS=Homo sapiens]	1.139	0.362378819
Mannose-1-phosphate guanyltransferase alpha [OS=Homo sapiens]	1.139	0.100412994
Ras-related protein Rab-3D [OS=Homo sapiens]	1.139	0.29814637
LIM and senescent cell antigen-like-containing domain protein 1 [OS=Homo sap	1.139	0.228847352
Complement factor B [OS=Homo sapiens]	1.139	0.999910498
U6 snRNA-associated Sm-like protein LSm1 [OS=Homo sapiens]	1.139	0.084610714
Tubulin beta-2A chain [OS=Homo sapiens]	1.14	0.188491563
Exportin-5 [OS=Homo sapiens]	1.14	0.029685112
Beta-arrestin-1 [OS=Homo sapiens]	1.14	0.085236398
[3-methyl-2-oxobutanoate dehydrogenase [lipoamide]] kinase, mitochondrial [C	1.14	0.215197842
Calcium/calmodulin-dependent protein kinase kinase 2 [OS=Homo sapiens]	1.14	0.812096611
BAG family molecular chaperone regulator 4 [OS=Homo sapiens]	1.14	0.073182086
Phosphatidylinositol glycan anchor biosynthesis class U protein [OS=Homo sapi	1.14	0.90713917
Eukaryotic translation initiation factor 2-alpha kinase 3 [OS=Homo sapiens]	1.14	0.093966394
Mediator of RNA polymerase II transcription subunit 28 [OS=Homo sapiens]	1.14	0.444011281
Phosphatidylinositol 3,4,5-trisphosphate 3-phosphatase and dual-specificity pr	1.14	0.122281888
Coiled-coil domain-containing protein 115 [OS=Homo sapiens]	1.14	0.151118632
Adenylyl cyclase-associated protein 1 [OS=Homo sapiens]	1.141	0.012684398
Baculoviral IAP repeat-containing protein 6 [OS=Homo sapiens]	1.141	0.263921801
Electron transfer flavoprotein subunit alpha, mitochondrial [OS=Homo sapiens]	1.141	0.355934786
Proteasome subunit alpha type-7 [OS=Homo sapiens]	1.141	0.829957462
E3 ubiquitin-protein ligase TRIM56 [OS=Homo sapiens]	1.141	0.031714017
Transmembrane 9 superfamily member 2 [OS=Homo sapiens]	1.141	0.999735233
Isocitrate dehydrogenase [NAD] subunit beta, mitochondrial [OS=Homo sapiens	1.141	0.047782739
Surfeit locus protein 4 [OS=Homo sapiens]	1.141	0.666261857
Ras-related protein Rab-18 [OS=Homo sapiens]	1.141	0.002718062
Holocytochrome c-type synthase [OS=Homo sapiens]	1.141	0.989850349
Apoptosis regulator BAX [OS=Homo sapiens]	1.141	0.131413329
CCR4-NOT transcription complex subunit 7 [OS=Homo sapiens]	1.141	0.122866199
Ras-related GTP-binding protein A [OS=Homo sapiens]	1.141	0.266515732
E3 ubiquitin-protein ligase SHPRH [OS=Homo sapiens]	1.141	0.007366312
rRNA methyltransferase 1, mitochondrial [OS=Homo sapiens]	1.141	0.005866649
Neogenin [OS=Homo sapiens]	1.141	0.749019204
Pleckstrin homology domain-containing family M member 2 [OS=Homo sapiens]	1.141	0.42488768
Protein DGCR6 [OS=Homo sapiens]	1.141	0.052982575
Mitochondrial dynamics protein MID51 [OS=Homo sapiens]	1.141	0.937210568
PMS1 protein homolog 1 [OS=Homo sapiens]	1.141	0.767716083
Metalloproteinase inhibitor 2 [OS=Homo sapiens]	1.141	0.869296007
Calreticulin [OS=Homo sapiens]	1.142	0.025906981
Prolyl endopeptidase [OS=Homo sapiens]	1.142	0.007972949
Serine/threonine-protein phosphatase 2A 56 kDa regulatory subunit delta isofor	1.142	0.010058764
Carnitine O-acetyltransferase [OS=Homo sapiens]	1.142	0.037619715
Proteasome subunit alpha type-2 [OS=Homo sapiens]	1.142	0.627379704
Cytoplasmic dynein 1 light intermediate chain 2 [OS=Homo sapiens]	1.142	0.01861394
Aspartate--tRNA ligase, mitochondrial [OS=Homo sapiens]	1.142	0.43021394
Putative ATP-dependent RNA helicase DHX57 [OS=Homo sapiens]	1.142	0.054839842
Dolichyl-diphosphooligosaccharide--protein glycosyltransferase 48 kDa subunit	1.142	0.665145557
Intraflagellar transport protein 27 homolog [OS=Homo sapiens]	1.142	0.03314923
Phosphatidylinositol 4-kinase type 2-alpha [OS=Homo sapiens]	1.142	0.053215073
Axin interactor, dorsalization-associated protein [OS=Homo sapiens]	1.142	0.033066474
Clathrin light chain A [OS=Homo sapiens]	1.142	0.066286444
Bromodomain-containing protein 3 [OS=Homo sapiens]	1.142	0.141263646
1-phosphatidylinositol 3-phosphate 5-kinase [OS=Homo sapiens]	1.142	0.492271394
Sacsin [OS=Homo sapiens]	1.142	0.953926357
UV radiation resistance-associated gene protein [OS=Homo sapiens]	1.142	0.681498184
SPRY domain-containing protein 7 [OS=Homo sapiens]	1.142	0.971924915
Non-receptor tyrosine-protein kinase TYK2 [OS=Homo sapiens]	1.142	0.024057688
Protein preY, mitochondrial [OS=Homo sapiens]	1.142	0.860556607
Neuferricin [OS=Homo sapiens]	1.142	0.444838452
WASH complex subunit 2A [OS=Homo sapiens]	1.143	0.017655357
Syntaxin-binding protein 2 [OS=Homo sapiens]	1.143	0.003388388
NEDD8-activating enzyme E1 catalytic subunit [OS=Homo sapiens]	1.143	0.291003367
Nucleoside diphosphate kinase B [OS=Homo sapiens]	1.143	0.216443293
Ras-related protein Rab-2A [OS=Homo sapiens]	1.143	0.330500147
Peptidyl-prolyl cis-trans isomerase-like 1 [OS=Homo sapiens]	1.143	0.001616848
Alpha-galactosidase A [OS=Homo sapiens]	1.143	0.040262297
Serine/threonine-protein phosphatase 4 regulatory subunit 3A [OS=Homo sapie	1.143	0.020518671
Lipoamide acyltransferase component of branched-chain alpha-keto acid dehyc	1.143	0.064418104
Conserved oligomeric Golgi complex subunit 8 [OS=Homo sapiens]	1.143	0.938609915
Phospholipase D2 [OS=Homo sapiens]	1.143	0.450025003
YY1-associated factor 2 [OS=Homo sapiens]	1.143	0.99739797
ATP synthase subunit a [OS=Homo sapiens]	1.143	0.999999764
Feline leukemia virus subgroup C receptor-related protein 1 [OS=Homo sapiens]	1.143	0.996537398
C-Jun-amino-terminal kinase-interacting protein 4 [OS=Homo sapiens]	1.144	0.020443485
Guanine nucleotide-binding protein-like 1 [OS=Homo sapiens]	1.144	0.255658793

ATP-dependent Clp protease proteolytic subunit, mitochondrial [OS=Homo sapi	1.144	0.131124181
AP-1 complex subunit gamma-like 2 [OS=Homo sapiens]	1.144	0.614205779
Histone-lysine N-methyltransferase 2C [OS=Homo sapiens]	1.144	0.999966325
[Pyruvate dehydrogenase [acetyl-transferring]]-phosphatase 1, mitochondrial [C	1.145	0.023385102
Phosphomannomutase 2 [OS=Homo sapiens]	1.145	0.040028875
Protein arginine N-methyltransferase 7 [OS=Homo sapiens]	1.145	0.733425762
FAST kinase domain-containing protein 5, mitochondrial [OS=Homo sapiens]	1.145	0.875773451
WD repeat domain phosphoinositide-interacting protein 4 [OS=Homo sapiens]	1.145	0.027865691
Ubiquitin carboxyl-terminal hydrolase MINDY-3 [OS=Homo sapiens]	1.145	0.788940617
ETS domain-containing protein Elk-1 [OS=Homo sapiens]	1.145	0.998991786
[Pyruvate dehydrogenase (acetyl-transferring)] kinase isozyme 1, mitochondrial	1.145	0.90576849
Epsin-1 [OS=Homo sapiens]	1.145	0.566747005
UPF0690 protein C1orf52 [OS=Homo sapiens]	1.145	0.25521936
Proteasome subunit alpha type-3 [OS=Homo sapiens]	1.146	0.278004009
Cysteine desulfurase [OS=Homo sapiens]	1.146	0.014663346
Septin-6 [OS=Homo sapiens]	1.146	0.081986637
Integrin alpha-3 [OS=Homo sapiens]	1.146	0.157535415
Transmembrane emp24 domain-containing protein 10 [OS=Homo sapiens]	1.146	0.788674106
Microtubule-associated protein 1S [OS=Homo sapiens]	1.146	0.11984321
Dolichyl-diphosphooligosaccharide--protein glycosyltransferase subunit STT3A	1.146	0.999756981
Tetratricopeptide repeat protein 38 [OS=Homo sapiens]	1.146	0.308888883
Peptidyl-prolyl cis-trans isomerase FKBP8 [OS=Homo sapiens]	1.146	0.404073149
Protein OS-9 [OS=Homo sapiens]	1.146	0.085987453
Mitochondrial import inner membrane translocase subunit Tim13 [OS=Homo sa	1.146	0.280681047
Katanin p80 WD40 repeat-containing subunit B1 [OS=Homo sapiens]	1.146	0.198705282
E3 ubiquitin-protein ligase HECTD3 [OS=Homo sapiens]	1.146	0.041040334
Protein unc-119 homolog B [OS=Homo sapiens]	1.146	0.984015712
E3 ubiquitin-protein ligase RNF213 [OS=Homo sapiens]	1.147	0.062350637
UDP-N-acetylglucosamine--peptide N-acetylglucosaminyltransferase 110 kDa s	1.147	0.019163265
Serine/threonine-protein kinase MRCK beta [OS=Homo sapiens]	1.147	0.004282782
NSFL1 cofactor p47 [OS=Homo sapiens]	1.147	0.075495402
Lipid scramblase CLPTM1L [OS=Homo sapiens]	1.147	0.14862967
Sorting nexin-1 [OS=Homo sapiens]	1.147	0.010572698
6-phosphofructo-2-kinase/fructose-2,6-bisphosphatase 2 [OS=Homo sapiens]	1.147	0.046536585
ARF GTPase-activating protein GIT1 [OS=Homo sapiens]	1.147	0.197202136
Rho GTPase-activating protein 17 [OS=Homo sapiens]	1.147	0.183653274
Sodium/potassium-transporting ATPase subunit beta-3 [OS=Homo sapiens]	1.147	0.96060153
Synaptobrevin homolog YKT6 [OS=Homo sapiens]	1.147	0.01274836
Cytoplasmic dynein 2 heavy chain 1 [OS=Homo sapiens]	1.147	0.150136741
AP-1 complex subunit sigma-1A [OS=Homo sapiens]	1.147	0.095507745
D-glutamate cyclase, mitochondrial [OS=Homo sapiens]	1.147	0.028779615
Dynactin subunit 3 [OS=Homo sapiens]	1.147	0.042137516
Ubiquitin-conjugating enzyme E2 E2 [OS=Homo sapiens]	1.147	0.554828401
Protein NipSnap homolog 3A [OS=Homo sapiens]	1.147	0.625805341
Dynein light chain Tctex-type 3 [OS=Homo sapiens]	1.147	0.599536285
Replication protein A 70 kDa DNA-binding subunit [OS=Homo sapiens]	1.148	0.066764556
Aldehyde dehydrogenase family 3 member A2 [OS=Homo sapiens]	1.148	0.092762409
Coxsackievirus and adenovirus receptor [OS=Homo sapiens]	1.148	0.959542234
Short/branched chain specific acyl-CoA dehydrogenase, mitochondrial [OS=Hor	1.148	0.130449127
Toll-interacting protein [OS=Homo sapiens]	1.148	0.023855608
Thymosin beta-4 [OS=Homo sapiens]	1.148	0.151672719
Vesicle-associated membrane protein 2 [OS=Homo sapiens]	1.148	0.99996475
Ataxin-3 [OS=Homo sapiens]	1.148	0.396854003
PITH domain-containing protein 1 [OS=Homo sapiens]	1.148	0.242080233
Thiopurine S-methyltransferase [OS=Homo sapiens]	1.148	0.067540595
Heat shock factor protein 2 [OS=Homo sapiens]	1.148	0.476447805
Protein GPR107 [OS=Homo sapiens]	1.148	0.998240022
Betaine--homocysteine S-methyltransferase 1 [OS=Homo sapiens]	1.148	0.748954983
Insulin receptor substrate 1 [OS=Homo sapiens]	1.148	0.994125539
Eukaryotic translation initiation factor 4 gamma 1 [OS=Homo sapiens]	1.149	0.058288625
Cytochrome c [OS=Homo sapiens]	1.149	0.998532163
Tubulin-specific chaperone E [OS=Homo sapiens]	1.149	0.136468976
Interferon-induced, double-stranded RNA-activated protein kinase [OS=Homo s	1.149	0.004417114
Maspardin [OS=Homo sapiens]	1.149	0.189728934
Transmembrane 9 superfamily member 3 [OS=Homo sapiens]	1.149	0.999935448
Methylmalonyl-CoA mutase, mitochondrial [OS=Homo sapiens]	1.149	0.021717538
Protein MEMO1 [OS=Homo sapiens]	1.149	0.680142436
BLOC-1-related complex subunit 6 [OS=Homo sapiens]	1.149	0.052597939
Osteoclast-stimulating factor 1 [OS=Homo sapiens]	1.149	0.822708377
Actin-related protein 3B [OS=Homo sapiens]	1.149	0.956584485
Mediator of RNA polymerase II transcription subunit 13 [OS=Homo sapiens]	1.149	0.707801265
Annexin A4 [OS=Homo sapiens]	1.15	0.011021115
Multifunctional procollagen lysine hydroxylase and glycosyltransferase LH3 [OS	1.15	0.001762689
Pyruvate dehydrogenase E1 component subunit alpha, somatic form, mitochond	1.15	0.140427221
Mitochondrial ribonuclease P catalytic subunit [OS=Homo sapiens]	1.15	0.421010062

UBX domain-containing protein 1 [OS=Homo sapiens]	1.15	0.173538655
Transmembrane 9 superfamily member 4 [OS=Homo sapiens]	1.15	0.999836719
Paraneoplastic antigen Ma2 [OS=Homo sapiens]	1.15	0.003970439
StAR-related lipid transfer protein 7, mitochondrial [OS=Homo sapiens]	1.15	0.198387674
Eukaryotic translation initiation factor 4E type 2 [OS=Homo sapiens]	1.15	0.021873315
Inhibitor of nuclear factor kappa-B kinase subunit alpha [OS=Homo sapiens]	1.15	0.373935646
UPF0598 protein C8orf82 [OS=Homo sapiens]	1.15	0.033892598
Inter-alpha-trypsin inhibitor heavy chain H3 [OS=Homo sapiens]	1.15	0.87082264
MOB-like protein phocein [OS=Homo sapiens]	1.15	0.125228891
Cytochrome c oxidase assembly protein COX19 [OS=Homo sapiens]	1.15	0.348188219
Elongation factor Ts, mitochondrial [OS=Homo sapiens]	1.151	0.001944649
3-hydroxyacyl-CoA dehydrogenase type-2 [OS=Homo sapiens]	1.151	0.318663996
Crk-like protein [OS=Homo sapiens]	1.151	0.012133334
Acidic leucine-rich nuclear phosphoprotein 32 family member A [OS=Homo sap	1.151	0.33957927
High mobility group protein B2 [OS=Homo sapiens]	1.151	0.073192949
Translocation protein SEC63 homolog [OS=Homo sapiens]	1.151	0.931478558
Charged multivesicular body protein 2b [OS=Homo sapiens]	1.151	0.006495073
Male-specific lethal 1 homolog [OS=Homo sapiens]	1.151	0.112097156
Protein LSM12 [OS=Homo sapiens]	1.151	0.029641331
EARP and GARP complex-interacting protein 1 [OS=Homo sapiens]	1.151	0.042206677
E3 ubiquitin-protein ligase RNF170 [OS=Homo sapiens]	1.151	0.997009072
Dynactin subunit 6 [OS=Homo sapiens]	1.151	0.67639602
Mitogen-activated protein kinase kinase kinase kinase 5 [OS=Homo sapiens]	1.151	0.239320298
AP-2 complex subunit alpha-1 [OS=Homo sapiens]	1.152	0.000279063
Peroxisomal multifunctional enzyme type 2 [OS=Homo sapiens]	1.152	0.079657102
Leukotriene A-4 hydrolase [OS=Homo sapiens]	1.152	0.015421173
Intercellular adhesion molecule 1 [OS=Homo sapiens]	1.152	0.102699133
WD repeat-containing protein 11 [OS=Homo sapiens]	1.152	0.574680005
Ganglioside-induced differentiation-associated protein 1 [OS=Homo sapiens]	1.152	0.852105943
Protein misato homolog 1 [OS=Homo sapiens]	1.152	0.519655089
Mitochondrial fission 1 protein [OS=Homo sapiens]	1.152	0.007079605
Serine/threonine-protein kinase 38-like [OS=Homo sapiens]	1.152	0.238203111
Gem-associated protein 6 [OS=Homo sapiens]	1.152	0.121269574
Cysteine-rich and transmembrane domain-containing protein 1 [OS=Homo sapi	1.152	0.999775752
Heat shock protein 75 kDa, mitochondrial [OS=Homo sapiens]	1.153	0.056818417
Ribonuclease inhibitor [OS=Homo sapiens]	1.153	0.218242644
Sorting nexin-2 [OS=Homo sapiens]	1.153	0.099947669
ER membrane protein complex subunit 1 [OS=Homo sapiens]	1.153	0.357673596
Growth factor receptor-bound protein 2 [OS=Homo sapiens]	1.153	0.06916715
Conserved oligomeric Golgi complex subunit 1 [OS=Homo sapiens]	1.153	0.056079539
NPC intracellular cholesterol transporter 1 [OS=Homo sapiens]	1.153	0.08476256
Coiled-coil domain-containing protein 43 [OS=Homo sapiens]	1.153	0.13575187
TGF-beta-activated kinase 1 and MAP3K7-binding protein 1 [OS=Homo sapiens]	1.153	0.055150813
E3 SUMO-protein ligase PIAS2 [OS=Homo sapiens]	1.153	0.995923438
UPF0669 protein C6orf120 [OS=Homo sapiens]	1.153	0.588292531
Cytoplasmic phosphatidylinositol transfer protein 1 [OS=Homo sapiens]	1.153	0.632018089
Alpha-ketoglutarate-dependent dioxygenase alkB homolog 6 [OS=Homo sapiens	1.153	0.31078638
Ataxin-2-like protein [OS=Homo sapiens]	1.154	0.005219039
Melanoma-associated antigen 4 [OS=Homo sapiens]	1.154	0.135552937
Haloacid dehalogenase-like hydrolase domain-containing 5 [OS=Homo sapiens]	1.154	0.057671005
Dolichyl-diphosphooligosaccharide--protein glycosyltransferase subunit STT3B	1.154	0.998629211
Glycogen [starch] synthase, muscle [OS=Homo sapiens]	1.154	0.02795955
Sorbitol dehydrogenase [OS=Homo sapiens]	1.154	0.12475068
TBC1 domain family member 4 [OS=Homo sapiens]	1.154	0.017951554
Protein disulfide isomerase CRELD2 [OS=Homo sapiens]	1.154	0.036736456
Shiftless antiviral inhibitor of ribosomal frameshifting protein [OS=Homo sapiens	1.154	0.478740523
PRA1 family protein 2 [OS=Homo sapiens]	1.154	0.018778379
Leucine-rich repeat protein SHOC-2 [OS=Homo sapiens]	1.154	0.76687106
Annexin A6 [OS=Homo sapiens]	1.155	0.030854212
Filamin-C [OS=Homo sapiens]	1.155	0.006556396
Glutathione S-transferase Mu 3 [OS=Homo sapiens]	1.155	0.051885461
RNA-binding protein EWS [OS=Homo sapiens]	1.155	0.035406755
Succinate--CoA ligase [ADP-forming] subunit beta, mitochondrial [OS=Homo sa	1.155	0.045974626
YTH domain-containing family protein 3 [OS=Homo sapiens]	1.155	0.003132681
2-oxoisovalerate dehydrogenase subunit alpha, mitochondrial [OS=Homo sapie	1.155	0.336866104
Immunity-related GTPase family Q protein [OS=Homo sapiens]	1.155	0.002022717
Ras-related protein Rab-9A [OS=Homo sapiens]	1.155	0.004260472
Zinc finger protein 407 [OS=Homo sapiens]	1.155	0.989723477
AP-4 complex subunit mu-1 [OS=Homo sapiens]	1.155	0.656370409
Probable ubiquitin carboxyl-terminal hydrolase FAF-X [OS=Homo sapiens]	1.156	0.026870407
Coatomer subunit delta [OS=Homo sapiens]	1.156	0.009254382
Fructose-bisphosphate aldolase C [OS=Homo sapiens]	1.156	0.025353495
Fibronectin type III domain-containing protein 3B [OS=Homo sapiens]	1.156	0.067183883
Ras GTPase-activating protein 1 [OS=Homo sapiens]	1.156	0.005162896
GPI transamidase component PIG-S [OS=Homo sapiens]	1.156	0.425196085

Multiple inositol polyphosphate phosphatase 1 [OS=Homo sapiens]	1.156	0.254483763
B-cell CLL/lymphoma 9-like protein [OS=Homo sapiens]	1.156	0.08989998
Basal cell adhesion molecule [OS=Homo sapiens]	1.156	0.50332074
Arylsulfatase A [OS=Homo sapiens]	1.156	0.265994521
DnaJ homolog subfamily B member 12 [OS=Homo sapiens]	1.156	0.12703656
Echinoderm microtubule-associated protein-like 2 [OS=Homo sapiens]	1.156	0.021009653
COP9 signalosome complex subunit 7a [OS=Homo sapiens]	1.156	0.044635793
Carnosine N-methyltransferase [OS=Homo sapiens]	1.156	0.056830202
Choline/ethanolaminephosphotransferase 1 [OS=Homo sapiens]	1.156	0.917072781
Voltage-gated potassium channel subunit beta-2 [OS=Homo sapiens]	1.156	0.941889465
Coatomer subunit beta [OS=Homo sapiens]	1.157	0.032973577
Phosphoribosylformylglycinamidine synthase [OS=Homo sapiens]	1.157	0.025820126
General vesicular transport factor p115 [OS=Homo sapiens]	1.157	0.007772736
Insulin-degrading enzyme [OS=Homo sapiens]	1.157	0.005336417
Syntaxin-7 [OS=Homo sapiens]	1.157	0.032199093
Receptor-type tyrosine-protein phosphatase F [OS=Homo sapiens]	1.157	0.988676657
Protein transport protein Sec61 subunit alpha isoform 1 [OS=Homo sapiens]	1.157	0.396981681
Vacuolar protein sorting-associated protein 18 homolog [OS=Homo sapiens]	1.157	0.220066487
SHC-transforming protein 1 [OS=Homo sapiens]	1.157	0.02124554
Armadillo repeat-containing protein 8 [OS=Homo sapiens]	1.157	0.017281518
Syntaxin-12 [OS=Homo sapiens]	1.157	0.051295336
Nucleotidyltransferase MB21D2 [OS=Homo sapiens]	1.157	0.073800085
Apoptosis-associated speck-like protein containing a CARD [OS=Homo sapiens]	1.157	0.873719198
5'-AMP-activated protein kinase subunit gamma-2 [OS=Homo sapiens]	1.157	0.930920846
E3 ubiquitin-protein ligase UBR3 [OS=Homo sapiens]	1.157	0.74740856
Exocyst complex component 6 [OS=Homo sapiens]	1.157	0.033006187
Trafficking protein particle complex subunit 2 [OS=Homo sapiens]	1.157	0.845395757
Melanoma inhibitory activity protein 2 [OS=Homo sapiens]	1.158	0.074939621
ADP-ribosylation factor GTPase-activating protein 1 [OS=Homo sapiens]	1.158	0.100476245
Ras-related protein Rab-6A [OS=Homo sapiens]	1.158	0.17426511
Replication protein A 14 kDa subunit [OS=Homo sapiens]	1.158	0.579257671
SCY1-like protein 2 [OS=Homo sapiens]	1.158	0.014312893
Ubiquitin-like modifier-activating enzyme ATG7 [OS=Homo sapiens]	1.158	0.051993295
Periostin [OS=Homo sapiens]	1.158	0.780847428
Hornerin [OS=Homo sapiens]	1.158	1
Inactive ubiquitin carboxyl-terminal hydrolase 54 [OS=Homo sapiens]	1.158	0.996982265
Cytosolic acyl coenzyme A thioester hydrolase [OS=Homo sapiens]	1.159	0.00988147
Aminoacylase-1 [OS=Homo sapiens]	1.159	0.128049344
Omega-amidase NIT2 [OS=Homo sapiens]	1.159	0.039512459
Chitobiosyldiphosphodolichol beta-mannosyltransferase [OS=Homo sapiens]	1.159	0.02161425
Glycerol-3-phosphate dehydrogenase 1-like protein [OS=Homo sapiens]	1.159	0.059044535
Vitamin K epoxide reductase complex subunit 1 [OS=Homo sapiens]	1.159	0.41935417
Guanine nucleotide-binding protein subunit beta-like protein 1 [OS=Homo sapie	1.159	0.18204636
ADP-ribosylation factor-binding protein GGA2 [OS=Homo sapiens]	1.159	0.198821331
Adenomatous polyposis coli protein [OS=Homo sapiens]	1.159	0.3284814
Putative bifunctional UDP-N-acetylglucosamine transferase and deubiquitinase	1.159	0.318334365
Calumenin [OS=Homo sapiens]	1.16	0.014420469
Glycylpeptide N-tetradecanoyltransferase 1 [OS=Homo sapiens]	1.16	0.042223592
Tuberin [OS=Homo sapiens]	1.16	0.047571468
BH3-interacting domain death agonist [OS=Homo sapiens]	1.16	0.009127609
Acidic leucine-rich nuclear phosphoprotein 32 family member E [OS=Homo sap	1.16	0.046302229
GDP-fucose protein O-fucosyltransferase 1 [OS=Homo sapiens]	1.16	0.38883121
Transmembrane emp24 domain-containing protein 7 [OS=Homo sapiens]	1.16	0.980254098
Cyclin-G-associated kinase [OS=Homo sapiens]	1.16	0.01031264
DNA dC->dU-editing enzyme APOBEC-3F [OS=Homo sapiens]	1.16	0.021908328
MARCKS-related protein [OS=Homo sapiens]	1.16	0.006853346
Choline transporter-like protein 2 [OS=Homo sapiens]	1.16	0.047364771
MAP kinase-interacting serine/threonine-protein kinase 1 [OS=Homo sapiens]	1.16	0.385083158
Mesoderm induction early response protein 2 [OS=Homo sapiens]	1.16	0.993294456
Hydroxysteroid dehydrogenase-like protein 2 [OS=Homo sapiens]	1.161	0.013584614
Malate dehydrogenase, mitochondrial [OS=Homo sapiens]	1.161	0.023969913
Ethylmalonyl-CoA decarboxylase [OS=Homo sapiens]	1.161	0.069193849
Unconventional prefoldin RPB5 interactor 1 [OS=Homo sapiens]	1.161	0.160608514
Protein FAM136A [OS=Homo sapiens]	1.161	0.099697102
Hippocalcin-like protein 1 [OS=Homo sapiens]	1.161	0.013434211
Rab5 GDP/GTP exchange factor [OS=Homo sapiens]	1.161	0.438295251
Microtubule nucleation factor SSNA1 [OS=Homo sapiens]	1.161	0.159057578
Protein TANC1 [OS=Homo sapiens]	1.161	0.008127144
Ubiquitin-conjugating enzyme E2 H [OS=Homo sapiens]	1.161	0.011432646
Piezo-type mechanosensitive ion channel component 1 [OS=Homo sapiens]	1.161	0.977583511
Myotubularin-related protein 10 [OS=Homo sapiens]	1.161	0.354482655
DNA damage-binding protein 1 [OS=Homo sapiens]	1.162	0.01064611
Dihydropyrimidinase-related protein 2 [OS=Homo sapiens]	1.162	0.008844899
Exportin-7 [OS=Homo sapiens]	1.162	0.07664873
Lipid droplet-regulating VLDL assembly factor AUP1 [OS=Homo sapiens]	1.162	0.00488872

Mitogen-activated protein kinase kinase kinase kinase 2 [OS=Homo sapiens]	1.162	0.021997517
Probable arginine--tRNA ligase, mitochondrial [OS=Homo sapiens]	1.162	0.108267558
Protein NDRG1 [OS=Homo sapiens]	1.162	0.196604896
Lysophosphatidylserine lipase ABHD12 [OS=Homo sapiens]	1.162	0.027861619
Deoxyribose-phosphate aldolase [OS=Homo sapiens]	1.162	0.82254771
COMM domain-containing protein 9 [OS=Homo sapiens]	1.162	0.685232051
Phosphatidylinositol 4,5-bisphosphate 3-kinase catalytic subunit delta isoform [	1.162	0.851309083
Ribonuclease T2 [OS=Homo sapiens]	1.162	0.960779265
Serine/threonine-protein kinase mTOR [OS=Homo sapiens]	1.163	0.02384354
Hsp90 co-chaperone Cdc37 [OS=Homo sapiens]	1.163	0.014057183
Serpin B6 [OS=Homo sapiens]	1.163	0.008468891
Reticulocalbin-2 [OS=Homo sapiens]	1.163	0.002653276
Ras-related protein Rab-1A [OS=Homo sapiens]	1.163	0.110357614
Programmed cell death protein 6 [OS=Homo sapiens]	1.163	0.19927026
Thioredoxin-dependent peroxide reductase, mitochondrial [OS=Homo sapiens]	1.163	0.004627913
5-oxoprolinase [OS=Homo sapiens]	1.163	0.06283609
Nuclear receptor coactivator 2 [OS=Homo sapiens]	1.163	0.286159189
Apolipoprotein B-100 [OS=Homo sapiens]	1.163	0.744646784
Zinc finger protein 771 [OS=Homo sapiens]	1.163	0.588522572
NEDD4-binding protein 1 [OS=Homo sapiens]	1.163	0.140897515
Elongation factor Tu, mitochondrial [OS=Homo sapiens]	1.164	0.01596654
Serine--tRNA ligase, mitochondrial [OS=Homo sapiens]	1.164	0.090906631
Pyruvate dehydrogenase E1 component subunit beta, mitochondrial [OS=Homo	1.164	0.000471229
Elongin-C [OS=Homo sapiens]	1.164	0.009475222
RNA 3'-terminal phosphate cyclase [OS=Homo sapiens]	1.164	0.13093504
Adaptin ear-binding coat-associated protein 2 [OS=Homo sapiens]	1.164	0.016123545
Ubiquitin/ISG15-conjugating enzyme E2 L6 [OS=Homo sapiens]	1.164	0.233362153
HEAT repeat-containing protein 5B [OS=Homo sapiens]	1.164	0.256065746
Leucine carboxyl methyltransferase 1 [OS=Homo sapiens]	1.164	0.149797312
AN1-type zinc finger protein 1 [OS=Homo sapiens]	1.164	0.026350888
Aftiphilin [OS=Homo sapiens]	1.164	0.358825126
Ubiquitin-conjugating enzyme E2 D1 [OS=Homo sapiens]	1.164	0.140826402
Rabenosyn-5 [OS=Homo sapiens]	1.164	0.124250675
Protein disulfide-isomerase [OS=Homo sapiens]	1.165	0.039398453
Isocitrate dehydrogenase [NADP], mitochondrial [OS=Homo sapiens]	1.165	0.029563481
Sorting nexin-6 [OS=Homo sapiens]	1.165	0.062982931
1-phosphatidylinositol 4,5-bisphosphate phosphodiesterase beta-3 [OS=Homo	1.165	0.027785154
mRNA cap guanine-N7 methyltransferase [OS=Homo sapiens]	1.165	0.011518903
Phosphatidylinositol transfer protein alpha isoform [OS=Homo sapiens]	1.165	0.017992686
F-box/LRR-repeat protein 18 [OS=Homo sapiens]	1.165	0.15311092
Hydroxyacylglutathione hydrolase, mitochondrial [OS=Homo sapiens]	1.165	0.314758973
Glycogen synthase kinase-3 alpha [OS=Homo sapiens]	1.165	0.080640678
Protein RER1 [OS=Homo sapiens]	1.165	0.950738593
Bisphosphoglycerate mutase [OS=Homo sapiens]	1.165	0.128487775
Microtubule cross-linking factor 1 [OS=Homo sapiens]	1.165	0.016488283
Phospholipid-transporting ATPase ABCA3 [OS=Homo sapiens]	1.165	0.861664538
Aconitate hydratase, mitochondrial [OS=Homo sapiens]	1.166	0.020236067
Presequence protease, mitochondrial [OS=Homo sapiens]	1.166	0.003763203
E3 ubiquitin-protein ligase UBR1 [OS=Homo sapiens]	1.166	0.066968958
ADP-ribosylation factor-related protein 1 [OS=Homo sapiens]	1.166	0.927733285
Sorting nexin-24 [OS=Homo sapiens]	1.166	0.899883481
Ubiquitin carboxyl-terminal hydrolase 8 [OS=Homo sapiens]	1.167	0.023236135
Caldesmon [OS=Homo sapiens]	1.167	0.008963211
Translation initiation factor eIF-2B subunit delta [OS=Homo sapiens]	1.167	0.002124007
PCI domain-containing protein 2 [OS=Homo sapiens]	1.167	0.092842742
Aspartyl aminopeptidase [OS=Homo sapiens]	1.167	0.032427424
Integrin alpha-2 [OS=Homo sapiens]	1.167	0.137433383
Pyridoxal phosphate homeostasis protein [OS=Homo sapiens]	1.167	0.034106398
Tetratricopeptide repeat protein 17 [OS=Homo sapiens]	1.167	0.147374117
Polyamine-transporting ATPase 13A3 [OS=Homo sapiens]	1.167	0.656904139
Procollagen-lysine,2-oxoglutarate 5-dioxygenase 1 [OS=Homo sapiens]	1.168	0.002086771
Melanoma-associated antigen 9 [OS=Homo sapiens]	1.168	0.170844703
Isocitrate dehydrogenase [NAD] subunit alpha, mitochondrial [OS=Homo sapier	1.168	0.027669739
3-hydroxyisobutyryl-CoA hydrolase, mitochondrial [OS=Homo sapiens]	1.168	0.076530603
Arf-GAP domain and FG repeat-containing protein 2 [OS=Homo sapiens]	1.168	0.745266439
AP-3 complex subunit sigma-1 [OS=Homo sapiens]	1.168	0.02363265
Secretory carrier-associated membrane protein 2 [OS=Homo sapiens]	1.168	0.086397403
Costars family protein ABRACL [OS=Homo sapiens]	1.168	0.650829287
Uncharacterized protein C17orf80 [OS=Homo sapiens]	1.168	0.917736826
Ubiquitin-like modifier-activating enzyme 1 [OS=Homo sapiens]	1.169	0.113920264
Phosphatidylinositol-binding clathrin assembly protein [OS=Homo sapiens]	1.169	0.006495205
Serine/threonine-protein phosphatase 4 regulatory subunit 2 [OS=Homo sapien:	1.169	0.051740113
Isopentenyl-diphosphate Delta-isomerase 1 [OS=Homo sapiens]	1.169	0.237897172
PDZ domain-containing protein 11 [OS=Homo sapiens]	1.169	0.019065928
LYR motif-containing protein 4 [OS=Homo sapiens]	1.169	0.096179337

Ankyrin repeat domain-containing protein 40 [OS=Homo sapiens]	1.169	0.380986148
F-box only protein 30 [OS=Homo sapiens]	1.169	0.500497086
Dysbindin [OS=Homo sapiens]	1.169	0.170684886
Alpha-1,3-mannosyl-glycoprotein 2-beta-N-acetylglucosaminyltransferase [OS=	1.169	0.709486597
Microtubule-associated protein RP/EB family member 1 [OS=Homo sapiens]	1.17	0.060169264
Ubiquitin carboxyl-terminal hydrolase isozyme L1 [OS=Homo sapiens]	1.17	0.095341799
Activated RNA polymerase II transcriptional coactivator p15 [OS=Homo sapiens	1.17	0.018493812
Transcription initiation factor IIE subunit beta [OS=Homo sapiens]	1.17	0.034363928
SH3 and PX domain-containing protein 2B [OS=Homo sapiens]	1.17	0.012840434
Leucine-rich repeat-containing protein 20 [OS=Homo sapiens]	1.17	0.070006293
Methionyl-tRNA formyltransferase, mitochondrial [OS=Homo sapiens]	1.17	0.222935488
Cytosolic Fe-S cluster assembly factor NUBP2 [OS=Homo sapiens]	1.17	0.806671401
Tetraspanin-4 [OS=Homo sapiens]	1.17	0.222101559
Radial spoke head protein 3 homolog [OS=Homo sapiens]	1.17	0.851946326
ATP-dependent DNA helicase Q1 [OS=Homo sapiens]	1.171	0.191155372
La-related protein 1 [OS=Homo sapiens]	1.171	0.001848256
Anion exchange protein 2 [OS=Homo sapiens]	1.171	0.774681955
Carnitine O-palmitoyltransferase 2, mitochondrial [OS=Homo sapiens]	1.171	0.002270707
Cofilin-2 [OS=Homo sapiens]	1.171	0.017926563
Protein ERGIC-53 [OS=Homo sapiens]	1.171	0.20960559
Dedicator of cytokinesis protein 1 [OS=Homo sapiens]	1.171	0.01105451
Ubiquitin-associated protein 1 [OS=Homo sapiens]	1.171	0.067213854
Proteasome subunit beta type-6 [OS=Homo sapiens]	1.171	0.30572546
Nuclear transport factor 2 [OS=Homo sapiens]	1.171	0.191078204
Steroid receptor RNA activator 1 [OS=Homo sapiens]	1.171	0.14614752
Leukocyte surface antigen CD47 [OS=Homo sapiens]	1.171	0.999974241
Neudesin [OS=Homo sapiens]	1.171	0.039853429
Tetratricopeptide repeat protein 5 [OS=Homo sapiens]	1.171	0.355347907
BLOC-1-related complex subunit 5 [OS=Homo sapiens]	1.171	0.439583925
Zinc finger protein 384 [OS=Homo sapiens]	1.171	0.819649125
Coatomer subunit alpha [OS=Homo sapiens]	1.172	0.005399433
Lon protease homolog, mitochondrial [OS=Homo sapiens]	1.172	0.001308882
Neurolysin, mitochondrial [OS=Homo sapiens]	1.172	0.044835089
Reticulon-4 [OS=Homo sapiens]	1.172	0.007943732
Transcription factor BTF3 homolog 4 [OS=Homo sapiens]	1.172	0.078899079
Ubiquinone biosynthesis protein COQ9, mitochondrial [OS=Homo sapiens]	1.172	0.003752428
Protein NOXP20 [OS=Homo sapiens]	1.172	0.024189238
Ankyrin repeat domain-containing protein SOWAHC [OS=Homo sapiens]	1.172	0.395688282
FAU ubiquitin-like and ribosomal protein S30 [OS=Homo sapiens]	1.172	0.812236591
Deoxyguanosine kinase, mitochondrial [OS=Homo sapiens]	1.172	0.062036309
Biogenesis of lysosome-related organelles complex 1 subunit 2 [OS=Homo sapi	1.172	0.744424777
Methylcrotonoyl-CoA carboxylase beta chain, mitochondrial [OS=Homo sapiens	1.173	0.0513374
Replication protein A 32 kDa subunit [OS=Homo sapiens]	1.173	0.002584152
Lysosomal acid glucosylceramidase [OS=Homo sapiens]	1.173	0.000358189
Coactosin-like protein [OS=Homo sapiens]	1.173	0.00583658
Protein O-mannosyl-transferase TMTC3 [OS=Homo sapiens]	1.173	0.618775501
ATPase inhibitor, mitochondrial [OS=Homo sapiens]	1.173	0.015259363
G patch domain-containing protein 2 [OS=Homo sapiens]	1.173	0.159250872
28S ribosomal protein S18c, mitochondrial [OS=Homo sapiens]	1.173	0.988953032
Endonuclease G, mitochondrial [OS=Homo sapiens]	1.173	0.114798214
FUN14 domain-containing protein 1 [OS=Homo sapiens]	1.173	0.988423325
N-acetylglucosamine-6-phosphate deacetylase [OS=Homo sapiens]	1.173	0.397462832
Centromere-associated protein E [OS=Homo sapiens]	1.173	0.903154706
Microtubule-associated protein 4 [OS=Homo sapiens]	1.174	0.00469119
Tyrosine-protein phosphatase non-receptor type 11 [OS=Homo sapiens]	1.174	0.041067928
Adenylate kinase isoenzyme 1 [OS=Homo sapiens]	1.174	0.006694613
Histone acetyltransferase p300 [OS=Homo sapiens]	1.174	0.15249465
BolA-like protein 2 [OS=Homo sapiens]	1.174	0.002272884
NFX1-type zinc finger-containing protein 1 [OS=Homo sapiens]	1.174	0.003525691
Fumarylacetoacetate hydrolase domain-containing protein 2A [OS=Homo sapie	1.174	0.1616362
Vacuolar protein sorting-associated protein 37A [OS=Homo sapiens]	1.174	0.803263243
Tubulin beta-2B chain [OS=Homo sapiens]	1.175	0.032238049
Lanosterol synthase [OS=Homo sapiens]	1.175	0.004315462
1-phosphatidylinositol 4,5-bisphosphate phosphodiesterase gamma-1 [OS=Hor	1.175	0.065131084
[F-actin]-monooxygenase MICAL1 [OS=Homo sapiens]	1.175	0.528192044
GPN-loop GTPase 3 [OS=Homo sapiens]	1.175	0.853966097
Derlin-2 [OS=Homo sapiens]	1.175	0.212989062
Dymeclin [OS=Homo sapiens]	1.175	0.886528882
Nucleotide-binding oligomerization domain-containing protein 1 [OS=Homo sap	1.175	0.66923761
Heat shock 70 kDa protein 4 [OS=Homo sapiens]	1.176	0.007730107
14-3-3 protein zeta/delta [OS=Homo sapiens]	1.176	0.009168852
Mitochondrial-processing peptidase subunit alpha [OS=Homo sapiens]	1.176	0.019845365
Putative glutamine amidotransferase-like class 1 domain-containing protein 3B,	1.176	0.023742911
Focal adhesion kinase 1 [OS=Homo sapiens]	1.176	0.083371833
Sorting nexin-5 [OS=Homo sapiens]	1.176	0.004618556

N(G),N(G)-dimethylarginine dimethylaminohydrolase 2 [OS=Homo sapiens]	1.176	0.020578977
Quinone oxidoreductase [OS=Homo sapiens]	1.176	0.01816911
Receptor-interacting serine/threonine-protein kinase 2 [OS=Homo sapiens]	1.176	0.813236196
Frizzled-2 [OS=Homo sapiens]	1.176	0.985658355
Calcium-binding and coiled-coil domain-containing protein 1 [OS=Homo sapien	1.176	0.999999732
Sorting nexin-15 [OS=Homo sapiens]	1.176	0.239410069
Oligosaccharyltransferase complex subunit OSTC [OS=Homo sapiens]	1.176	0.639374659
Trifunctional enzyme subunit beta, mitochondrial [OS=Homo sapiens]	1.177	0.044198096
Nucleobindin-1 [OS=Homo sapiens]	1.177	0.017368239
Transmembrane emp24 domain-containing protein 2 [OS=Homo sapiens]	1.177	0.999831453
GTP-binding protein 1 [OS=Homo sapiens]	1.177	0.007987579
Oligoribonuclease, mitochondrial [OS=Homo sapiens]	1.177	0.02177218
Propionyl-CoA carboxylase alpha chain, mitochondrial [OS=Homo sapiens]	1.177	0.011802739
CD99 antigen [OS=Homo sapiens]	1.177	0.113594004
Transmembrane protein 263 [OS=Homo sapiens]	1.177	0.104436062
UPF0538 protein C2orf76 [OS=Homo sapiens]	1.177	0.421387772
Tudor-interacting repair regulator protein [OS=Homo sapiens]	1.177	0.998746902
E3 ubiquitin-protein ligase TRIM37 [OS=Homo sapiens]	1.177	0.444677192
Apolipoprotein M [OS=Homo sapiens]	1.177	0.601556881
Neurofibromin [OS=Homo sapiens]	1.178	0.008061
Ubiquitin-associated protein 2 [OS=Homo sapiens]	1.178	0.023577678
Anoctamin-6 [OS=Homo sapiens]	1.178	0.403687781
Serine/threonine-protein phosphatase 2A catalytic subunit alpha isoform [OS=H	1.178	0.077886136
Segment polarity protein dishevelled homolog DVL-3 [OS=Homo sapiens]	1.178	0.996503531
Cytochrome c oxidase assembly factor 7 [OS=Homo sapiens]	1.178	0.084847119
Peptidyl-prolyl cis-trans isomerase NIMA-interacting 1 [OS=Homo sapiens]	1.178	0.515209199
TATA box-binding protein-associated factor RNA polymerase I subunit B [OS=Ho	1.178	0.69852231
Tripeptidyl-peptidase 2 [OS=Homo sapiens]	1.179	0.292295438
Apoptosis-inducing factor 1, mitochondrial [OS=Homo sapiens]	1.179	0.035301393
Angio-associated migratory cell protein [OS=Homo sapiens]	1.179	0.071044998
2-hydroxyacyl-CoA lyase 2 [OS=Homo sapiens]	1.179	0.107213062
Cytosolic purine 5'-nucleotidase [OS=Homo sapiens]	1.179	0.356687487
Basement membrane-specific heparan sulfate proteoglycan core protein [OS=H	1.179	0.004140738
Endoplasmic reticulum-Golgi intermediate compartment protein 1 [OS=Homo s	1.179	0.029053449
Ion channel TACAN [OS=Homo sapiens]	1.179	0.927675212
Low density lipoprotein receptor adapter protein 1 [OS=Homo sapiens]	1.179	0.261675139
HSPB1-associated protein 1 [OS=Homo sapiens]	1.179	0.749058788
Phosphatidate cytidylyltransferase, mitochondrial [OS=Homo sapiens]	1.179	0.907748597
Protein transport protein Sec31A [OS=Homo sapiens]	1.18	0.030583127
Phenylalanine--tRNA ligase beta subunit [OS=Homo sapiens]	1.18	0.023150058
Sorting nexin-27 [OS=Homo sapiens]	1.18	0.002104836
3'(2'),5'-bisphosphate nucleotidase 1 [OS=Homo sapiens]	1.18	0.165091934
HLA class I histocompatibility antigen, B alpha chain [OS=Homo sapiens]	1.18	0.491164122
Prenylcysteine oxidase 1 [OS=Homo sapiens]	1.18	0.022239922
PDZ and LIM domain protein 4 [OS=Homo sapiens]	1.18	0.091638654
Reticulon-4-interacting protein 1, mitochondrial [OS=Homo sapiens]	1.18	0.125673313
Protein tyrosine phosphatase type IVA 2 [OS=Homo sapiens]	1.18	0.042854561
Vacuolar ATPase assembly integral membrane protein VMA21 [OS=Homo sapier	1.18	0.473324449
Calmin [OS=Homo sapiens]	1.18	0.357343077
Thrombospondin-4 [OS=Homo sapiens]	1.18	0.235462652
DET1- and DDB1-associated protein 1 [OS=Homo sapiens]	1.18	0.023403544
Probable RNA polymerase II nuclear localization protein SLC7A6OS [OS=Homo s	1.18	0.233738361
Peptidyl-prolyl cis-trans isomerase B [OS=Homo sapiens]	1.181	0.03681879
Diphosphomevalonate decarboxylase [OS=Homo sapiens]	1.181	0.112334156
NADPH-dependent diflavin oxidoreductase 1 [OS=Homo sapiens]	1.181	0.039456061
AP-2 complex subunit sigma [OS=Homo sapiens]	1.181	0.087787058
MAP kinase-activating death domain protein [OS=Homo sapiens]	1.181	0.056660197
TBC1 domain family member 7 [OS=Homo sapiens]	1.181	0.103009343
Trifunctional enzyme subunit alpha, mitochondrial [OS=Homo sapiens]	1.182	0.000247349
Vacuolar protein sorting-associated protein 4B [OS=Homo sapiens]	1.182	0.000689389
Chromodomain-helicase-DNA-binding protein 1 [OS=Homo sapiens]	1.182	0.034539466
Glucosamine 6-phosphate N-acetyltransferase [OS=Homo sapiens]	1.182	0.347623768
Zinc finger FYVE domain-containing protein 16 [OS=Homo sapiens]	1.182	0.132964446
Calcium/calmodulin-dependent protein kinase type 1D [OS=Homo sapiens]	1.182	0.065014231
Protein FAM117B [OS=Homo sapiens]	1.182	0.323516792
MAP/microtubule affinity-regulating kinase 4 [OS=Homo sapiens]	1.182	0.000385012
Protein Hook homolog 2 [OS=Homo sapiens]	1.182	0.990099204
Acetyl-CoA carboxylase 1 [OS=Homo sapiens]	1.183	0.002591776
Insulin-like growth factor 2 mRNA-binding protein 2 [OS=Homo sapiens]	1.183	3.46426E-05
ADP-ribosylation factor 1 [OS=Homo sapiens]	1.183	0.05390389
Dipeptidyl peptidase 9 [OS=Homo sapiens]	1.183	0.01321202
Enoyl-CoA delta isomerase 1, mitochondrial [OS=Homo sapiens]	1.183	0.00920898
Ras-related GTP-binding protein C [OS=Homo sapiens]	1.183	0.414899941
2-iminobutanoate/2-iminopropanoate deaminase [OS=Homo sapiens]	1.183	0.843810125
Endosome-associated-trafficking regulator 1 [OS=Homo sapiens]	1.183	0.013278955

Zinc-regulated GTPase metalloprotein activator 1F [OS=Homo sapiens]	1.183	0.749404227
Plasma membrane calcium-transporting ATPase 1 [OS=Homo sapiens]	1.184	0.315823258
Charged multivesicular body protein 4b [OS=Homo sapiens]	1.184	0.014236709
Single-stranded DNA-binding protein 4 [OS=Homo sapiens]	1.184	0.027786663
Myotubularin-related protein 1 [OS=Homo sapiens]	1.184	0.108870883
Calpain-1 catalytic subunit [OS=Homo sapiens]	1.185	0.050224732
Transmembrane emp24 domain-containing protein 9 [OS=Homo sapiens]	1.185	0.017341336
Alpha-mannosidase 2 [OS=Homo sapiens]	1.185	0.443850931
Peptidyl-prolyl cis-trans isomerase FKBP2 [OS=Homo sapiens]	1.185	0.027418808
Mitochondrial intermediate peptidase [OS=Homo sapiens]	1.185	0.019773242
HLA class I histocompatibility antigen, C alpha chain [OS=Homo sapiens]	1.185	0.305801584
Hemoglobin subunit alpha [OS=Homo sapiens]	1.185	0.481598699
Apolipoprotein A-I [OS=Homo sapiens]	1.185	0.758154694
WD repeat domain phosphoinositide-interacting protein 2 [OS=Homo sapiens]	1.185	0.001700117
Dolichyl-phosphate beta-glucosyltransferase [OS=Homo sapiens]	1.185	0.346226795
Methylmalonyl-CoA epimerase, mitochondrial [OS=Homo sapiens]	1.185	0.016304293
Annexin A5 [OS=Homo sapiens]	1.186	0.004756847
Ras-related protein Rab-8B [OS=Homo sapiens]	1.186	0.06423224
Putative lipid scramblase CLPTM1 [OS=Homo sapiens]	1.186	0.240151087
Tyrosine-protein kinase JAK1 [OS=Homo sapiens]	1.186	0.171951158
Serine/threonine-protein phosphatase 6 regulatory subunit 2 [OS=Homo sapiens	1.186	0.007131356
Iron-responsive element-binding protein 2 [OS=Homo sapiens]	1.186	0.001653432
Ras-related protein Rab-24 [OS=Homo sapiens]	1.186	0.039047808
Iron-sulfur cluster assembly 2 homolog, mitochondrial [OS=Homo sapiens]	1.186	0.300591896
Alsin [OS=Homo sapiens]	1.186	0.533292257
Cytoplasmic aconitate hydratase [OS=Homo sapiens]	1.187	0.018793872
Leucine--tRNA ligase, mitochondrial [OS=Homo sapiens]	1.187	0.007897172
Armadillo-like helical domain-containing protein 3 [OS=Homo sapiens]	1.187	0.025697161
Casein kinase I isoform delta [OS=Homo sapiens]	1.187	0.401378565
Vam6/Vps39-like protein [OS=Homo sapiens]	1.187	0.184981633
Ras-related protein Rab-23 [OS=Homo sapiens]	1.187	0.603259091
Transcription initiation factor IIA subunit 1 [OS=Homo sapiens]	1.187	0.165613756
Cytosolic iron-sulfur assembly component 2A [OS=Homo sapiens]	1.187	0.315569973
E3 SUMO-protein ligase PIAS3 [OS=Homo sapiens]	1.187	0.090162628
UPF0235 protein C15orf40 [OS=Homo sapiens]	1.187	0.098968946
Tubulin polyglutamylase complex subunit 1 [OS=Homo sapiens]	1.187	0.999999116
Major vault protein [OS=Homo sapiens]	1.188	0.001622763
Glutamate dehydrogenase 1, mitochondrial [OS=Homo sapiens]	1.188	0.0087239
Ribosomal protein S6 kinase alpha-1 [OS=Homo sapiens]	1.188	0.035647952
Transaldolase [OS=Homo sapiens]	1.188	0.012415258
UPF0489 protein C5orf22 [OS=Homo sapiens]	1.188	0.130605683
Carboxymethylenebutenolidase homolog [OS=Homo sapiens]	1.188	0.009158918
Malonyl-CoA-acyl carrier protein transacylase, mitochondrial [OS=Homo sapien	1.188	0.007082379
Zinc finger protein 428 [OS=Homo sapiens]	1.188	0.999981535
Hsp90 co-chaperone Cdc37-like 1 [OS=Homo sapiens]	1.188	0.334467223
F-box only protein 7 [OS=Homo sapiens]	1.188	0.592571382
Coiled-coil domain-containing protein 70 [OS=Homo sapiens]	1.188	0.493083575
High mobility group nucleosome-binding domain-containing protein 3 [OS=Hom	1.188	0.702795265
Spermidine synthase [OS=Homo sapiens]	1.189	0.055720931
Glutamate--cysteine ligase catalytic subunit [OS=Homo sapiens]	1.189	0.018257818
PDZ domain-containing protein GIPC1 [OS=Homo sapiens]	1.189	0.092261035
CTTNBP2 N-terminal-like protein [OS=Homo sapiens]	1.189	0.005615417
Delta(24)-sterol reductase [OS=Homo sapiens]	1.189	0.253552065
Repressor of RNA polymerase III transcription MAF1 homolog [OS=Homo sapien	1.189	0.883962943
NAD kinase [OS=Homo sapiens]	1.189	0.052132433
N-acetylglucosamine-1-phosphotransferase subunit gamma [OS=Homo sapiens	1.189	0.493326593
Fascin [OS=Homo sapiens]	1.19	0.004888935
Protein disulfide-isomerase A6 [OS=Homo sapiens]	1.19	0.01308211
Plasma membrane calcium-transporting ATPase 4 [OS=Homo sapiens]	1.19	0.44182676
Calcium-binding protein 39 [OS=Homo sapiens]	1.19	0.064330827
Protein PRRC1 [OS=Homo sapiens]	1.19	0.003845414
Ankyrin repeat and BTB/POZ domain-containing protein 2 [OS=Homo sapiens]	1.19	0.086581873
Cilia- and flagella-associated protein 36 [OS=Homo sapiens]	1.19	0.942550605
Somatostatin receptor type 2 [OS=Homo sapiens]	1.19	0.868937887
Cullin-associated NEDD8-dissociated protein 1 [OS=Homo sapiens]	1.191	0.008114771
Phosphoglycerate mutase 1 [OS=Homo sapiens]	1.191	0.001781042
Calpain small subunit 1 [OS=Homo sapiens]	1.191	0.10401551
Amyloid-beta precursor protein [OS=Homo sapiens]	1.191	0.005768406
Mitochondrial import inner membrane translocase subunit Tim8 A [OS=Homo sa	1.191	0.751922651
Lys-63-specific deubiquitinase BRCC36 [OS=Homo sapiens]	1.191	0.512039024
Pleckstrin homology domain-containing family A member 5 [OS=Homo sapiens]	1.191	0.223990065
Microtubule-associated protein 1B [OS=Homo sapiens]	1.192	0.000623915
GrpE protein homolog 1, mitochondrial [OS=Homo sapiens]	1.192	0.017521776
Parathymosin [OS=Homo sapiens]	1.192	0.093509933
2-hydroxyacyl-CoA lyase 1 [OS=Homo sapiens]	1.192	0.201329926

Dual specificity mitogen-activated protein kinase kinase 3 [OS=Homo sapiens]	1.192	0.000875646
SPRY domain-containing protein 4 [OS=Homo sapiens]	1.192	0.049196309
CCR4-NOT transcription complex subunit 6-like [OS=Homo sapiens]	1.192	0.455034711
PRA1 family protein 3 [OS=Homo sapiens]	1.192	0.019435958
Protein YIPF6 [OS=Homo sapiens]	1.192	0.685745998
Phosphatidylinositol 3,4,5-trisphosphate-dependent Rac exchanger 2 protein [C	1.192	0.695931794
Triosephosphate isomerase [OS=Homo sapiens]	1.193	0.049745338
Protein canopy homolog 2 [OS=Homo sapiens]	1.193	0.0291289
Coiled-coil domain-containing protein 85C [OS=Homo sapiens]	1.193	0.013666003
Trafficking protein particle complex subunit 13 [OS=Homo sapiens]	1.193	0.529488199
Beta-2-microglobulin [OS=Homo sapiens]	1.193	0.816895041
Peptidyl-prolyl cis-trans isomerase C [OS=Homo sapiens]	1.193	0.003543325
NAD-dependent protein deacetylase sirtuin-3, mitochondrial [OS=Homo sapien	1.193	0.030114945
Synaptogyrin-2 [OS=Homo sapiens]	1.193	0.852060869
Elongation factor 1-alpha 2 [OS=Homo sapiens]	1.194	0.271545026
Prolyl 3-hydroxylase 1 [OS=Homo sapiens]	1.194	0.003400378
Serine/threonine-protein phosphatase 4 catalytic subunit [OS=Homo sapiens]	1.194	0.057385396
Protein S100-A11 [OS=Homo sapiens]	1.194	0.824167354
DNA-directed RNA polymerases I and III subunit RPAC1 [OS=Homo sapiens]	1.194	0.014590632
60S ribosomal protein L14 [OS=Homo sapiens]	1.194	0.479771856
Negative elongation factor C/D [OS=Homo sapiens]	1.194	0.044984791
COMM domain-containing protein 2 [OS=Homo sapiens]	1.194	0.0224959
Microtubule-associated protein 6 [OS=Homo sapiens]	1.194	0.107346709
Procollagen galactosyltransferase 1 [OS=Homo sapiens]	1.195	0.000172046
Polypeptide N-acetylgalactosaminyltransferase 2 [OS=Homo sapiens]	1.195	0.934320504
ATP-dependent 6-phosphofructokinase, muscle type [OS=Homo sapiens]	1.195	0.007558477
DCC-interacting protein 13-alpha [OS=Homo sapiens]	1.195	0.044761975
CCHC-type zinc finger nucleic acid binding protein [OS=Homo sapiens]	1.195	0.307190034
ADP-ribosylation factor-like protein 3 [OS=Homo sapiens]	1.195	0.231029263
Rho guanine nucleotide exchange factor 18 [OS=Homo sapiens]	1.195	0.264618727
CD99 antigen-like protein 2 [OS=Homo sapiens]	1.195	0.04811314
Guided entry of tail-anchored proteins factor CAMLG [OS=Homo sapiens]	1.195	0.125296788
52 kDa repressor of the inhibitor of the protein kinase [OS=Homo sapiens]	1.195	0.171974617
Signal recognition particle receptor subunit beta [OS=Homo sapiens]	1.196	0.02469205
Ephrin type-B receptor 4 [OS=Homo sapiens]	1.196	0.097370782
Pigment epithelium-derived factor [OS=Homo sapiens]	1.196	0.99988283
FHF complex subunit HOOK interacting protein 2B [OS=Homo sapiens]	1.196	0.355495695
Band 4.1-like protein 4B [OS=Homo sapiens]	1.196	0.981120866
Gamma-aminobutyric acid receptor-associated protein-like 2 [OS=Homo sapier	1.196	0.128254276
Ferritin light chain [OS=Homo sapiens]	1.196	0.110666943
Peroxiredoxin-4 [OS=Homo sapiens]	1.197	0.369764851
Microtubule-associated protein RP/EB family member 2 [OS=Homo sapiens]	1.197	0.001375813
6-phosphogluconolactonase [OS=Homo sapiens]	1.198	0.004967748
Mitochondrial-processing peptidase subunit beta [OS=Homo sapiens]	1.198	0.031599253
Acyl-CoA dehydrogenase family member 10 [OS=Homo sapiens]	1.198	0.104563597
Myotubularin-related protein 12 [OS=Homo sapiens]	1.198	0.024325505
Sickle tail protein homolog [OS=Homo sapiens]	1.198	0.037180701
Solute carrier family 12 member 9 [OS=Homo sapiens]	1.198	0.025983824
Cullin-2 [OS=Homo sapiens]	1.199	0.006064597
Glyoxylate reductase/hydroxypyruvate reductase [OS=Homo sapiens]	1.199	0.087175574
Bridge-like lipid transfer protein family member 3B [OS=Homo sapiens]	1.199	0.006421359
Small nuclear ribonucleoprotein F [OS=Homo sapiens]	1.199	0.999264173
E3 ubiquitin-protein ligase RNF216 [OS=Homo sapiens]	1.199	0.57355803
Mitogen-activated protein kinase 1 [OS=Homo sapiens]	1.2	0.002219725
Phosphatidylinositol-3-phosphatase SAC1 [OS=Homo sapiens]	1.2	0.009096858
Ubiquitin-conjugating enzyme E2 K [OS=Homo sapiens]	1.2	0.001896038
Ubiquitin-like domain-containing CTD phosphatase 1 [OS=Homo sapiens]	1.2	0.024935438
Oxysterol-binding protein-related protein 1 [OS=Homo sapiens]	1.2	0.067169492
Melanoma-associated antigen D1 [OS=Homo sapiens]	1.2	0.000491507
WD repeat-containing protein 55 [OS=Homo sapiens]	1.2	0.029141188
Intron Large complex component GCFC2 [OS=Homo sapiens]	1.2	0.250358671
Protein Njmu-R1 [OS=Homo sapiens]	1.2	0.05555006
Tumor necrosis factor receptor superfamily member 5 [OS=Homo sapiens]	1.2	0.979892905
Coatomer subunit gamma-1 [OS=Homo sapiens]	1.201	0.061386185
Glyoxalase domain-containing protein 4 [OS=Homo sapiens]	1.201	0.001982317
Acyl-coenzyme A thioesterase 1 [OS=Homo sapiens]	1.201	0.023214871
Kinesin-like protein KIF21A [OS=Homo sapiens]	1.201	0.002530123
RNA-binding protein 12 [OS=Homo sapiens]	1.201	0.066653732
Prolyl 4-hydroxylase subunit alpha-2 [OS=Homo sapiens]	1.201	0.005576148
Glycine dehydrogenase (decarboxylating), mitochondrial [OS=Homo sapiens]	1.201	0.063289411
Abl interactor 2 [OS=Homo sapiens]	1.201	0.028769514
Rho family-interacting cell polarization regulator 1 [OS=Homo sapiens]	1.201	0.130912339
BRCA1-associated protein [OS=Homo sapiens]	1.201	0.023443455
Beta-1,3-glucosyltransferase [OS=Homo sapiens]	1.201	0.077601382
Tropomyosin alpha-4 chain [OS=Homo sapiens]	1.202	0.000909871

Insulin-like growth factor 1 receptor [OS=Homo sapiens]	1.202	0.565282844
Dynein axonemal light chain 1 [OS=Homo sapiens]	1.202	0.012225799
Membralin [OS=Homo sapiens]	1.202	0.663910557
Apolipoprotein E [OS=Homo sapiens]	1.203	0.022320302
Epidermal growth factor receptor substrate 15 [OS=Homo sapiens]	1.203	0.002852917
Palmitoyl-protein thioesterase ABHD10, mitochondrial [OS=Homo sapiens]	1.203	0.085727388
Membrane-associated progesterone receptor component 2 [OS=Homo sapiens]	1.203	0.077378109
Acetyl-CoA acetyltransferase, mitochondrial [OS=Homo sapiens]	1.204	0.002440651
Very-long-chain 3-oxoacyl-CoA reductase [OS=Homo sapiens]	1.204	0.084049715
Phosphatidylserine lipase ABHD16A [OS=Homo sapiens]	1.204	0.000336187
F-box only protein 21 [OS=Homo sapiens]	1.204	0.021771281
AP-5 complex subunit zeta-1 [OS=Homo sapiens]	1.204	0.875270018
TSC22 domain family protein 1 [OS=Homo sapiens]	1.204	0.005975456
Probable bifunctional dTTP/UTP pyrophosphatase/methyltransferase protein [OS	1.205	0.035911542
Protein S100-A6 [OS=Homo sapiens]	1.205	0.008402099
Zinc finger protein-like 1 [OS=Homo sapiens]	1.205	0.470904387
[F-actin]-monooxygenase MICAL2 [OS=Homo sapiens]	1.205	0.443702208
Isocitrate dehydrogenase [NAD] subunit gamma, mitochondrial [OS=Homo sapi	1.205	0.585927333
Ubiquilin-4 [OS=Homo sapiens]	1.206	0.000838051
Beta-hexosaminidase subunit alpha [OS=Homo sapiens]	1.206	0.07108273
Engulfment and cell motility protein 2 [OS=Homo sapiens]	1.206	0.010156986
Peptidyl-prolyl cis-trans isomerase NIMA-interacting 4 [OS=Homo sapiens]	1.206	0.09145762
Inorganic pyrophosphatase 2, mitochondrial [OS=Homo sapiens]	1.206	0.257615874
Endoribonuclease YbeY [OS=Homo sapiens]	1.206	0.225069876
Store-operated calcium entry-associated regulatory factor [OS=Homo sapiens]	1.206	0.446564301
Pyroglutamyl-peptidase 1 [OS=Homo sapiens]	1.206	0.021231929
Golgi resident protein GCP60 [OS=Homo sapiens]	1.207	0.022981264
ADP-ribosylation factor GTPase-activating protein 3 [OS=Homo sapiens]	1.207	0.232173469
DENN domain-containing protein 5A [OS=Homo sapiens]	1.207	0.741270506
Protein YIPF5 [OS=Homo sapiens]	1.207	0.078286921
Oxidation resistance protein 1 [OS=Homo sapiens]	1.207	0.487331992
Selenoprotein K [OS=Homo sapiens]	1.207	0.546170432
Very long-chain specific acyl-CoA dehydrogenase, mitochondrial [OS=Homo sap	1.208	0.007108429
Dedicator of cytokinesis protein 7 [OS=Homo sapiens]	1.208	0.019521629
Protein S100-A4 [OS=Homo sapiens]	1.208	0.112410029
Carnitine O-palmitoyltransferase 1, liver isoform [OS=Homo sapiens]	1.209	0.001270562
WD repeat-containing protein 81 [OS=Homo sapiens]	1.209	0.007849606
Intermembrane lipid transfer protein VPS13A [OS=Homo sapiens]	1.209	0.349304743
Ubiquitin-like modifier-activating enzyme 5 [OS=Homo sapiens]	1.209	0.010243366
Persulfide dioxygenase ETHE1, mitochondrial [OS=Homo sapiens]	1.209	0.170279607
DNA-directed RNA polymerase II subunit GRINL1A, isoforms 4/5 [OS=Homo sap	1.209	0.016968455
Protein YIPF3 [OS=Homo sapiens]	1.209	0.21481105
Type-1 angiotensin II receptor-associated protein [OS=Homo sapiens]	1.209	0.076567239
Arylsulfatase D [OS=Homo sapiens]	1.209	0.51232289
Iron-sulfur cluster assembly enzyme ISCU [OS=Homo sapiens]	1.209	0.003588405
U6 snRNA-associated Sm-like protein LSm6 [OS=Homo sapiens]	1.209	0.001599977
Inosine triphosphate pyrophosphatase [OS=Homo sapiens]	1.21	0.004523996
Phosphatidylinositol 5-phosphate 4-kinase type-2 gamma [OS=Homo sapiens]	1.21	0.397689062
RING finger protein 10 [OS=Homo sapiens]	1.21	0.01884605
Protein O-glucosyltransferase 1 [OS=Homo sapiens]	1.21	0.070760268
Fructosamine-3-kinase [OS=Homo sapiens]	1.21	0.377479084
Sialic acid synthase [OS=Homo sapiens]	1.211	0.056214375
Sorcin [OS=Homo sapiens]	1.211	0.021401952
Autophagy-related protein 2 homolog A [OS=Homo sapiens]	1.211	0.022747026
CDGSH iron-sulfur domain-containing protein 2 [OS=Homo sapiens]	1.211	0.585640752
Spatacsin [OS=Homo sapiens]	1.211	0.54683884
Monoacylglycerol lipase ABHD6 [OS=Homo sapiens]	1.211	0.218177561
Cilium assembly protein DZIP1 [OS=Homo sapiens]	1.211	0.963308363
Serine/threonine-protein kinase N1 [OS=Homo sapiens]	1.212	0.003634776
Sorting nexin-3 [OS=Homo sapiens]	1.212	0.025323985
Protein FAM91A1 [OS=Homo sapiens]	1.212	0.00053743
Delta-1-pyrroline-5-carboxylate dehydrogenase, mitochondrial [OS=Homo sapie	1.213	0.002442099
Protein-arginine deiminase type-2 [OS=Homo sapiens]	1.213	0.856147883
ATP-binding cassette sub-family B member 6 [OS=Homo sapiens]	1.213	0.642650225
Integrin beta-1 [OS=Homo sapiens]	1.214	0.014764016
NAD kinase 2, mitochondrial [OS=Homo sapiens]	1.214	0.001642133
Ubiquitin-conjugating enzyme E2 N [OS=Homo sapiens]	1.214	0.049976663
Retinoid-inducible serine carboxypeptidase [OS=Homo sapiens]	1.214	0.003739821
Calcium/calmodulin-dependent protein kinase type II subunit delta [OS=Homo s	1.214	0.016792351
Cytokine receptor-like factor 3 [OS=Homo sapiens]	1.214	0.01323093
Diphthine--ammonia ligase [OS=Homo sapiens]	1.214	0.021969355
Arf-GAP with GTPase, ANK repeat and PH domain-containing protein 3 [OS=Hom	1.214	0.005006548
Pleckstrin homology domain-containing family F member 2 [OS=Homo sapiens]	1.214	0.646982537
SEC23-interacting protein [OS=Homo sapiens]	1.215	0.002614155
Poly(rC)-binding protein 3 [OS=Homo sapiens]	1.215	0.377074118

RNA polymerase II subunit A C-terminal domain phosphatase [OS=Homo sapien	1.215	0.009073104
HLA class I histocompatibility antigen, A alpha chain [OS=Homo sapiens]	1.215	0.315524918
Myeloid-derived growth factor [OS=Homo sapiens]	1.215	0.001560991
Ubiquitin carboxyl-terminal hydrolase 25 [OS=Homo sapiens]	1.215	0.256103532
Lysine-specific demethylase 5C [OS=Homo sapiens]	1.215	0.047739446
Tumor necrosis factor receptor type 1-associated DEATH domain protein [OS=H	1.215	0.061292642
Coenzyme Q-binding protein COQ10 homolog B, mitochondrial [OS=Homo sapi	1.215	0.441936049
Keratinocyte-associated transmembrane protein 2 [OS=Homo sapiens]	1.215	0.04085552
Regulator of G-protein signaling 3 [OS=Homo sapiens]	1.215	0.999994336
Serine palmitoyltransferase 2 [OS=Homo sapiens]	1.216	0.000352906
Deoxyribonuclease-2-alpha [OS=Homo sapiens]	1.216	0.13108656
Synaptotagmin-like protein 2 [OS=Homo sapiens]	1.216	0.000323162
Leucine-rich repeat and calponin homology domain-containing protein 4 [OS=H	1.216	0.170661251
Serine incorporator 1 [OS=Homo sapiens]	1.216	0.184569917
AN1-type zinc finger protein 6 [OS=Homo sapiens]	1.216	0.007430755
Staphylococcal nuclease domain-containing protein 1 [OS=Homo sapiens]	1.217	0.000662218
ADP-ribosylation factor 5 [OS=Homo sapiens]	1.217	0.002404647
Putative protein-lysine deacylase ABHD14B [OS=Homo sapiens]	1.217	0.009800563
Sialate O-acetylesterase [OS=Homo sapiens]	1.217	0.064716126
N-acetylgalactosaminyltransferase 7 [OS=Homo sapiens]	1.218	0.989047484
Protein NipSnap homolog 2 [OS=Homo sapiens]	1.218	0.024817412
GATOR complex protein MIOS [OS=Homo sapiens]	1.218	0.279320883
mRNA (2'-O-methyladenosine-N(6)-)-methyltransferase [OS=Homo sapiens]	1.218	0.01447897
Nuclear receptor coactivator 1 [OS=Homo sapiens]	1.218	0.11836534
Derlin-1 [OS=Homo sapiens]	1.219	0.8462404
Rab-like protein 6 [OS=Homo sapiens]	1.22	0.013577804
NADPH:adrenodoxin oxidoreductase, mitochondrial [OS=Homo sapiens]	1.22	0.074781302
Cytochrome P450 2S1 [OS=Homo sapiens]	1.22	0.017866573
C-X-C motif chemokine 14 [OS=Homo sapiens]	1.22	0.960837213
Tropomyosin alpha-1 chain [OS=Homo sapiens]	1.221	0.002772507
NF-kappa-B inhibitor beta [OS=Homo sapiens]	1.221	0.020648034
Cyclin-dependent kinase 18 [OS=Homo sapiens]	1.221	0.115460527
Nectin-3 [OS=Homo sapiens]	1.221	0.598061336
ATPase GET3 [OS=Homo sapiens]	1.222	0.020442915
Autophagy protein 5 [OS=Homo sapiens]	1.222	0.521260214
Galactosylgalactosylxylosylprotein 3-beta-glucuronosyltransferase 3 [OS=Hom	1.222	0.63949988
Selenocysteine lyase [OS=Homo sapiens]	1.222	0.129835874
IgG receptor FcRn large subunit p51 [OS=Homo sapiens]	1.222	0.442125793
Glutathione S-transferase kappa 1 [OS=Homo sapiens]	1.223	0.007967211
TRIO and F-actin-binding protein [OS=Homo sapiens]	1.223	0.05928895
TP53-regulated inhibitor of apoptosis 1 [OS=Homo sapiens]	1.223	0.065284344
Complement C1q tumor necrosis factor-related protein 3 [OS=Homo sapiens]	1.223	0.902931059
Thiosulfate:glutathione sulfurtransferase [OS=Homo sapiens]	1.223	0.980280306
Atypical kinase COQ8B, mitochondrial [OS=Homo sapiens]	1.224	0.00089331
FAD synthase [OS=Homo sapiens]	1.224	0.035709766
PIH1 domain-containing protein 1 [OS=Homo sapiens]	1.224	0.440161895
Ubiquitin-like protein 7 [OS=Homo sapiens]	1.224	0.703955957
Nuclear receptor-binding protein 2 [OS=Homo sapiens]	1.224	0.1541147
ELMO domain-containing protein 2 [OS=Homo sapiens]	1.224	0.993343739
Isoleucine--tRNA ligase, mitochondrial [OS=Homo sapiens]	1.225	0.017343697
Glutaryl-CoA dehydrogenase, mitochondrial [OS=Homo sapiens]	1.225	0.012806074
Cytochrome c oxidase assembly factor 4 homolog, mitochondrial [OS=Homo sa	1.225	0.047109859
Nuclear distribution protein nudE homolog 1 [OS=Homo sapiens]	1.225	0.008145116
CDK2-associated and cullin domain-containing protein 1 [OS=Homo sapiens]	1.225	0.013206548
Signal peptide peptidase-like 2B [OS=Homo sapiens]	1.225	0.050555024
Protein MANBAL [OS=Homo sapiens]	1.225	0.08695006
N-alpha-acetyltransferase 16, NatA auxiliary subunit [OS=Homo sapiens]	1.226	0.015462275
MAP/microtubule affinity-regulating kinase 3 [OS=Homo sapiens]	1.226	0.02746193
Glia maturation factor beta [OS=Homo sapiens]	1.226	0.004775879
N-acetylgalactosamine kinase [OS=Homo sapiens]	1.226	0.007263829
Mitogen-activated protein kinase 8 [OS=Homo sapiens]	1.226	0.003116257
Calmodulin-regulated spectrin-associated protein 2 [OS=Homo sapiens]	1.226	0.094748988
Polyphosphoinositide phosphatase [OS=Homo sapiens]	1.226	0.069738254
Protoporphyrinogen oxidase [OS=Homo sapiens]	1.226	0.037479656
Trafficking protein particle complex subunit 11 [OS=Homo sapiens]	1.226	0.067011092
NF-kappa-B inhibitor epsilon [OS=Homo sapiens]	1.226	0.059714521
WASH complex subunit 5 [OS=Homo sapiens]	1.227	0.005763665
Rho guanine nucleotide exchange factor 7 [OS=Homo sapiens]	1.227	0.015692285
Ras-related protein Rab-5B [OS=Homo sapiens]	1.227	0.008738211
Plasma alpha-L-fucosidase [OS=Homo sapiens]	1.227	0.143318001
Nuclear cap-binding protein subunit 2 [OS=Homo sapiens]	1.227	0.256636084
BAG family molecular chaperone regulator 1 [OS=Homo sapiens]	1.227	0.19783229
Peroxisomal leader peptide-processing protease [OS=Homo sapiens]	1.227	0.731965511
2-Hydroxyacid oxidase 1 [OS=Homo sapiens]	1.227	1
Ribosomal protein S6 kinase alpha-3 [OS=Homo sapiens]	1.228	0.000235807

Procathepsin L [OS=Homo sapiens]	1.228	0.001133065
Selenocysteine insertion sequence-binding protein 2-like [OS=Homo sapiens]	1.228	0.001578637
Patatin-like phospholipase domain-containing protein 4 [OS=Homo sapiens]	1.228	0.739887758
Protein NCBP2AS2 [OS=Homo sapiens]	1.228	0.983191959
Uveal autoantigen with coiled-coil domains and ankyrin repeats [OS=Homo sapi	1.229	0.034419553
Flavin reductase (NADPH) [OS=Homo sapiens]	1.23	0.000161887
ADP-ribosylation factor-like protein 8A [OS=Homo sapiens]	1.23	0.37735875
m7GpppX diphosphatase [OS=Homo sapiens]	1.23	0.059019664
Serine/threonine-protein kinase WNK3 [OS=Homo sapiens]	1.23	0.455701068
Coronin-7 [OS=Homo sapiens]	1.23	0.019090355
LIM and SH3 domain protein 1 [OS=Homo sapiens]	1.231	0.000614288
Glycogen synthase kinase-3 beta [OS=Homo sapiens]	1.231	0.001477922
Golgi to ER traffic protein 4 homolog [OS=Homo sapiens]	1.231	0.014258587
5'-nucleotidase domain-containing protein 2 [OS=Homo sapiens]	1.231	0.066755495
Fructose-2,6-bisphosphatase TIGAR [OS=Homo sapiens]	1.231	0.011143146
Vacuolar protein sorting-associated protein 41 homolog [OS=Homo sapiens]	1.231	0.003877017
Sorting nexin-29 [OS=Homo sapiens]	1.231	0.79083609
Delta(3,5)-Delta(2,4)-dienoyl-CoA isomerase, mitochondrial [OS=Homo sapiens	1.232	0.00804237
Endoplasmic reticulum mannosyl-oligosaccharide 1,2-alpha-mannosidase [OS=	1.232	0.002088505
BSD domain-containing protein 1 [OS=Homo sapiens]	1.232	0.001932611
Valacyclovir hydrolase [OS=Homo sapiens]	1.232	0.013309135
FLYWCH family member 2 [OS=Homo sapiens]	1.232	0.021522728
Transcription factor E2F4 [OS=Homo sapiens]	1.232	0.25120806
Nardilysin [OS=Homo sapiens]	1.233	0.001685114
Acidic leucine-rich nuclear phosphoprotein 32 family member B [OS=Homo sap	1.233	0.604213563
Acyl-coenzyme A thioesterase 13 [OS=Homo sapiens]	1.233	0.002121604
RUN and FYVE domain-containing protein 2 [OS=Homo sapiens]	1.233	0.010007592
DCC-interacting protein 13-beta [OS=Homo sapiens]	1.233	0.223224035
Arylsulfatase B [OS=Homo sapiens]	1.233	0.007324999
Nuclear factor of activated T-cells, cytoplasmic 1 [OS=Homo sapiens]	1.233	0.038396788
Activity-regulated cytoskeleton-associated protein [OS=Homo sapiens]	1.233	0.922505759
AP-1 complex subunit gamma-1 [OS=Homo sapiens]	1.234	0.003375794
Cystathionine gamma-lyase [OS=Homo sapiens]	1.234	0.000631313
GPI-anchor transamidase [OS=Homo sapiens]	1.234	0.811258585
Eukaryotic translation initiation factor 2-alpha kinase 1 [OS=Homo sapiens]	1.234	0.278314544
Small EDRK-rich factor 1 [OS=Homo sapiens]	1.234	0.253074398
Arf-GAP with SH3 domain, ANK repeat and PH domain-containing protein 1 [OS=	1.235	0.006292841
Catechol O-methyltransferase [OS=Homo sapiens]	1.235	0.015041599
ADP-ribosylation factor-like protein 1 [OS=Homo sapiens]	1.235	0.018221687
Divergent protein kinase domain 2A [OS=Homo sapiens]	1.235	0.873314186
Desumoylating isopeptidase 1 [OS=Homo sapiens]	1.235	0.333170841
Serine/threonine-protein kinase 24 [OS=Homo sapiens]	1.236	0.033442509
Vacuolar-sorting protein SNF8 [OS=Homo sapiens]	1.236	0.0545747
Lysosome-associated membrane glycoprotein 2 [OS=Homo sapiens]	1.236	0.062427367
Ubiquitin-fold modifier 1 [OS=Homo sapiens]	1.236	0.013741487
Protein disulfide-isomerase A4 [OS=Homo sapiens]	1.237	0.000582743
Vigilin [OS=Homo sapiens]	1.237	0.002106865
Glycine cleavage system H protein, mitochondrial [OS=Homo sapiens]	1.237	0.002866747
Cation-dependent mannose-6-phosphate receptor [OS=Homo sapiens]	1.237	0.000179805
Rho guanine nucleotide exchange factor 10 [OS=Homo sapiens]	1.237	0.003147945
Protein transport protein Sec61 subunit beta [OS=Homo sapiens]	1.237	0.483590799
Protein MMP24OS [OS=Homo sapiens]	1.237	0.075605257
Vacuolar protein sorting-associated protein 11 homolog [OS=Homo sapiens]	1.237	0.006962056
Xaa-Pro dipeptidase [OS=Homo sapiens]	1.238	0.014001662
Myotubularin-related protein 5 [OS=Homo sapiens]	1.238	0.007159424
Cyclin-dependent kinase 4 [OS=Homo sapiens]	1.238	0.137102736
Tumor necrosis factor receptor superfamily member 10A [OS=Homo sapiens]	1.238	0.546634839
Peptidyl-prolyl cis-trans isomerase FKBP3 [OS=Homo sapiens]	1.239	0.00207534
Uroporphyrinogen decarboxylase [OS=Homo sapiens]	1.239	0.824016081
Ras-related protein Rab-43 [OS=Homo sapiens]	1.239	0.267453545
40-kDa huntingtin-associated protein [OS=Homo sapiens]	1.239	0.005977929
Phosphatidylinositol 4-kinase type 2-beta [OS=Homo sapiens]	1.239	0.775796402
Protein disulfide-isomerase A3 [OS=Homo sapiens]	1.24	0.000703017
Malonate--CoA ligase ACSF3, mitochondrial [OS=Homo sapiens]	1.24	0.003510238
Cyclin-dependent kinase 5 [OS=Homo sapiens]	1.24	0.162055632
Putative peptidyl-tRNA hydrolase PTRHD1 [OS=Homo sapiens]	1.24	0.026681881
Hamartin [OS=Homo sapiens]	1.24	0.051089582
Tumor suppressor candidate 2 [OS=Homo sapiens]	1.24	0.207996304
Protein DDI1 homolog 2 [OS=Homo sapiens]	1.241	0.002745902
Tripartite motif-containing protein 3 [OS=Homo sapiens]	1.241	0.003518611
Glycylpeptide N-tetradecanoyltransferase 2 [OS=Homo sapiens]	1.242	0.001237135
GRIP1-associated protein 1 [OS=Homo sapiens]	1.242	0.009259479
Serine/threonine-protein kinase 4 [OS=Homo sapiens]	1.242	0.000718353
PDZ domain-containing protein 8 [OS=Homo sapiens]	1.242	0.010154581
Thymosin beta-10 [OS=Homo sapiens]	1.242	0.362036348

UDP-glucose:glycoprotein glucosyltransferase 1 [OS=Homo sapiens]	1.243	0.023022656
Profilin-2 [OS=Homo sapiens]	1.243	0.01096887
Mothers against decapentaplegic homolog 3 [OS=Homo sapiens]	1.243	0.048780172
Phosphatidylinositol 3,4,5-trisphosphate 5-phosphatase 2 [OS=Homo sapiens]	1.243	0.002577062
Trafficking protein particle complex subunit 4 [OS=Homo sapiens]	1.243	0.018992188
Epimerase family protein SDR39U1 [OS=Homo sapiens]	1.243	0.073096947
3-ketoacyl-CoA thiolase, mitochondrial [OS=Homo sapiens]	1.244	0.000772417
Transmembrane emp24 domain-containing protein 4 [OS=Homo sapiens]	1.244	0.128245233
SH3 domain-binding glutamic acid-rich-like protein 3 [OS=Homo sapiens]	1.244	0.000462123
E3 ubiquitin-protein ligase RNF25 [OS=Homo sapiens]	1.244	0.008757117
ADP-ribosylation factor-like protein 10 [OS=Homo sapiens]	1.244	0.076081706
Lysosomal alpha-glucosidase [OS=Homo sapiens]	1.245	0.001182409
3-mercaptopyruvate sulfurtransferase [OS=Homo sapiens]	1.245	0.031400077
Transmembrane protein 245 [OS=Homo sapiens]	1.245	0.032483542
Capping protein, Arp2/3 and myosin-I linker protein 3 [OS=Homo sapiens]	1.245	0.079775503
NADPH--cytochrome P450 reductase [OS=Homo sapiens]	1.246	0.066073409
FAD-linked sulfhydryl oxidase ALR [OS=Homo sapiens]	1.246	0.196820562
HIG1 domain family member 2A, mitochondrial [OS=Homo sapiens]	1.246	0.999975146
Gamma-interferon-inducible lysosomal thiol reductase [OS=Homo sapiens]	1.247	0.03691931
Haloacid dehalogenase-like hydrolase domain-containing protein 3 [OS=Homo s	1.247	0.01304133
Alpha-2-antiplasmin [OS=Homo sapiens]	1.247	0.425391756
Sodium-dependent phosphate transporter 2 [OS=Homo sapiens]	1.247	0.500204886
1,25-dihydroxyvitamin D(3) 24-hydroxylase, mitochondrial [OS=Homo sapiens]	1.247	0.855936644
Palmitoyl-protein thioesterase 1 [OS=Homo sapiens]	1.248	0.076403918
Huntingtin [OS=Homo sapiens]	1.248	0.017221271
Ubiquitin-conjugating enzyme E2 variant 3 [OS=Homo sapiens]	1.248	0.003953017
Ragulator complex protein LAMTOR1 [OS=Homo sapiens]	1.248	0.355390316
Puromycin-sensitive aminopeptidase [OS=Homo sapiens]	1.249	0.001427278
Glycogen debranching enzyme [OS=Homo sapiens]	1.249	0.008770098
Ubiquitin conjugation factor E4 A [OS=Homo sapiens]	1.249	0.03936774
Stromal membrane-associated protein 1 [OS=Homo sapiens]	1.249	0.010607763
Vacuolar protein-sorting-associated protein 25 [OS=Homo sapiens]	1.249	0.098897407
Protein VAC14 homolog [OS=Homo sapiens]	1.25	0.398316542
GDP-fucose protein O-fucosyltransferase 2 [OS=Homo sapiens]	1.25	0.002296049
Golgi membrane protein 1 [OS=Homo sapiens]	1.25	0.360933917
Sorting nexin-13 [OS=Homo sapiens]	1.25	0.242943427
Smoothened homolog [OS=Homo sapiens]	1.25	0.999996427
Heparin cofactor 2 [OS=Homo sapiens]	1.25	0.625956973
Sialidase-1 [OS=Homo sapiens]	1.251	0.326097583
Tyrosine-protein phosphatase non-receptor type 12 [OS=Homo sapiens]	1.251	0.008802901
Echinoderm microtubule-associated protein-like 1 [OS=Homo sapiens]	1.251	0.000618857
Cartilage-associated protein [OS=Homo sapiens]	1.251	4.22885E-05
Serine/threonine-protein phosphatase 4 regulatory subunit 4 [OS=Homo sapiens	1.251	0.052787367
Lysophospholipase-like protein 1 [OS=Homo sapiens]	1.251	0.003446467
Actin nucleation-promoting factor WASL [OS=Homo sapiens]	1.252	0.031587585
Ester hydrolase C11orf54 [OS=Homo sapiens]	1.252	0.352758853
Neuroblastoma suppressor of tumorigenicity 1 [OS=Homo sapiens]	1.252	0.222738653
Kininogen-1 [OS=Homo sapiens]	1.252	0.14873851
Phosphatidylinositol 4,5-bisphosphate 3-kinase catalytic subunit alpha isoform	1.252	0.072647262
Serine/threonine-protein phosphatase 2A 65 kDa regulatory subunit A beta isofo	1.253	0.005077936
E3 ubiquitin-protein ligase RNF114 [OS=Homo sapiens]	1.253	0.004108474
Ganglioside GM2 activator [OS=Homo sapiens]	1.253	0.026646852
Target of rapamycin complex 2 subunit MAPKAP1 [OS=Homo sapiens]	1.253	0.001427906
Transmembrane protein 106B [OS=Homo sapiens]	1.253	0.34607189
Isochorismatase domain-containing protein 1 [OS=Homo sapiens]	1.253	0.229682427
Peptidyl-prolyl cis-trans isomerase FKBP10 [OS=Homo sapiens]	1.254	0.000347189
Vesicle-trafficking protein SEC22b [OS=Homo sapiens]	1.254	0.001432143
Mitochondrial enolase superfamily member 1 [OS=Homo sapiens]	1.254	0.009671559
Protein YIPF4 [OS=Homo sapiens]	1.254	0.294426518
Negative regulator of P-body association [OS=Homo sapiens]	1.254	0.013214718
Ubiquitin conjugation factor E4 B [OS=Homo sapiens]	1.255	0.049906781
Huntingtin-interacting protein 1-related protein [OS=Homo sapiens]	1.255	0.004799541
Sorting nexin-4 [OS=Homo sapiens]	1.255	0.003495145
Endoplasmic reticulum aminopeptidase 2 [OS=Homo sapiens]	1.255	0.008711591
Alpha-1,3/1,6-mannosyltransferase ALG2 [OS=Homo sapiens]	1.255	0.278467714
Galectin-related protein [OS=Homo sapiens]	1.255	0.231884581
Adenine phosphoribosyltransferase [OS=Homo sapiens]	1.256	0.163184263
Translation machinery-associated protein 7 [OS=Homo sapiens]	1.256	0.059100058
Lethal(2) giant larvae protein homolog 1 [OS=Homo sapiens]	1.256	5.05428E-05
Charged multivesicular body protein 1a [OS=Homo sapiens]	1.256	0.000447623
Medium-chain acyl-CoA ligase ACSF2, mitochondrial [OS=Homo sapiens]	1.256	0.004718666
Laminin subunit beta-1 [OS=Homo sapiens]	1.256	0.001001992
1-phosphatidylinositol 4,5-bisphosphate phosphodiesterase eta-1 [OS=Homo s	1.256	0.376368779
Ral GTPase-activating protein subunit beta [OS=Homo sapiens]	1.258	0.0241994
Glutamine-dependent NAD(+) synthetase [OS=Homo sapiens]	1.258	0.006430791

HAUS augmin-like complex subunit 2 [OS=Homo sapiens]	1.258	0.583975541
GDH/6PGL endoplasmic bifunctional protein [OS=Homo sapiens]	1.258	0.048323448
PAN2-PAN3 deadenylation complex subunit PAN3 [OS=Homo sapiens]	1.258	0.976783074
Legumain [OS=Homo sapiens]	1.259	0.002188557
Serine protease HTRA1 [OS=Homo sapiens]	1.259	0.018424834
DmX-like protein 2 [OS=Homo sapiens]	1.259	0.000965883
Glucocorticoid-induced transcript 1 protein [OS=Homo sapiens]	1.259	0.036693262
SLAIN motif-containing protein 2 [OS=Homo sapiens]	1.259	0.963734011
C-reactive protein [OS=Homo sapiens]	1.259	0.236229962
DnaJ homolog subfamily C member 13 [OS=Homo sapiens]	1.26	0.000429151
Secernin-1 [OS=Homo sapiens]	1.26	0.023948282
Ubiquitin domain-containing protein UBFD1 [OS=Homo sapiens]	1.26	0.004889114
Spermatogenesis-defective protein 39 homolog [OS=Homo sapiens]	1.26	0.008592576
Ral GTPase-activating protein subunit alpha-1 [OS=Homo sapiens]	1.26	0.001818649
Alpha-N-acetylgalactosaminidase [OS=Homo sapiens]	1.26	0.014607306
Ribosomal protein S6 kinase alpha-4 [OS=Homo sapiens]	1.26	0.001043568
NF-kappa-B inhibitor-interacting Ras-like protein 2 [OS=Homo sapiens]	1.26	0.103360129
Multivesicular body subunit 12A [OS=Homo sapiens]	1.26	0.177392746
Glutamine--fructose-6-phosphate aminotransferase [isomerizing] 1 [OS=Homo	1.261	4.26066E-05
Y-box-binding protein 3 [OS=Homo sapiens]	1.261	0.002509763
E3 ubiquitin-protein ligase SMURF2 [OS=Homo sapiens]	1.261	0.749287088
Protein archease [OS=Homo sapiens]	1.261	0.031175731
Rab proteins geranylgeranyltransferase component A 2 [OS=Homo sapiens]	1.261	0.976936421
Lipoyltransferase 1, mitochondrial [OS=Homo sapiens]	1.261	0.257738943
Endoplasmic reticulum aminopeptidase 1 [OS=Homo sapiens]	1.262	0.01592245
Glycine amidinotransferase, mitochondrial [OS=Homo sapiens]	1.262	0.00080318
E3 ubiquitin-protein ligase RNF123 [OS=Homo sapiens]	1.262	0.019826308
Fibronectin [OS=Homo sapiens]	1.262	0.393362159
CD276 antigen [OS=Homo sapiens]	1.262	0.007015034
Microtubule-associated proteins 1A/1B light chain 3 beta 2 [OS=Homo sapiens]	1.262	0.003930818
Membrane cofactor protein [OS=Homo sapiens]	1.262	0.018176523
WD repeat-containing protein 47 [OS=Homo sapiens]	1.262	0.838997606
Syntaxin-6 [OS=Homo sapiens]	1.263	0.004910075
E3 ubiquitin-protein ligase rififylin [OS=Homo sapiens]	1.263	0.91220998
Golgin subfamily A member 5 [OS=Homo sapiens]	1.264	0.001369492
eIF-2-alpha kinase GCN2 [OS=Homo sapiens]	1.264	0.000290228
Rap1 GTPase-activating protein 2 [OS=Homo sapiens]	1.264	0.00014886
Eukaryotic translation initiation factor 4 gamma 3 [OS=Homo sapiens]	1.265	0.002110139
5'-3' exonuclease PLD3 [OS=Homo sapiens]	1.265	0.03103388
tRNA-dihydrouridine(20) synthase [NAD(P)+]-like [OS=Homo sapiens]	1.265	0.008385011
TBC1 domain family member 2B [OS=Homo sapiens]	1.265	0.686223866
Haloacid dehalogenase-like hydrolase domain-containing protein 2 [OS=Homo s	1.265	0.783773651
Epoxide hydrolase 1 [OS=Homo sapiens]	1.266	0.002902835
Secernin-2 [OS=Homo sapiens]	1.266	0.004580317
5'-deoxynucleotidase HDDC2 [OS=Homo sapiens]	1.266	0.00178293
Histone lysine demethylase PHF8 [OS=Homo sapiens]	1.266	0.911097645
Microtubule-associated tumor suppressor 1 [OS=Homo sapiens]	1.266	0.269045702
Dipeptidyl peptidase 8 [OS=Homo sapiens]	1.267	0.44236598
Poly(A) polymerase alpha [OS=Homo sapiens]	1.268	0.00060444
Integrin beta-5 [OS=Homo sapiens]	1.268	0.002623671
Kelch repeat and BTB domain-containing protein 6 [OS=Homo sapiens]	1.268	0.002567224
Aspartyl/asparaginyl beta-hydroxylase [OS=Homo sapiens]	1.268	0.006174325
WW domain-containing transcription regulator protein 1 [OS=Homo sapiens]	1.268	0.008824682
Phosphatidylserine decarboxylase proenzyme, mitochondrial [OS=Homo sapier	1.268	0.003776839
DAZ-associated protein 2 [OS=Homo sapiens]	1.268	0.060619008
Fibrinogen beta chain [OS=Homo sapiens]	1.268	0.217807168
Hemoglobin subunit delta [OS=Homo sapiens]	1.268	0.549275184
Aldehyde dehydrogenase X, mitochondrial [OS=Homo sapiens]	1.269	0.01724059
Vesicular integral-membrane protein VIP36 [OS=Homo sapiens]	1.269	0.002819412
Copine-1 [OS=Homo sapiens]	1.269	0.000697403
NF-kappa-B inhibitor-interacting Ras-like protein 1 [OS=Homo sapiens]	1.269	0.000443884
GTP-binding protein 2 [OS=Homo sapiens]	1.269	0.238648652
Nucleotide exchange factor SIL1 [OS=Homo sapiens]	1.269	0.003696008
FGGY carbohydrate kinase domain-containing protein [OS=Homo sapiens]	1.269	0.04036677
Serine/threonine-protein kinase 19 [OS=Homo sapiens]	1.27	0.991584277
BTB/POZ domain-containing protein KCTD1 [OS=Homo sapiens]	1.27	0.121771609
Transmembrane emp24 domain-containing protein 1 [OS=Homo sapiens]	1.271	0.058892779
Transmembrane emp24 domain-containing protein 5 [OS=Homo sapiens]	1.271	0.212365156
Vacuolar protein sorting-associated protein 16 homolog [OS=Homo sapiens]	1.271	0.542285808
Adipocyte plasma membrane-associated protein [OS=Homo sapiens]	1.272	0.002040956
Mitogen-activated protein kinase kinase kinase kinase 4 [OS=Homo sapiens]	1.272	0.020094894
Phosphatidylinositol 5-phosphate 4-kinase type-2 alpha [OS=Homo sapiens]	1.272	0.000836103
Integral membrane protein 2C [OS=Homo sapiens]	1.272	0.003245683
Amyloid beta precursor protein binding family B member 1 [OS=Homo sapiens]	1.272	0.013292293
Transmembrane protein 248 [OS=Homo sapiens]	1.272	0.003235763

Vacuolar protein sorting-associated protein 33B [OS=Homo sapiens]	1.273	0.000262329
Actin-related protein 2/3 complex subunit 1A [OS=Homo sapiens]	1.273	0.000433931
SH3 domain-binding protein 5-like [OS=Homo sapiens]	1.273	0.346698489
Lysosomal acid phosphatase [OS=Homo sapiens]	1.274	0.014605533
Ectopic P granules protein 5 homolog [OS=Homo sapiens]	1.274	0.00762888
Lambda-crystallin homolog [OS=Homo sapiens]	1.274	0.001603139
RNA polymerase I-specific transcription initiation factor RRN3 [OS=Homo sapie	1.274	0.52563578
Ras GTPase-activating-like protein IQGAP2 [OS=Homo sapiens]	1.275	0.003052168
Midkine [OS=Homo sapiens]	1.275	0.512391387
Protein phosphatase 1 regulatory subunit 21 [OS=Homo sapiens]	1.275	0.004000376
Golgi apparatus membrane protein TVP23 homolog C [OS=Homo sapiens]	1.275	0.476769416
Gamma-glutamylcyclotransferase [OS=Homo sapiens]	1.276	4.29129E-05
Cytoplasmic tRNA 2-thiolation protein 2 [OS=Homo sapiens]	1.276	0.022986235
Protein FAN [OS=Homo sapiens]	1.276	0.004617604
Disintegrin and metalloproteinase domain-containing protein 10 [OS=Homo sap	1.277	0.000304364
Adenylate kinase 4, mitochondrial [OS=Homo sapiens]	1.277	1.93434E-05
Leucine-rich repeat-containing protein 57 [OS=Homo sapiens]	1.277	0.000235853
BET1 homolog [OS=Homo sapiens]	1.277	0.000636547
Protein transport protein Sec24C [OS=Homo sapiens]	1.278	0.003070691
DnaJ homolog subfamily C member 10 [OS=Homo sapiens]	1.278	0.052566596
Inositol polyphosphate 5-phosphatase OCRL [OS=Homo sapiens]	1.279	0.247663095
Ras-related protein Rab-39A [OS=Homo sapiens]	1.279	0.460601325
Small EDRK-rich factor 2 [OS=Homo sapiens]	1.279	0.997257995
Thymidine kinase 2, mitochondrial [OS=Homo sapiens]	1.279	0.953622947
Integrin alpha-V [OS=Homo sapiens]	1.28	0.000387332
C-type mannose receptor 2 [OS=Homo sapiens]	1.28	0.001468561
Spermine synthase [OS=Homo sapiens]	1.28	6.06692E-05
Reticulocalbin-1 [OS=Homo sapiens]	1.28	0.000752463
Pre-mRNA-processing factor 40 homolog B [OS=Homo sapiens]	1.28	0.272601527
Cytochrome c oxidase subunit 6B2 [OS=Homo sapiens]	1.28	0.981507715
Methionine-R-sulfoxide reductase B2, mitochondrial [OS=Homo sapiens]	1.28	0.928992721
Epidermal growth factor receptor [OS=Homo sapiens]	1.281	0.000335461
Complement C3 [OS=Homo sapiens]	1.281	0.273358539
Low-density lipoprotein receptor [OS=Homo sapiens]	1.281	0.058609915
Beta-galactosidase [OS=Homo sapiens]	1.281	0.000439287
Protein Aster-A [OS=Homo sapiens]	1.282	0.002499956
Stromal cell-derived factor 2 [OS=Homo sapiens]	1.282	0.441471903
Kremen protein 2 [OS=Homo sapiens]	1.282	0.733176879
Sorting nexin-30 [OS=Homo sapiens]	1.283	0.001746391
40S ribosomal protein S26 [OS=Homo sapiens]	1.283	0.194653185
Proline-serine-threonine phosphatase-interacting protein 2 [OS=Homo sapiens]	1.283	0.280716124
Endoplasmic reticulum-Golgi intermediate compartment protein 2 [OS=Homo s	1.283	0.003169933
Alpha-2-macroglobulin [OS=Homo sapiens]	1.284	0.57685852
ATP-dependent (S)-NAD(P)H-hydrate dehydratase [OS=Homo sapiens]	1.284	0.018431534
Histone-arginine methyltransferase CARM1 [OS=Homo sapiens]	1.285	0.004329478
Formylglycine-generating enzyme [OS=Homo sapiens]	1.285	0.000944246
Neuralized-like protein 4 [OS=Homo sapiens]	1.285	0.067908534
Ubiquitin thioesterase OTUB1 [OS=Homo sapiens]	1.286	0.005041233
Ras-related protein Rab-13 [OS=Homo sapiens]	1.286	0.00753529
Prolow-density lipoprotein receptor-related protein 1 [OS=Homo sapiens]	1.286	0.004370424
Peroxisomal carnitine O-octanoyltransferase [OS=Homo sapiens]	1.286	0.000747411
Beta-centractin [OS=Homo sapiens]	1.287	0.001075518
Transmembrane protein 181 [OS=Homo sapiens]	1.287	0.070735199
Phosphatidylinositol 3-kinase regulatory subunit beta [OS=Homo sapiens]	1.288	0.547463206
Malonyl-CoA decarboxylase, mitochondrial [OS=Homo sapiens]	1.288	0.054926532
Coiled-coil domain-containing protein 146 [OS=Homo sapiens]	1.288	0.95950716
Calpain-2 catalytic subunit [OS=Homo sapiens]	1.289	0.003931908
Protein FAM98A [OS=Homo sapiens]	1.289	0.00034651
Tissue alpha-L-fucosidase [OS=Homo sapiens]	1.289	0.000348348
Antithrombin-III [OS=Homo sapiens]	1.289	0.236264766
Agrin [OS=Homo sapiens]	1.289	0.960539504
Solute carrier family 2, facilitated glucose transporter member 8 [OS=Homo sap	1.289	0.510948568
Carbohydrate sulfotransferase 6 [OS=Homo sapiens]	1.289	0.06592891
Tropomyosin beta chain [OS=Homo sapiens]	1.29	0.002216466
Grancalcin [OS=Homo sapiens]	1.29	0.707342152
F-box only protein 22 [OS=Homo sapiens]	1.29	0.003020699
Lumican [OS=Homo sapiens]	1.29	0.462827783
Alpha-mannosidase 2C1 [OS=Homo sapiens]	1.29	0.853331956
Acyl-protein thioesterase 2 [OS=Homo sapiens]	1.291	0.004377965
Sorting nexin-12 [OS=Homo sapiens]	1.291	0.001599146
Matrix metalloproteinase-14 [OS=Homo sapiens]	1.291	0.008330138
Mannosyl-oligosaccharide glucosidase [OS=Homo sapiens]	1.292	0.002029533
Rapamycin-insensitive companion of mTOR [OS=Homo sapiens]	1.292	0.000428505
Cathepsin B [OS=Homo sapiens]	1.292	0.000250723
Coiled-coil domain-containing protein 175 [OS=Homo sapiens]	1.292	0.012230625

ERO1-like protein beta [OS=Homo sapiens]	1.293	0.000119472
Protein IMPACT [OS=Homo sapiens]	1.293	0.013319854
Kinesin-like protein KIF13A [OS=Homo sapiens]	1.293	0.027644946
Trinucleotide repeat-containing gene 6A protein [OS=Homo sapiens]	1.294	0.096764037
Adapter molecule crk [OS=Homo sapiens]	1.295	0.001420733
DnaJ homolog subfamily C member 16 [OS=Homo sapiens]	1.295	0.013029925
Cytochrome b-245 chaperone 1 [OS=Homo sapiens]	1.295	0.466905972
Alpha-fetoprotein [OS=Homo sapiens]	1.295	0.39548697
Caspase-3 [OS=Homo sapiens]	1.295	0.148253887
Succinate--hydroxymethylglutarate CoA-transferase [OS=Homo sapiens]	1.296	0.015174252
Phospholipase A2 group XV [OS=Homo sapiens]	1.296	0.001102367
Jerky protein homolog [OS=Homo sapiens]	1.296	0.009892921
Insulin-like growth factor 2 mRNA-binding protein 1 [OS=Homo sapiens]	1.297	1.05121E-06
Lysophosphatidylcholine acyltransferase 1 [OS=Homo sapiens]	1.297	0.000112863
Vitamin K-dependent gamma-carboxylase [OS=Homo sapiens]	1.297	0.184107805
Thioredoxin domain-containing protein 5 [OS=Homo sapiens]	1.298	0.021803042
Transcription initiation factor IIA subunit 2 [OS=Homo sapiens]	1.298	0.003994486
Eukaryotic translation initiation factor 4E-binding protein 2 [OS=Homo sapiens]	1.298	0.385587035
Acid ceramidase [OS=Homo sapiens]	1.298	0.002502723
NLR family member X1 [OS=Homo sapiens]	1.298	0.99937368
Ras GTPase-activating protein-binding protein 2 [OS=Homo sapiens]	1.299	0.00013856
UBX domain-containing protein 6 [OS=Homo sapiens]	1.299	0.000993337
2-amino-3-ketobutyrate coenzyme A ligase, mitochondrial [OS=Homo sapiens]	1.299	0.00454186
S-adenosylhomocysteine hydrolase-like protein 1 [OS=Homo sapiens]	1.3	1.36685E-05
Amyloid beta precursor like protein 2 [OS=Homo sapiens]	1.3	0.000161265
Kynurenine--oxoglutarate transaminase 1 [OS=Homo sapiens]	1.3	0.082456357
Twisted gastrulation protein homolog 1 [OS=Homo sapiens]	1.3	0.000386023
Zinc finger TRAF-type-containing protein 1 [OS=Homo sapiens]	1.3	0.206210973
A-kinase anchor protein 11 [OS=Homo sapiens]	1.3	0.056195858
Sorting nexin-7 [OS=Homo sapiens]	1.3	0.197359815
Thrombospondin-1 [OS=Homo sapiens]	1.301	0.08168055
Ubiquitin carboxyl-terminal hydrolase 33 [OS=Homo sapiens]	1.301	0.956046554
Phospholipid transfer protein [OS=Homo sapiens]	1.302	0.08499346
2-5A-dependent ribonuclease [OS=Homo sapiens]	1.302	0.61724265
Damage-control phosphatase ARMT1 [OS=Homo sapiens]	1.304	0.002653669
Lysosome-associated membrane glycoprotein 1 [OS=Homo sapiens]	1.304	0.010800609
Serum paraoxonase/arylesterase 2 [OS=Homo sapiens]	1.304	0.356037207
Voltage-dependent calcium channel subunit alpha-2/delta-1 [OS=Homo sapiens	1.304	0.290518554
Short-chain specific acyl-CoA dehydrogenase, mitochondrial [OS=Homo sapien	1.305	0.001066719
ADP-dependent glucokinase [OS=Homo sapiens]	1.305	0.014638248
Rho guanine nucleotide exchange factor 11 [OS=Homo sapiens]	1.305	0.266963548
Zinc finger protein 706 [OS=Homo sapiens]	1.305	1.13798E-05
Diablo IAP-binding mitochondrial protein [OS=Homo sapiens]	1.306	1.96559E-05
Torsin-1B [OS=Homo sapiens]	1.306	0.001848594
DnaJ homolog subfamily C member 14 [OS=Homo sapiens]	1.306	0.64769086
Protein phosphatase inhibitor 2 [OS=Homo sapiens]	1.307	0.000790368
Myristoylated alanine-rich C-kinase substrate [OS=Homo sapiens]	1.307	0.023742352
Uncharacterized protein KIAA1522 [OS=Homo sapiens]	1.307	0.000259534
Folliculin [OS=Homo sapiens]	1.307	0.592398049
Metallothionein-2 [OS=Homo sapiens]	1.308	0.496021849
Formin-binding protein 1-like [OS=Homo sapiens]	1.308	0.013158199
Peroxiredoxin-5, mitochondrial [OS=Homo sapiens]	1.309	0.000788502
ADP-ribosylation factor-binding protein GGA3 [OS=Homo sapiens]	1.309	0.00353748
CD320 antigen [OS=Homo sapiens]	1.309	0.004964309
Proteasome maturation protein [OS=Homo sapiens]	1.309	0.016124128
Spermatogenesis-associated protein 20 [OS=Homo sapiens]	1.309	0.109854429
Lipopolysaccharide-induced tumor necrosis factor-alpha factor [OS=Homo sapi	1.31	0.050466268
Single-stranded DNA-binding protein, mitochondrial [OS=Homo sapiens]	1.311	0.448141938
Glia maturation factor gamma [OS=Homo sapiens]	1.311	0.000232583
DNA excision repair protein ERCC-6 [OS=Homo sapiens]	1.311	0.996228702
Golgin subfamily A member 4 [OS=Homo sapiens]	1.312	0.001475872
Dual specificity mitogen-activated protein kinase kinase 4 [OS=Homo sapiens]	1.312	0.003776763
Lactotransferrin [OS=Homo sapiens]	1.312	0.188214247
CCN family member 2 [OS=Homo sapiens]	1.313	0.268764848
Reticulophagy regulator 3 [OS=Homo sapiens]	1.313	0.029782102
Protein rogdi homolog [OS=Homo sapiens]	1.313	0.04805286
Cation-independent mannose-6-phosphate receptor [OS=Homo sapiens]	1.314	0.002620538
DDB1- and CUL4-associated factor 1 [OS=Homo sapiens]	1.314	0.000423879
Lysosomal protective protein [OS=Homo sapiens]	1.314	0.024333657
Arf-GAP with SH3 domain, ANK repeat and PH domain-containing protein 2 [OS=	1.314	0.198525206
Alpha-1,3-mannosyl-glycoprotein 4-beta-N-acetylglucosaminyltransferase B [O	1.314	0.116987785
FUN14 domain-containing protein 2 [OS=Homo sapiens]	1.314	0.076276537
Poly [ADP-ribose] polymerase tankyrase-1 [OS=Homo sapiens]	1.314	0.745451115
Prolyl 4-hydroxylase subunit alpha-1 [OS=Homo sapiens]	1.315	4.20686E-06
Inactive tyrosine-protein kinase 7 [OS=Homo sapiens]	1.315	5.2713E-05

Collagen alpha-1(V) chain [OS=Homo sapiens]	1.315	0.031600681
F-box DNA helicase 1 [OS=Homo sapiens]	1.315	0.5180408
Selenide, water dikinase 1 [OS=Homo sapiens]	1.316	0.001045017
Acylpyruvase FAHD1, mitochondrial [OS=Homo sapiens]	1.316	0.018063752
Bleomycin hydrolase [OS=Homo sapiens]	1.316	2.09608E-05
Sodium/hydrogen exchanger 7 [OS=Homo sapiens]	1.316	0.310974014
Aldo-keto reductase family 1 member A1 [OS=Homo sapiens]	1.317	0.000893879
Copine-2 [OS=Homo sapiens]	1.317	0.008237755
Cartilage oligomeric matrix protein [OS=Homo sapiens]	1.317	0.17978044
Beta-1,3-galactosyltransferase 6 [OS=Homo sapiens]	1.317	1
DENN domain-containing protein 5B [OS=Homo sapiens]	1.319	0.008866097
Probable inactive tRNA-specific adenosine deaminase-like protein 3 [OS=Homo	1.32	0.777033631
DNA helicase B [OS=Homo sapiens]	1.32	0.002877384
NAD(P)H-hydrate epimerase [OS=Homo sapiens]	1.322	0.008238631
Receptor-type tyrosine-protein phosphatase eta [OS=Homo sapiens]	1.323	0.008943475
Glutamine amidotransferase-like class 1 domain-containing protein 1 [OS=Hom	1.323	0.006749563
Autophagy-related protein 13 [OS=Homo sapiens]	1.323	0.395685063
Serpin B9 [OS=Homo sapiens]	1.324	0.00210763
Nectin-2 [OS=Homo sapiens]	1.324	0.003747987
Nucleoside diphosphate kinase 3 [OS=Homo sapiens]	1.324	0.007301754
GA-binding protein subunit beta-1 [OS=Homo sapiens]	1.325	0.767994897
Sorting nexin-33 [OS=Homo sapiens]	1.325	0.094395048
Kinesin heavy chain isoform 5A [OS=Homo sapiens]	1.326	0.088657682
NPC intracellular cholesterol transporter 2 [OS=Homo sapiens]	1.326	0.005083455
Mapk-regulated corepressor-interacting protein 1 [OS=Homo sapiens]	1.326	6.45044E-05
Coiled-coil domain-containing protein 28A [OS=Homo sapiens]	1.326	0.009769529
Mitogen-activated protein kinase kinase kinase 2 [OS=Homo sapiens]	1.326	0.862104081
Copper transport protein ATOX1 [OS=Homo sapiens]	1.326	0.002101423
Differentially expressed in FDCP 8 homolog [OS=Homo sapiens]	1.326	0.145493461
Pyruvate dehydrogenase phosphatase regulatory subunit, mitochondrial [OS=Hc	1.327	0.000731839
Endoplasmic reticulum-Golgi intermediate compartment protein 3 [OS=Homo s	1.327	0.005550821
ATP synthase mitochondrial F1 complex assembly factor 1 [OS=Homo sapiens]	1.327	0.406573456
Homologous recombination OB-fold protein [OS=Homo sapiens]	1.327	0.317362417
Fibulin-1 [OS=Homo sapiens]	1.328	0.124570652
Interferon regulatory factor 3 [OS=Homo sapiens]	1.328	0.034670794
Galactocerebrosidase [OS=Homo sapiens]	1.329	0.068506118
Protein transport protein Sec24A [OS=Homo sapiens]	1.33	0.00791007
Galactose-1-phosphate uridylyltransferase [OS=Homo sapiens]	1.33	0.003443682
Deleted in malignant brain tumors 1 protein [OS=Homo sapiens]	1.33	0.588656646
Receptor-type tyrosine-protein phosphatase S [OS=Homo sapiens]	1.33	0.389122955
Astrocytic phosphoprotein PEA-15 [OS=Homo sapiens]	1.331	0.000142697
Sorting nexin-8 [OS=Homo sapiens]	1.332	0.00015283
Protein wntless homolog [OS=Homo sapiens]	1.332	0.003641128
Homeobox protein TGIF1 [OS=Homo sapiens]	1.332	0.001480443
Fermitin family homolog 2 [OS=Homo sapiens]	1.333	0.000104808
Melanoma-associated antigen D2 [OS=Homo sapiens]	1.333	0.000176707
Basigin [OS=Homo sapiens]	1.334	0.03727712
Coiled-coil domain-containing protein 186 [OS=Homo sapiens]	1.334	0.009050415
Fibrinogen gamma chain [OS=Homo sapiens]	1.334	0.99163459
Serine/threonine-protein phosphatase 2A 56 kDa regulatory subunit alpha isofor	1.335	0.001556354
Plexin-A1 [OS=Homo sapiens]	1.335	0.00921211
Death-associated protein 1 [OS=Homo sapiens]	1.335	0.002394427
N-acetyl-D-glucosamine kinase [OS=Homo sapiens]	1.336	0.000916017
Peptidyl-prolyl cis-trans isomerase FKBP1A [OS=Homo sapiens]	1.336	0.008366442
Desmin [OS=Homo sapiens]	1.336	0.220881148
Phospholipid-transporting ATPase VA [OS=Homo sapiens]	1.336	0.99914313
Phospholipase B-like 1 [OS=Homo sapiens]	1.337	6.78629E-05
Adenosine deaminase [OS=Homo sapiens]	1.337	0.015495354
Endoplasmic reticulum resident protein 44 [OS=Homo sapiens]	1.337	8.99848E-05
Coiled-coil domain-containing protein 117 [OS=Homo sapiens]	1.337	0.138339862
Interferon alpha/beta receptor 1 [OS=Homo sapiens]	1.337	0.001991628
Nucleoside diphosphate kinase, mitochondrial [OS=Homo sapiens]	1.338	0.001701137
ATP synthase subunit s, mitochondrial [OS=Homo sapiens]	1.338	0.010360866
Protein FAM234B [OS=Homo sapiens]	1.338	0.008389565
Peroxisome proliferator-activated receptor delta [OS=Homo sapiens]	1.338	0.177487704
DDB1- and CUL4-associated factor 6 [OS=Homo sapiens]	1.339	0.011188099
Hydroxymethylglutaryl-CoA lyase, mitochondrial [OS=Homo sapiens]	1.339	0.002140787
Inactive tyrosine-protein kinase transmembrane receptor ROR1 [OS=Homo sapi	1.339	0.059361844
Signal transducer and activator of transcription 2 [OS=Homo sapiens]	1.34	0.00700327
Tyrosine-protein phosphatase non-receptor type 3 [OS=Homo sapiens]	1.34	0.65425738
Myotubularin-related protein 2 [OS=Homo sapiens]	1.341	0.016639901
Heme oxygenase 1 [OS=Homo sapiens]	1.342	0.002876524
Bis(5'-adenosyl)-triphosphatase ENPP4 [OS=Homo sapiens]	1.342	0.002516354
UPF0729 protein C18orf32 [OS=Homo sapiens]	1.342	0.086310003
Transforming growth factor beta receptor type 3 [OS=Homo sapiens]	1.342	0.359581686

Sorting nexin-11 [OS=Homo sapiens]	1.343	0.124810931
Phospholysine phosphohistidine inorganic pyrophosphate phosphatase [OS=Hc	1.344	0.000755803
Ankyrin repeat and IBR domain-containing protein 1 [OS=Homo sapiens]	1.344	0.139017592
Lysosomal alpha-mannosidase [OS=Homo sapiens]	1.345	7.7027E-05
WD repeat and FYVE domain-containing protein 1 [OS=Homo sapiens]	1.345	0.135294297
Motile sperm domain-containing protein 2 [OS=Homo sapiens]	1.345	0.010809738
Collagen alpha-1(I) chain [OS=Homo sapiens]	1.345	0.212382708
G-protein-signaling modulator 1 [OS=Homo sapiens]	1.345	0.019519454
Beta-mannosidase [OS=Homo sapiens]	1.345	0.373246989
Lysosomal amino acid transporter 1 homolog [OS=Homo sapiens]	1.345	0.405113344
RNA-binding protein with multiple splicing 2 [OS=Homo sapiens]	1.345	0.916281175
Protein FAM118B [OS=Homo sapiens]	1.347	0.010861684
Tumor protein p63-regulated gene 1-like protein [OS=Homo sapiens]	1.347	0.017306618
Transferrin receptor protein 1 [OS=Homo sapiens]	1.348	0.000166784
TATA box-binding protein-like 1 [OS=Homo sapiens]	1.348	0.002167517
V-type proton ATPase subunit S1 [OS=Homo sapiens]	1.348	0.000868207
Apolipoprotein C-III [OS=Homo sapiens]	1.348	0.833837958
Cathepsin D [OS=Homo sapiens]	1.349	0.003555056
Regucalcin [OS=Homo sapiens]	1.349	0.786162149
Heat shock protein beta-8 [OS=Homo sapiens]	1.349	0.305562806
Plastin-1 [OS=Homo sapiens]	1.35	4.49767E-06
Vacuolar protein sorting-associated protein 8 homolog [OS=Homo sapiens]	1.35	0.620105156
Transmembrane protein 184B [OS=Homo sapiens]	1.35	0.005033985
Albumin [OS=Homo sapiens]	1.351	0.305391418
Serotransferrin [OS=Homo sapiens]	1.351	0.495767534
Synaptic vesicle membrane protein VAT-1 homolog [OS=Homo sapiens]	1.352	2.08145E-05
40S ribosomal protein S29 [OS=Homo sapiens]	1.352	0.376496137
Proton-transporting V-type ATPase complex assembly regulator TMEM9 [OS=Ho	1.352	0.01380438
Sugar phosphate exchanger 3 [OS=Homo sapiens]	1.352	0.057745776
MHC class I polypeptide-related sequence A [OS=Homo sapiens]	1.353	0.182177775
Isovaleryl-CoA dehydrogenase, mitochondrial [OS=Homo sapiens]	1.354	0.000287549
Probable serine carboxypeptidase CPVL [OS=Homo sapiens]	1.354	0.435928941
Superoxide dismutase [Cu-Zn] [OS=Homo sapiens]	1.355	0.11896424
DIS3-like exonuclease 1 [OS=Homo sapiens]	1.355	0.043131182
Angiopoietin-related protein 4 [OS=Homo sapiens]	1.355	0.026592578
Nucleus accumbens-associated protein 1 [OS=Homo sapiens]	1.356	0.033445718
Protein phosphatase 1 regulatory subunit 14C [OS=Homo sapiens]	1.358	0.842151948
EF-hand calcium-binding domain-containing protein 14 [OS=Homo sapiens]	1.358	0.048056758
TBC1 domain family member 17 [OS=Homo sapiens]	1.359	0.012483218
Sorting nexin-9 [OS=Homo sapiens]	1.361	0.002072033
Jupiter microtubule associated homolog 1 [OS=Homo sapiens]	1.361	6.73295E-05
Protein phosphatase 1 regulatory subunit 12C [OS=Homo sapiens]	1.361	0.001784096
Ubiquitin carboxyl-terminal hydrolase 32 [OS=Homo sapiens]	1.361	0.172756753
Alpha-1B-glycoprotein [OS=Homo sapiens]	1.362	0.244206337
DNA-directed RNA polymerases I and III subunit RPAC2 [OS=Homo sapiens]	1.363	0.764186103
Immunoglobulin lambda-1 light chain [OS=Homo sapiens]	1.363	0.350572225
10 kDa heat shock protein, mitochondrial [OS=Homo sapiens]	1.364	0.00056562
Pleckstrin homology domain-containing family M member 1 [OS=Homo sapiens]	1.365	0.59545055
Cyclin-dependent kinase 17 [OS=Homo sapiens]	1.366	0.797628789
Bax inhibitor 1 [OS=Homo sapiens]	1.366	0.721299192
C-C motif chemokine 2 [OS=Homo sapiens]	1.367	0.000148443
14 kDa phosphohistidine phosphatase [OS=Homo sapiens]	1.368	0.000221182
Protein transport protein Sec23A [OS=Homo sapiens]	1.369	0.000596182
Plasminogen [OS=Homo sapiens]	1.369	0.221566716
Transmembrane protein 65 [OS=Homo sapiens]	1.369	0.152010436
Uncharacterized protein KIAA2012 [OS=Homo sapiens]	1.369	0.339583705
Alpha-2-HS-glycoprotein [OS=Homo sapiens]	1.37	0.34195112
Integral membrane protein 2B [OS=Homo sapiens]	1.37	0.00189668
Inter-alpha-trypsin inhibitor heavy chain H1 [OS=Homo sapiens]	1.37	0.166259398
Serpin H1 [OS=Homo sapiens]	1.371	0.002378494
Echinoderm microtubule-associated protein-like 4 [OS=Homo sapiens]	1.371	4.19298E-05
Inter-alpha-trypsin inhibitor heavy chain H2 [OS=Homo sapiens]	1.371	0.380330595
Ceramide transfer protein [OS=Homo sapiens]	1.372	0.000225263
Interleukin-13 receptor subunit alpha-1 [OS=Homo sapiens]	1.372	0.132896769
Golgin subfamily A member 1 [OS=Homo sapiens]	1.373	0.002768791
ER lumen protein-retaining receptor 2 [OS=Homo sapiens]	1.375	0.994222733
Calpain-15 [OS=Homo sapiens]	1.375	0.170347423
Prothrombin [OS=Homo sapiens]	1.377	0.103253354
Palmitoyltransferase ZDHHC3 [OS=Homo sapiens]	1.378	0.163038737
Anaphase-promoting complex subunit 10 [OS=Homo sapiens]	1.379	0.11508881
Glycerophosphodiester phosphodiesterase 1 [OS=Homo sapiens]	1.38	0.051034097
Junctional adhesion molecule C [OS=Homo sapiens]	1.381	5.49082E-05
Ubiquitin-associated and SH3 domain-containing protein B [OS=Homo sapiens]	1.381	0.498619876
Brefeldin A-inhibited guanine nucleotide-exchange protein 1 [OS=Homo sapiens	1.382	0.000255841
Synaptogyrin-3 [OS=Homo sapiens]	1.382	0.178771138

Sortilin [OS=Homo sapiens]	1.383	0.004173971
Pygopus homolog 2 [OS=Homo sapiens]	1.383	0.257170267
Trophoblast glycoprotein [OS=Homo sapiens]	1.383	0.005482236
2-oxoglutarate and iron-dependent oxygenase domain-containing protein 2 [OS=	1.383	0.1120626
Branched-chain-amino-acid aminotransferase, mitochondrial [OS=Homo sapie	1.384	0.00127608
Integrin alpha-6 [OS=Homo sapiens]	1.385	0.000300745
Tetranectin [OS=Homo sapiens]	1.387	0.081285887
Receptor-type tyrosine-protein phosphatase alpha [OS=Homo sapiens]	1.387	0.037363264
Late secretory pathway protein AVL9 homolog [OS=Homo sapiens]	1.388	0.016892008
Neurobeachin-like protein 2 [OS=Homo sapiens]	1.388	0.004063205
Protein TSSC4 [OS=Homo sapiens]	1.388	0.890028517
Vitamin D-binding protein [OS=Homo sapiens]	1.389	0.104747812
Microtubule-associated protein RP/EB family member 3 [OS=Homo sapiens]	1.389	0.133584316
Protein canopy homolog 4 [OS=Homo sapiens]	1.39	0.060520662
Vacuolar protein sorting-associated protein 54 [OS=Homo sapiens]	1.39	0.076465646
Dynamin-1 [OS=Homo sapiens]	1.391	0.000729307
PPP2R1A-PPP2R2A-interacting phosphatase regulator 1 [OS=Homo sapiens]	1.391	0.812289626
Nucleoside diphosphate phosphatase ENTPD5 [OS=Homo sapiens]	1.391	0.110322579
RISC-loading complex subunit TARBP2 [OS=Homo sapiens]	1.392	3.23473E-05
von Willebrand factor A domain-containing protein 5A [OS=Homo sapiens]	1.395	0.001161263
Actin remodeling regulator NHS [OS=Homo sapiens]	1.395	0.001790889
Pantetheinase [OS=Homo sapiens]	1.396	0.194693817
Homer protein homolog 1 [OS=Homo sapiens]	1.397	0.260811988
ADP-ribosylation factor-like protein 8B [OS=Homo sapiens]	1.398	0.00873501
Transmembrane protein 205 [OS=Homo sapiens]	1.398	0.116509925
Lysophosphatidic acid phosphatase type 6 [OS=Homo sapiens]	1.398	0.000920845
DNA topoisomerase I, mitochondrial [OS=Homo sapiens]	1.398	0.380411521
Autophagy-related protein 101 [OS=Homo sapiens]	1.398	0.050510841
Semaphorin-4C [OS=Homo sapiens]	1.398	0.01334418
Mannose-6-phosphate isomerase [OS=Homo sapiens]	1.399	0.033065805
Rhomboid domain-containing protein 3 [OS=Homo sapiens]	1.4	0.025221317
Calcium channel flower homolog [OS=Homo sapiens]	1.401	0.393545974
Copper chaperone for superoxide dismutase [OS=Homo sapiens]	1.402	3.44946E-05
Secretory carrier-associated membrane protein 1 [OS=Homo sapiens]	1.403	0.000122885
Amidophosphoribosyltransferase [OS=Homo sapiens]	1.403	5.62763E-05
Complement C4-A [OS=Homo sapiens]	1.403	0.269064028
Trafficking protein particle complex subunit 6A [OS=Homo sapiens]	1.404	0.047201946
Sex hormone-binding globulin [OS=Homo sapiens]	1.405	0.837863452
Inactive glycosyltransferase 25 family member 3 [OS=Homo sapiens]	1.406	0.005602124
Partitioning defective 6 homolog beta [OS=Homo sapiens]	1.408	0.000340515
Protein phosphatase 1H [OS=Homo sapiens]	1.408	0.540507967
Glucosamine-6-phosphate isomerase 1 [OS=Homo sapiens]	1.409	0.025506085
Dipeptidyl peptidase 2 [OS=Homo sapiens]	1.41	0.000308756
Diacylglycerol kinase zeta [OS=Homo sapiens]	1.41	0.029995566
F-BAR and double SH3 domains protein 1 [OS=Homo sapiens]	1.41	0.003334222
Contactin-associated protein 1 [OS=Homo sapiens]	1.411	6.04175E-05
Mitogen-activated protein kinase kinase kinase kinase 3 [OS=Homo sapiens]	1.411	0.001303157
D-2-hydroxyglutarate dehydrogenase, mitochondrial [OS=Homo sapiens]	1.412	0.00062283
Transcription factor 25 [OS=Homo sapiens]	1.412	0.065535367
Hepatocyte growth factor activator [OS=Homo sapiens]	1.415	0.065953679
Cyclin-Y-like protein 1 [OS=Homo sapiens]	1.415	0.440865264
ADP-ribosylation factor-like protein 5B [OS=Homo sapiens]	1.416	0.017624347
DDB1- and CUL4-associated factor 8 [OS=Homo sapiens]	1.419	0.010641088
NADP-dependent malic enzyme, mitochondrial [OS=Homo sapiens]	1.421	0.050772524
Run domain Beclin-1-interacting and cysteine-rich domain-containing protein [C	1.425	0.716727386
Protein O-glucosyltransferase 2 [OS=Homo sapiens]	1.427	0.000861497
Phospholipid scramblase 3 [OS=Homo sapiens]	1.427	0.383087374
Oncostatin-M-specific receptor subunit beta [OS=Homo sapiens]	1.429	0.007450709
Ceroid-lipofuscinosis neuronal protein 5 [OS=Homo sapiens]	1.43	0.000324995
Gamma-aminobutyric acid receptor-associated protein-like 1 [OS=Homo sapier	1.431	0.123911128
Disks large-associated protein 4 [OS=Homo sapiens]	1.432	0.001736464
Isobutyryl-CoA dehydrogenase, mitochondrial [OS=Homo sapiens]	1.434	0.08487243
Plasmanylethanolamine desaturase [OS=Homo sapiens]	1.435	0.000240147
Gelsolin [OS=Homo sapiens]	1.436	5.0078E-06
Alpha-(1,3)-fucosyltransferase 11 [OS=Homo sapiens]	1.436	0.000105214
N-acetylglucosamine-1-phosphodiester alpha-N-acetylglucosaminidase [OS=H	1.437	0.001219849
Trinucleotide repeat-containing gene 6B protein [OS=Homo sapiens]	1.439	1.74146E-05
GDP-L-fucose synthase [OS=Homo sapiens]	1.439	0.000113863
Urokinase plasminogen activator surface receptor [OS=Homo sapiens]	1.439	0.048725944
GTP-binding protein RAD [OS=Homo sapiens]	1.439	0.037678452
Carboxypeptidase D [OS=Homo sapiens]	1.441	0.00015015
Di-N-acetylchitobiase [OS=Homo sapiens]	1.442	0.000651826
Cortexin-1 [OS=Homo sapiens]	1.442	0.022326868
Inter-alpha-trypsin inhibitor heavy chain H4 [OS=Homo sapiens]	1.443	0.398459469
Serpin B7 [OS=Homo sapiens]	1.443	0.438938051

Sortilin-related receptor [OS=Homo sapiens]	1.445	0.000319164
Superoxide dismutase [Mn], mitochondrial [OS=Homo sapiens]	1.445	0.024144479
Selenoprotein F [OS=Homo sapiens]	1.448	5.64749E-05
Chitinase domain-containing protein 1 [OS=Homo sapiens]	1.449	0.000499968
4-trimethylaminobutyraldehyde dehydrogenase [OS=Homo sapiens]	1.45	4.47907E-06
Retinol-binding protein 4 [OS=Homo sapiens]	1.451	0.557354568
Sodium-dependent multivitamin transporter [OS=Homo sapiens]	1.452	0.05580479
Peptidyl-prolyl cis-trans isomerase FKBP9 [OS=Homo sapiens]	1.453	8.49804E-05
Metallothionein-1F [OS=Homo sapiens]	1.455	0.591904797
Thyroxine-binding globulin [OS=Homo sapiens]	1.457	0.215475414
EH domain-containing protein 1 [OS=Homo sapiens]	1.459	0.000148464
Phospholipase DDHD1 [OS=Homo sapiens]	1.459	0.007865904
Erythroid differentiation-related factor 1 [OS=Homo sapiens]	1.462	0.22653344
Protein spinster homolog 1 [OS=Homo sapiens]	1.462	0.109917168
Disintegrin and metalloproteinase domain-containing protein 17 [OS=Homo sap	1.463	7.37953E-06
F-BAR domain only protein 2 [OS=Homo sapiens]	1.464	0.000144396
Zinc finger FYVE domain-containing protein 9 [OS=Homo sapiens]	1.466	0.015532116
Protein disulfide isomerase CRELD1 [OS=Homo sapiens]	1.466	0.000347878
Protein FAM184B [OS=Homo sapiens]	1.466	0.107750559
Ras-related protein Rab-30 [OS=Homo sapiens]	1.469	0.03998976
Interleukin-6 receptor subunit beta [OS=Homo sapiens]	1.47	4.77625E-05
Renin receptor [OS=Homo sapiens]	1.474	0.01279874
Tripeptidyl-peptidase 1 [OS=Homo sapiens]	1.476	0.000240935
Podocalyxin [OS=Homo sapiens]	1.477	0.0013247
UDP-N-acetylglucosamine--dolichyl-phosphate N-acetylglucosaminephosphotr	1.48	0.683923468
Misshapen-like kinase 1 [OS=Homo sapiens]	1.481	3.13207E-07
Glucoside xylosyltransferase 1 [OS=Homo sapiens]	1.481	1.90889E-05
Centrosomal protein of 85 kDa-like [OS=Homo sapiens]	1.481	0.308592066
Acetyl-coenzyme A synthetase 2-like, mitochondrial [OS=Homo sapiens]	1.486	0.017014891
Beta-hexosaminidase subunit beta [OS=Homo sapiens]	1.489	2.33374E-05
Mitochondrial protein C2orf69 [OS=Homo sapiens]	1.493	0.019339293
Mitogen-activated protein kinase kinase kinase 19 [OS=Homo sapiens]	1.496	0.221088493
Synaptophysin [OS=Homo sapiens]	1.498	0.000219608
Calponin-2 [OS=Homo sapiens]	1.5	2.9612E-06
Tyrosine-protein phosphatase non-receptor type 9 [OS=Homo sapiens]	1.502	0.332999997
Insulin-like growth factor-binding protein 2 [OS=Homo sapiens]	1.507	0.002684373
Protein tweety homolog 3 [OS=Homo sapiens]	1.507	5.23738E-05
Activating molecule in BECN1-regulated autophagy protein 1 [OS=Homo sapiens	1.508	0.801278303
Collagen alpha-1(III) chain [OS=Homo sapiens]	1.509	0.277408153
Insulin-like growth factor-binding protein 4 [OS=Homo sapiens]	1.511	0.099034112
Protein argonaute-1 [OS=Homo sapiens]	1.512	1.42718E-05
P2X purinoceptor 4 [OS=Homo sapiens]	1.513	0.0378344
WD repeat and FYVE domain-containing protein 3 [OS=Homo sapiens]	1.514	0.381917131
N-acetylglucosamine-6-sulfatase [OS=Homo sapiens]	1.515	5.59818E-05
Lysosomal thioesterase PPT2 [OS=Homo sapiens]	1.515	0.011614725
Deoxyribonuclease-1-like 1 [OS=Homo sapiens]	1.516	0.183902833
Ral guanine nucleotide dissociation stimulator-like 2 [OS=Homo sapiens]	1.517	0.011940329
Prosaposin [OS=Homo sapiens]	1.518	8.18279E-07
Exostosin-2 [OS=Homo sapiens]	1.518	0.000100845
Eukaryotic translation initiation factor 5A-2 [OS=Homo sapiens]	1.519	2.00678E-05
BTB/POZ domain-containing adapter for CUL3-mediated RhoA degradation prot	1.521	0.515642875
Neuroplastin [OS=Homo sapiens]	1.522	0.00153893
Insulin-like growth factor II [OS=Homo sapiens]	1.522	0.00649553
Tyrosine-protein phosphatase non-receptor type substrate 1 [OS=Homo sapiens	1.525	0.03133148
Vesicle transport through interaction with t-SNAREs homolog 1B [OS=Homo sap	1.527	6.10345E-05
MHC class I polypeptide-related sequence B [OS=Homo sapiens]	1.529	0.001390119
Transforming growth factor beta-1 proprotein [OS=Homo sapiens]	1.531	0.00056233
Selenoprotein N [OS=Homo sapiens]	1.531	0.019748215
Glycogenin-1 [OS=Homo sapiens]	1.532	1.42666E-05
Ras-related protein Rab-4B [OS=Homo sapiens]	1.532	3.14077E-06
Prolyl 3-hydroxylase 3 [OS=Homo sapiens]	1.534	0.080486829
Protein transport protein Sec24D [OS=Homo sapiens]	1.536	0.00041365
Clusterin [OS=Homo sapiens]	1.537	3.23281E-07
Collagen alpha-2(VI) chain [OS=Homo sapiens]	1.537	9.06966E-07
Fibronectin type-III domain-containing protein 3A [OS=Homo sapiens]	1.542	0.003019142
Polyadenylate-binding protein-interacting protein 2 [OS=Homo sapiens]	1.543	0.000342526
ATP-binding cassette sub-family C member 5 [OS=Homo sapiens]	1.549	0.258013783
Lysyl oxidase homolog 2 [OS=Homo sapiens]	1.551	2.73337E-07
Beta-2-glycoprotein 1 [OS=Homo sapiens]	1.551	0.203281561
Protein FAM3C [OS=Homo sapiens]	1.552	0.011411604
Peptidyl-prolyl cis-trans isomerase F, mitochondrial [OS=Homo sapiens]	1.553	0.001024704
Laminin subunit gamma-1 [OS=Homo sapiens]	1.556	6.9174E-06
Signal recognition particle receptor subunit alpha [OS=Homo sapiens]	1.56	2.78663E-06
Progranulin [OS=Homo sapiens]	1.562	0.000131855
Citramalyl-CoA lyase, mitochondrial [OS=Homo sapiens]	1.562	0.474837629

Transmembrane prolyl 4-hydroxylase [OS=Homo sapiens]	1.564	0.041649537
TBC1 domain family member 8 [OS=Homo sapiens]	1.566	0.000818982
Protein disulfide-isomerase A5 [OS=Homo sapiens]	1.584	6.71633E-06
Cytosolic arginine sensor for mTORC1 subunit 2 [OS=Homo sapiens]	1.584	0.002482681
Insulin-like growth factor 2 mRNA-binding protein 3 [OS=Homo sapiens]	1.587	4.89616E-10
D-aminoacyl-tRNA deacylase 2 [OS=Homo sapiens]	1.589	0.001911622
Kelch-like protein 29 [OS=Homo sapiens]	1.594	0.269363869
G1/S-specific cyclin-E1 [OS=Homo sapiens]	1.595	7.06949E-05
Alpha-2-macroglobulin receptor-associated protein [OS=Homo sapiens]	1.598	8.10646E-05
ERO1-like protein alpha [OS=Homo sapiens]	1.603	2.55629E-06
FYVE and coiled-coil domain-containing protein 1 [OS=Homo sapiens]	1.604	7.10809E-06
Gamma-enolase [OS=Homo sapiens]	1.606	2.12932E-05
E3 ubiquitin-protein ligase RNF167 [OS=Homo sapiens]	1.608	0.053082847
Lipid droplet-associated hydrolase [OS=Homo sapiens]	1.617	0.734587823
Complement C5 [OS=Homo sapiens]	1.617	0.135111239
Selenoprotein M [OS=Homo sapiens]	1.632	0.014921382
Transcription factor HIVEP3 [OS=Homo sapiens]	1.632	0.440549648
Rho GTPase-activating protein 12 [OS=Homo sapiens]	1.633	1.55695E-05
LIM domain kinase 1 [OS=Homo sapiens]	1.663	0.000532633
LIM domain-containing protein 2 [OS=Homo sapiens]	1.663	0.001553017
Adaptin ear-binding coat-associated protein 1 [OS=Homo sapiens]	1.667	6.87337E-05
Testican-1 [OS=Homo sapiens]	1.667	0.136165811
N(G),N(G)-dimethylarginine dimethylaminohydrolase 1 [OS=Homo sapiens]	1.672	6.33681E-05
Solute carrier family 35 member F6 [OS=Homo sapiens]	1.677	0.146905143
Kinase suppressor of Ras 1 [OS=Homo sapiens]	1.684	0.570536011
Laminin subunit beta-2 [OS=Homo sapiens]	1.694	3.43102E-07
Multiple epidermal growth factor-like domains protein 10 [OS=Homo sapiens]	1.712	0.273593973
Pre-B-cell leukemia transcription factor-interacting protein 1 [OS=Homo sapiens	1.719	5.96233E-06
Natural resistance-associated macrophage protein 2 [OS=Homo sapiens]	1.737	0.163064947
Glucose 1,6-bisphosphate synthase [OS=Homo sapiens]	1.761	6.38264E-07
Cytochrome P450 26B1 [OS=Homo sapiens]	1.783	0.058703935
DnaJ homolog subfamily B member 9 [OS=Homo sapiens]	1.79	0.110433404
Phospholipid hydroperoxide glutathione peroxidase [OS=Homo sapiens]	1.804	0.000246674
Pseudouridine-5'-phosphatase [OS=Homo sapiens]	1.809	0.015218438
Cytosolic arginine sensor for mTORC1 subunit 1 [OS=Homo sapiens]	1.858	2.20552E-05
Neurofascin [OS=Homo sapiens]	1.881	0.048969486
Protein argonaute-2 [OS=Homo sapiens]	1.93	6.95548E-09
Keratin, type I cytoskeletal 14 [OS=Homo sapiens]	1.941	0.809562007
Coagulation factor X [OS=Homo sapiens]	1.948	0.196348666
Ubiquitin thioesterase OTU1 [OS=Homo sapiens]	1.964	0.003270178
Zinc finger CCHC domain-containing protein 3 [OS=Homo sapiens]	2.165	1.31335E-06
Integrin alpha-5 [OS=Homo sapiens]	2.187	1.31276E-07
Neurosecretory protein VGF [OS=Homo sapiens]	2.195	5.02631E-05
Endoribonuclease Dicer [OS=Homo sapiens]	2.294	3.60964E-08
Keratin, type II cytoskeletal 5 [OS=Homo sapiens]	2.386	0.981410422
Cyclin-dependent kinase inhibitor 1B [OS=Homo sapiens]	2.584	1.84982E-07
Solute carrier family 2, facilitated glucose transporter member 3 [OS=Homo sap	2.824	2.82852E-05
Semaphorin-4B [OS=Homo sapiens]	3.258	0.001128266

Supporting Table S1. Table showing list of proteins being up- and down-regulated following ISG15 loss in Flo1 cells. Flo1 cells were treated either with control or *ISG15* siRNAs as detailed in experimental methods section. Forty eight (48) hours post-transfection, cells were either sham radiated or irradiated 4 Gy. Samples were collected 6 hours post-irradiation and cell lysates were subjected to TMTpro analysis according to the manufacturer's protocol to identify altered protein expression using mass spectrometry. For each treatment group, three independent replicates were labeled with distinct isobaric tags to perform quantitative proteomic profiling. List of proteins showing statistically significant (P<0.001) difference in protein expression between irradiated Flo1 cells transfected with *ISG15* vs. control siRNA.
